# Supplementary material for: Thousandfold Expansion Microscopy
Source: bioRxiv. 2026 Jul 21:2026.05.31.729018. Originally published 2026 Jun 1. Preprint. [Version 2] doi: 10.64898/2026.05.31.729018 (PMC13252410; doi:10.64898/2026.05.31.729018)
Supplement: Supplement 1 [file NIHPP2026.05.31.729018v2-supplement-1.pdf]

bioRxiv preprint doi: https://doi.org/10.64898/2026.05.31.729018; this version posted July 21, 2026. The copyright holder for this preprint (which was not certified by peer review) is the author/funder, who has granted bioRxiv a license to display the preprint in perpetuity. It is made available under a CC-BY 4.0 International license.

Supplementary Figures

Supplementary Figure 1. Representative hydrogels at successive expansion stages.
The hydrogel consists of four interpenetrating polymer networks generated by sequential monomer infiltration, polymerization under deoxygenated conditions (>8 h, 20 °C), and re-expansion in water. Linear expansion was determined by measuring one gel edge with a ruler (minimum spacing 0.1 cm).
(A) First network (~18×). The primary swellable gel was cast from the monomer solution (**Fig. 1A**). At (i) 1×, the gel is colorless and transparent with white specks consistent with trapped air. After expansion in water to (ii) ~18×, the gel is transparent with straight edges and flat surfaces.
(B) Second network (~100× total). The fully expanded ~18× gel was infiltrated with a charged monomer solution and polymerized, yielding a composite gel that contracts to (i) ~10× linear expansion. At this stage, the gel is translucent with spherical bubbles. Re-expansion to (ii) ~100× (~10× from i) produces a transparent gel with straight edges and flat surfaces that stands upright without visible axial bending.
(C) Third network (~500× total). The fully expanded ~100× gel was infiltrated with a charged monomer solution and polymerized, yielding a composite gel that contracts to (i) ~50× linear expansion. The gel is translucent with spherical bubbles. Re-expansion to (ii) ~500× (~10× from i) produces a transparent gel with straight edges and flat surfaces that stands upright without visible axial bending.
(D) Fourth network (~1500× total). The fully expanded ~500× gel was infiltrated with a charged monomer solution and polymerized, yielding a composite gel that contracts to (i) ~250× linear expansion. Re-expansion to (ii) ~1500× (~6× from i) produces a transparent gel with slightly inward-curved (concave) surfaces, indicating resistance to further swelling, perhaps imposed by the covalently crosslinked network. (This did not seem to be gravity-induced: the gel remained rigid and self-supporting, with the slight inward curvature reflecting elastic constraint opposing further swelling, for this specific gel condition.) Upon equilibration in 1× PBS for imaging, the gel contracts to ~1000× linear expansion, and the slight concavity goes away. Furthermore, with optimization of the gelling process (**Supp. Fig. 31**), round 4 gels expand without any perceptible concavity.

Supplementary Figure 2. Nanobody imaging across different linear expansion factors. (A) ONE microscopy at ~18×, ~50×, and ~100× expansion, of representative fields of view from representative gels. (B) Confocal imaging at ~100×, ~500×, and ~1000× expansion. Representative datasets are shown, from 2-7 experimental replicates.

Supplementary Figure 3. mCLING peptide imaging across different linear expansion factors. (A–C) ONE microscopy at ~18×, ~50×, and ~100× expansion. (D–F) Confocal imaging at ~500×, ~750×, and ~1000× expansion.

Supplementary Figure 4. Pan-lysine anchoring, proteolytic cleavage, and post-expansion labeling illustrated using the mCLING peptide.
Gray boxes indicate reactions performed inside the expansion hydrogel; all other steps were performed in solution.
A. Chemical structure of the mCLING peptide.
B.i NHS-acrylate (Acryloyl-X) reacts with primary amines via NHS ester–amine coupling.
B.ii All primary amines on the mCLING peptide, ideally, are functionalized with polymerizable acrylate groups.
C. Hydrogel polymerization (gray box). Acrylate-functionalized amines (abbreviated A) are covalently attached to the gel network, anchoring the peptide at the labeled sites.

bioRxiv preprint doi: https://doi.org/10.64898/2026.05.31.729018; this version posted July 21, 2026. The copyright holder for this preprint (which was not certified by peer review) is the author/funder, who has granted bioRxiv a license to display the preprint in perpetuity. It is made available under a CC-BY 4.0 International license.

D. Proteinase K digestion in which the amide bond between the anchoring site and Atto647N is cleaved. All peptide backbone amide bonds are digested. Fragments lacking an anchoring site are removed during expansion. Because Atto647N is not anchored, that dye is lost.
E. Proteinase K digestion in which the amide bond between the anchoring site and Atto647N is not cleaved. Atto647N remains attached to an anchored fragment and is therefore retained and displaced during expansion according to the anchoring atom. Cleavage of this bond is unlikely due to steric constraints of the Atto647N substituent in the Proteinase K substrate recognition site [53].
F. NHS-ester fluorescein labeling of remaining free primary amines. The blue sphere denotes a primary amine that has been conjugated with fluorescein. In this panel, the full chemical conjugation product is shown.
G.I–III. Labeling outcomes for case D (Atto647N cleaved and removed).
In G and H, the blue sphere is used as a symbolic shorthand indicating an NH_2 group conjugated with fluorescein.
G.I Both the N-terminal amine and the lysine side-chain amine are anchored. No free primary amines remain; no fluorescein labeling occurs.
G.II Only the lysine side-chain amine is anchored. The free N-terminal amine is fluorescein-labeled (blue sphere). Upon expansion, the dye is displaced to the position of the lysine anchoring atom.
G.III Only the N-terminal amine is anchored. The lysine side-chain amine is fluorescein-labeled (blue sphere). The dye is displaced to the position of the N-terminal anchoring atom.
H.I–III. Labeling outcomes for case E (Atto647N retained).
Configurations are the same as G.I–III, except that Atto647N remains attached and is displaced according to its anchoring site.

Supplementary Figure 5. Distance distribution between adjacent lysine side chains.
Primary amines were functionalized with NHS–ester acrylate to covalently anchor the nitrogen atom to the polymer network. In the schematic, the nitrogen of the first primary amine bearing the Atto647N dye is shown as an orange sphere; adjacent lysine ε-amine nitrogens are shown as blue spheres. These nitrogen atoms are attached to the hydrogel and displaced during expansion. Thus, the measured positions of Atto647N and fluorescein correspond to the displaced coordinates of their respective anchoring nitrogens.
Inter-lysine distances were quantified by molecular dynamics.
(A) Distribution of distances (nm).
(B) Minimum distance (~0.44 nm), parallel side chains.
(C) Peak distance (~0.88 nm), staggered orientation.
(D) Larger distance (~1.41 nm), antiparallel orientation.

Supplementary Figure 6. Molecular dynamics (MD) simulation of k-nearest-neighbor (kNN) distance distributions for the mCLING peptide followed by analysis under defined anchoring, cleavage, and labeling scenarios.
Molecular dynamics (MD) simulations generated 1001 conformations of the mCLING peptide (see Methods: mCLING molecular dynamics simulations). All kNN distance distributions shown were computed across the full ensemble of 1001 conformations. Atomic model panels display the first conformation.
For each conformation, fluorophore positions were assigned to the atomic coordinates of gel-anchoring atoms defined in **Supplementary Figure 4**. Orange spheres denote Atto647N anchoring sites and blue spheres denote fluorescein anchoring sites. k-nearest-neighbor (kNN) distances were computed as Euclidean distances between anchoring-site atomic coordinates. In atomic model panels, arrows originate from a selected anchoring-site atom and point to its k-nearest neighboring sites; neighboring sites are numbered by increasing Euclidean distance, with labels 1–5 or 1–4 indicating the kNN rank for the representative

bioRxiv preprint doi: https://doi.org/10.64898/2026.05.31.729018; this version posted July 21, 2026. The copyright holder for this preprint (which was not certified by peer review) is the author/funder, who has granted bioRxiv a license to display the preprint in perpetuity. It is made available under a CC-BY 4.0 International license.

conformation. All quantitative distributions were computed using all 1001 conformations, with one distance per kNN rank per conformation.
Two terminal fluorescein labeling scenarios defined in **Supplementary Figure 4** were simulated: (A) terminal fluorescein assigned to a lysine side-chain amine (**Supplementary Fig. 4G.ii and 4H.ii**), and (B) terminal fluorescein assigned to the N-terminal amine (**Supplementary Fig. 4G.iii and 4H.iii**). In **Supplementary Figure 4**, panel G denotes configurations in which Atto647N is cleaved and removed, whereas panel H denotes configurations in which Atto647N is retained. (C) shows pooled distance distributions combining both terminal fluorescein labeling scenarios.

(A) Terminal fluorescein assigned to a lysine side-chain amine. (i) Representative atomic model illustrating the anchoring configuration corresponding to **Supplementary Fig. 4H.ii**, in which Atto647N is retained and the terminal fluorescein is assigned to a lysine side-chain amine. Arrows originate from the Atto647N anchoring-site atom and point to its k-nearest fluorescein neighbors (kNN ranks 1–5). (ii) Same model as (i), with arrows originating from each fluorescein anchoring-site atom in turn and pointing to its nearest fluorescein neighbors (kNN ranks 1–4). (iii) Representative atomic model for the corresponding Atto-cleaved configuration (**Supplementary Fig. 4G.ii**), in which Atto647N is removed and the newly generated N-terminus is labeled with fluorescein (kNN ranks 1–5). (iv) Atto647N→fluorescein kNN distance distributions (ranks 1–5) computed from the configuration in (i) across all 1001 conformations, with the Atto647N anchoring site queried against fluorescein-labeled sites (one distance per kNN rank per conformation). (v) Fluorescein→fluorescein kNN distance distributions (ranks 1–4) computed from the same anchoring configuration as in (iv), using only fluorescein-labeled sites. (vi) Fluorescein→fluorescein kNN distance distributions (ranks 1–5) computed from the same anchoring geometry as in (iv), after treating the Atto647N anchoring-site coordinate as a fluorescein site for analysis. This corresponds to the Atto-cleaved case in which the newly generated N-terminus is labeled with fluorescein while the anchoring atom remains unchanged, such that the underlying anchoring-site coordinates are identical. (vii–ix) Five-choose-four subsampling was used to reflect molecule-to-molecule variation in which anchoring and labeling sites are present after anchoring, proteolysis, and post-expansion labeling—including outcomes such as **Supplementary Fig. 4G.i and 4H.i**, in which both the N-terminal amine and a lysine side-chain amine are anchored. All subsets of four labeling-site coordinates were selected from the five available sites for each peptide conformation. (vii) Atto647N→fluorescein kNN distance distributions computed from five-choose-four subsampled fluorescein site sets in Atto-retained configurations. (viii) Fluorescein→fluorescein kNN distance distributions computed from the same five-choose-four subsampled fluorescein site sets as in (vii). (ix) Fluorescein→fluorescein kNN distance distributions computed using five-choose-four subsampling in Atto-cleaved configurations, with the former Atto647N anchoring-site coordinate treated as a fluorescein site for analysis.

(B) Terminal fluorescein assigned to the N-terminal amine. (i–ix) Same anchoring configurations, kNN definitions, and five-choose-four subsampling procedures described in (A)(i–ix), applied to configurations in which the terminal fluorescein is assigned to the N-terminal amine (**Supplementary Fig. 4G.iii and 4H.iii**).

(C) Combined distance distributions. (i) Combined Atto647N→fluorescein kNN distance distributions pooled across both terminal fluorescein labeling scenarios for configurations in which Atto647N is retained (**Supplementary Fig. 4H**). (ii) Combined fluorescein→fluorescein kNN distance distributions pooled across both terminal fluorescein labeling scenarios for configurations in which Atto647N is retained (**Supplementary Fig. 4H**). (iii) Combined fluorescein→fluorescein kNN distance distributions pooled across both terminal fluorescein labeling scenarios for configurations in which Atto647N is cleaved (**Supplementary Fig. 4G**).

Supplementary Figure 7. 3D regions containing putative mCLING peptides after ~1000× expansion.

bioRxiv preprint doi: https://doi.org/10.64898/2026.05.31.729018; this version posted July 21, 2026. The copyright holder for this preprint (which was not certified by peer review) is the author/funder, who has granted bioRxiv a license to display the preprint in perpetuity. It is made available under a CC-BY 4.0 International license.

Three-dimensional renderings of six regions selected to be representative with respect to signal brightness within a 10 × 20 μm field spanning the full z depth (4.4 μm) after ~1000× linear expansion. Boxed regions highlight examples of putative mCLING peptides, defined as kNN-derived clusters containing one Atto647N signal (magenta) and at least four proximal fluorescein signals (green). Boxes are shown for illustrative examples and do not mark all such clusters within the field. Because mCLING peptides contain a hydrophobic palmitoyl tail, intermolecular interactions may occur, and some boxed regions may contain more than one putative peptide due to spatial overlap; in cases of overlap, one box was randomly selected.

Supplementary Figure 8. RMSD of coarse putative peptides before nearest-neighbor filtering.
From 27,426 Atto647N localizations (three gels, eight regions), fluorescein signals were associated with the nearest Atto647N, yielding 10,696 coarse putative peptides (see Methods, Identification of putative peptides). Each contains one Atto647N and four or five fluoresceins; assignments may include signals from neighboring peptides.
For each coarse putative peptide, observed coordinates were aligned to a 1,001-conformation molecular-dynamics ensemble spanning anchoring, digestion, and labeling outcomes (**Supplementary Figs. 4, 6**), and RMSD was computed. Boxplots show the median, IQR, whiskers extending to the farthest data point within 1.5×IQR of each box edge, and outliers. (A) All gels. (B) Individual gels. (C) Individual regions.

Supplementary Figure 9. RMSD of putative peptides after nearest-neighbor filtering.
Nearest-neighbor filtering (see Methods; filtered subset of **Supplementary Fig. 8**; same peptide set as **Fig. 3C**) yields the final putative peptides. RMSD was computed by alignment to the molecular-dynamics ensemble and shown as boxplots.
(A) All gels. (B) By gel. (C) By region.
Summary statistics: Table S13 (A), Table S14 (B), Table S15 (C).

Supplementary Figure 10. Per-axis RMSD of putative peptides (same peptide set as Fig. 3C).
Per-axis RMSD (x, y, z) was computed using the same analysis as Supplementary Fig. 9. Fig. 3C shows pooled data; here the same data are shown pooled, by gel, and by region to assess variation.
Summary statistics: Table S13 (A), Table S14 (B), Table S15 (C).

Supplementary Figure 11. Single-molecule expansion factors (same peptide set as Fig. 3C).
An isotropic expansion factor was estimated for each peptide by minimizing RMSD to the molecular-dynamics ensemble, using the same analysis as Supplementary Fig. 9. Fig. 3C shows pooled data; here the same data are shown pooled, by gel, and by region to assess variation.
(A) All gels combined. (B) By gel. (C) By region.
Summary statistics: Table S13 (A), Table S14 (B), Table S15 (C).

Supplementary Figure 12. RMSD histograms (same peptide set and RMSD values as Fig. 3C).
The same RMSD values are replotted as histograms with interquartile ranges; only the visualization differs. Fig. 3C shows pooled data; here the same data are shown pooled, by gel, and by region to assess variation.
(A) All data combined. (B) By gel. (C) By region.
Summary statistics: Table S13 (A), Table S14 (B), Table S15 (C).

Supplementary Figure 13. Robustness of RMSD to MD ensemble size.
Using the filtered putative peptides defined in Supplementary Fig. 9, total RMSD was recomputed after alignment to MD reference ensembles containing 10–1,001 conformations. For each ensemble size, RMSD

bioRxiv preprint doi: https://doi.org/10.64898/2026.05.31.729018; this version posted July 21, 2026. The copyright holder for this preprint (which was not certified by peer review) is the author/funder, who has granted bioRxiv a license to display the preprint in perpetuity. It is made available under a CC-BY 4.0 International license.

distributions are summarized as boxplots (median, IQR, whiskers to farthest data points within 1.5×IQR of box edges, outliers).
Summary statistics are reported in **Table S16**.

Supplementary Figure 14. RMSD histograms across MD ensemble sizes.
For the same peptides and RMSD values analyzed in Supplementary Fig. 13, distributions are replotted as histograms to visualize distribution shape as a function of MD ensemble size. Panels correspond to MD ensemble sizes: (A) 10; (B) 100; (C) 200; (D) 300; (E) 400; (F) 500; (G) 600; (H) 700; (I) 800; (J) 900; (K) 1,001 conformations.
Summary statistics are reported in **Table S16.**

Supplementary Figure 15. Peptide overlays below the IQR of the Fig. 3C.i RMSD distribution.
Putative peptides with total RMSD below the lower quartile of **Fig. 3C.i** are shown. Fluorophore coordinates are overlaid after optimal alignment to the molecular-dynamics ensemble (**Supplementary Fig. 6**). Peptide length reflects anchoring, cleavage, and labeling outcomes (**Supplementary Fig. 4**), yielding four or five fluorescein sites.
(A) 1 Atto647N followed by 5 fluoresceins.
(B) 1 Atto647N followed by 4 fluoresceins.

Supplementary Figure 16. Peptide overlays within the IQR of the Fig. 3C.i RMSD distribution.
Same analysis and representation as Supplementary Fig. 15, for peptides with RMSD within the interquartile range of Fig. 3C.i.

Supplementary Figure 17. Peptide overlays above the IQR of the Fig. 3C.i RMSD distribution.
Same analysis and representation as Supplementary Fig. 15, for peptides with RMSD above the upper quartile of Fig. 3C.i.

Supplementary Figure 18. Emitter detection using Gaussian correlation to identify true fluorescent emitters.
Confocal images were acquired as described in Methods (confocal z-stack acquisition). All images shown are raw, with no background subtraction or additional processing. Candidate fluorescent emitters were filtered by intensity and size and evaluated by Gaussian correlation (Methods, 3D emitter detection), using a correlation coefficient >0.70 as the acceptance threshold.
Each subpanel (A.i–A.iii, B.i–B.iii) shows three representative emitters. For each emitter, a central z-slice image is displayed with the emitter center marked by a cross. From this center, one diagonal line scan (upper left to lower right) is shown, along with 3 additional line scans: upper right to lower left, vertical (top to bottom), and horizontal (left to right). Intensity profiles from these 4 line scans are plotted, and their average is shown as a bold trace. In the line-scan plots, the x-axis denotes distance along the line scan and the y-axis denotes fluorescence intensity (arbitrary units).
(A) Atto647N emitters.
(A.i) Accepted emitters with high Gaussian correlation (≈0.9), drawn from 27,426 Atto647N emitters across three gels and eight regions.
(A.ii) Accepted emitters near the Gaussian-correlation threshold (≈0.7), drawn from the same 27,426 Atto647N emitters.
(A.iii) Rejected emitters with Gaussian correlation <0.7, drawn from the 21,705 emitters that failed the Gaussian-correlation threshold after intensity and size filtering (49,131 total candidates across three gels and eight regions).

bioRxiv preprint doi: https://doi.org/10.64898/2026.05.31.729018; this version posted July 21, 2026. The copyright holder for this preprint (which was not certified by peer review) is the author/funder, who has granted bioRxiv a license to display the preprint in perpetuity. It is made available under a CC-BY 4.0 International license.

(B) Fluorescein emitters.
(B.i) Accepted emitters with high Gaussian correlation (≈0.9), drawn from 125,395 fluorescein emitters across three gels and eight regions.
(B.ii) Accepted emitters near the Gaussian-correlation threshold (≈0.7), drawn from the same 125,395 fluorescein emitters.
(B.iii) Rejected emitters with Gaussian correlation <0.7, drawn from the 462,408 emitters that failed the Gaussian-correlation threshold after intensity and size filtering (587,803 total candidates across three gels and eight regions).

Supplementary Figure 19. k-nearest-neighbor (kNN) 1–5 distance distributions for Atto647N–fluorescein (magenta) and fluorescein–fluorescein (green).
Histograms use 0.05 μm bins with Gaussian smoothing (σ = 1 bin). Peaks are defined as the maxima of the smoothed distributions. n denotes the number of distances per kNN rank. Summary distance statistics corresponding to each panel are reported in **Tables S5–S8**.
A) All gels combined (3 gels, 8 regions): Atto647N–fluorescein, n = 27,426; fluorescein–fluorescein, n = 125,395.
(See pooled distance statistics in **Table S5**.)
B) Partitioned by four-round gelation experiment (biological replicates); each ~1000× gel was generated by an independent four-network casting sequence, initiated from an independently cast first-round ~18× gel.
B.i) Gel 1 (2 regions): Atto647N–fluorescein, n = 9,266; fluorescein–fluorescein, n = 63,733 (statistics in **Table S6**).
B.ii) Gel 2 (4 regions): Atto647N–fluorescein, n = 11,671; fluorescein–fluorescein, n = 32,765 (statistics in **Table S7**).
B.iii) Gel 3 (2 regions): Atto647N–fluorescein, n = 6,489; fluorescein–fluorescein, n = 28,897 (statistics in **Table S8**).

Supplementary Figure 20. Randomization analysis of k-nearest-neighbor (kNN) distance distributions, all gels.
Each region is a three-dimensional imaging volume containing Atto647N and fluorescein emitters, each represented by an (x, y, z) coordinate. For the randomized control, emitter counts were preserved and all coordinates were randomly chosen within the same volume. kNN distance distributions were recomputed from the randomized coordinates. Histograms use 0.05 μm bins with Gaussian smoothing (σ = 1 bin). Peak positions are defined as the bin center corresponding to the global maximum of the smoothed distribution. Distances are aggregated across eight regions from three gels. Observed Atto647N–fluorescein distributions are shown in magenta with randomized controls in gray (n = 27,426 distances per kNN rank). Observed fluorescein–fluorescein distributions are shown in green with randomized controls in gray (n = 125,395 distances per kNN rank). Panels A–E show kNN ranks 1–5. Panel F shows robustness across 100 randomizations assessed using two-sample Kolmogorov–Smirnov tests (***, $p < 0.001$); KS D statistics report effect size. Corresponding Kolmogorov–Smirnov D statistics by kNN rank (1–5) are reported in **Table S9**.

Supplementary Figures 21–23 show the same randomization test as Supplementary Figure 20, with distances aggregated at the level of individual gels (statistics in Tables **S10-12**).

Supplementary Figure 21. Randomization analysis of k-nearest-neighbor (kNN) distance distributions for gel 1 (statistics in Table S10).

Supplementary Figure 22. Randomization analysis of k-nearest-neighbor (kNN) distance distributions for gel 2 (statistics in Table S11).

bioRxiv preprint doi: https://doi.org/10.64898/2026.05.31.729018; this version posted July 21, 2026. The copyright holder for this preprint (which was not certified by peer review) is the author/funder, who has granted bioRxiv a license to display the preprint in perpetuity. It is made available under a CC-BY 4.0 International license.

Supplementary Figure 23. Randomization analysis of k-nearest-neighbor (kNN) distance distributions for gel 3 (statistics in Table S12).

Supplementary Figure 24. Preprocessing and transformer-based feature extraction pipeline for microscopy images. Raw fluorescence microscopy images are first partitioned into fixed-size patches. Each patch is flattened into a one-dimensional vector and supplemented with positional encodings to retain spatial information. The resulting sequence of embedded patches is processed by a transformer encoder, which integrates local texture features with global spatial relationships. The output is a structured feature map that serves as input for downstream graph-based modeling.

Supplementary Figure 25. Graph formulation from transformer-derived patch features. Each node corresponds to a transformer-derived patch feature. Pairwise similarities between feature vectors define edge weights, forming a weighted, undirected graph G(V, E). Edges are retained only when their similarity exceeds a predefined threshold. The resulting symmetric adjacency matrix A(i, j) encodes structural relationships across the image and enables graph-based segmentation and clustering.

Supplementary Figure 26. **Unsupervised graph-based segmentation framework integrating Normalized Cut and correlation clustering losses.** Node features are iteratively updated through aggregation, propagation, and normalization steps to refine graph embeddings. The training objective combines the Normalized Cut (NCut) loss and a correlation clustering loss to promote coherent partitioning. A global max pooling layer aggregates node-wise information, followed by a fully connected layer and softmax classifier that assign each node to foreground or background. The combined loss enforces consistent separation of structurally similar regions within the graph.

Supplementary Figure 27. **Post-segmentation analysis for separation of heterogeneous foreground objects.** Following foreground–background separation, connected components are extracted from the primary mask. Objects are filtered using geometric and intensity-based descriptors, including eccentricity, aspect ratio, extent, and intensity criteria. Partitioning around medoids clustering is subsequently applied to group objects of similar identity. Decision boundaries delineate the resulting clusters, and bounding boxes indicate individual, physically plausible particle instances.

Supplementary Figure 28. **End-to-end *ab initio* 3D reconstruction pipeline from preprocessed 2D images.** The workflow begins with segmented images and preprocessing steps including intensity normalization, background suppression, Gaussian smoothing, and total variation–based enrichment. Preprocessed inputs are mapped into a Fourier domain representation and encoded using a convolutional variational autoencoder (VAE). The encoder performs amortized inference to learn latent variables under a combined objective incorporating KL divergence and symmetry loss, followed by affine transformations in the decoder to generate consistent reconstructions. Reconstructed 2D slices are stacked and integrated in the spatial domain to produce a volumetric density map V(u, v, w), yielding an ab initio 3D molecular model (**Fig. 4**).

Supplementary Figure 29. Additional GFP examples at different expansion factors. Representative 2D ONE GFP images are shown at (A) 50-fold expansion and (B) 100-fold expansion.

Supplementary Figure 30. GFP imaged with ONE microscopy across expansion factors.
Representative GFP images at (A) 18-fold, (B) 50-fold, and (C) 100-fold linear expansion. Each panel shows a field-of-view overview and a corresponding magnified region from the same image.

bioRxiv preprint doi: https://doi.org/10.64898/2026.05.31.729018; this version posted July 21, 2026. The copyright holder for this preprint (which was not certified by peer review) is the author/funder, who has granted bioRxiv a license to display the preprint in perpetuity. It is made available under a CC-BY 4.0 International license.

Supplementary Figure 31. Four-network gels generated using a modified 1000ExM process. In a modified 1000ExM process, each sequential casting round includes two incubations in activated monomer solution before polymerization. The second incubation replaces the monomer-depleted and/or diluted first solution with fresh activated monomer solution, for further infiltration.

(A) Schematic showing how per-round shrinkage factors, per-round expansion factors, and cumulative linear expansion factors were calculated during four-network gel formation. After the first gel was expanded, each subsequent casting round included incubation in activated monomer solution (two rounds), polymerization of the next network, measurement of the fully gelled composite, and re-expansion in water. Per-round shrinkage factors were measured after full gelation and calculated as gel length after polymerization divided by gel length before monomer solution addition and polymerization (i.e., at the end of the last expansion round). Per-round expansion factors were calculated as gel length after water expansion divided by gel length before water expansion. Cumulative linear expansion factors were calculated by multiplying the sequential shrinkage and expansion factors through all rounds up to the current round.

(B) Per-round shrinkage factors for rounds 2–4. Boxplots (throughout this figure) show the median (middle line), interquartile range (top and bottom of box), whiskers extending to the farthest data point within 1.5×IQR of each box edge, and individual gel measurements as open circles.

(C) Per-round expansion factors for rounds 1–4. Note that some circles have similar or identical values - this is not an error, and we include all the raw data in Table S18.

(D) Cumulative linear expansion factors after each expansion round, with round 4 gels reaching up to 2534× linear expansion.

(E) Images of fully expanded round 4 gels.

(F) Images of round 4 gels (made at the same time) maintained at room temperature on a dry surface for 98 hours (chosen arbitrarily). Gel linear size and height remained roughly unchanged over this period, with linear measurements showing a 0.3±0.5% change in expansion factor.

bioRxiv preprint doi: https://doi.org/10.64898/2026.05.31.729018; this version posted July 21, 2026. The copyright holder for this preprint (which was not certified by peer review) is the author/funder, who has granted bioRxiv a license to display the preprint in perpetuity. It is made available under a CC-BY 4.0 International license.

Supplementary Tables

Table S1: x, y, and z dimensions and total volume of the eight imaged 3D regions.

Table S2: Atto647N and fluorescein emitter counts after local maxima detection.

Table S3: Atto647N and fluorescein emitter counts after local maxima detection and intensity- and size-based filtering

Table S4: Atto647N and fluorescein emitter counts after local maxima detection, intensity- and size-based filtering, and Gaussian shape filtering

Table S5. Pooled k-nearest neighbor (kNN) distance statistics (k = 1–5) across all gels and all regions (see **Supplementary Figure 19A).**

Table S6. Pooled k-nearest neighbor (kNN) distance statistics (k = 1–5) across all regions from gel 1 (see **Supplementary Figure 19B.i**).

Table S7. Pooled k-nearest neighbor (kNN) distance statistics (k = 1–5) across all regions from gel 2 (see **Supplementary Figure 19B.ii**).

Table S8. Pooled k-nearest neighbor (kNN) distance statistics (k = 1–5) across all regions from gel 3 (see **Supplementary Figure 19B.iii**).

Table S9: KS D randomization statistics by kNN rank (1–5) from all gels (see **Supplementary Figure 20**).

Table S10: KS D randomization statistics by kNN rank (1–5) from gel 1 (see **Supplementary Figure 21**).

Table S11: KS D randomization statistics by kNN rank (1–5) from gel 2 (see **Supplementary Figure 22**).

Table S12: KS D randomization statistics by kNN rank (1–5) from gel 3 (see **Supplementary Figure 23**).

Table S13: Summary statistics of single-molecule expansion factors (**Supplementary Figure 11A**), per-axis RMSD (**Supplementary Figure 10A**), and total RMSD (see **Supplementary Figure 9A**, boxplots; **Supplementary Figure 12A**, histograms) for all putative peptides pooled across all gels and regions.

Table S14: Summary statistics of single-molecule expansion factors (**Supplementary Figure 11B**), per-axis RMSD (**Supplementary Figure 10B**), and total RMSD (see **Supplementary Figure 9A**, boxplots; **Supplementary Figure 12A**, histograms) for all putative peptides, stratified by gel (Gel 1-3).

Table S15: Summary statistics of single-molecule expansion factors (**Supplementary Figure 11C**), per-axis RMSD (**Supplementary Figure 10C**), and total RMSD (see **Supplementary Figure 9C**, boxplots; **Supplementary Figure 12C**, histograms) for all putative peptides, stratified by imaging region across Gel 1-3 (n=8 regions).

bioRxiv preprint doi: https://doi.org/10.64898/2026.05.31.729018; this version posted July 21, 2026. The copyright holder for this preprint (which was not certified by peer review) is the author/funder, who has granted bioRxiv a license to display the preprint in perpetuity. It is made available under a CC-BY 4.0 International license.

Table S16. Summary statistics of total RMSD distributions as a function of the number of molecular-dynamics (MD) reference conformations (see **Supplementary Figure 13**, boxplots; **Supplementary Figure 14**, histograms).

Table S17: Statistics elements for **Fig. 2**.

Table S18. Measured gel lengths and calculated shrinkage and expansion factors during four-network gel formation. For each round, the table reports lengths before and after expansion, per-round expansion factors, and cumulative linear expansion factors. For rounds 2–4, the table also reports lengths before monomer addition and after gelation, which were used to calculate per-round shrinkage factors. Cumulative linear expansion factors were calculated by multiplying all sequential shrinkage and expansion factors through the indicated round.

bioRxiv preprint doi: https://doi.org/10.64898/2026.05.31.729018; this version posted July 21, 2026. The copyright holder for this preprint (which was not certified by peer review) is the author/funder, who has granted bioRxiv a license to display the preprint in perpetuity. It is made available under a CC-BY 4.0 International license.

Supplementary Notes

Supplementary Note 1: 1000ExM Protocol, Updated

Activated monomer solution preparation

Reagent	Amount in 620 µL Activated Monomer Solution (mol)	Concentration (M)	Percentage
Sodium Acrylate (AK Scientific, R624)	1.69×10^{-3} mol	2.73 M	25.6% (w/v)
N,N-Dimethylacrylamide (DMAA; ThermoFisher, 432021000)	2.60×10^{-3} mol	4.19 M	44.2% (v/v)
N,N,N′,N′-Tetramethylethylenediamine (TEMED; Sigma, T9281)	1.52×10^{-6} mol	2.45 mM	0.037% (v/v)
Potassium persulfate (KPS; Sigma, 216224)	3.33×10^{-6} mol	5.37 mM	0.145% (w/v)

1. Weigh 3.14 g sodium acrylate into a 50 mL Eppendorf tube and dissolve it in 6 mL acidified Tris buffer, prepared from: 1 M Tris pH 8.0 (ThermoFisher, #AM9856), 32% HCl solution (Sigma, #W530574), and water. (To make 50 mL of acidified Tris, add 40 mL water, then 5.00 mL 1 M Tris pH 8.0, then add 1.18 mL 32% HCl, and bring the final volume to 50.0 mL with water.) Vortex the acrylate solution for approximately 5 to 10 minutes, until the solution is completely clear.
2. Add 5.4 mL N,N-dimethylacrylamide to the sodium acrylate solution and vortex for less than 1 minute.
3. Prepare a 10% N,N,N′,N′-tetramethylethylenediamine solution in water. Add 45 µL of this solution to the above mixture and mix again.
4. Check that the solution is clear and free of precipitate. If precipitate is present (possible for some batches of sodium acrylate), filter the solution using a syringe filter (Sigma, #SLGSR33SS) and collect the clear filtrate.
5. Degas the solution with nitrogen gas at room temperature (~20°C) using a Pasteur pipette connected to a nitrogen source (Sigma, #BR747725). Place the tip of the pipette at the bottom of the 50 mL tube and bubble for 20 minutes at a low-to-moderate flow rate sufficient to produce steady bubbling without splashing.
6. Pipette 600 µL of the degassed monomer solution into an Eppendorf tube. Add 20 µL potassium persulfate solution (45 mg/mL stock in water) to activate polymerization (which will proceed slowly). The resulting mixture is the activated monomer solution.

Note: These volumes yield enough activated monomer solution for 9 iterative gels (620 µL per gel, applied twice, 1240 µL total per gel), incubated in 12-well plastic plates (do not use glass plates, as this seems to cause premature gelation).

To make the first gel:

7. Prepare the sealable plastic container (Rubbermaid 9.6 Cup Brilliance Food Storage Container) used to hold the gel chamber. Modify the lid to contain two small holes: one inlet and one outlet. Confirm the container does not leak by sealing both holes with tape, adding water to the container, and checking whether any water escapes; if leaks are found, do not proceed.
8. Use the lid of the 12-well plate as a small platform to hold the hydrophobic microscope slides during gelation. Add 30 to 50 µL of activated monomer solution onto a hydrophobic microscope slide (CytoSlide Fluorosilane, CYTONIX); the volume used depends on the desired gel thickness. Place a 1.2 mm coverslip with the attached sample (proteins or cells) on top of the monomer solution, biological

bioRxiv preprint doi: https://doi.org/10.64898/2026.05.31.729018; this version posted July 21, 2026. The copyright holder for this preprint (which was not certified by peer review) is the author/funder, who has granted bioRxiv a license to display the preprint in perpetuity. It is made available under a CC-BY 4.0 International license.

sample face down, with no spacer. Together, the slide, monomer solution, and coverslip form the gel chamber. Place the gel chamber, still resting on the lid platform, inside the prepared container. Insert a pipette tip, connected via rubber tubing to a nitrogen line, into the inlet hole to deliver nitrogen gas, and leave the second hole open as a vent to allow air to escape as nitrogen fills the container.

9. Purge the container with nitrogen on high for 20 minutes at room temperature to displace oxygen, making sure the nitrogen stream is not blowing directly at the gel. No supplemental moisture was added to the Tupperware in this or any other step. After purging, remove the nitrogen line and seal both holes with tape to maintain a nitrogen atmosphere. Allow the gel to polymerize overnight (8-16 hours) at room temperature.
10. Immerse the gel in excess deionized water at room temperature (~20 °C) for 3 hours to remove unreacted components and allow expansion. Replace the water at least twice, continuing exchanges as needed until full linear expansion of approximately 15-18× is reached.

To form the next network within an already-expanded gel (all steps, unless otherwise noted, are performed at room temperature):

11. Cut each expanded gel into pieces up to 2 cm in length and approximately 1 cm in width, on the lid of a 12-well plate (see photo below), with an asymmetric corner cut to mark which side the sample is on. Then, place each gel piece into an individual well of a 12-well plate.

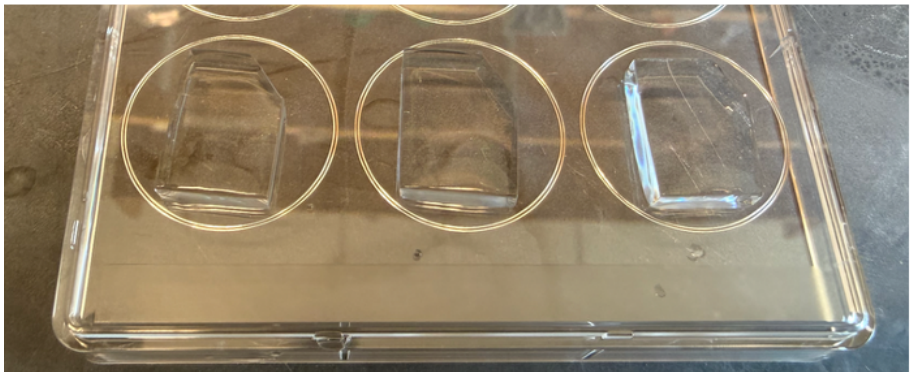

12. Add 600 μL degassed monomer solution and 20 μL potassium persulfate solution (45 mg/mL stock) to each well to make the activated monomer solution. Position the plate at approximately a 45° angle in a Tupperware container so that the gels are fully immersed from the top, bottom, and sides (critical). Insert a pipette tip, connected via rubber tubing to a nitrogen line, into the inlet hole to deliver nitrogen gas, and leave the second hole open as a vent to allow air to escape as nitrogen fills the container. Leave the nitrogen on and shake for 20 minutes.
13. Prepare a new 12-well plate with 600 μL monomer solution and 20 μL KPS solution in each well. Transfer the gels into the new solution, keeping the nitrogen on, and shake for another 20 minutes.
14. Remove the gels quickly. Place each gel onto a hydrophobic glass slide with the sample facing down, and quickly cover it with a coverslip 12mm in diameter. No spacer and no backfilling were used.
15. Purge the Tupperware with nitrogen gas for 1 hour, then allow the gel to polymerize overnight (≥8 hours).
16. After gelation is complete, measure the linear dimensions of each gel. The shrinkage factor for each round can be determined by measuring gel size after polymerization, which typically shrinks from 2 cm to approximately 1 to 1.2 cm. Example:

bioRxiv preprint doi: https://doi.org/10.64898/2026.05.31.729018; this version posted July 21, 2026. The copyright holder for this preprint (which was not certified by peer review) is the author/funder, who has granted bioRxiv a license to display the preprint in perpetuity. It is made available under a CC-BY 4.0 International license.

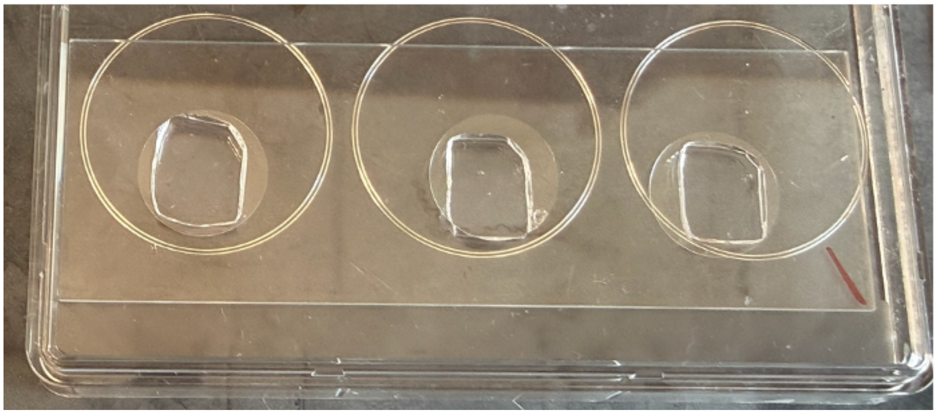

17. Use a paintbrush dipped in 1x PBS to detach the gel from the glass, then cut away the edges of the gel.

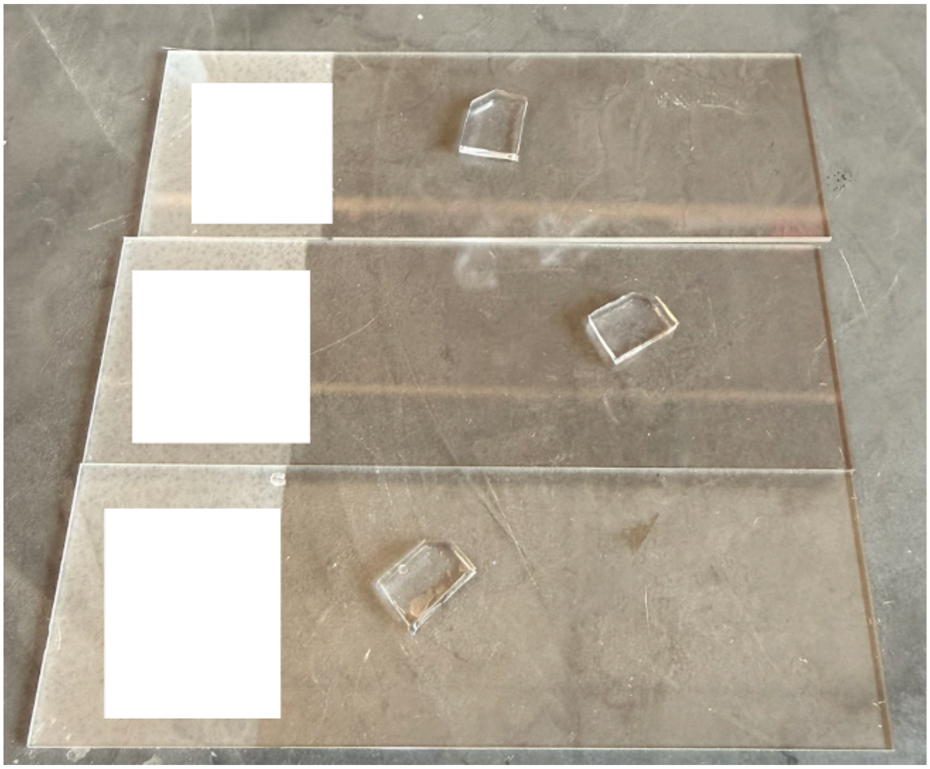

18. Place each gel piece (less than 1 cm in length) in a 140 mm petri dish and incubate in water, filled to the top of the dish, until expansion reaches equilibrium, typically 24 to 48 hours. Measure the final length and height of the gel to determine the expansion factor for each round. The gel typically expands approximately 10-fold (for example, from 0.8 cm to 8 cm). No intermediate water exchanges are required.

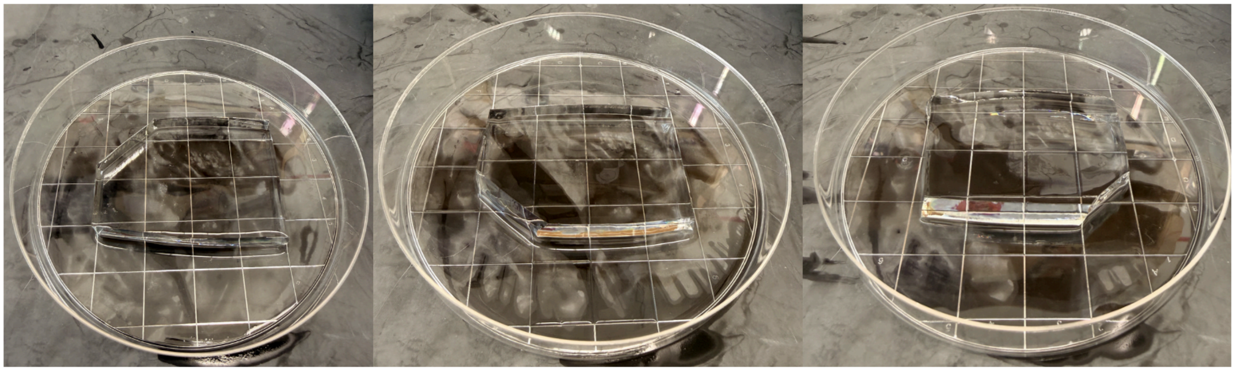

bioRxiv preprint doi: https://doi.org/10.64898/2026.05.31.729018; this version posted July 21, 2026. The copyright holder for this preprint (which was not certified by peer review) is the author/funder, who has granted bioRxiv a license to display the preprint in perpetuity. It is made available under a CC-BY 4.0 International license.

19. After round 2 (typically 60 to 100×), axially section the gel, sample side facing down, to generate thinner gel pieces for the next casting round. To prepare a slicing guide, glue (using superglue) three microscope slides together to make a 3 mm thick spacer, and prepare two such spacers. Place one spacer on either side of the gel, rest a long razor blade (Leica microtome blades, Electron Microscopy Sciences #63065-LP) on top of the spacers, and slide it through the gel to produce an approximately 3 mm thick axial section. Use these 3 mm thick gel sections for the subsequent iterative gel-casting round. Shown are the sectioning chamber (left) and two pieces of gel, sectioned on (right).

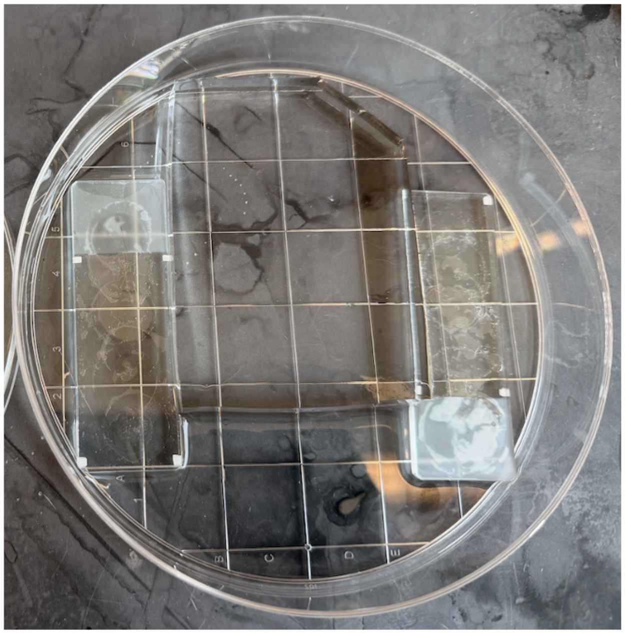

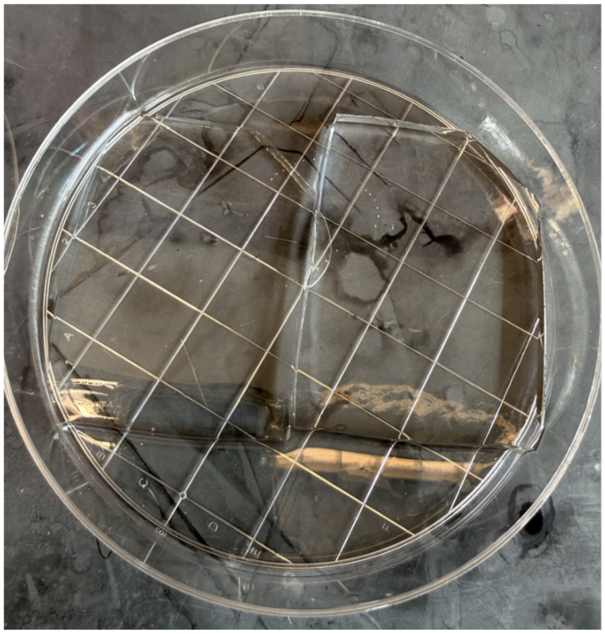

20. Cut the 3 mm thick gel into pieces 2 cm in length to repeat the iterative gelation steps and form the round 3 gels (typically 250 to 500×). Similarly, after axially slicing the fully expanded round 3 gels, cut them into 2 cm pieces to form the round 4 gels (typically 1000 to 2500×).

Supplementary Note 2: 1000ExM Protocol, Original

To prepare the activated monomer solution:

1. Weigh 2.090 ± 0.002 g of sodium acrylate and dissolve it in 4 mL of acidified Tris buffer prepared as described in Supplementary Note 1.
2. Add 3.600 mL N,N-dimethylacrylamide to the sodium acrylate solution and mix thoroughly.
3. Prepare a 10% solution of N,N,N′,N′-tetramethylethylenediamine (90 µL water + 10 µL pure compound). Add 30 µL of this solution to the above mixture and mix again until fully incorporated.
4. Check if the solution is precipitate free. Otherwise, filter the solution using a syringe filter (Sigma, #SLGSR33SS) and collect the clear supernatant.
5. Bubble the solution with nitrogen gas using a Pasteur pipette directly connected to a nitrogen source (Sigma, #BR747725) at room temperature (~20 °C) for 15 min using a low-to-moderate flow rate sufficient to produce steady bubbling without splashing.
6. Pipette 900 µL of the degassed monomer solution into an Eppendorf tube.
7. Add 30 µL potassium persulfate solution (45 mg/mL stock in water). The resulting solution is the activated monomer solution.

Note: These volumes yield enough activated monomer solution for 6 iterative gels (930 µL per gel), incubated in 6 well plastic plates.

bioRxiv preprint doi: https://doi.org/10.64898/2026.05.31.729018; this version posted July 21, 2026. The copyright holder for this preprint (which was not certified by peer review) is the author/funder, who has granted bioRxiv a license to display the preprint in perpetuity. It is made available under a CC-BY 4.0 International license.

To make the first gel:

8. Add 50 µL activated monomer solution onto a hydrophobic microscope slide (CytoSlide Fluorosilane, CYTONIX) in a Tupperware container.
9. Place a coverslip with the attached sample (proteins or cells) on top, with the biological sample face down (no spacer).
10. Place the gel chamber inside a sealable plastic container (e.g., Tupperware). Before beginning any procedures, modify the lid to contain two small holes: one inlet and one outlet. Insert a pipette tip (connected via rubber tubing to a nitrogen line) into the inlet hole to deliver nitrogen gas. Leave the second hole open to serve as a vent, allowing air to escape as nitrogen fills the container.
11. Purge with nitrogen for 1 hour to displace oxygen at room temperature. (No supplemental moisture was added to the Tupperware, in this or any other step.) After purging, remove the nitrogen line and seal both holes with tape to maintain a nitrogen atmosphere. Allow the gel to polymerize overnight (≥8 hours) at room temperature.
12. Immerse the gel in excess deionized water at room temperature (~20 °C) for 3 hours to remove unreacted components and allow expansion. Replace the water at least twice, continuing exchanges as needed until full ~18× linear expansion is reached.

To make an iterative gel throughout an expanded gel (all steps unless otherwise noted are done at room temperature):

13. Place each expanded gel piece into an individual well of a 6-well plate. Before doing so, cut the gel into pieces up to approximately 3 cm in length and 1.5 cm in width.
14. Add 900 µL degassed monomer solution and 30 µL potassium persulfate solution (45 mg/mL stock) to each well to make the activated monomer solution.
15. Position the plate at approximately 45° angle in a Tupperware container so that gels are fully immersed from the top, bottom, and sides (critical).
16. Degas the Tupperware with nitrogen gas for 10 minutes, then place on a shaker for 35 minutes to incubate.
17. Remove gels quickly. Place each gel onto a hydrophobic glass slide with the sample facing down and quickly cover with a coverslip (within 30 seconds) to minimize oxygen exposure. No spacer, and no backfilling, were performed.
18. Purge the Tupperware with nitrogen gas for 1 hour, then allow the gel to form overnight (≥8 hours).
19. After gelation is complete, measure the linear dimensions of each gel. To determine the shrinkage factor for each round, compare the gel size immediately after transfer to the 6-well plate with the gel size after gelation is complete.
20. Then incubate the gel in water until the expansion reaches equilibrium, typically 24–48 h. Measure the final linear dimensions to determine the expansion factor for each round. Measure the gel along multiple edges, as well as the gel height, to assess expansion in all dimensions.
21. To reach the maximum iterative expansion capacity, perform up to three additional rounds of iterative gel casting after the initial gel. The second, third, and fourth rounds yield approximately 100×, 500×, and 1500× linear expansion, respectively. To fully expand the 100×, 500×, and 1000–1500× gels, place an approximately 1 cm gel piece in a large Petri dish (140 mm diameter; Thermo Fisher, 08-757-100). Add ~100 mL deionized water, or enough water to completely fill the dish. Incubate at room temperature (~20 °C) for 24–48 h, until the gel is fully expanded. Water exchange is not required.
22. After the 100× expansion round, axially section the gel to generate thinner gel pieces for the next casting round to reach ~500× expansion. After the 500× expansion round, repeat the axial sectioning step to generate thinner gel pieces for the next casting round to reach ~1000–1500× expansion.

bioRxiv preprint doi: https://doi.org/10.64898/2026.05.31.729018; this version posted July 21, 2026. The copyright holder for this preprint (which was not certified by peer review) is the author/funder, who has granted bioRxiv a license to display the preprint in perpetuity. It is made available under a CC-BY 4.0 International license.

23. To prepare a slicing guide, glue three microscope slides together to make a 3 mm-thick spacer. Prepare two spacers and place them on either side of the gel. Place a razor blade on top of the spacers and slide it through the gel to produce an approximately 3 mm-thick axial section. Use these 3 mm-thick gel sections for the subsequent iterative gel-casting round.

Note: In the original gel recipe (single monomer solution incubation), gels are sometimes not completely permeated with polymer throughout, resulting in variable structural soundness. Approximately 1 to 2 gels out of a 6-well batch per plate come out suboptimal. Use the improved protocol (double incubation) for maximum reproducibility. The composition of the monomer solution is unchanged between protocols

bioRxiv preprint doi: https://doi.org/10.64898/2026.05.31.729018; this version posted July 21, 2026. The copyright holder for this preprint (which was not certified by peer review) is the author/funder, who has granted bioRxiv a license to display the preprint in perpetuity. It is made available under a CC-BY 4.0 International license.

Supplementary Note 3: GFP 3D Reconstruction

Raw fluorescence images acquired from One-step Nanometer-scale Expansion (ONE) microscopy [5] are processed to isolate individual instances before reconstruction. For segmentation, we develop an unsupervised framework based on graph neural networks. As shown in **Supplementary Figure 24**, each image is divided into transformer derived [59] patches, that serve as local descriptors of the underlying signal. As shown in **Supplementary Figure 25**, the resulting feature vectors are embedded into a weighted graph whose edges encode patch similarity. Cluster assignments are obtained by minimizing a correlation clustering objective, $L_{CC}(S)=-\sum_{i,j}W_{ij}\sum_{c}S_{ic}S_{jc}$, where $S_{ic} \in 0,1$ denotes the assignment of node i to cluster c. As shown in **Supplementary Figure 26**, foreground and background separation is achieved in a two-stage process, ensuring robust isolation of protein complexes. Each segmented particle is extracted as a fixed-size sub-image $I_{0.}$ Based on connected component analysis and physical constraint-based filtering, we are performing partitions around mediods [60] to segregate the similar identity objects from the foreground mixed population. As shown in **Supplementary Figure 27**, this formulation encourages patches with similar appearance to co-cluster, yielding segmentation masks that isolate individual protein complexes for downstream analysis.

To enhance the structural signal of each isolated complex, we apply a physics-informed deconvolution [61] procedure in polar coordinates. Given an observed blurred image $I_0(r,\theta)$, the estimate $I_k(r,\theta)$ at iteration k, is iteratively refined according to $I_{k+1}(r,\theta)=I_k(r,\theta).I_0(r,\theta)/\{(I_k*R)(r,\theta)\}*R^{flip}(r,\theta)]$, where $R^{flip}(r,\theta)$ is the flipped PSF kernel, * is the convolution in polar coordinates. This formulation improves recovery of fine structural details compared to Gaussian PSF-based Lucy-Richardson deconvolution.

After normalization, the resized image is then processed by an encoder network $\Psi_\theta(X)$ as shown in **Supplementary Figure 28**, which extracts a compact latent representation of the underlying molecular state. This encoder maps the normalized image to a low-dimensional latent variable: $\Psi_\theta(X)=(R,t,s,z)$, where $R \in SO(3)$ is a rotation matrix defining particle orientation, $t \in R^2$ is a translation vector, $s > 0$ is a scaling factor accounting for gel expansion variability and $z \in R^d$ encodes molecular conformation.

As shown in **Supplementary Figure 28**, the decoder V_ϵ acts as a continuous implicit representation of the 3D density in Fourier space, mapping frequency coordinates and conformation codes to Fourier coefficients: $V_\epsilon(k,z)\rightarrow X'(k;z)$, $X'(k;z)\in R$, where $k=(k_x,k_y,k_z)^T$ is a 3D frequency coordinate and z is the latent conformation vector. Thus, V_ϵ defines the Fourier space representation of the protein complex.

To generate a 2D projection, the decoder is evaluated slice-by-slice on a grid of frequency coordinates corresponding to the central slice through the 3D Fourier volume, rotated according to the estimated pose: $k'_{grid}=s^{-1}\cdot Rk_{grid}$, $P'_c=V_\epsilon(k'_{grid},z)$, $P'(u)=P'_c(u)\cdot e^{-2\pi i(uxtx+uyty)}$. Here, k_{grid} are the 2D frequency coordinates in the projection plane, k'_{grid} are these coordinates rotated into the volume's frame, P'_c is the centered Fourier slice decoded from the density field, and $P'(u)$ is the final Fourier slice after applying the translation phase shift.

Model optimization is performed by comparing this predicted Fourier slice to the Fourier transform of the target image. The target image is transformed to the Fourier domain, $Y'=F[X]$. The symmetric mean squared error loss is minimized through the equation: $L\ (P', Y') = \min\ (\|P'-Y'\|^2_2, \|P'-F[flip(X)]\|^2_2)$. The overall training objective is $\min_{\theta,\epsilon}E_{X\sim data}[L(P'(X;\Psi_\theta,V_\epsilon);Y')]$, where θ are the encoder parameters and ϵ are the decoder parameters.

Following training, the generator is evaluated slice-by-slice across a full grid of frequency coordinates to build a complete 3D Fourier volume. An inverse Fourier transform then produces a volumetric density map that represents a physically plausible 3D molecular structure obtained without imposing predefined atomic

bioRxiv preprint doi: https://doi.org/10.64898/2026.05.31.729018; this version posted July 21, 2026. The copyright holder for this preprint (which was not certified by peer review) is the author/funder, who has granted bioRxiv a license to display the preprint in perpetuity. It is made available under a CC-BY 4.0 International license.

coordinates or using external templates or supervision. The resulting volumetric data is saved in MRC format, and final reconstructions were visualized and analyzed using UCSF ChimeraX [62].

bioRxiv preprint doi: https://doi.org/10.64898/2026.05.31.729018; this version posted July 21, 2026. The copyright holder for this preprint (which was not certified by peer review) is the author/funder, who has granted bioRxiv a license to display the preprint in perpetuity. It is made available under a CC-BY 4.0 International license.

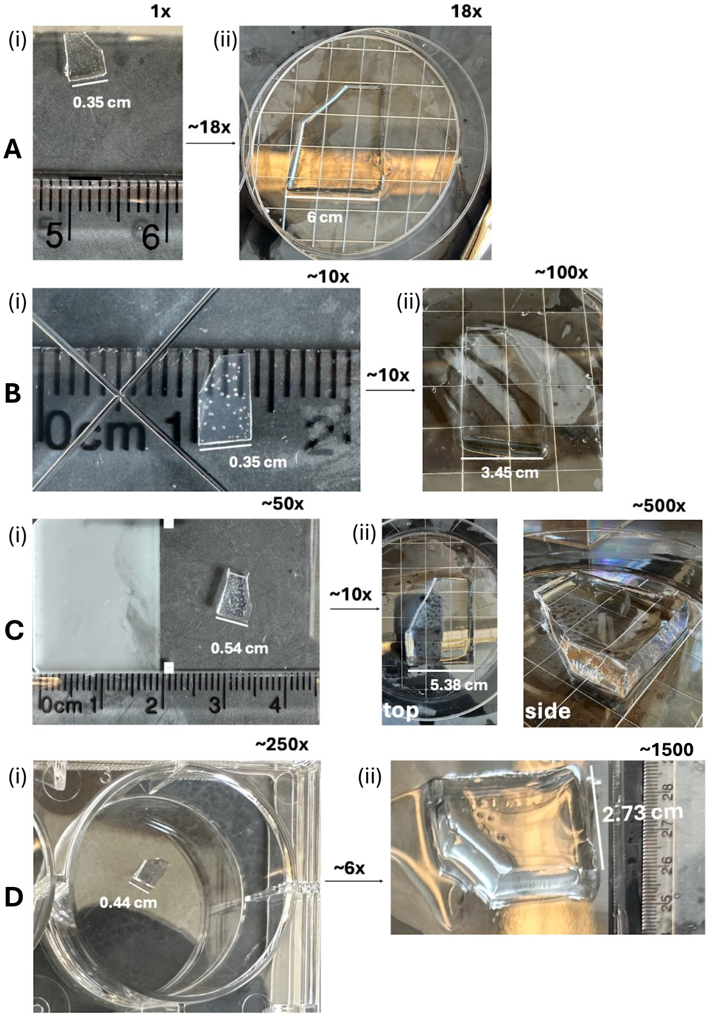

Supplementary Figure 1. Representative hydrogels at successive expansion stages.

bioRxiv preprint doi: https://doi.org/10.64898/2026.05.31.729018; this version posted July 21, 2026. The copyright holder for this preprint (which was not certified by peer review) is the author/funder, who has granted bioRxiv a license to display the preprint in perpetuity. It is made available under a CC-BY 4.0 International license.

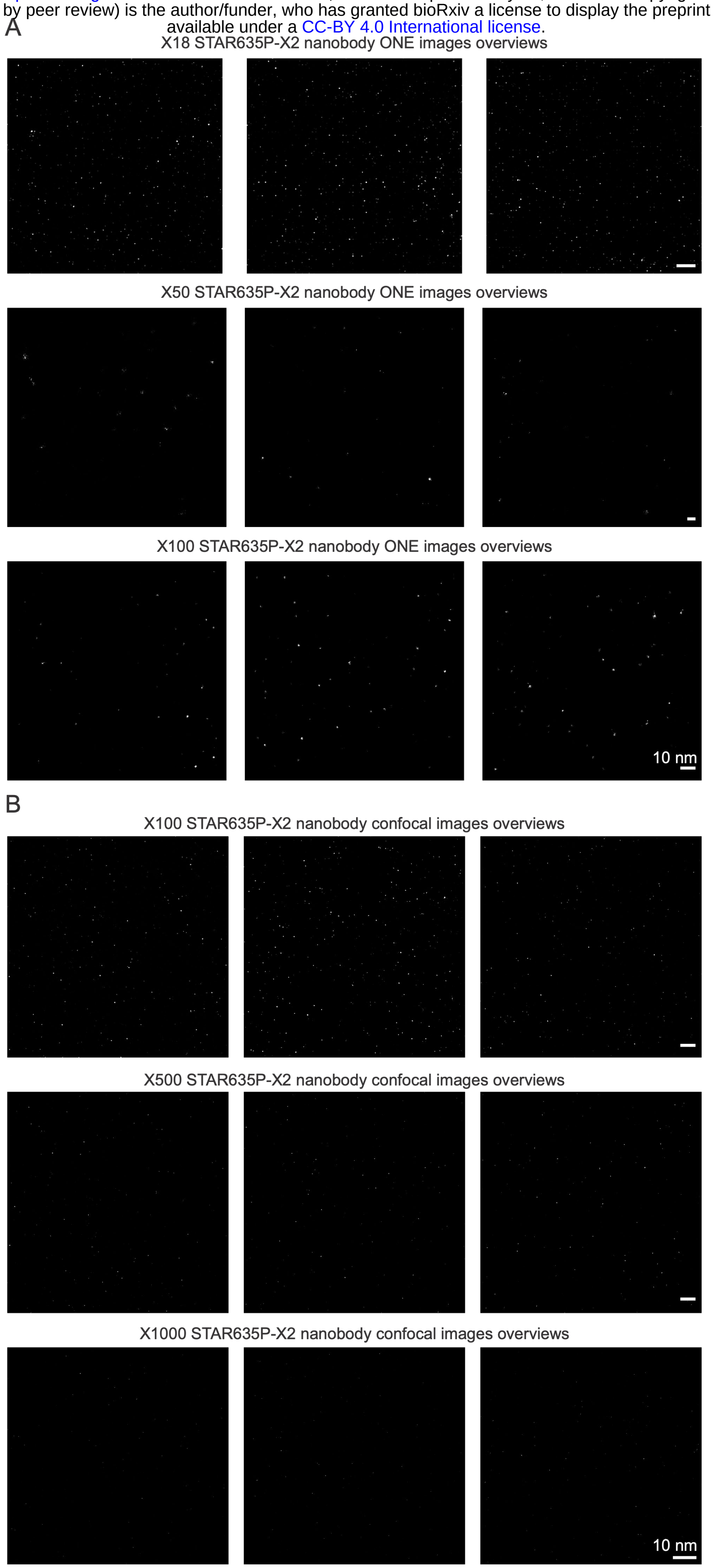

Supplementary Figure 2. Nanobody imaging across different linear expansion factors.

bioRxiv preprint doi: https://doi.org/10.64898/2026.05.31.729018; this version posted July 21, 2026. The copyright holder for this preprint (which was not certified by peer review) is the author/funder, who has granted bioRxiv a license to display the preprint in perpetuity. It is made available under a CC-BY 4.0 International license.

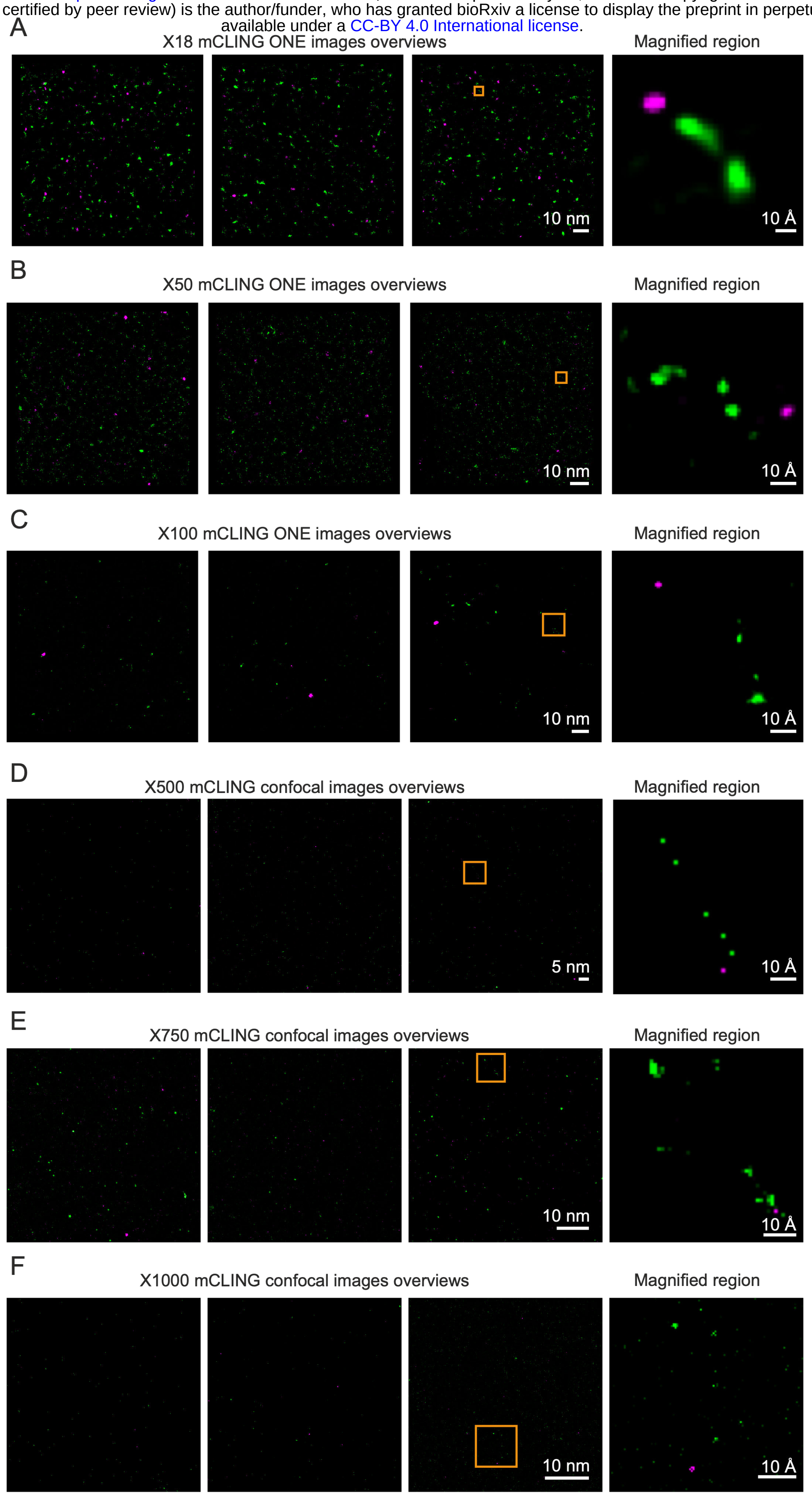

Supplementary Figure 3. mCLING peptide imaging across different linear expansion factors.

bioRxiv preprint doi: https://doi.org/10.64898/2026.05.31.729018; this version posted July 21, 2026. The copyright holder for this preprint (which was not certified by peer review) is the author/funder, who has granted bioRxiv a license to display the preprint in perpetuity. It is made available under a CC-BY 4.0 International license.

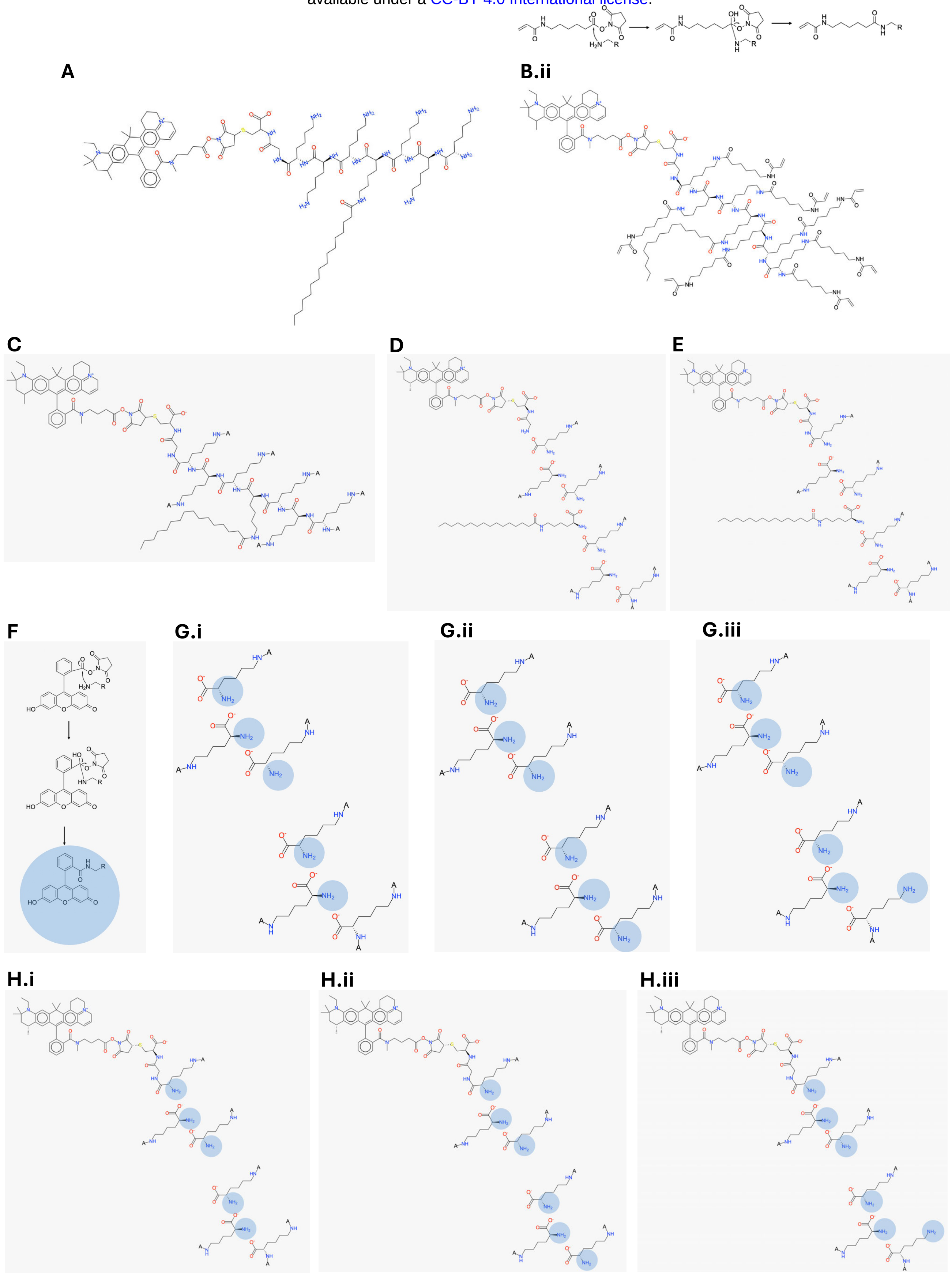

Supplementary Figure 4. Pan-lysine anchoring, proteolytic cleavage, and post-expansion labeling chemistry illustrated using the mCLING peptide.

bioRxiv preprint doi: https://doi.org/10.64898/2026.05.31.729018; this version posted July 21, 2026. The copyright holder for this preprint (which was not certified by peer review) is the author/funder, who has granted bioRxiv a license to display the preprint in perpetuity. It is made available under a CC-BY 4.0 International license.

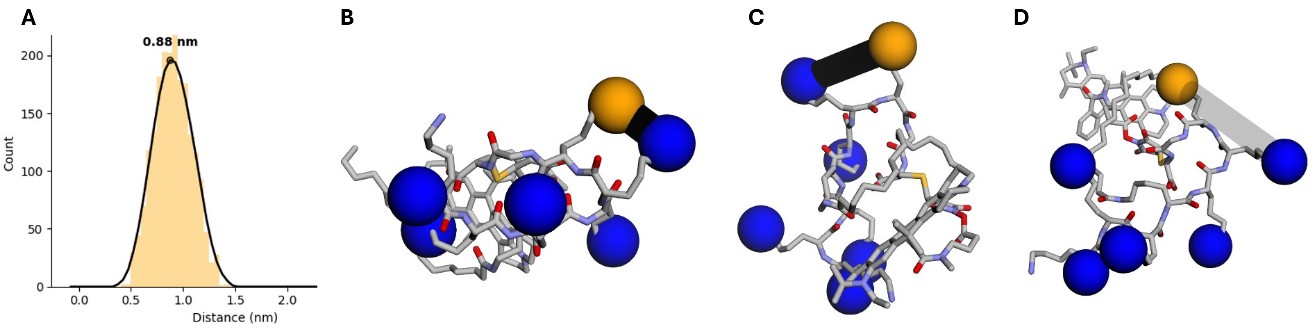

Supplementary Figure 5. Distance distribution between adjacent lysine side chains.

bioRxiv preprint doi: https://doi.org/10.64898/2026.05.31.729018; this version posted July 21, 2026. The copyright holder for this preprint (which was not certified by peer review) is the author/funder, who has granted bioRxiv a license to display the preprint in perpetuity. It is made available under a CC-BY 4.0 International license.

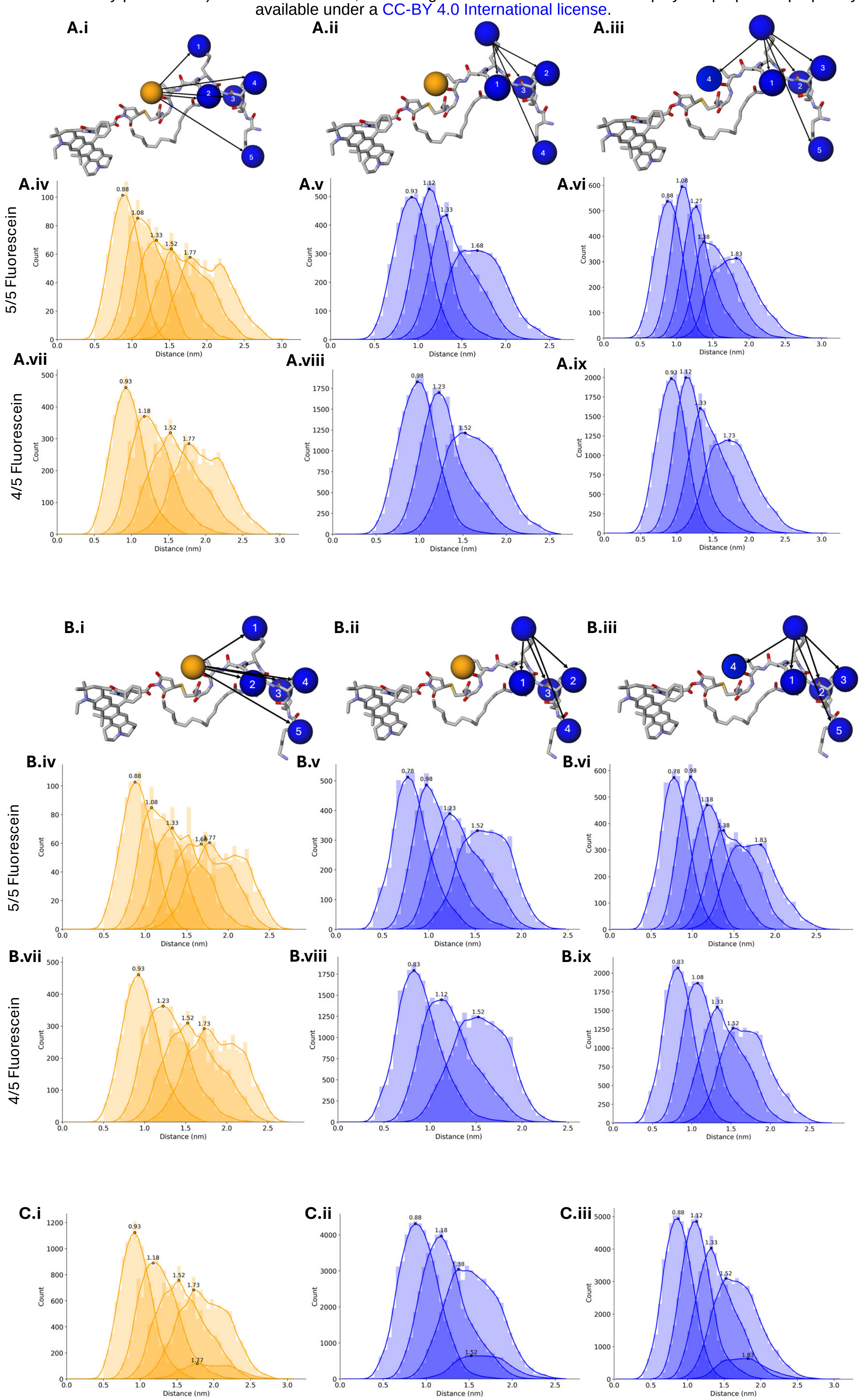

Supplementary Figure 6. Molecular dynamics (MD) simulation of k-nearest-neighbor (kNN) distance distributions for the mCLING peptide under defined anchoring, cleavage, and labeling scenarios.

bioRxiv preprint doi: https://doi.org/10.64898/2026.05.31.729018; this version posted July 21, 2026. The copyright holder for this preprint (which was not certified by peer review) is the author/funder, who has granted bioRxiv a license to display the preprint in perpetuity. It is made available under a CC-BY 4.0 International license.

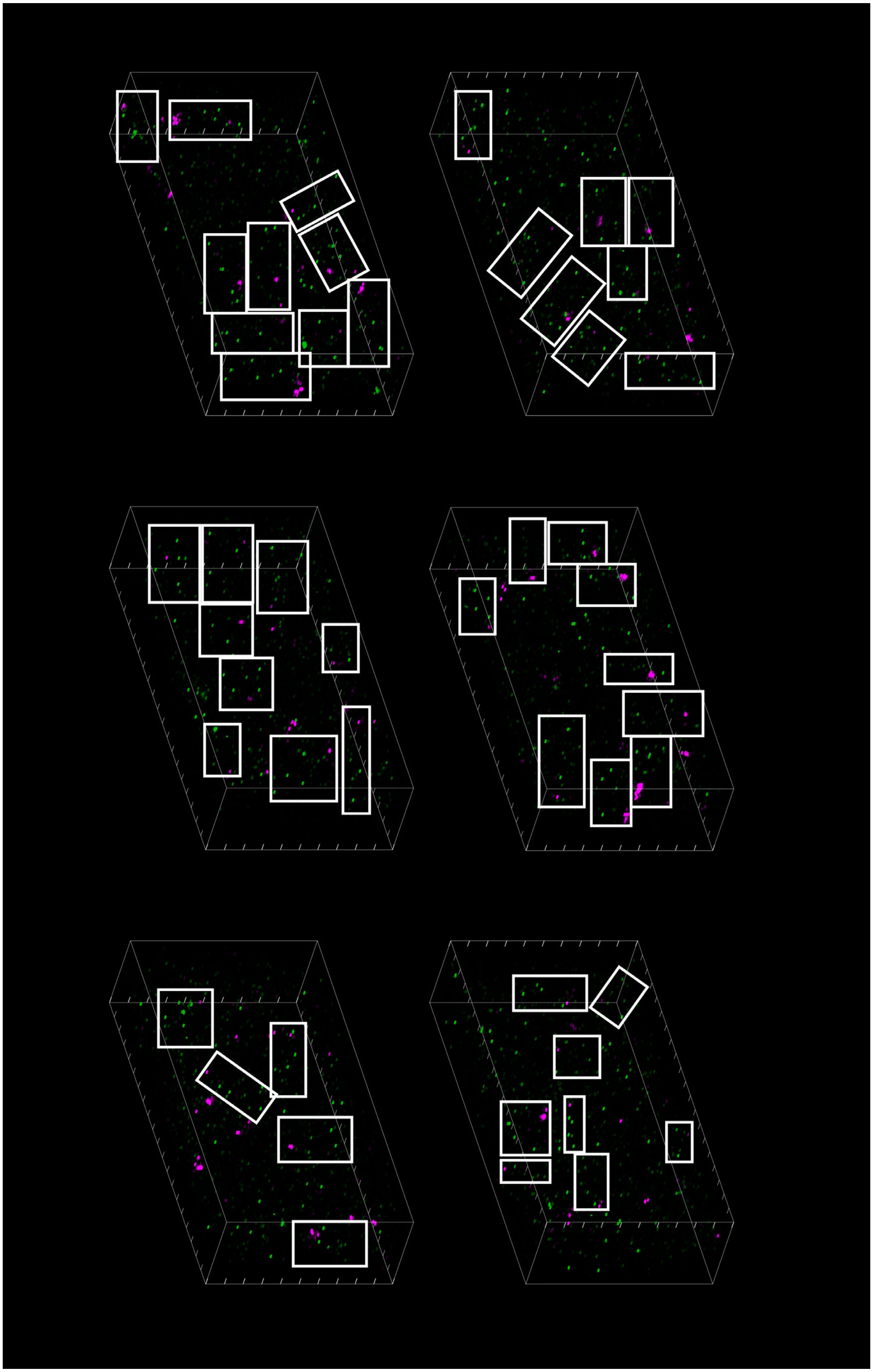

Supplementary Figure 7. 3D regions containing putative mCLING peptides after ~1000× expansion.

bioRxiv preprint doi: https://doi.org/10.64898/2026.05.31.729018; this version posted July 21, 2026. The copyright holder for this preprint (which was not certified by peer review) is the author/funder, who has granted bioRxiv a license to display the preprint in perpetuity. It is made available under a CC-BY 4.0 International license.

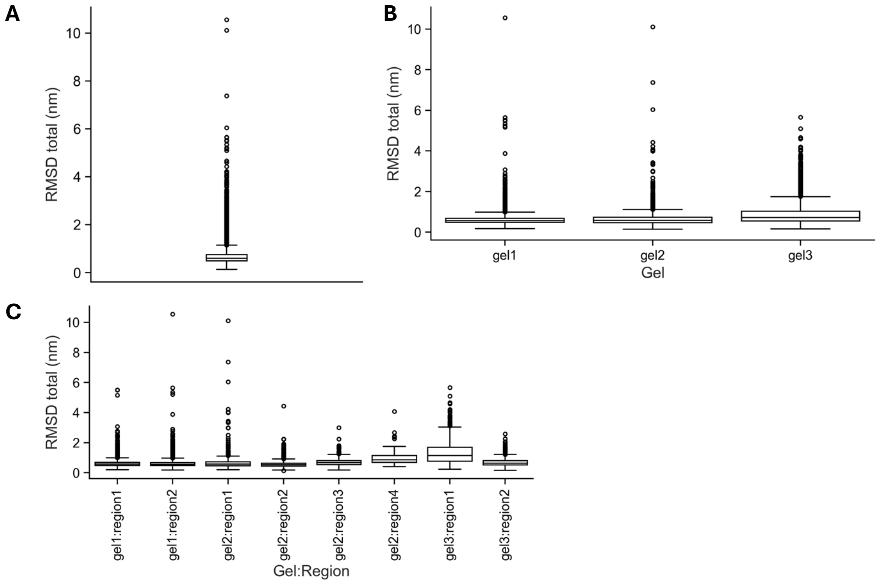

Supplementary Figure 8. RMSD analysis of coarse putative peptides before nearest-neighbor filtering.

bioRxiv preprint doi: https://doi.org/10.64898/2026.05.31.729018; this version posted July 21, 2026. The copyright holder for this preprint (which was not certified by peer review) is the author/funder, who has granted bioRxiv a license to display the preprint in perpetuity. It is made available under a CC-BY 4.0 International license.

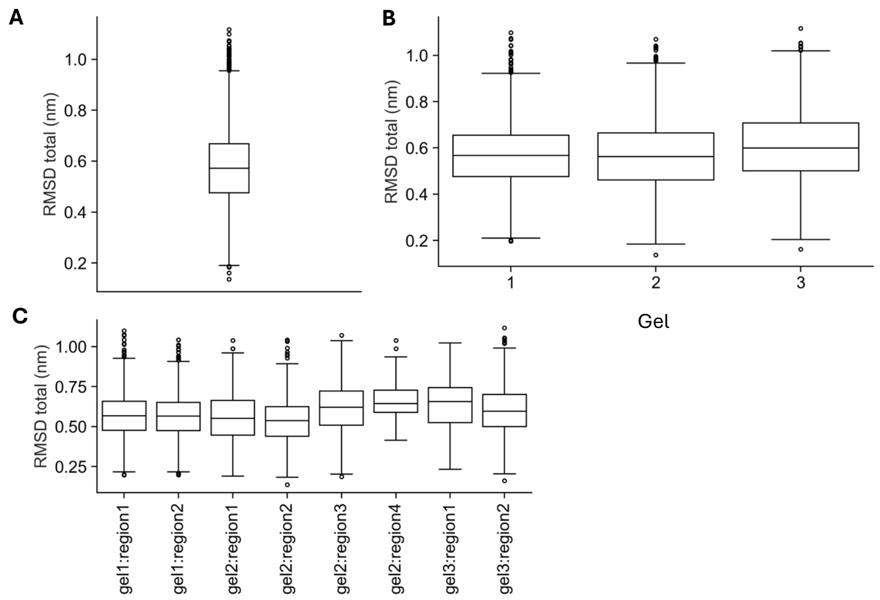

Supplementary Figure 9. RMSD analysis of putative peptides after nearest-neighbor filtering.

bioRxiv preprint doi: https://doi.org/10.64898/2026.05.31.729018; this version posted July 21, 2026. The copyright holder for this preprint (which was not certified by peer review) is the author/funder, who has granted bioRxiv a license to display the preprint in perpetuity. It is made available under a CC-BY 4.0 International license.

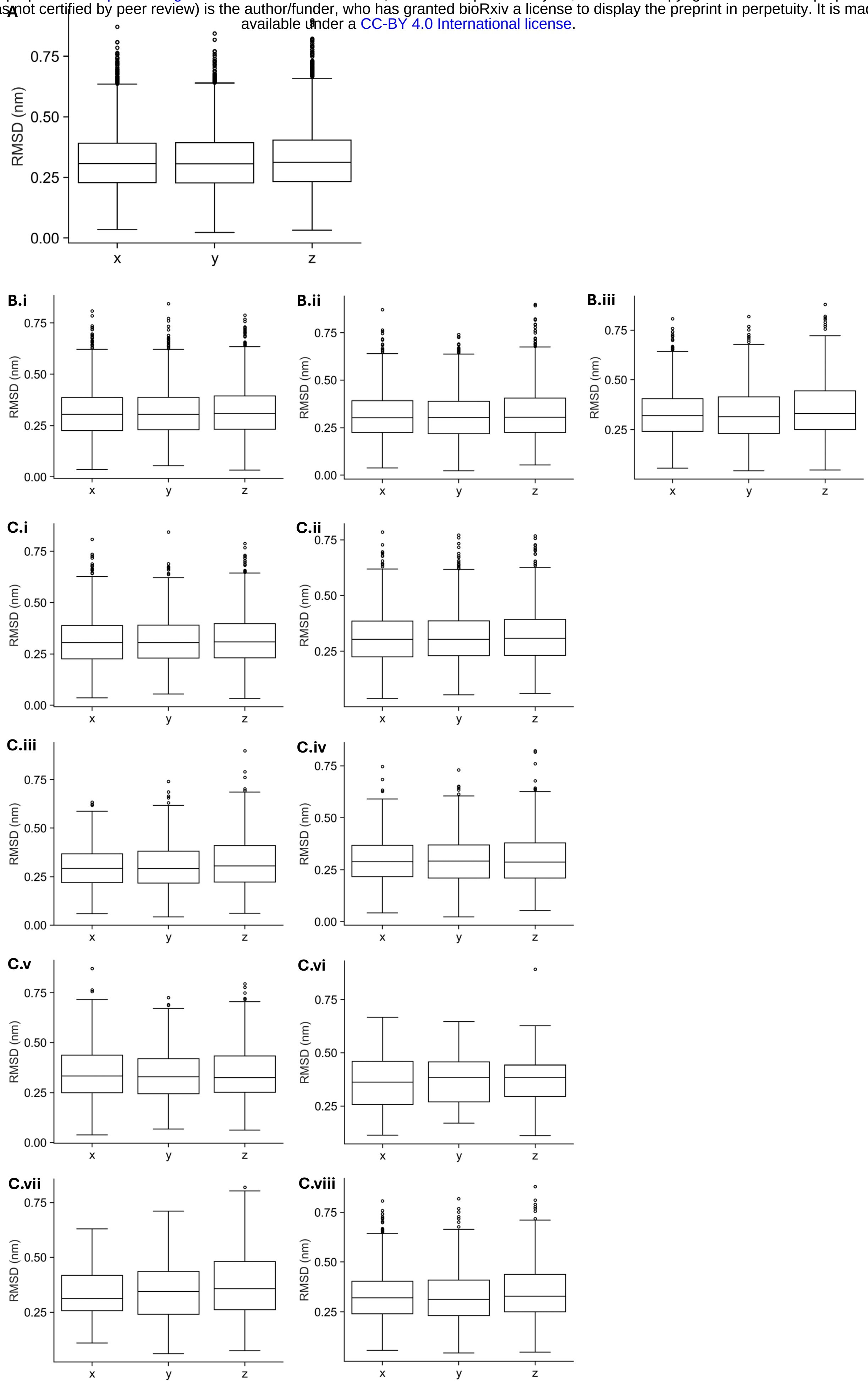

Supplementary Figure 10. Per-axis RMSD of putative peptides (same peptide set as Fig. 3C).

bioRxiv preprint doi: https://doi.org/10.64898/2026.05.31.729018; this version posted July 21, 2026. The copyright holder for this preprint (which was not certified by peer review) is the author/funder, who has granted bioRxiv a license to display the preprint in perpetuity. It is made available under a CC-BY 4.0 International license.

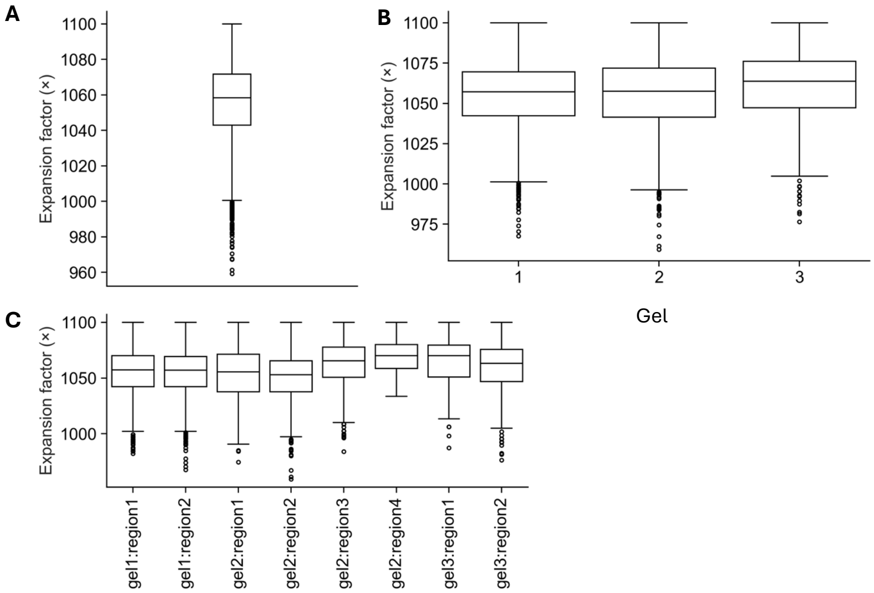

Supplementary Figure 11. Single-molecule expansion factors (same peptide set as Fig. 3C).

bioRxiv preprint doi: https://doi.org/10.64898/2026.05.31.729018; this version posted July 21, 2026. The copyright holder for this preprint (which was not certified by peer review) is the author/funder, who has granted bioRxiv a license to display the preprint in perpetuity. It is made available under a CC-BY 4.0 International license.

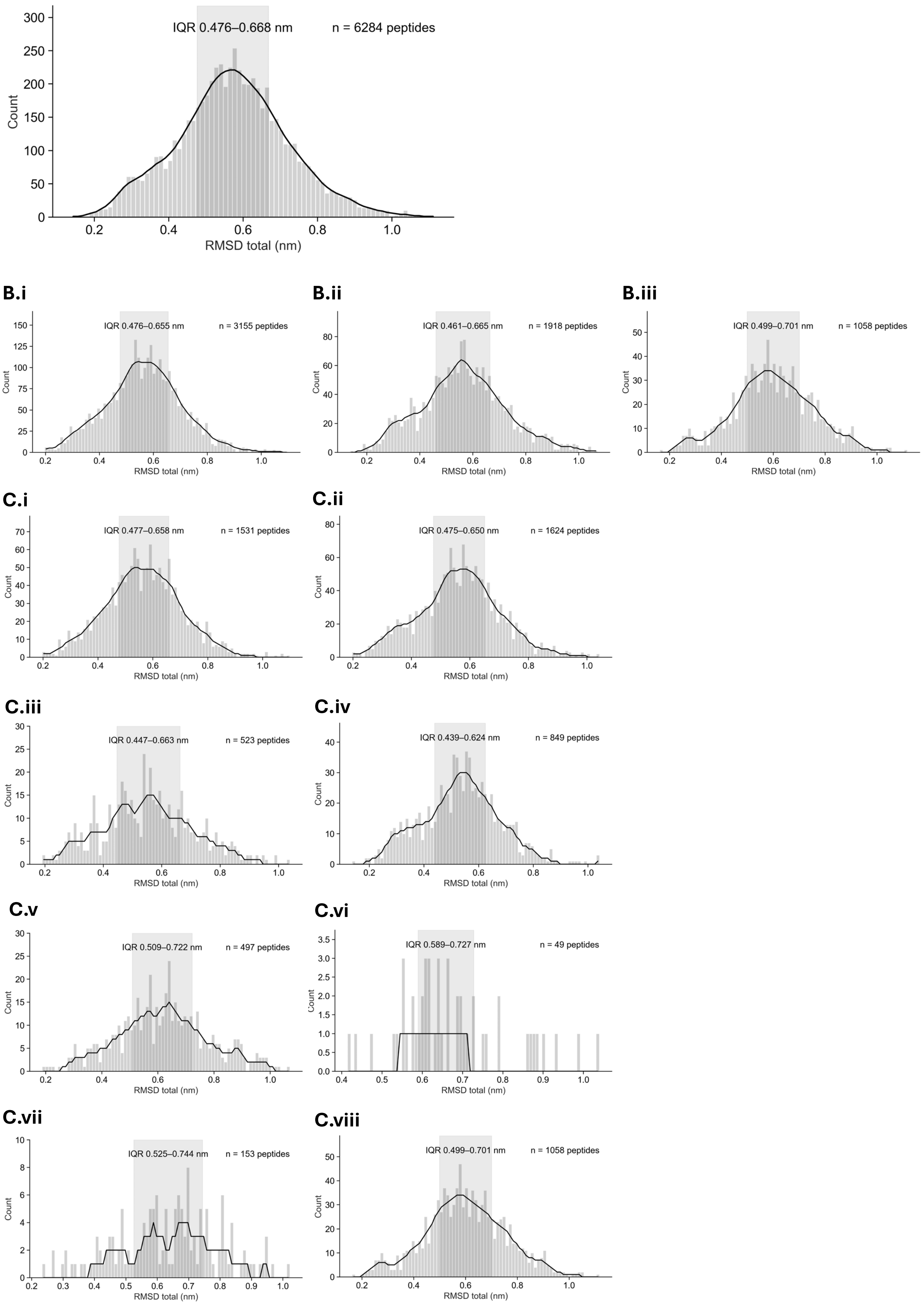

Supplementary Figure 12. RMSD histograms (same peptide set and RMSD values as Fig. 3C).

bioRxiv preprint doi: https://doi.org/10.64898/2026.05.31.729018; this version posted July 21, 2026. The copyright holder for this preprint (which was not certified by peer review) is the author/funder, who has granted bioRxiv a license to display the preprint in perpetuity. It is made available under a CC-BY 4.0 International license.

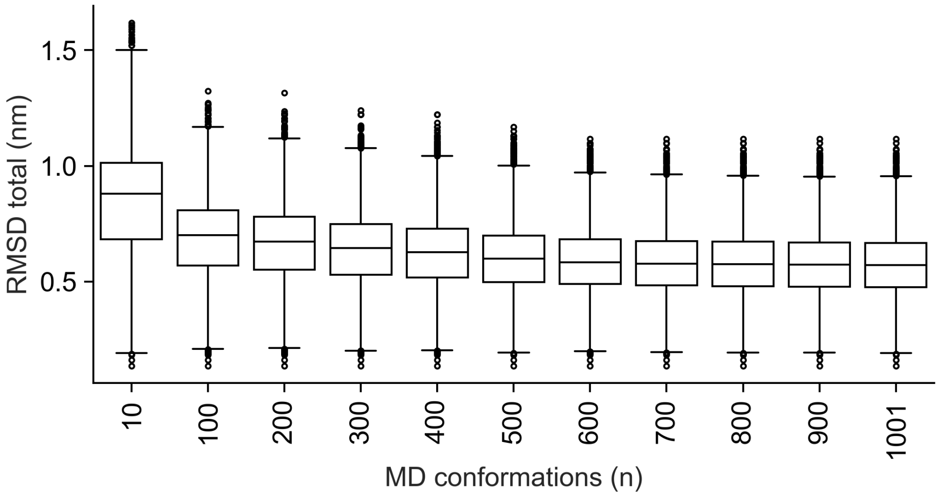

Supplementary Figure 13. Robustness of RMSD to MD ensemble size.

bioRxiv preprint doi: https://doi.org/10.64898/2026.05.31.729018; this version posted July 21, 2026. The copyright holder for this preprint (which was not certified by peer review) is the author/funder, who has granted bioRxiv a license to display the preprint in perpetuity. It is made available under a CC-BY 4.0 International license.

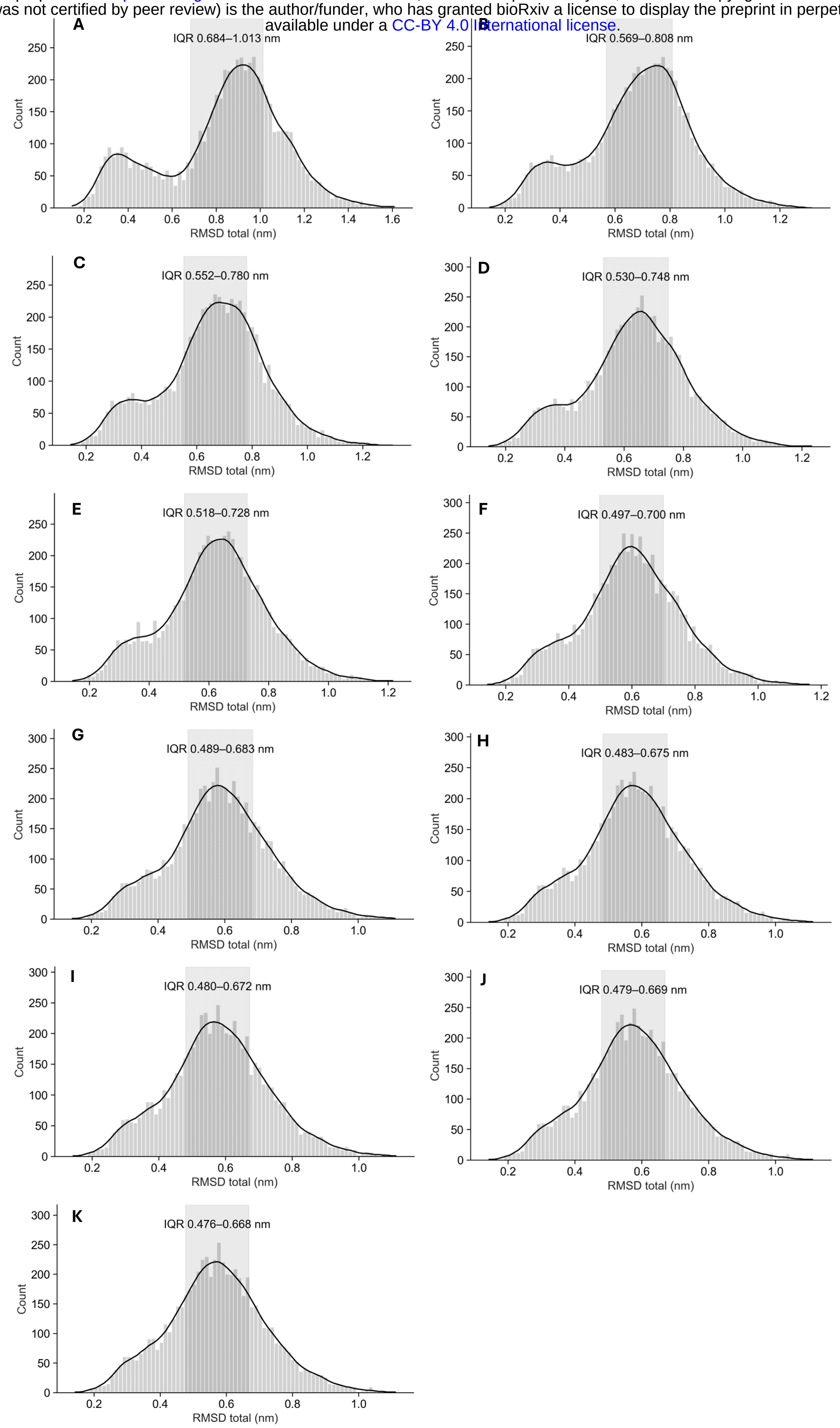

Supplementary Figure 14. RMSD histograms across MD ensemble sizes.

bioRxiv preprint doi: https://doi.org/10.64898/2026.05.31.729018; this version posted July 21, 2026. The copyright holder for this preprint (which was not certified by peer review) is the author/funder, who has granted bioRxiv a license to display the preprint in perpetuity. It is made available under a CC-BY 4.0 International license.

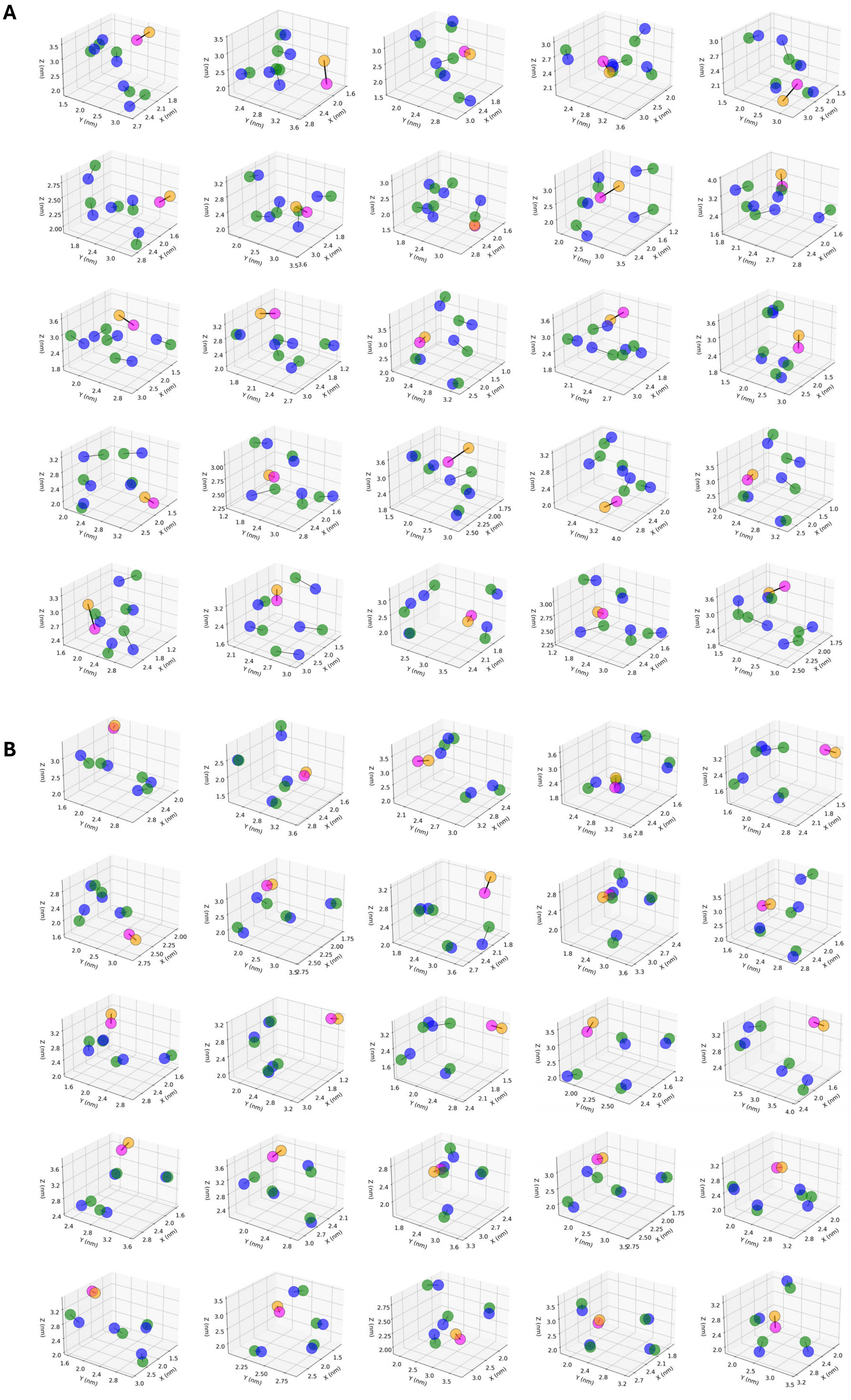

Supplementary Figure 15. Peptide overlays below the IQR of the Fig. 3C.i RMSD distribution.

bioRxiv preprint doi: https://doi.org/10.64898/2026.05.31.729018; this version posted July 21, 2026. The copyright holder for this preprint (which was not certified by peer review) is the author/funder, who has granted bioRxiv a license to display the preprint in perpetuity. It is made available under a CC-BY 4.0 International license.

A

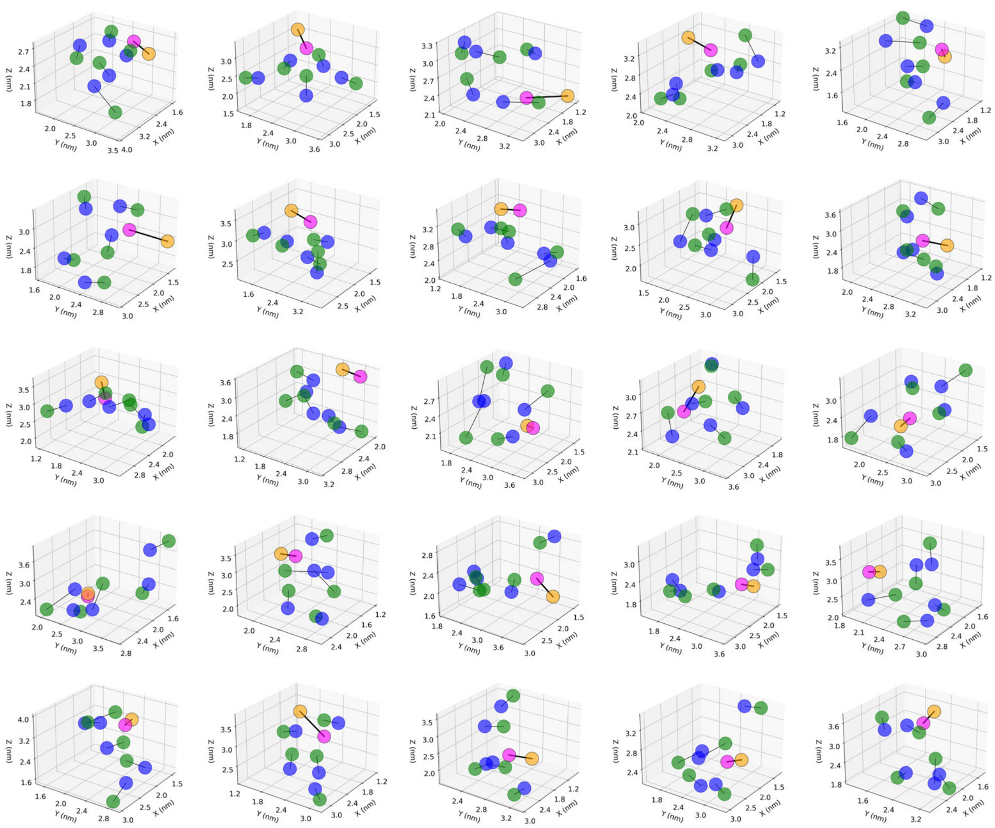

B

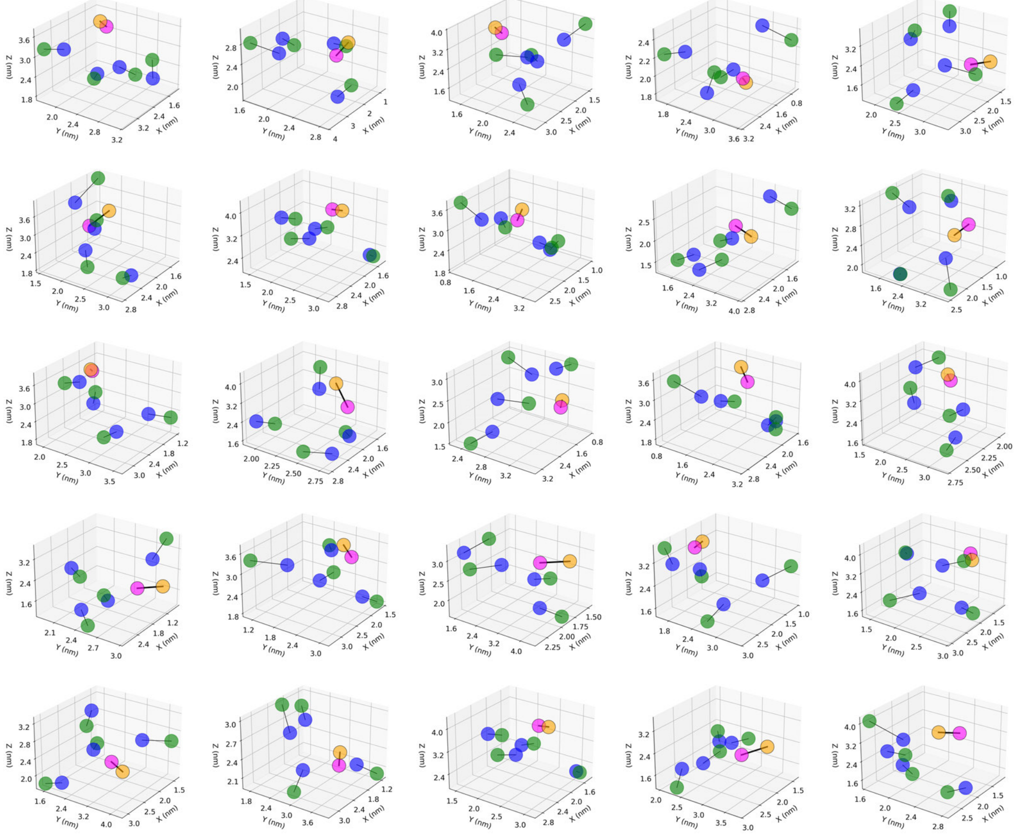

Supplementary Figure 16. Peptide overlays within the IQR of the Fig. 3C.i RMSD distribution.

bioRxiv preprint doi: https://doi.org/10.64898/2026.05.31.729018; this version posted July 21, 2026. The copyright holder for this preprint (which was not certified by peer review) is the author/funder, who has granted bioRxiv a license to display the preprint in perpetuity. It is made available under a CC-BY 4.0 International license.

A

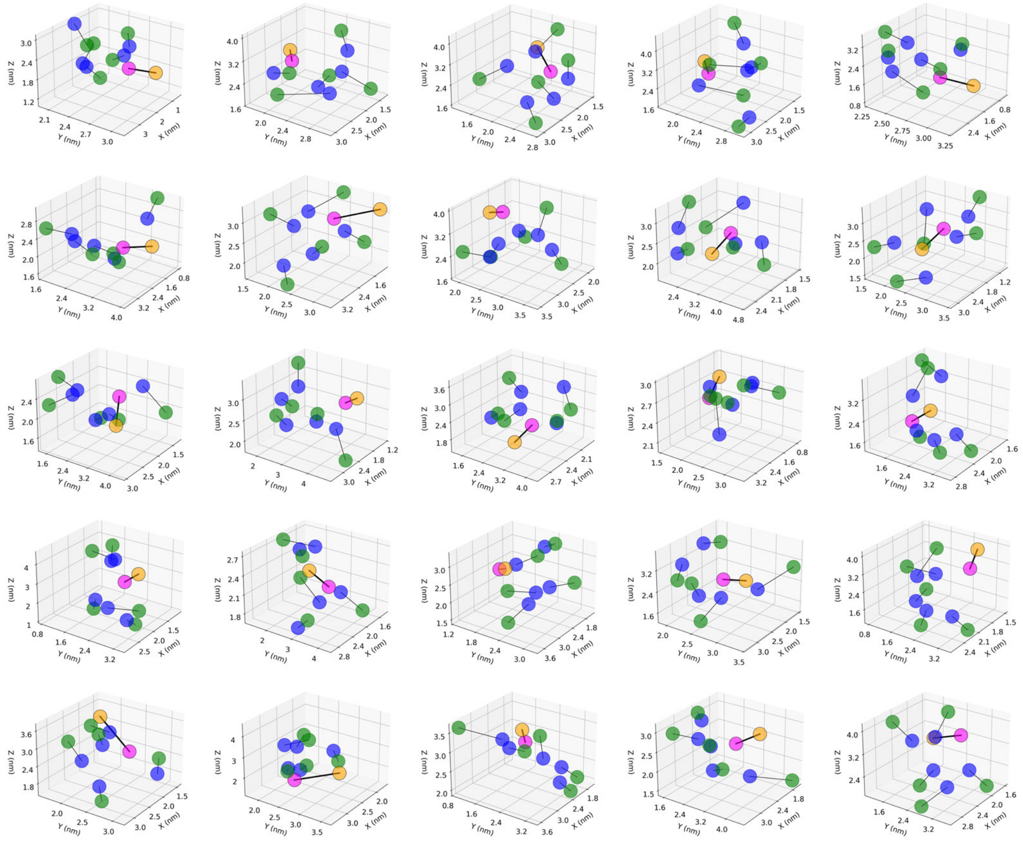

B

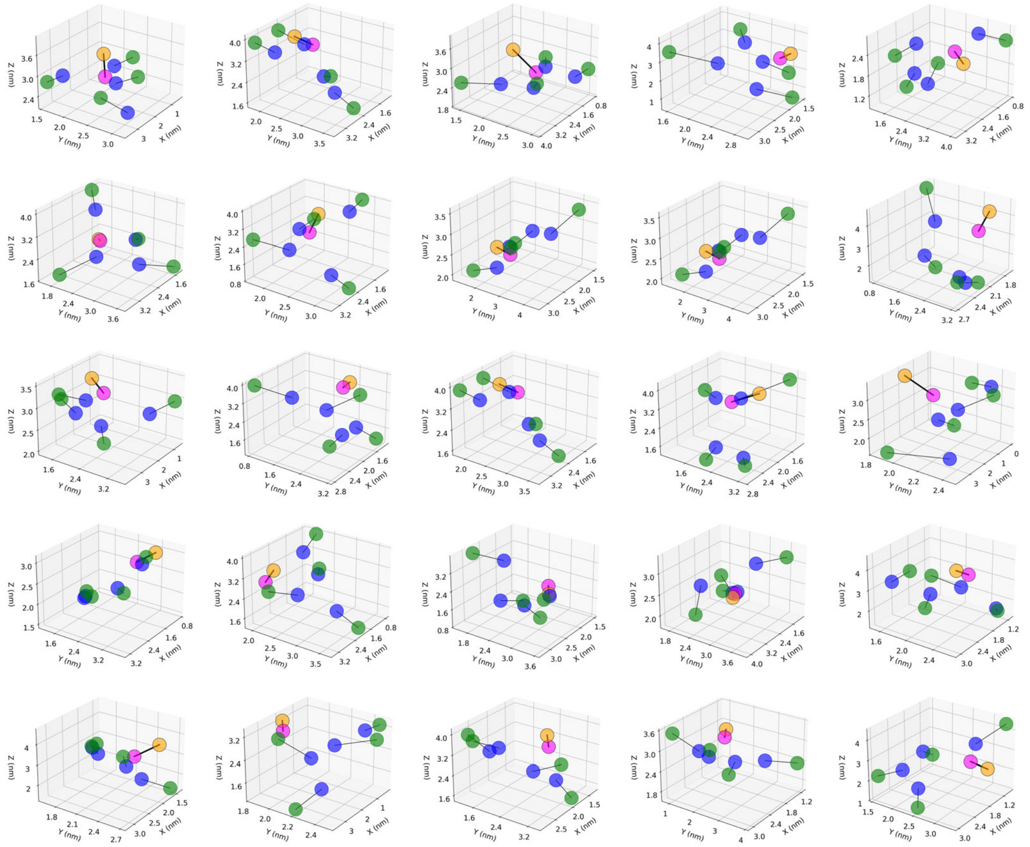

Supplementary Figure 17. Peptide overlays above the IQR of the Fig. 3C.i RMSD distribution.

bioRxiv preprint doi: https://doi.org/10.64898/2026.05.31.729018; this version posted July 21, 2026. The copyright holder for this preprint (which was not certified by peer review) is the author/funder, who has granted bioRxiv a license to display the preprint in perpetuity. It is made available under a CC-BY 4.0 International license.

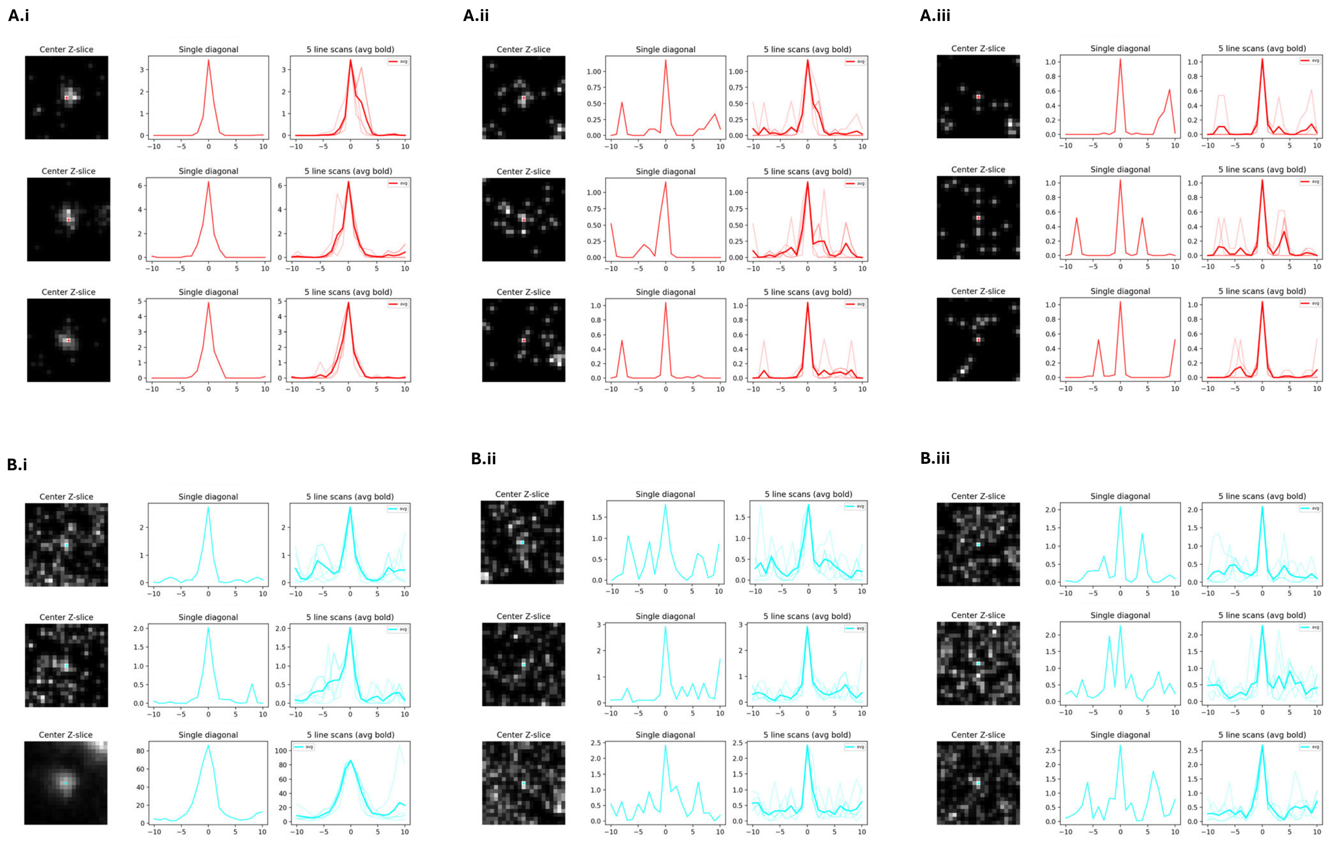

Supplementary Figure 18. Emitter detection using Gaussian correlation to identify true fluorescent emitters.

bioRxiv preprint doi: https://doi.org/10.64898/2026.05.31.729018; this version posted July 21, 2026. The copyright holder for this preprint (which was not certified by peer review) is the author/funder, who has granted bioRxiv a license to display the preprint in perpetuity. It is made available under a CC-BY 4.0 International license.

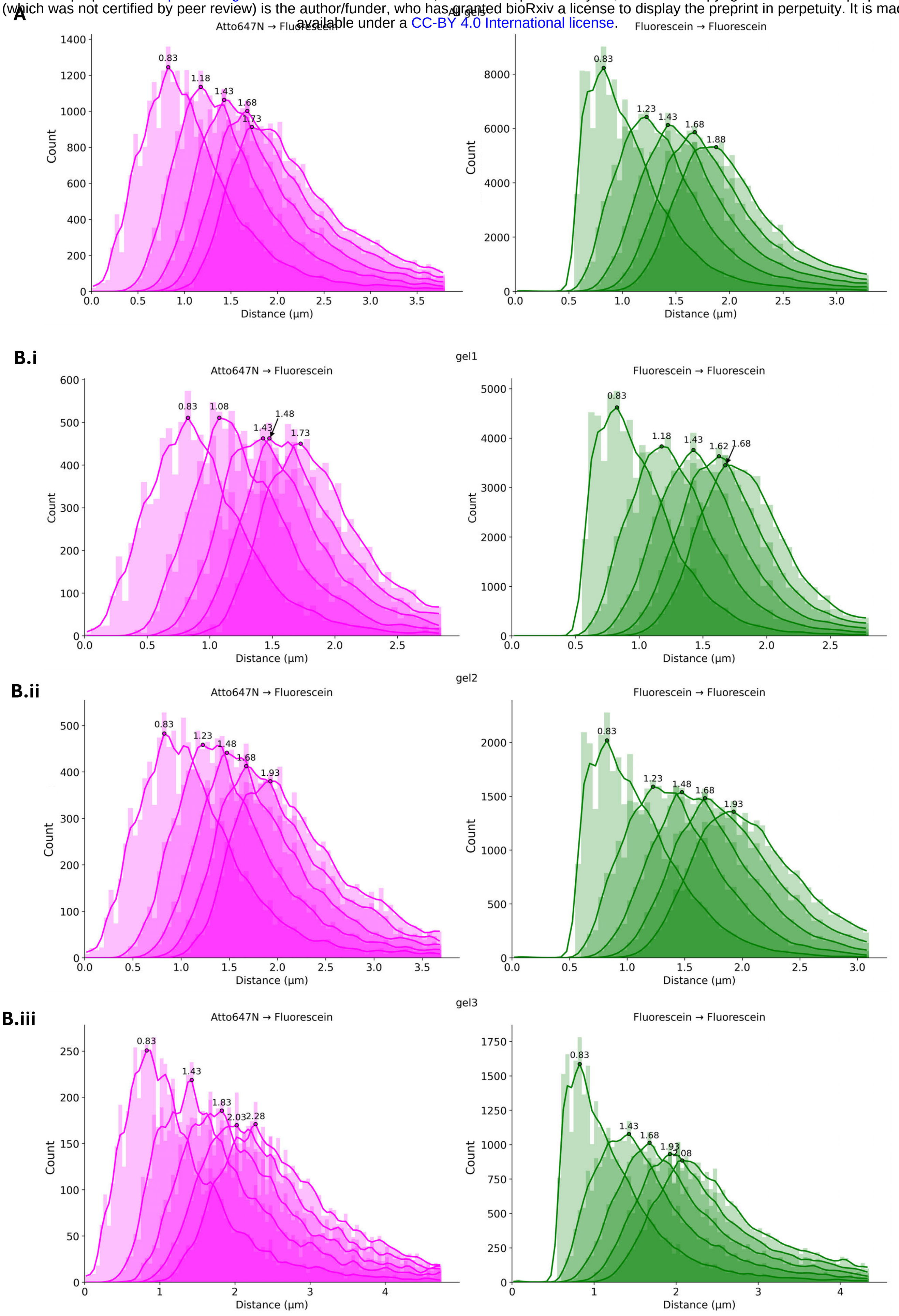

Supplementary Figure 19. k-nearest-neighbor (kNN) 1–5 distance distributions for Atto647N–fluorescein (magenta) and fluorescein–fluorescein (green).

bioRxiv preprint doi: https://doi.org/10.64898/2026.05.31.729018; this version posted July 21, 2026. The copyright holder for this preprint (which was not certified by peer review) is the author/funder, who has granted bioRxiv a license to display the preprint in perpetuity. It is made available under a CC-BY 4.0 International license.

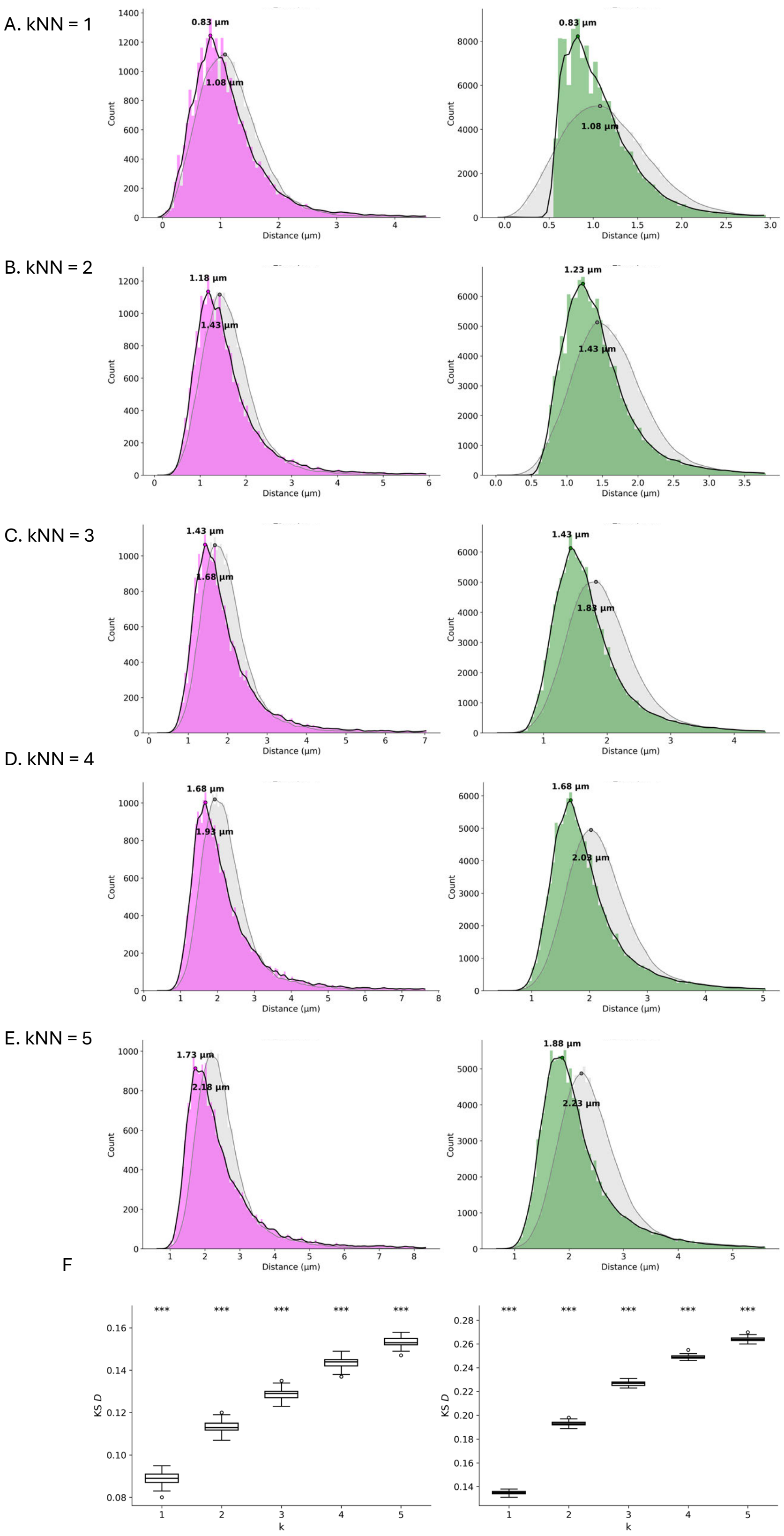

Supplementary Figure 20. Randomization analysis of k-nearest-neighbor (kNN) distance distributions across all gels.

bioRxiv preprint doi: https://doi.org/10.64898/2026.05.31.729018; this version posted July 21, 2026. The copyright holder for this preprint (which was not certified by peer review) is the author/funder, who has granted bioRxiv a license to display the preprint in perpetuity. It is made available under a CC-BY 4.0 International license.

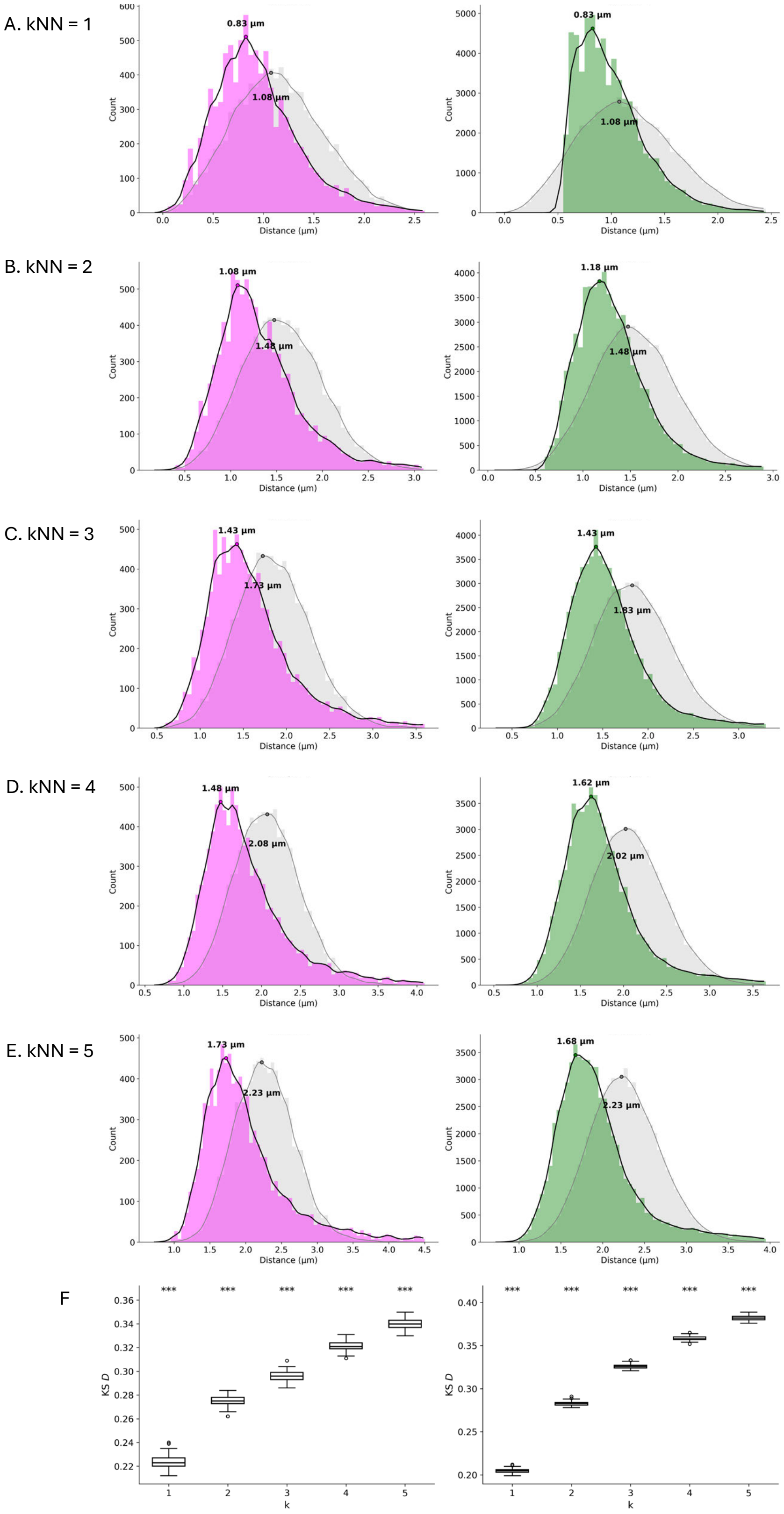

Supplementary Figure 21. Randomization analysis of k-nearest-neighbor (kNN) distance distributions for gel 1.

bioRxiv preprint doi: https://doi.org/10.64898/2026.05.31.729018; this version posted July 21, 2026. The copyright holder for this preprint (which was not certified by peer review) is the author/funder, who has granted bioRxiv a license to display the preprint in perpetuity. It is made available under a CC-BY 4.0 International license.

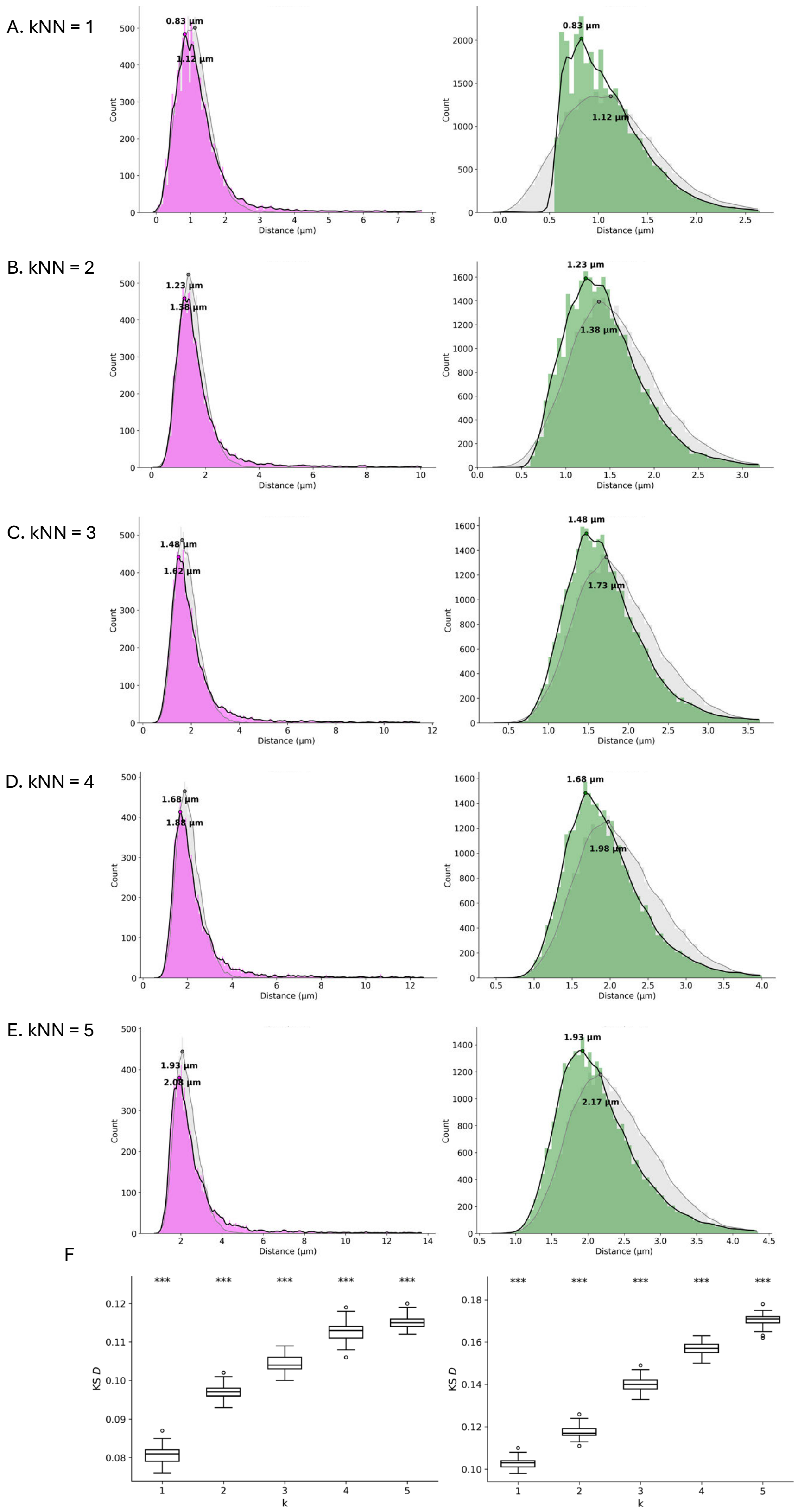

Supplementary Figure 22. Randomization analysis of k-nearest-neighbor (kNN) distance distributions for gel 2.

bioRxiv preprint doi: https://doi.org/10.64898/2026.05.31.729018; this version posted July 21, 2026. The copyright holder for this preprint (which was not certified by peer review) is the author/funder, who has granted bioRxiv a license to display the preprint in perpetuity. It is made available under a CC-BY 4.0 International license.

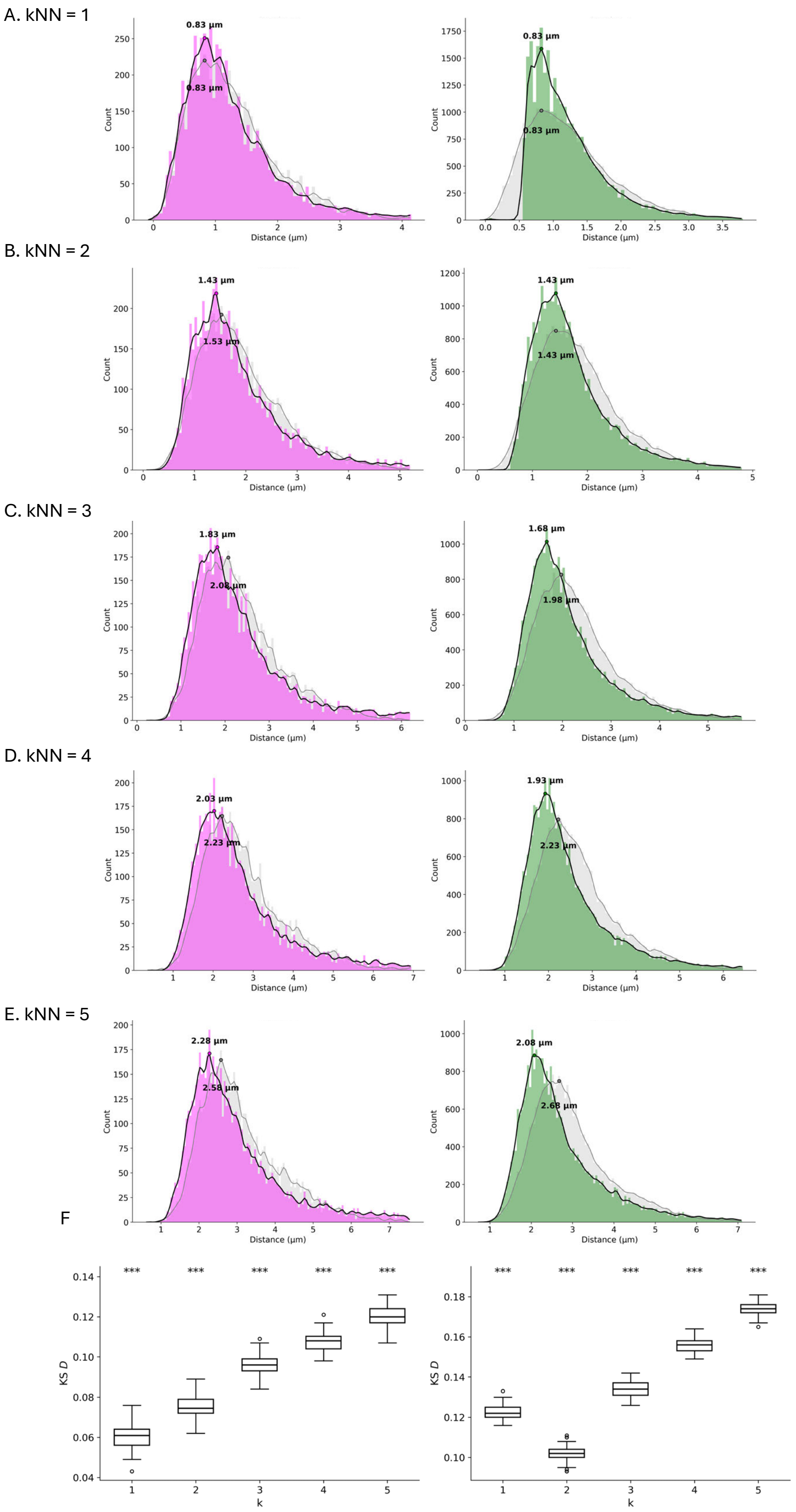

Supplementary Figure 23. Randomization analysis of k-nearest-neighbor (kNN) distance distributions for gel 3.

bioRxiv preprint doi: https://doi.org/10.64898/2026.05.31.729018; this version posted July 21, 2026. The copyright holder for this preprint (which was not certified by peer review) is the author/funder, who has granted bioRxiv a license to display the preprint in perpetuity. It is made available under a CC-BY 4.0 International license.

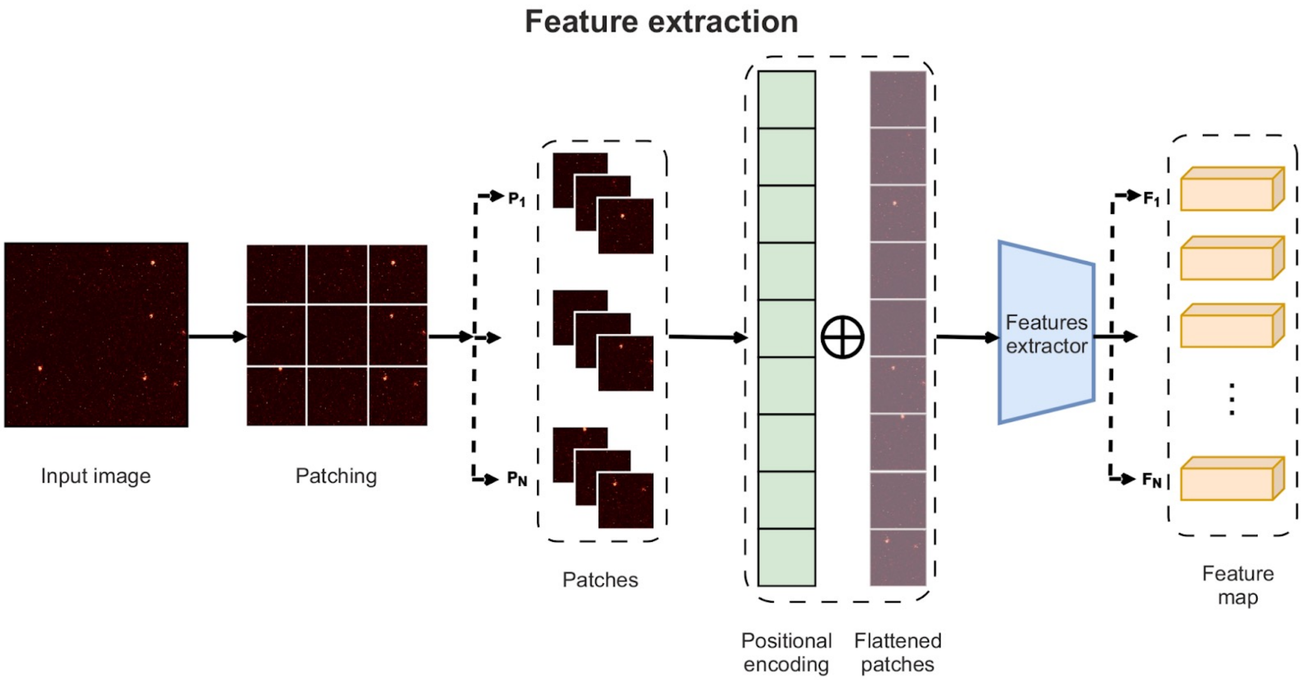

Supplementary Figure 24. Preprocessing and transformer-based feature extraction pipeline for microscopy images.

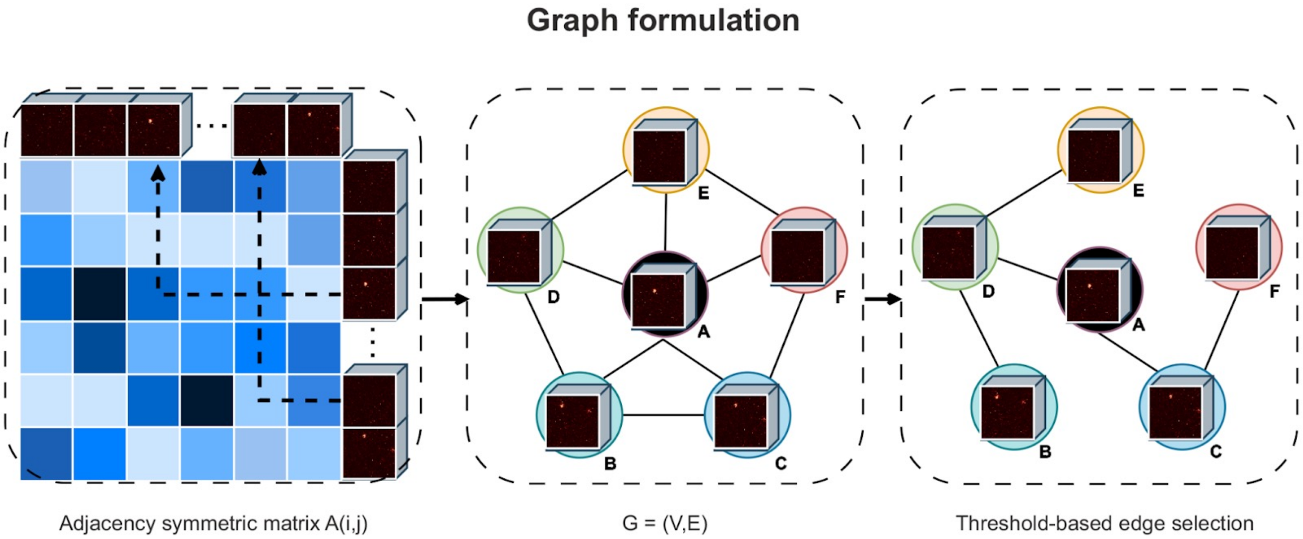

Supplementary Figure 25. Graph formulation from transformer-derived patch features.

bioRxiv preprint doi: https://doi.org/10.64898/2026.05.31.729018; this version posted July 21, 2026. The copyright holder for this preprint (which was not certified by peer review) is the author/funder, who has granted bioRxiv a license to display the preprint in perpetuity. It is made available under a CC-BY 4.0 International license.

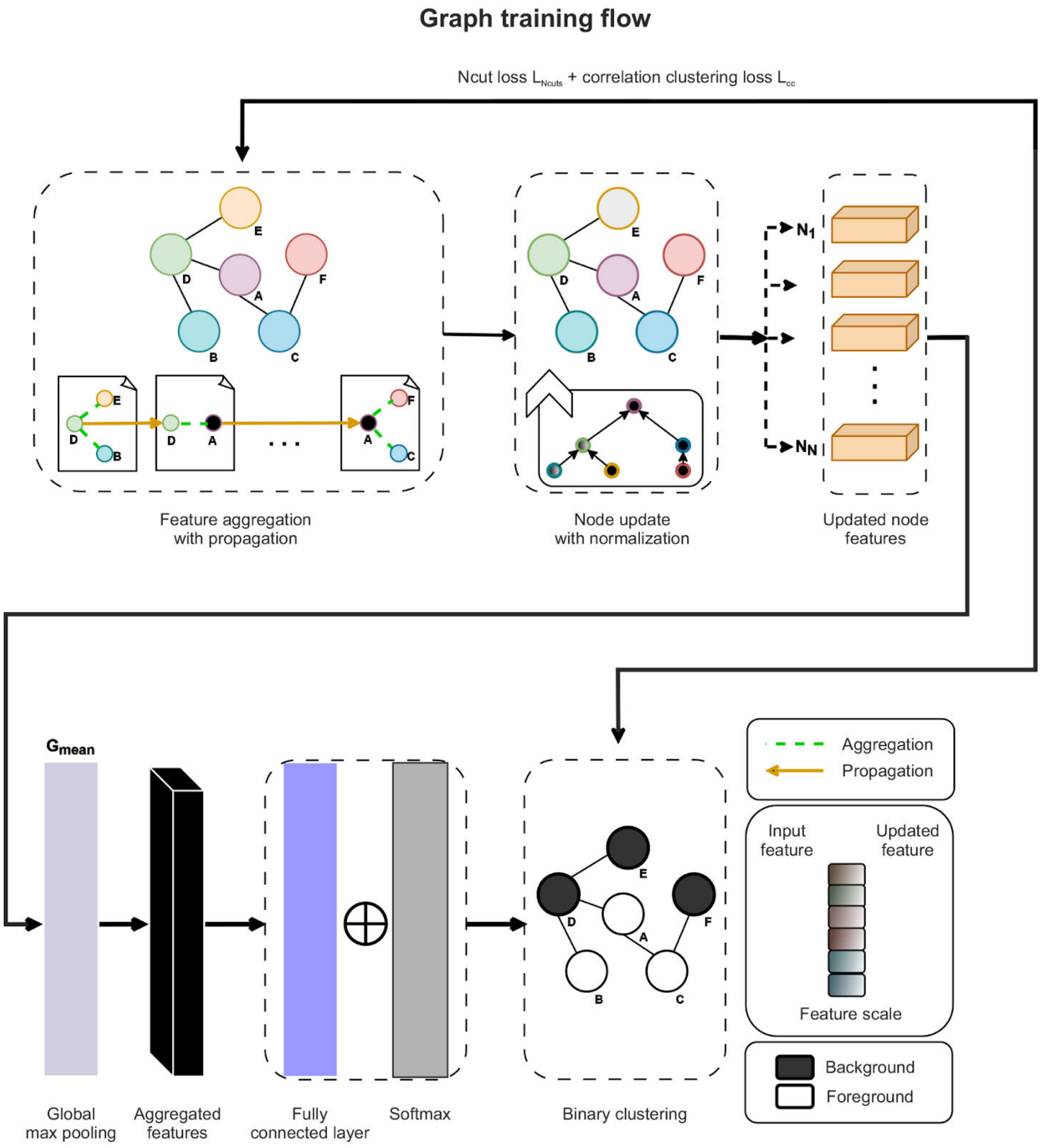

Supplementary Figure 26. **Unsupervised graph-based segmentation framework integrating Normalized Cut and correlation clustering losses.**

bioRxiv preprint doi: https://doi.org/10.64898/2026.05.31.729018; this version posted July 21, 2026. The copyright holder for this preprint (which was not certified by peer review) is the author/funder, who has granted bioRxiv a license to display the preprint in perpetuity. It is made available under a CC-BY 4.0 International license.

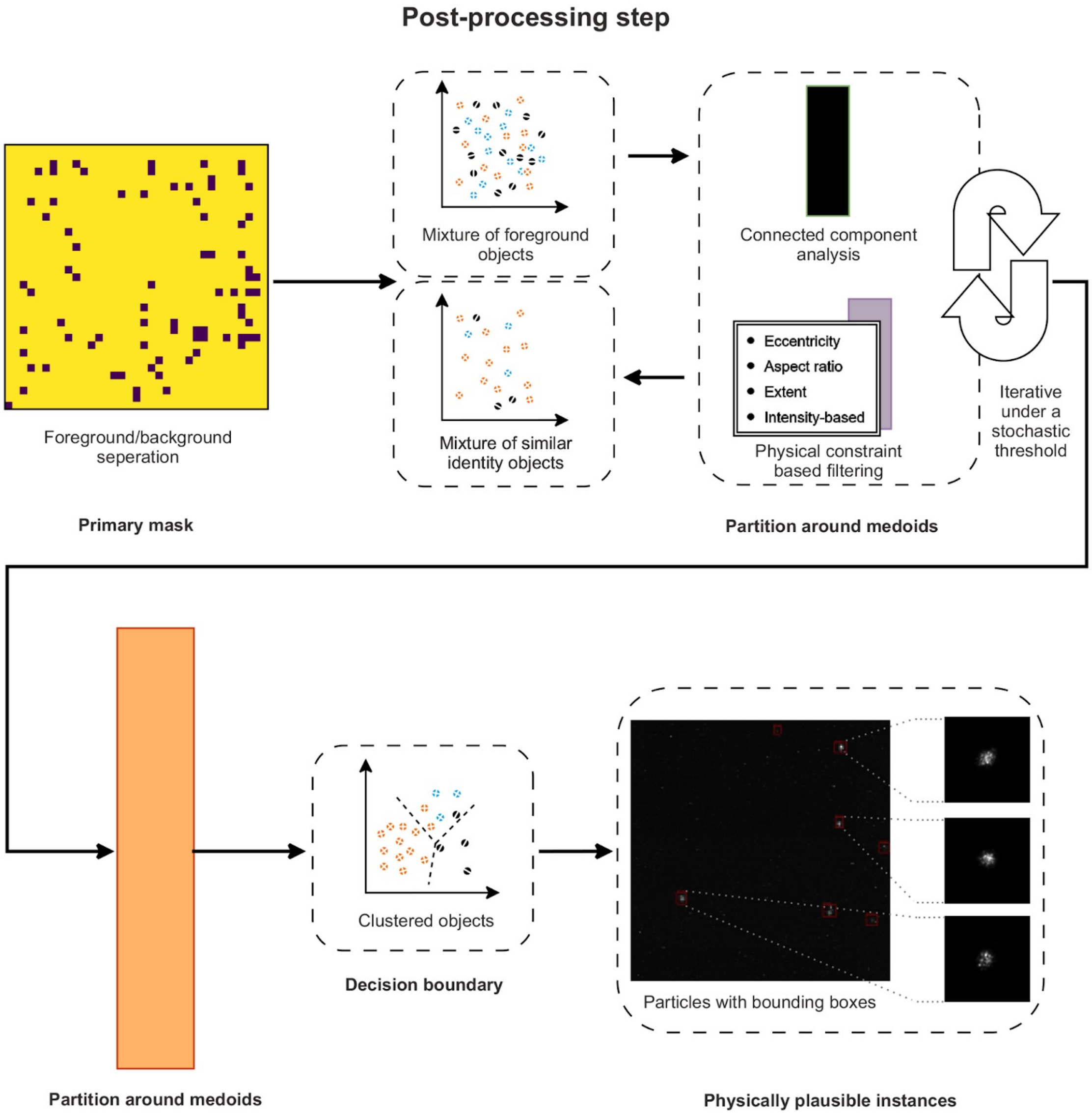

Supplementary Figure 27. Post-segmentation analysis for separation of heterogeneous foreground objects.

bioRxiv preprint doi: https://doi.org/10.64898/2026.05.31.729018; this version posted July 21, 2026. The copyright holder for this preprint (which was not certified by peer review) is the author/funder, who has granted bioRxiv a license to display the preprint in perpetuity. It is made available under a CC-BY 4.0 International license.

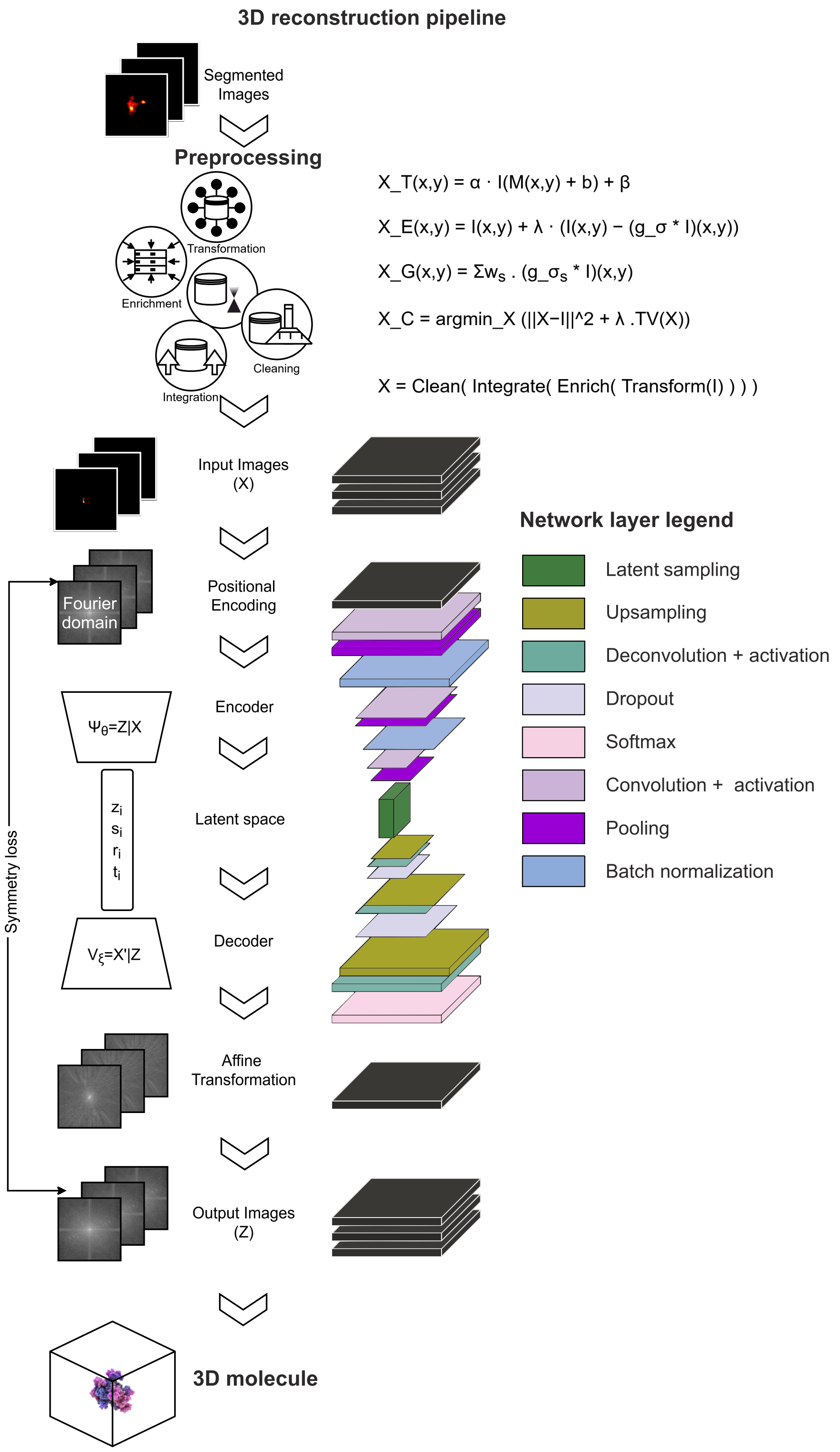

Supplementary Figure 28. End-to-end *ab initio* 3D reconstruction pipeline from preprocessed 2D images.

bioRxiv preprint doi: https://doi.org/10.64898/2026.05.31.729018; this version posted July 21, 2026. The copyright holder for this preprint (which was not certified by peer review) is the author/funder, who has granted bioRxiv a license to display the preprint in perpetuity. It is made available under a CC-BY 4.0 International license.

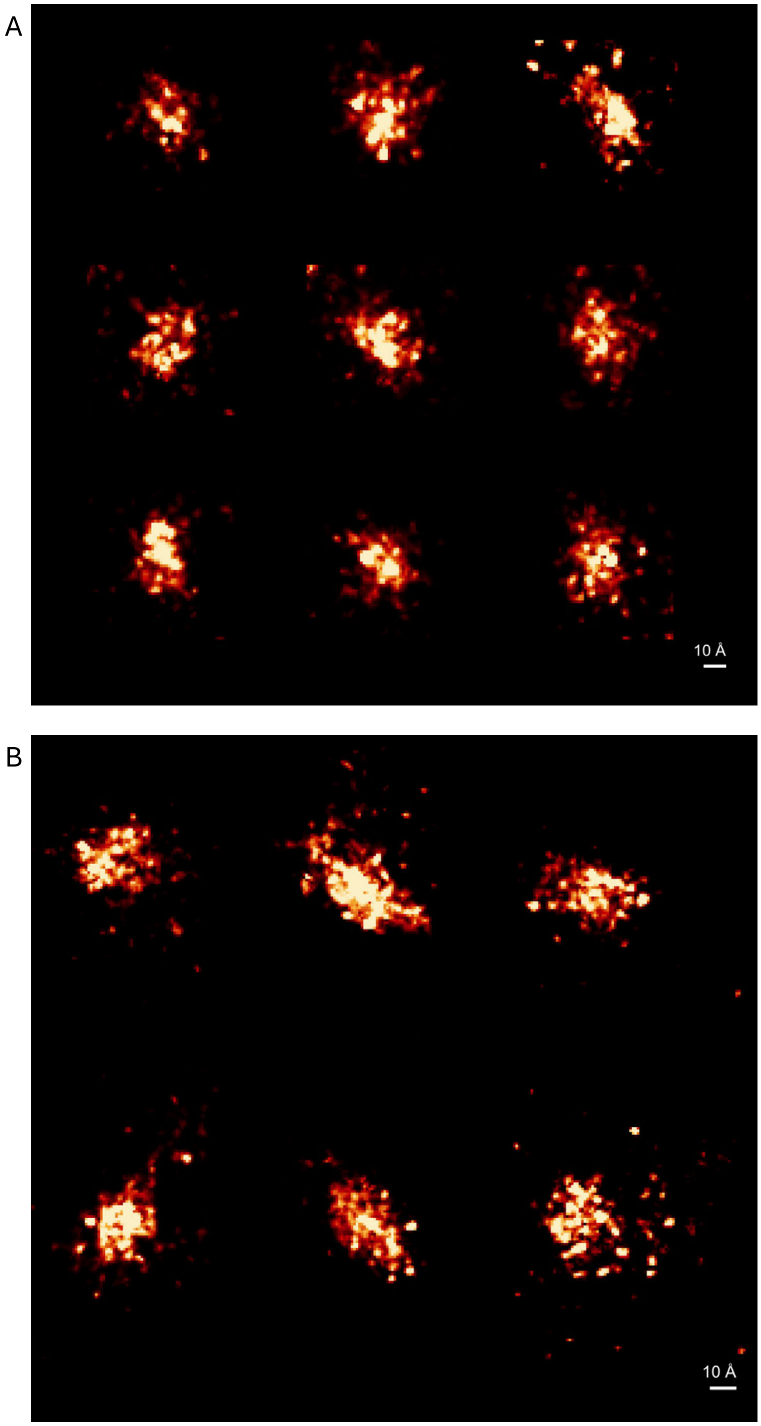

Supplementary Figure 29. Additional GFP examples at different expansion factors.

bioRxiv preprint doi: https://doi.org/10.64898/2026.05.31.729018; this version posted July 21, 2026. The copyright holder for this preprint (which was not certified by peer review) is the author/funder, who has granted bioRxiv a license to display the preprint in perpetuity. It is made available under a CC-BY 4.0 International license.

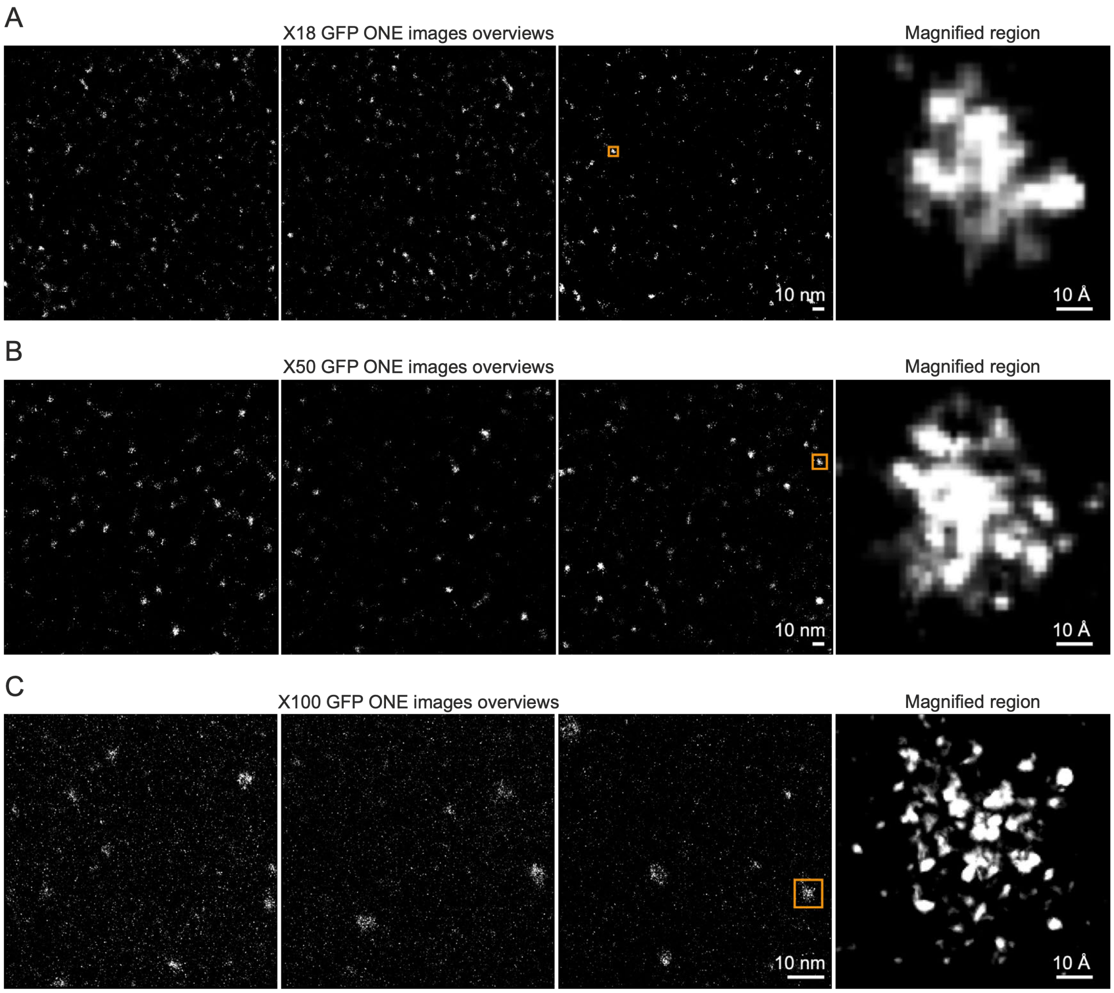

Supplementary Figure 30. GFP imaged with ONE microscopy across expansion factors.

bioRxiv preprint doi: https://doi.org/10.64898/2026.05.31.729018; this version posted July 21, 2026. The copyright holder for this preprint (which was not certified by peer review) is the author/funder, who has granted bioRxiv a license to display the preprint in perpetuity. It is made available under a CC-BY 4.0 International license.

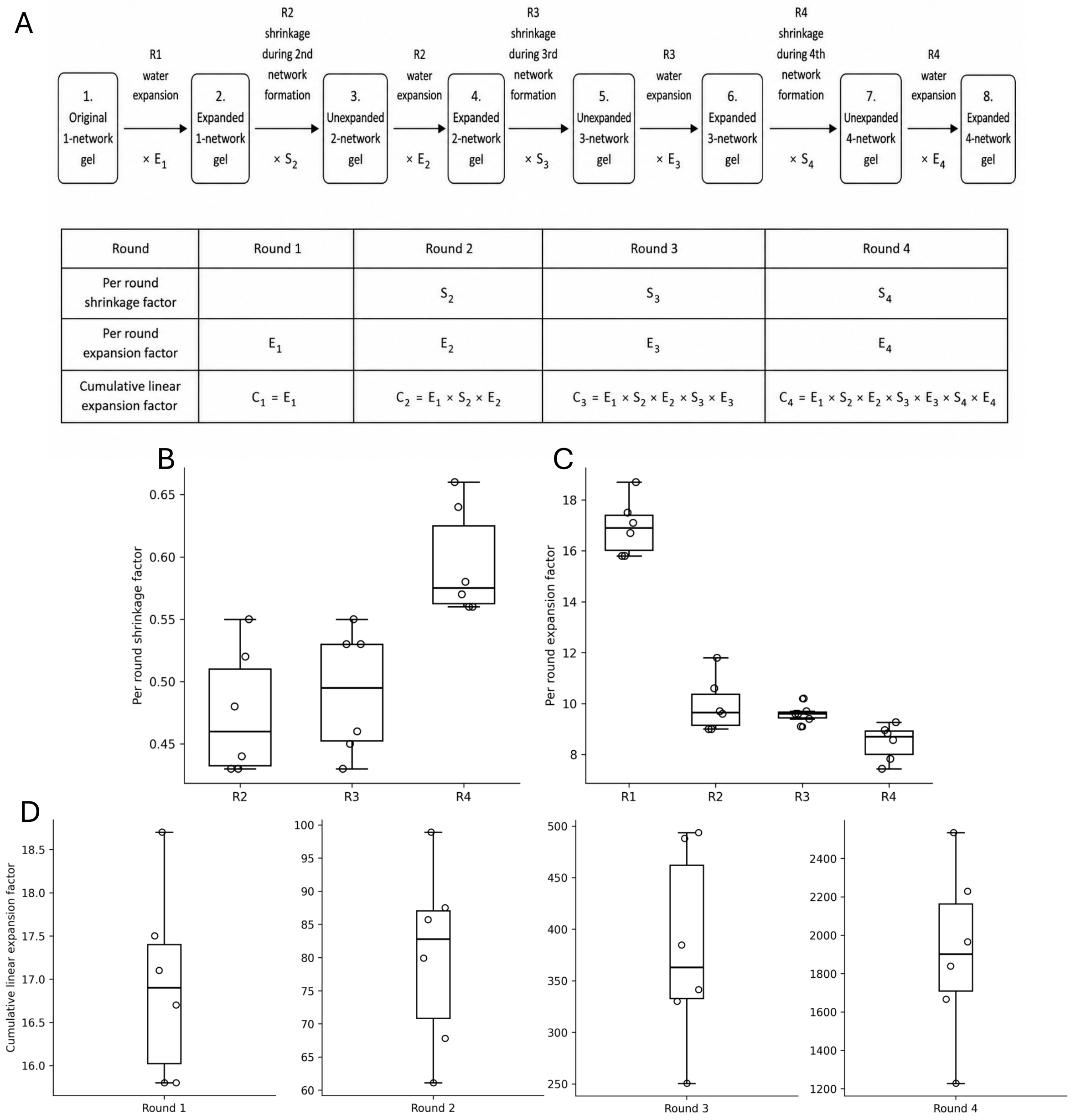

Round	Round 1	Round 2	Round 3	Round 4
Per round shrinkage factor		S_2	S_3	S_4
Per round expansion factor	E_1	E_2	E_3	E_4
Cumulative linear expansion factor	$C_1 = E_1$	$C_2 = E_1 \times S_2 \times E_2$	$C_3 = E_1 \times S_2 \times E_2 \times S_3 \times E_3$	$C_4 = E_1 \times S_2 \times E_2 \times S_3 \times E_3 \times S_4 \times E_4$

Supplementary Figure 31. Four-network gels generated using a modified 1000ExM process.

bioRxiv preprint doi: https://doi.org/10.64898/2026.05.31.729018; this version posted July 21, 2026. The copyright holder for this preprint (which was not certified by peer review) is the author/funder, who has granted bioRxiv a license to display the preprint in perpetuity. It is made available under a CC-BY 4.0 International license.

E

Gel 1

Gel 2

Gel 3

Gel 4

Gel 5

Gel 6

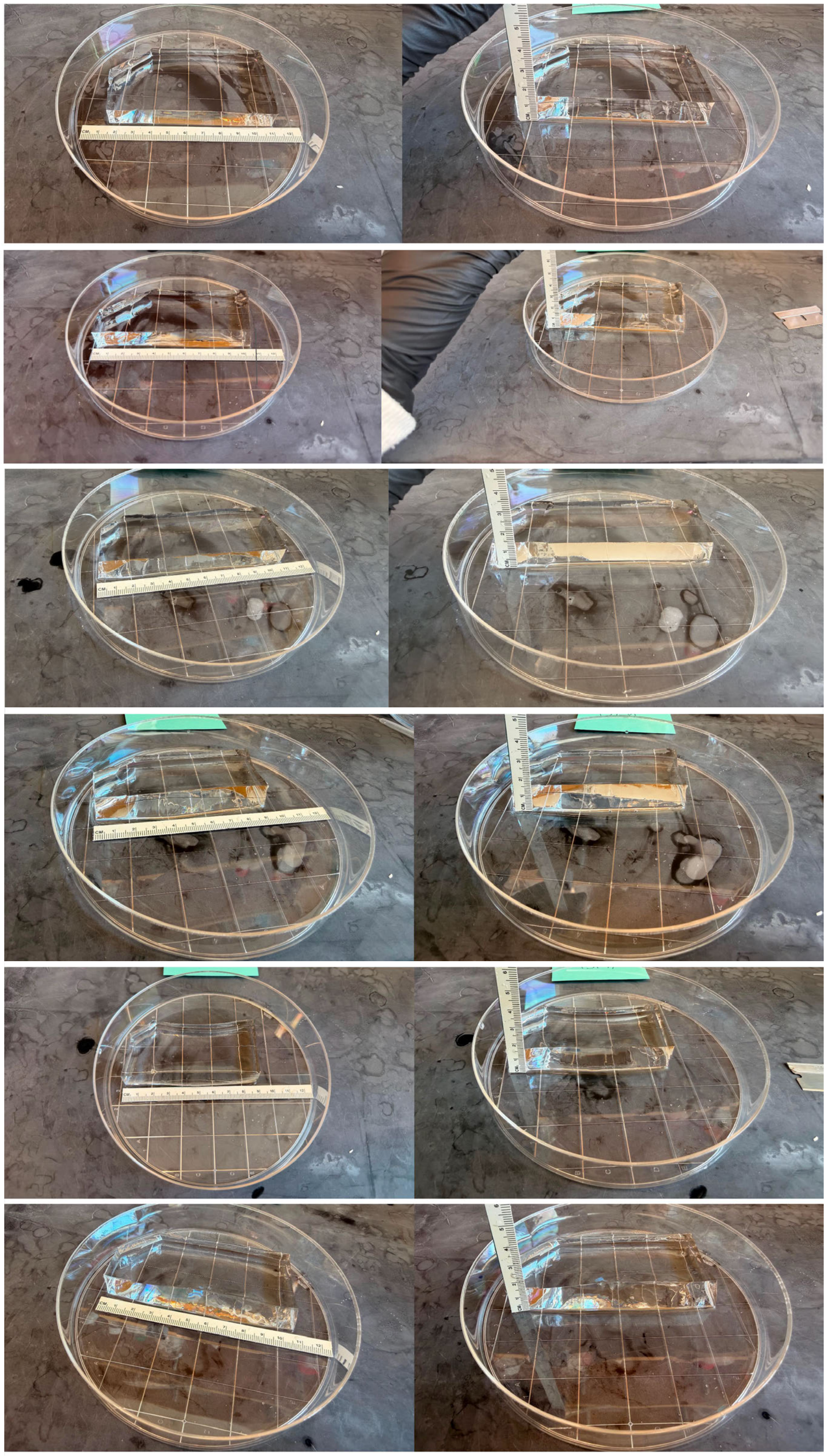

Supplementary Figure 31. Continued.

bioRxiv preprint doi: https://doi.org/10.64898/2026.05.31.729018; this version posted July 21, 2026. The copyright holder for this preprint (which was not certified by peer review) is the author/funder, who has granted bioRxiv a license to display the preprint in perpetuity. It is made available under a CC-BY 4.0 International license.

F

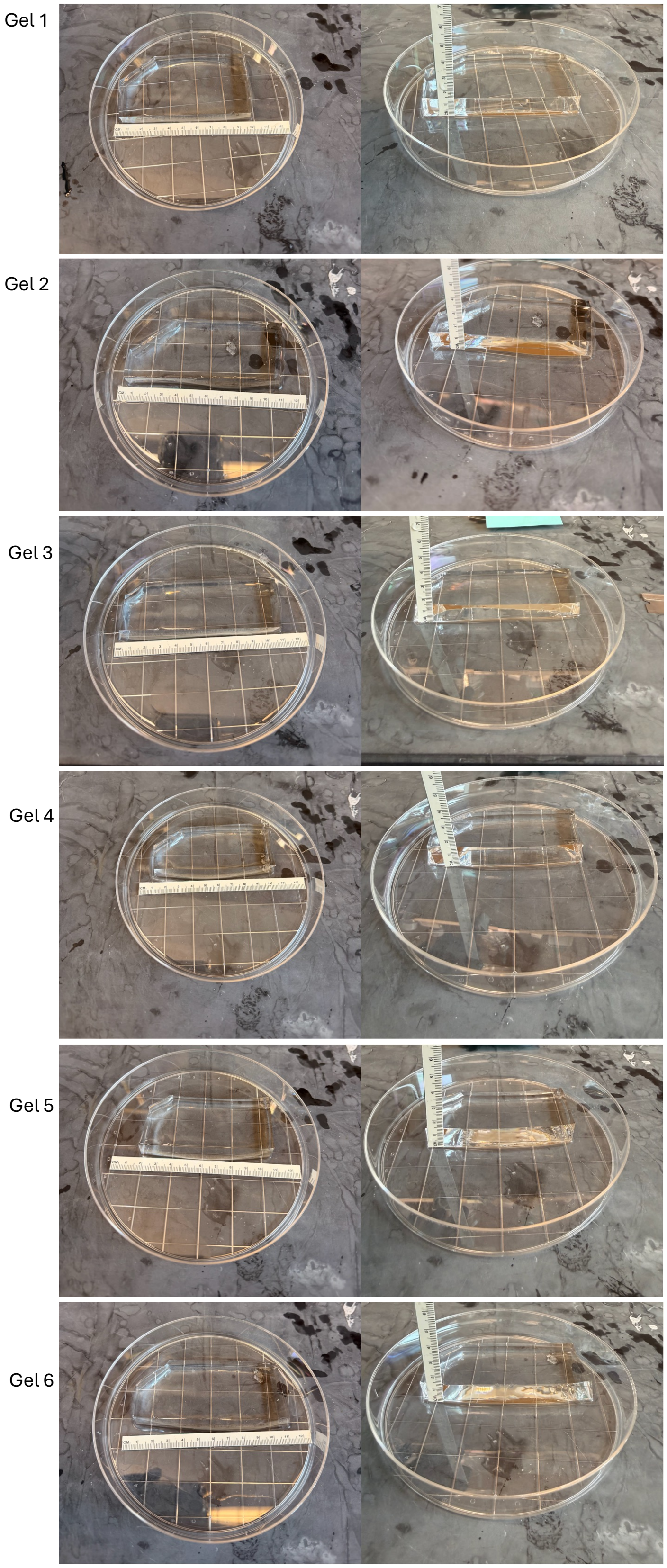

Supplementary Figure 31. Continued.

bioRxiv preprint doi: https://doi.org/10.64898/2026.05.31.729018; this version posted July 21, 2026. The copyright holder for this preprint (which was not certified by peer review) is the author/funder, who has granted bioRxiv a license to display the preprint in perpetuity. It is made available under a CC-BY 4.0 International license.

gel_region	Z thickness (µm)	Y width (µm)	X height (µm)	Volume (µm³)
gel1:region1	4.4	279.09	279.09	3.42×10^5
gel1:region2	4.4	279.09	279.09	3.42×10^5
gel2:region1	3.4	139.59	232.59	1.10×10^5
gel2:region2	3.4	226.49	131.4	1.01×10^5
gel2:region3	3.4	223.31	134.95	1.02×10^5
gel2:region4	3.4	179.18	90.82	5.53×10^4
gel3:region1	2.2	1906.59	46.59	1.95×10^5
gel3:region2	2.2	260.98	340.42	1.95×10^5

Table S1: x, y, and z dimensions and total volume of the eight imaged 3D regions

gel_region	Atto647N emitters	Fluorescein emitters	Fluorescein / Atto647N
gel1:region1	584,761	436,827	**0.75**
gel1:region2	589,176	437,280	**0.74**
gel2:region1	172,503	145,811	**0.85**
gel2:region2	1,309,470	709,989	**0.54**
gel2:region3	472,152	152,744	**0.32**
gel2:region4	98,142	82,268	**0.84**
gel3:region1	349,057	287,299	**0.82**
gel3:region2	17,664,588	16,559,923	**0.94**

Table S2: Atto647N and fluorescein emitter counts after local maxima detection

gel_region	Atto647N emitters	Fluorescein emitters	Fluorescein / Atto647N
gel1:region1	6,280	150,786	**24**
gel1:region2	6,773	154,567	**22.8**
gel2:region1	5,415	35,235	**6.51**
gel2:region2	11,688	50,809	**4.35**
gel2:region3	3,998	23,157	**5.79**
gel2:region4	351	8,863	**25.3**
gel3:region1	4,962	36,289	**7.31**
gel3:region2	9,664	128,097	**13.3**

Table S3: Atto647N and fluorescein emitter counts after local maxima detection and intensity- and size-based filtering

gel_region	Atto647N emitters	Fluorescein emitters	Fluorescein / Atto647N
gel1:region1	4,445	31,567	**7.1**
gel1:region2	4,821	32,166	**6.67**
gel2:region1	3,254	7,206	**2.21**
gel2:region2	6,074	13,444	**2.21**
gel2:region3	2,133	8,573	**4.02**
gel2:region4	210	3,542	**16.9**
gel3:region1	2,225	8,161	**3.67**
gel3:region2	4,264	20,736	**4.86**

Table S4: Atto647N and fluorescein emitter counts after local maxima detection, intensity- and size-based filtering, and Gaussian shape filtering

bioRxiv preprint doi: https://doi.org/10.64898/2026.05.31.729018; this version posted July 21, 2026. The copyright holder for this preprint (which was not certified by peer review) is the author/funder, who has granted bioRxiv a license to display the preprint in perpetuity. It is made available under a CC-BY 4.0 International license.

Atto647N to Fluorescein

subset	k (neighbor order)	n (distance pairs)	Mean (µm)	STD (µm)	Median (µm)	P25 (µm)	P75 (µm)	P5 (µm)	P95 (µm)	P1 (µm)	P99 (µm)	Histogram peak (µm)
all_gels	1	27426	1.249	1.231	1.004	0.701	1.4	0.384	2.638	0.203	7.127	0.825
all_gels	2	27426	1.758	1.547	1.416	1.101	1.876	0.76	3.538	0.599	9.48	1.175
all_gels	3	27426	2.126	1.822	1.704	1.365	2.247	1.021	4.221	0.825	10.906	1.425
all_gels	4	27426	2.433	2.149	1.945	1.578	2.557	1.218	4.775	1.025	11.899	1.675
all_gels	5	27426	2.7	2.365	2.158	1.754	2.838	1.384	5.294	1.198	12.963	1.725

Fluorescein to Fluorescein

subset	k (neighbor order)	n (distance pairs)	Mean (µm)	STD (µm)	Median (µm)	P25 (µm)	P75 (µm)	P5 (µm)	P95 (µm)	P1 (µm)	P99 (µm)	Histogram peak (µm)
all_gels	1	125395	1.101	0.525	0.986	0.774	1.275	0.61	1.939	0.567	2.981	0.825
all_gels	2	125395	1.476	0.671	1.339	1.093	1.671	0.825	2.562	0.692	3.914	1.225
all_gels	3	125395	1.769	0.785	1.6	1.33	1.963	1.037	3.053	0.874	4.732	1.425
all_gels	4	125395	2.015	0.889	1.811	1.534	2.222	1.221	3.463	1.038	5.387	1.675
all_gels	5	125395	2.232	0.978	1.998	1.698	2.451	1.38	3.826	1.2	5.968	1.875

Table S5. Pooled k-nearest neighbor (kNN) distance statistics (k = 1–5) across all gels and all regions (see **Supplementary Figure 19A).**

Atto647N to Fluorescein

subset	k (neighbor order)	n (distance pairs)	Mean (µm)	STD (µm)	Median (µm)	P25 (µm)	P75 (µm)	P5 (µm)	P95 (µm)	P1 (µm)	P99 (µm)	Histogram peak (µm)
gel1	1	9266	0.986	0.704	0.883	0.643	1.186	0.35	1.841	0.203	2.834	0.825
gel1	2	9266	1.381	0.868	1.24	1.017	1.559	0.713	2.311	0.583	3.569	1.075
gel1	3	9266	1.665	1.004	1.5	1.249	1.832	0.96	2.72	0.8	4.21	1.425
gel1	4	9266	1.898	1.136	1.691	1.437	2.06	1.153	3.124	0.989	4.777	1.475
gel1	5	9266	2.101	1.26	1.872	1.602	2.256	1.305	3.45	1.151	5.531	1.725

Fluorescein to Fluorescein

subset	k (neighbor order)	n (distance pairs)	Mean (µm)	STD (µm)	Median (µm)	P25 (µm)	P75 (µm)	P5 (µm)	P95 (µm)	P1 (µm)	P99 (µm)	Histogram peak (µm)
gel1	1	63733	1.021	0.422	0.937	0.758	1.182	0.609	1.669	0.567	2.295	0.825
gel1	2	63733	1.342	0.53	1.254	1.047	1.515	0.809	2.101	0.683	3.025	1.175
gel1	3	63733	1.592	0.606	1.493	1.268	1.758	1.011	2.484	0.862	3.559	1.425
gel1	4	63733	1.8	0.678	1.681	1.453	1.961	1.181	2.832	1.019	4.005	1.625
gel1	5	63733	1.983	0.739	1.842	1.608	2.139	1.329	3.131	1.17	4.415	1.675

Table S6. Pooled k-nearest neighbor (kNN) distance statistics (k = 1–5) across all regions from gel 1 (see **Supplementary Figure 19B.i**).

Atto647N to Fluorescein

subset	k (neighbor order)	n (distance pairs)	Mean (µm)	STD (µm)	Median (µm)	P25 (µm)	P75 (µm)	P5 (µm)	P95 (µm)	P1 (µm)	P99 (µm)	Histogram peak (µm)
gel2	1	11671	1.443	1.643	1.079	0.757	1.502	0.41	3.404	0.22	9.989	0.825
gel2	2	11671	1.981	2.065	1.479	1.163	1.966	0.803	4.533	0.608	12.921	1.225
gel2	3	11671	2.368	2.444	1.763	1.425	2.313	1.047	5.313	0.843	15.236	1.475
gel2	4	11671	2.699	2.92	2.004	1.632	2.632	1.252	5.988	1.05	16.898	1.675
gel2	5	11671	2.981	3.213	2.212	1.811	2.909	1.431	6.686	1.207	19.129	1.925

Fluorescein to Fluorescein

subset	k (neighbor order)	n (distance pairs)	Mean (µm)	STD (µm)	Median (µm)	P25 (µm)	P75 (µm)	P5 (µm)	P95 (µm)	P1 (µm)	P99 (µm)	Histogram peak (µm)
gel2	1	32765	1.11	0.509	1.017	0.79	1.309	0.61	1.887	0.567	2.55	0.825
gel2	2	32765	1.478	0.628	1.383	1.119	1.706	0.835	2.341	0.693	3.266	1.225
gel2	3	32765	1.76	0.733	1.653	1.375	1.996	1.061	2.728	0.881	3.747	1.475
gel2	4	32765	1.995	0.831	1.866	1.58	2.239	1.252	3.031	1.058	4.205	1.675
gel2	5	32765	2.205	0.91	2.063	1.756	2.464	1.413	3.323	1.232	4.608	1.925

Table S7. Pooled k-nearest neighbor (kNN) distance statistics (k = 1–5) across all regions from gel 2 (see **Supplementary Figure 19B.ii**).

Atto647N to Fluorescein

subset	k (neighbor order)	n (distance pairs)	Mean (µm)	STD (µm)	Median (µm)	P25 (µm)	P75 (µm)	P5 (µm)	P95 (µm)	P1 (µm)	P99 (µm)	Histogram peak (µm)
gel3	1	6489	1.274	0.821	1.082	0.734	1.592	0.384	2.888	0.203	4.344	0.825
gel3	2	6489	1.896	1.026	1.638	1.219	2.274	0.817	3.958	0.612	5.745	1.425
gel3	3	6489	2.348	1.181	2.035	1.568	2.743	1.112	4.806	0.869	6.631	1.825
gel3	4	6489	2.721	1.309	2.36	1.857	3.146	1.367	5.466	1.112	7.398	2.025
gel3	5	6489	3.052	1.428	2.653	2.113	3.506	1.598	6.118	1.317	8.041	2.275

Fluorescein to Fluorescein

subset	k (neighbor order)	n (distance pairs)	Mean (µm)	STD (µm)	Median (µm)	P25 (µm)	P75 (µm)	P5 (µm)	P95 (µm)	P1 (µm)	P99 (µm)	Histogram peak (µm)
gel3	1	28897	1.267	0.684	1.085	0.815	1.495	0.612	2.548	0.567	3.942	0.825
gel3	2	28897	1.768	0.872	1.557	1.203	2.055	0.862	3.463	0.707	5.089	1.425
gel3	3	28897	2.169	1.013	1.911	1.521	2.492	1.118	4.117	0.916	5.985	1.675
gel3	4	28897	2.51	1.134	2.201	1.795	2.867	1.363	4.708	1.129	6.921	1.925
gel3	5	28897	2.81	1.24	2.458	2.025	3.209	1.571	5.232	1.329	7.704	2.075

Table S8. Pooled k-nearest neighbor (kNN) distance statistics (k = 1–5) across all regions from gel 3 (see **Supplementary Figure 19B.iii**).

bioRxiv preprint doi: https://doi.org/10.64898/2026.05.31.729018; this version posted July 21, 2026. The copyright holder for this preprint (which was not certified by peer review) is the author/funder, who has granted bioRxiv a license to display the preprint in perpetuity. It is made available under a CC-BY 4.0 International license.

Atto647N to Fluorescein

k (neighbor order)	Mean (µm)	STD (µm)	Median (µm)	P25 (µm)	P75 (µm)	P5 (µm)	P95 (µm)	KS tests with p < 0.001 (out of 100)	KS tests with p ≥ 0.001 (out of 100)
1	0.089	0.003	0.089	0.087	0.091	0.085	0.094	100	0
2	0.113	0.003	0.113	0.112	0.115	0.108	0.118	100	0
3	0.129	0.003	0.129	0.127	0.13	0.124	0.133	100	0
4	0.144	0.002	0.144	0.142	0.145	0.141	0.148	100	0
5	0.153	0.002	0.153	0.152	0.155	0.15	0.157	100	0

Fluorescein to Fluorescein

k (neighbor order)	Mean (µm)	STD (µm)	Median (µm)	P25 (µm)	P75 (µm)	P5 (µm)	P95 (µm)	KS tests with p < 0.001 (out of 100)	KS tests with p ≥ 0.001 (out of 100)
1	0.135	0.001	0.135	0.134	0.136	0.133	0.137	100	0
2	0.193	0.002	0.193	0.192	0.194	0.19	0.196	100	0
3	0.227	0.002	0.227	0.225	0.228	0.224	0.229	100	0
4	0.249	0.002	0.249	0.248	0.25	0.247	0.252	100	0
5	0.264	0.002	0.264	0.263	0.265	0.261	0.267	100	0

Table S9: KS D randomization statistics by kNN rank (1–5) from all gels (see **Supplementary Figure 20**).

Atto647N to Fluorescein

k (neighbor order)	Mean (µm)	STD (µm)	Median (µm)	P25 (µm)	P75 (µm)	P5 (µm)	P95 (µm)	KS tests with p < 0.001 (out of 100)	KS tests with p ≥ 0.001 (out of 100)
1	0.224	0.005	0.223	0.22	0.227	0.217	0.234	100	0
2	0.275	0.004	0.275	0.273	0.278	0.268	0.283	100	0
3	0.296	0.004	0.296	0.293	0.299	0.29	0.302	100	0
4	0.322	0.004	0.321	0.319	0.324	0.315	0.328	100	0
5	0.34	0.004	0.34	0.337	0.343	0.333	0.347	100	0

Fluorescein to Fluorescein

k (neighbor order)	Mean (µm)	STD (µm)	Median (µm)	P25 (µm)	P75 (µm)	P5 (µm)	P95 (µm)	KS tests with p < 0.001 (out of 100)	KS tests with p ≥ 0.001 (out of 100)
1	0.205	0.002	0.205	0.203	0.206	0.201	0.209	100	0
2	0.283	0.003	0.283	0.281	0.284	0.279	0.287	100	0
3	0.326	0.002	0.326	0.324	0.327	0.323	0.33	100	0
4	0.359	0.002	0.358	0.357	0.36	0.355	0.362	100	0
5	0.382	0.003	0.382	0.38	0.384	0.378	0.386	100	0

Table S10: KS D randomization statistics by kNN rank (1–5) from gel 1 (see **Supplementary Figure 21**).

Atto647N to Fluorescein

k (neighbor order)	Mean (µm)	STD (µm)	Median (µm)	P25 (µm)	P75 (µm)	P5 (µm)	P95 (µm)	KS tests with p < 0.001 (out of 100)	KS tests with p ≥ 0.001 (out of 100)
1	0.224	0.005	0.223	0.22	0.227	0.217	0.234	100	0
2	0.275	0.004	0.275	0.273	0.278	0.268	0.283	100	0
3	0.296	0.004	0.296	0.293	0.299	0.29	0.302	100	0
4	0.322	0.004	0.321	0.319	0.324	0.315	0.328	100	0
5	0.34	0.004	0.34	0.337	0.343	0.333	0.347	100	0

Fluorescein to Fluorescein

k (neighbor order)	Mean (µm)	STD (µm)	Median (µm)	P25 (µm)	P75 (µm)	P5 (µm)	P95 (µm)	KS tests with p < 0.001 (out of 100)	KS tests with p ≥ 0.001 (out of 100)
1	0.205	0.002	0.205	0.203	0.206	0.201	0.209	100	0
2	0.283	0.003	0.283	0.281	0.284	0.279	0.287	100	0
3	0.326	0.002	0.326	0.324	0.327	0.323	0.33	100	0
4	0.359	0.002	0.358	0.357	0.36	0.355	0.362	100	0
5	0.382	0.003	0.382	0.38	0.384	0.378	0.386	100	0

Table S11: KS D randomization statistics by kNN rank (1–5) from gel 2 (see **Supplementary Figure 22**).

Atto647N to Fluorescein

k (neighbor order)	Mean (µm)	STD (µm)	Median (µm)	P25 (µm)	P75 (µm)	P5 (µm)	P95 (µm)	KS tests with p < 0.001 (out of 100)	KS tests with p ≥ 0.001 (out of 100)
1	0.06	0.006	0.061	0.056	0.064	0.052	0.069	100	0
2	0.075	0.005	0.075	0.072	0.079	0.067	0.083	100	0
3	0.096	0.005	0.096	0.093	0.099	0.087	0.104	100	0
4	0.108	0.005	0.108	0.104	0.11	0.101	0.115	100	0
5	0.12	0.005	0.12	0.117	0.124	0.113	0.128	100	0

Fluorescein to Fluorescein

k (neighbor order)	Mean (µm)	STD (µm)	Median (µm)	P25 (µm)	P75 (µm)	P5 (µm)	P95 (µm)	KS tests with p < 0.001 (out of 100)	KS tests with p ≥ 0.001 (out of 100)
1	0.122	0.003	0.122	0.12	0.125	0.117	0.127	100	0
2	0.102	0.003	0.102	0.1	0.104	0.096	0.107	100	0
3	0.134	0.004	0.134	0.131	0.137	0.128	0.14	100	0
4	0.156	0.004	0.156	0.153	0.158	0.15	0.163	100	0
5	0.174	0.003	0.174	0.172	0.176	0.169	0.179	100	0

Table S12: KS D randomization statistics by kNN rank (1–5) from gel 3 (see **Supplementary Figure 23**).

bioRxiv preprint doi: https://doi.org/10.64898/2026.05.31.729018; this version posted July 21, 2026. The copyright holder for this preprint (which was not certified by peer review) is the author/funder, who has granted bioRxiv a license to display the preprint in perpetuity. It is made available under a CC-BY 4.0 International license.

All Gels	n (molecules)	Mean	STD	Median	P25	P75	P10	P90
Single-molecule expansion factor	6284	1055.92	21.554	1058.372	1043.009	1071.639	1027.077	1081.537
RMSD (x-axis, nm)	6284	0.317	0.121	0.308	0.228	0.392	0.17	0.475
RMSD (y-axis, nm)	6284	0.316	0.12	0.306	0.228	0.393	0.166	0.475
RMSD (z-axis, nm)	6284	0.325	0.126	0.312	0.232	0.404	0.174	0.493
Total RMSD (nm)	6284	0.573	0.151	0.572	0.476	0.668	0.372	0.764

Table S13: Summary statistics of single-molecule expansion factors (**Supplementary Figure 11A**), per-axis RMSD (**Supplementary Figure 10A**), and total RMSD (see **Supplementary Figure 9A**, boxplots; **Supplementary Figure 12A**, histograms) for all putative peptides pooled across all gels and regions.

_	n (molecules)	Mean	STD	Median	P25	P75	P10	P90
**Gel 1**								
Single-molecule expansion factor	3155	1054.678	20.745	1057.095	1042.238	1069.63	1026.919	1079.035
RMSD (x-axis, nm)	3155	0.313	0.117	0.305	0.225	0.386	0.17	0.466
RMSD (y-axis, nm)	3155	0.314	0.117	0.305	0.23	0.387	0.17	0.471
RMSD (z-axis, nm)	3155	0.318	0.118	0.308	0.231	0.393	0.175	0.474
Total RMSD (nm)	3155	0.565	0.14	0.567	0.476	0.655	0.378	0.742
**Gel 2**								
Single-molecule expansion factor	1918	1055.137	22.519	1057.447	1041.44	1071.891	1025.949	1081.618
RMSD (x-axis, nm)	1918	0.313	0.123	0.303	0.225	0.393	0.164	0.48
RMSD (y-axis, nm)	1918	0.31	0.12	0.304	0.219	0.389	0.156	0.469
RMSD (z-axis, nm)	1918	0.322	0.13	0.305	0.225	0.405	0.166	0.493
Total RMSD (nm)	1918	0.565	0.16	0.562	0.461	0.665	0.352	0.772
**Gel 3**								
Single-molecule expansion factor	1211	1060.396	21.496	1063.804	1047.249	1076.174	1030.608	1085.43
RMSD (x-axis, nm)	1211	0.331	0.124	0.32	0.241	0.405	0.184	0.496
RMSD (y-axis, nm)	1211	0.329	0.129	0.315	0.231	0.414	0.17	0.501
RMSD (z-axis, nm)	1211	0.349	0.137	0.332	0.251	0.444	0.182	0.53
Total RMSD (nm)	1211	0.604	0.158	0.6	0.5	0.708	0.401	0.811

Table S14: Summary statistics of single-molecule expansion factors (**Supplementary Figure 11B**), per-axis RMSD (**Supplementary Figure 10B**), and total RMSD (see **Supplementary Figure 9A**, boxplots; **Supplementary Figure 12A**, histograms) for all putative peptides, stratified by gel (Gel 1-3).

bioRxiv preprint doi: https://doi.org/10.64898/2026.05.31.729018; this version posted July 21, 2026. The copyright holder for this preprint (which was not certified by peer review) is the author/funder, who has granted bioRxiv a license to display the preprint in perpetuity. It is made available under a CC-BY 4.0 International license.

_	n (molecules)	Mean	STD	Median	P25	P75	P10	P90
**Gel 1, Region 1**								
Single-molecule expansion factor	1531	1055.032	20.607	1057.373	1042.318	1070.055	1027.387	1079.503
RMSD (x-axis, nm)	1531	0.315	0.119	0.306	0.226	0.387	0.171	0.474
RMSD (y-axis, nm)	1531	0.314	0.116	0.305	0.23	0.389	0.169	0.472
RMSD (z-axis, nm)	1531	0.32	0.119	0.309	0.231	0.397	0.176	0.476
Total RMSD (nm)	1531	0.568	0.14	0.568	0.477	0.658	0.385	0.746
**Gel 1, Region 2**								
Single-molecule expansion factor	1624	1054.344	20.876	1056.945	1042.213	1069.255	1026.531	1078.649
RMSD (x-axis, nm)	1624	0.312	0.115	0.304	0.225	0.385	0.168	0.461
RMSD (y-axis, nm)	1624	0.314	0.117	0.304	0.231	0.386	0.171	0.47
RMSD (z-axis, nm)	1624	0.317	0.117	0.308	0.232	0.392	0.173	0.472
Total RMSD (nm)	1624	0.563	0.14	0.566	0.475	0.65	0.369	0.741
**Gel 2, Region 1**								
Single-molecule expansion factor	523	1053.636	22.632	1055.618	1037.743	1071.221	1024.864	1081.227
RMSD (x-axis, nm)	523	0.301	0.116	0.293	0.219	0.369	0.16	0.466
RMSD (y-axis, nm)	523	0.303	0.117	0.292	0.217	0.382	0.151	0.452
RMSD (z-axis, nm)	523	0.322	0.137	0.306	0.222	0.41	0.165	0.512
Total RMSD (nm)	523	0.553	0.161	0.552	0.447	0.663	0.34	0.766
**Gel 2, Region 2**								
Single-molecule expansion factor	849	1050.75	21.995	1052.864	1037.705	1065.365	1022.149	1077.294
RMSD (x-axis, nm)	849	0.296	0.112	0.289	0.217	0.367	0.159	0.445
RMSD (y-axis, nm)	849	0.294	0.114	0.292	0.21	0.369	0.15	0.438
RMSD (z-axis, nm)	849	0.302	0.121	0.287	0.21	0.378	0.156	0.462
Total RMSD (nm)	849	0.533	0.146	0.537	0.439	0.624	0.332	0.716
**Gel 2, Region 3**								
Single-molecule expansion factor	497	1062.861	21.276	1065.479	1050.542	1077.634	1034.341	1088.583
RMSD (x-axis, nm)	497	0.35	0.139	0.334	0.25	0.437	0.184	0.533
RMSD (y-axis, nm)	497	0.338	0.127	0.33	0.245	0.419	0.176	0.505
RMSD (z-axis, nm)	497	0.348	0.133	0.326	0.251	0.433	0.193	0.52
Total RMSD (nm)	497	0.619	0.166	0.621	0.509	0.722	0.401	0.85
**Gel 2, Region 4**								
Single-molecule expansion factor	49	1068.823	16.836	1070.129	1058.476	1079.936	1043.815	1088.267
RMSD (x-axis, nm)	49	0.366	0.135	0.363	0.257	0.46	0.189	0.532
RMSD (y-axis, nm)	49	0.376	0.129	0.385	0.27	0.457	0.207	0.53
RMSD (z-axis, nm)	49	0.383	0.132	0.385	0.296	0.443	0.235	0.534
Total RMSD (nm)	49	0.675	0.137	0.643	0.589	0.727	0.547	0.881
**Gel 3, Region 1**								
Single-molecule expansion factor	153	1063.514	22.452	1070.043	1050.811	1079.503	1031.755	1086.738
RMSD (x-axis, nm)	153	0.337	0.121	0.313	0.257	0.417	0.192	0.501
RMSD (y-axis, nm)	153	0.344	0.139	0.345	0.241	0.435	0.173	0.517
RMSD (z-axis, nm)	153	0.376	0.151	0.358	0.261	0.48	0.191	0.579
Total RMSD (nm)	153	0.635	0.164	0.656	0.525	0.744	0.416	0.839
**Gel 3, Region 2**								
Single-molecule expansion factor	1058	1059.946	21.327	1063.189	1046.846	1075.58	1030.574	1085.255
RMSD (x-axis, nm)	1058	0.33	0.124	0.321	0.24	0.403	0.18	0.492
RMSD (y-axis, nm)	1058	0.326	0.127	0.313	0.231	0.409	0.169	0.498
RMSD (z-axis, nm)	1058	0.345	0.135	0.329	0.251	0.437	0.179	0.521
Total RMSD (nm)	1058	0.6	0.157	0.596	0.499	0.701	0.399	0.801

Table S15: Summary statistics of single-molecule expansion factors (**Supplementary Figure 11C**), per-axis RMSD (**Supplementary Figure 10C**), and total RMSD (see **Supplementary Figure 9C**, boxplots; **Supplementary Figure 12C**, histograms) for all putative peptides, stratified by imaging region across Gel 1-3 (n=8 regions).

bioRxiv preprint doi: https://doi.org/10.64898/2026.05.31.729018; this version posted July 21, 2026. The copyright holder for this preprint (which was not certified by peer review) is the author/funder, who has granted bioRxiv a license to display the preprint in perpetuity. It is made available under a CC-BY 4.0 International license.

Number of MD reference conformations	Mean RMSD (nm)	STD (nm)	Median RMSD (nm)	P25 (nm)	P75 (nm)	P10 (nm)	P90 (nm)	Minimum RMSD (nm)	Maximum RMSD (nm)	n (molecules)
10	0.8324	0.2729	0.8793	0.6835	1.0126	0.3847	1.1421	0.1366	1.6169	6284
100	0.681	0.1943	0.7013	0.5686	0.8085	0.3835	0.91	0.1366	1.323	6284
200	0.6586	0.1835	0.6726	0.5523	0.78	0.3833	0.8787	0.1366	1.3151	6284
300	0.634	0.1738	0.6444	0.5299	0.7485	0.3808	0.8486	0.1366	1.2392	6284
400	0.6201	0.1683	0.6275	0.518	0.728	0.3794	0.8298	0.1366	1.2219	6284
500	0.597	0.1596	0.599	0.4974	0.6997	0.3753	0.7954	0.1366	1.1679	6284
600	0.5846	0.154	0.5842	0.489	0.6831	0.3741	0.7786	0.1366	1.1171	6284
700	0.5795	0.1525	0.5778	0.4832	0.6754	0.3724	0.7724	0.1366	1.1171	6284
800	0.5771	0.1523	0.575	0.4804	0.6719	0.3723	0.7712	0.1366	1.1171	6284
900	0.5747	0.151	0.5733	0.4788	0.669	0.3722	0.7668	0.1366	1.1171	6284
1001	0.5725	0.1509	0.5718	0.4759	0.6678	0.3715	0.7645	0.1366	1.1171	6284

Table S16. Summary statistics of total RMSD distributions as a function of the number of molecular-dynamics (MD) reference conformations (see **Supplementary Figure 13**, boxplots; **Supplementary Figure 14**, histograms).

bioRxiv preprint doi: https://doi.org/10.64898/2026.05.31.729018; this version posted July 21, 2026. The copyright holder for this preprint (which was not certified by peer review) is the author/funder, who has granted bioRxiv a license to display the preprint in perpetuity. It is made available under a CC-BY 4.0 International license.

**Panel B**	18x (ONE)	50x (ONE)	100x (ONE)	100x (confocal)	500x (confocal)	1000x (confocal)	model
Mean	4.434317761	5.210717151	5.157849609	6.177624755	3.900797385	7.090570252	4.88207877
Median	4.220428333	4.269811	4.222855	5.460546	2.408406	5.822544	4.959
Std deviation	1.991380873	3.786436472	3.851486303	3.813227427	4.826060053	6.186493755	1.419420211
Std error	0.02054499	0.122462107	0.1289573	6.67E-03	0.037042528	0.22819226	0.044886008
% CV	44.90839359	72.66632139	74.67232655	61.72643336	123.7198341	87.24959396	29.07409482
90% conf	0.033799843	0.201645797	0.212355577	0.010972448	0.060938286	0.375850696	0.073906027
95% conf	0.04027337	0.240330327	0.253100129	0.013073567	0.072608534	0.447995574	0.088083297
99% conf	0.052934652	0.316094153	0.332907159	0.017182438	0.095432286	0.589355788	0.1158477
N	9395	956	892	326824	16974	735	1000

**Panel F**							
R	0.9255		Coefficient	Std. Error	t	P	VIF
R2	0.8566	y0	0.2809	0.618	0.4545	0.6685	4.7978<
Adj R2	0.8279	a	0.809	0.148	5.4646	0.0028	4.7978<
Standard error of estimate	0.7465						

**Panel G**							
R	0.925		Coefficient	Std. Error	t	P	VIF
R2	8556						
Adj R2	0.8483	y0	0.2613	0.1059	2.4682	0.0227	3.0004
Standard error of estimate	0.3055	a	0.699	0.0569	12.2836	<0.0001	3.0004

**Panel H**		
Mean	1.518872	1.161491304
Median	1.524	1.2634
Std deviation	1.096045975	0.79576071
Std error	0.219209195	0.165927578
% CV	72.16183952	68.51198169
90% conf	0.375076408	0.284948583
95% conf	0.45243455	0.344119637
99% conf	0.613161459	0.467745354
N	25	23
Mann-Whitney Ranksum test	P value	0.2156

**Panel I**		
Mean	0.655143478	0.568928571
Median	0.606	0.549
Std deviation	0.255672923	0.06306863
Std error	0.05331149	0.013762703
% CV	39.02548546	11.08550943
90% conf	0.091552192	0.023739047
95% conf	0.11056348	0.028709077
99% conf	0.150283647	0.039162624
N	23	21
Mann-Whitney Ranksum test	P value	0.3827

Table S17: Statistics elements for **Fig. 2**.

bioRxiv preprint doi: https://doi.org/10.64898/2026.05.31.729018; this version posted July 21, 2026. The copyright holder for this preprint (which was not certified by peer review) is the author/funder, who has granted bioRxiv a license to display the preprint in perpetuity. It is made available under a CC-BY 4.0 International license.

				**Round 1 before expansion length (cm)**	**Round 1 after expansion length (cm)**	**Round 1 expansion factor ($E_1 = C_1$)**
				1	15.8	15.8
				1	15.8	15.8
				1	17.5	17.5
				1	16.7	16.7
				1	17.1	17.1
				1	18.7	18.7
**Round 2 before monomer addition length (cm)**	**Round 2 after gelation length (cm)**	**Round 2 shrinkage factor (S_2)**	**Round 2 before expansion length (cm)**	**Round 2 after expansion length (cm)**	**Round 2 expansion factor (E_2)**	**Round 2 cumulative expansion factor (C_2)**
2.9	1.25	0.43	1	9	9.0	61.1
2.3	1.1	0.48	1	9	9.0	67.8
2.9	1.25	0.43	0.85	9	10.6	79.9
2.7	1.2	0.44	0.8	9.45	11.8	87.5
2.9	1.5	0.52	0.9	8.7	9.7	85.7
2.9	1.6	0.55	1	9.6	9.6	98.9
**Round 3 before monomer addition length (cm)**	**Round 3 after gelation length (cm)**	**Round 3 shrinkage factor (S_3)**	**Round 3 before expansion length (cm)**	**Round 3 after expansion length (cm)**	**Round 3 expansion factor (E_3)**	**Round 3 cumulative expansion factor (C_3)**
1.4	0.6	0.43	1.2	11	9.6	250.5
1.9	1	0.53	1.2	11	9.6	341.3
2.2	1	0.45	1.1	10	9.1	330.0
2.9	1.6	0.55	0.9	9.2	10.2	493.6
1.4	0.65	0.46	0.9	8.7	9.7	384.6
2	1.05	0.53	1.0	9.4	9.4	488.0
**Round 4 before monomer addition length (cm)**	**Round 4 after gelation length (cm)**	**Round 4 shrinkage factor (S_4)**	**Round 4 before expansion length (cm)**	**Round 4 after expansion length (cm)**	**Round 4 expansion factor (E_4)**	**Round 4 cumulative expansion factor (C_4)**
2.2	1.45	0.66	1.25	9.3	7.44	1228.2
2.1	1.35	0.64	1.15	10.3	8.96	1965.1
2.8	1.6	0.57	1.2	10.6	8.83	1665.9
2.6	1.5	0.58	1.15	9	7.83	2228.6
2.6	1.45	0.56	1.05	9	8.57	1838.6
2.5	1.4	0.56	1.1	10.2	9.27	2534.1

Table S18: Measured gel lengths and calculated shrinkage and expansion factors during four-network gel formation.
