# Supplementary material for: Thousandfold Expansion Microscopy
Source: bioRxiv. 2026 Jun 1:2026.05.31.729018. Preprint. [Version 1] doi: 10.64898/2026.05.31.729018 (PMC13252410; doi:10.64898/2026.05.31.729018)
Supplement: Supplementary file 1 [file NIHPP2026.05.31.729018v1-supplement-1.pdf]

bioRxiv preprint doi: https://doi.org/10.64898/2026.05.31.729018; this version posted June 1, 2026. The copyright holder for this preprint (which was not certified by peer review) is the author/funder, who has granted bioRxiv a license to display the preprint in perpetuity. It is made available under a CC-BY 4.0 International license.

# Supplementary Figures

**Supplementary Figure 1. Representative hydrogels at successive expansion stages.**
The hydrogel consists of four interpenetrating polymer networks generated by sequential monomer infiltration, polymerization under deoxygenated conditions (>8 h, 20 °C), and re-expansion in water. Linear expansion was determined by measuring one gel edge with a ruler (minimum spacing 0.1 cm).
(A) First network (~18×). The primary swellable gel was cast from the monomer solution (**Fig. 1A**). At (i) 1×, the gel is colorless and transparent with white specks consistent with trapped air. After expansion in water to (ii) ~18×, the gel is transparent with straight edges and flat surfaces.
(B) Second network (~100× total). The fully expanded ~18× gel was infiltrated with a charged monomer solution and polymerized, yielding a composite gel that contracts to (i) ~10× linear expansion. At this stage, the gel is translucent with spherical bubbles. Re-expansion to (ii) ~100× (~10× from i) produces a transparent gel with straight edges and flat surfaces that stands upright without visible axial bending.
(C) Third network (~500× total). The fully expanded ~100× gel was infiltrated with a charged monomer solution and polymerized, yielding a composite gel that contracts to (i) ~50× linear expansion. The gel is translucent with spherical bubbles. Re-expansion to (ii) ~500× (~10× from i) produces a transparent gel with straight edges and flat surfaces that stands upright without visible axial bending.
(D) Fourth network (~1500× total). The fully expanded ~500× gel was infiltrated with a charged monomer solution and polymerized, yielding a composite gel that contracts to (i) ~250× linear expansion. Re-expansion to (ii) ~1500× (~6× from i) produces a transparent gel with slightly inward-curved (concave) surfaces, indicating resistance to further swelling, perhaps imposed by the covalently crosslinked network. (This did not seem to be gravity-induced: the gel remained rigid and self-supporting, with the slight inward curvature reflecting elastic constraint opposing further swelling, for this specific gel condition.) Upon equilibration in 1× PBS for imaging, the gel contracts to ~1000× linear expansion, and the slight concavity goes away. Furthermore, with optimization of the gelling process (**Supp. Fig. 31**), round 4 gels expand without any perceptible concavity.

**Supplementary Figure 2. Nanobody imaging across different linear expansion factors.** (A) ONE microscopy at ~18×, ~50×, and ~100× expansion, of representative fields of view from representative gels. (B) Confocal imaging at ~100×, ~500×, and ~1000× expansion. Representative datasets are shown, from 2-7 experimental replicates.

**Supplementary Figure 3. mCLING peptide imaging across different linear expansion factors.** (A–C) ONE microscopy at ~18×, ~50×, and ~100× expansion. (D–F) Confocal imaging at ~500×, ~750×, and ~1000× expansion.

**Supplementary Figure 4. Pan-lysine anchoring, proteolytic cleavage, and post-expansion labeling illustrated using the mCLING peptide.**
Gray boxes indicate reactions performed inside the expansion hydrogel; all other steps were performed in solution.
A. Chemical structure of the mCLING peptide.
B.i NHS-acrylate (Acryloyl-X) reacts with primary amines via NHS ester–amine coupling.
B.ii All primary amines on the mCLING peptide, ideally, are functionalized with polymerizable acrylate groups.
C. Hydrogel polymerization (gray box). Acrylate-functionalized amines (abbreviated A) are covalently attached to the gel network, anchoring the peptide at the labeled sites.

bioRxiv preprint doi: https://doi.org/10.64898/2026.05.31.729018; this version posted June 1, 2026. The copyright holder for this preprint (which was not certified by peer review) is the author/funder, who has granted bioRxiv a license to display the preprint in perpetuity. It is made available under a CC-BY 4.0 International license.

D. Proteinase K digestion in which the amide bond between the anchoring site and Atto647N is cleaved. All peptide backbone amide bonds are digested. Fragments lacking an anchoring site are removed during expansion. Because Atto647N is not anchored, that dye is lost.
E. Proteinase K digestion in which the amide bond between the anchoring site and Atto647N is not cleaved. Atto647N remains attached to an anchored fragment and is therefore retained and displaced during expansion according to the anchoring atom. Cleavage of this bond is unlikely due to steric constraints of the Atto647N substituent in the Proteinase K substrate recognition site [53].
F. NHS-ester fluorescein labeling of remaining free primary amines. The blue sphere denotes a primary amine that has been conjugated with fluorescein. In this panel, the full chemical conjugation product is shown.
G.I–III. Labeling outcomes for case D (Atto647N cleaved and removed).
In G and H, the blue sphere is used as a symbolic shorthand indicating an $NH_2$ group conjugated with fluorescein.
G.I Both the N-terminal amine and the lysine side-chain amine are anchored. No free primary amines remain; no fluorescein labeling occurs.
G.II Only the lysine side-chain amine is anchored. The free N-terminal amine is fluorescein-labeled (blue sphere). Upon expansion, the dye is displaced to the position of the lysine anchoring atom.
G.III Only the N-terminal amine is anchored. The lysine side-chain amine is fluorescein-labeled (blue sphere). The dye is displaced to the position of the N-terminal anchoring atom.
H.I–III. Labeling outcomes for case E (Atto647N retained).
Configurations are the same as G.I–III, except that Atto647N remains attached and is displaced according to its anchoring site.

**Supplementary Figure 5. Distance distribution between adjacent lysine side chains.**
Primary amines were functionalized with NHS–ester acrylate to covalently anchor the nitrogen atom to the polymer network. In the schematic, the nitrogen of the first primary amine bearing the Atto647N dye is shown as an orange sphere; adjacent lysine ε-amine nitrogens are shown as blue spheres. These nitrogen atoms are attached to the hydrogel and displaced during expansion. Thus, the measured positions of Atto647N and fluorescein correspond to the displaced coordinates of their respective anchoring nitrogens.
Inter-lysine distances were quantified by molecular dynamics.
(A) Distribution of distances (nm).
(B) Minimum distance (~0.44 nm), parallel side chains.
(C) Peak distance (~0.88 nm), staggered orientation.
(D) Larger distance (~1.41 nm), antiparallel orientation.

**Supplementary Figure 6. Molecular dynamics (MD) simulation of k-nearest-neighbor (kNN) distance distributions for the mCLING peptide followed by analysis under defined anchoring, cleavage, and labeling scenarios.**
Molecular dynamics (MD) simulations generated 1001 conformations of the mCLING peptide (see Methods: mCLING molecular dynamics simulations). All kNN distance distributions shown were computed across the full ensemble of 1001 conformations. Atomic model panels display the first conformation.
For each conformation, fluorophore positions were assigned to the atomic coordinates of gel-anchoring atoms defined in **Supplementary Figure 4**. Orange spheres denote Atto647N anchoring sites and blue spheres denote fluorescein anchoring sites. k-nearest-neighbor (kNN) distances were computed as Euclidean distances between anchoring-site atomic coordinates. In atomic model panels, arrows originate from a selected anchoring-site atom and point to its k-nearest neighboring sites; neighboring sites are numbered by increasing Euclidean distance, with labels 1–5 or 1–4 indicating the kNN rank for the representative

bioRxiv preprint doi: https://doi.org/10.64898/2026.05.31.729018; this version posted June 1, 2026. The copyright holder for this preprint (which was not certified by peer review) is the author/funder, who has granted bioRxiv a license to display the preprint in perpetuity. It is made available under a CC-BY 4.0 International license.

conformation. All quantitative distributions were computed using all 1001 conformations, with one distance per kNN rank per conformation.
Two terminal fluorescein labeling scenarios defined in **Supplementary Figure 4** were simulated: (A) terminal fluorescein assigned to a lysine side-chain amine (**Supplementary Fig. 4G.ii and 4H.ii**), and (B) terminal fluorescein assigned to the N-terminal amine (**Supplementary Fig. 4G.iii and 4H.iii**). In **Supplementary Figure 4**, panel G denotes configurations in which Atto647N is cleaved and removed, whereas panel H denotes configurations in which Atto647N is retained. (C) shows pooled distance distributions combining both terminal fluorescein labeling scenarios.

(A) Terminal fluorescein assigned to a lysine side-chain amine. (i) Representative atomic model illustrating the anchoring configuration corresponding to **Supplementary Fig. 4H.ii**, in which Atto647N is retained and the terminal fluorescein is assigned to a lysine side-chain amine. Arrows originate from the Atto647N anchoring-site atom and point to its k-nearest fluorescein neighbors (kNN ranks 1–5). (ii) Same model as (i), with arrows originating from each fluorescein anchoring-site atom in turn and pointing to its nearest fluorescein neighbors (kNN ranks 1–4). (iii) Representative atomic model for the corresponding Atto-cleaved configuration (**Supplementary Fig. 4G.ii**), in which Atto647N is removed and the newly generated N-terminus is labeled with fluorescein (kNN ranks 1–5). (iv) Atto647N→fluorescein kNN distance distributions (ranks 1–5) computed from the configuration in (i) across all 1001 conformations, with the Atto647N anchoring site queried against fluorescein-labeled sites (one distance per kNN rank per conformation). (v) Fluorescein→fluorescein kNN distance distributions (ranks 1–4) computed from the same anchoring configuration as in (iv), using only fluorescein-labeled sites. (vi) Fluorescein→fluorescein kNN distance distributions (ranks 1–5) computed from the same anchoring geometry as in (iv), after treating the Atto647N anchoring-site coordinate as a fluorescein site for analysis. This corresponds to the Atto-cleaved case in which the newly generated N-terminus is labeled with fluorescein while the anchoring atom remains unchanged, such that the underlying anchoring-site coordinates are identical. (vii–ix) Five-choose-four subsampling was used to reflect molecule-to-molecule variation in which anchoring and labeling sites are present after anchoring, proteolysis, and post-expansion labeling—including outcomes such as **Supplementary Fig. 4G.i and 4H.i**, in which both the N-terminal amine and a lysine side-chain amine are anchored. All subsets of four labeling-site coordinates were selected from the five available sites for each peptide conformation. (vii) Atto647N→fluorescein kNN distance distributions computed from five-choose-four subsampled fluorescein site sets in Atto-retained configurations. (viii) Fluorescein→fluorescein kNN distance distributions computed from the same five-choose-four subsampled fluorescein site sets as in (vii). (ix) Fluorescein→fluorescein kNN distance distributions computed using five-choose-four subsampling in Atto-cleaved configurations, with the former Atto647N anchoring-site coordinate treated as a fluorescein site for analysis.

(B) Terminal fluorescein assigned to the N-terminal amine. (i–ix) Same anchoring configurations, kNN definitions, and five-choose-four subsampling procedures described in (A)(i–ix), applied to configurations in which the terminal fluorescein is assigned to the N-terminal amine (**Supplementary Fig. 4G.iii and 4H.iii**).

(C) Combined distance distributions. (i) Combined Atto647N→fluorescein kNN distance distributions pooled across both terminal fluorescein labeling scenarios for configurations in which Atto647N is retained (**Supplementary Fig. 4H**). (ii) Combined fluorescein→fluorescein kNN distance distributions pooled across both terminal fluorescein labeling scenarios for configurations in which Atto647N is retained (**Supplementary Fig. 4H**). (iii) Combined fluorescein→fluorescein kNN distance distributions pooled across both terminal fluorescein labeling scenarios for configurations in which Atto647N is cleaved (**Supplementary Fig. 4G**).

**Supplementary Figure 7. 3D regions containing putative mCLING peptides after ~1000× expansion.**

bioRxiv preprint doi: https://doi.org/10.64898/2026.05.31.729018; this version posted June 1, 2026. The copyright holder for this preprint (which was not certified by peer review) is the author/funder, who has granted bioRxiv a license to display the preprint in perpetuity. It is made available under a CC-BY 4.0 International license.

Three-dimensional renderings of six regions selected to be representative with respect to signal brightness within a 10 × 20 μm field spanning the full z depth (4.4 μm) after ~1000× linear expansion. Boxed regions highlight examples of putative mCLING peptides, defined as kNN-derived clusters containing one Atto647N signal (magenta) and at least four proximal fluorescein signals (green). Boxes are shown for illustrative examples and do not mark all such clusters within the field. Because mCLING peptides contain a hydrophobic palmitoyl tail, intermolecular interactions may occur, and some boxed regions may contain more than one putative peptide due to spatial overlap; in cases of overlap, one box was randomly selected.

**Supplementary Figure 8. RMSD of coarse putative peptides before nearest-neighbor filtering.**
From 27,426 Atto647N localizations (three gels, eight regions), fluorescein signals were associated with the nearest Atto647N, yielding 10,696 coarse putative peptides (see Methods, Identification of putative peptides). Each contains one Atto647N and four or five fluoresceins; assignments may include signals from neighboring peptides.
For each coarse putative peptide, observed coordinates were aligned to a 1,001-conformation molecular-dynamics ensemble spanning anchoring, digestion, and labeling outcomes (**Supplementary Figs. 4, 6**), and RMSD was computed. Boxplots show the median, IQR, whiskers extending to the farthest data point within 1.5×IQR of each box edge, and outliers. (A) All gels. (B) Individual gels. (C) Individual regions.

**Supplementary Figure 9. RMSD of putative peptides after nearest-neighbor filtering.**
Nearest-neighbor filtering (see Methods; filtered subset of **Supplementary Fig. 8**; same peptide set as **Fig. 3C**) yields the final putative peptides. RMSD was computed by alignment to the molecular-dynamics ensemble and shown as boxplots.
(A) All gels. (B) By gel. (C) By region.
Summary statistics: Table S13 (A), Table S14 (B), Table S15 (C).

**Supplementary Figure 10. Per-axis RMSD of putative peptides (same peptide set as Fig. 3C).**
Per-axis RMSD (x, y, z) was computed using the same analysis as Supplementary Fig. 9. Fig. 3C shows pooled data; here the same data are shown pooled, by gel, and by region to assess variation.
Summary statistics: Table S13 (A), Table S14 (B), Table S15 (C).

**Supplementary Figure 11. Single-molecule expansion factors (same peptide set as Fig. 3C).**
An isotropic expansion factor was estimated for each peptide by minimizing RMSD to the molecular-dynamics ensemble, using the same analysis as Supplementary Fig. 9. Fig. 3C shows pooled data; here the same data are shown pooled, by gel, and by region to assess variation.
(A) All gels combined. (B) By gel. (C) By region.
Summary statistics: Table S13 (A), Table S14 (B), Table S15 (C).

**Supplementary Figure 12. RMSD histograms (same peptide set and RMSD values as Fig. 3C).**
The same RMSD values are replotted as histograms with interquartile ranges; only the visualization differs. Fig. 3C shows pooled data; here the same data are shown pooled, by gel, and by region to assess variation.
(A) All data combined. (B) By gel. (C) By region.
Summary statistics: Table S13 (A), Table S14 (B), Table S15 (C).

**Supplementary Figure 13. Robustness of RMSD to MD ensemble size.**
Using the filtered putative peptides defined in Supplementary Fig. 9, total RMSD was recomputed after alignment to MD reference ensembles containing 10–1,001 conformations. For each ensemble size, RMSD

bioRxiv preprint doi: https://doi.org/10.64898/2026.05.31.729018; this version posted June 1, 2026. The copyright holder for this preprint (which was not certified by peer review) is the author/funder, who has granted bioRxiv a license to display the preprint in perpetuity. It is made available under a CC-BY 4.0 International license.

distributions are summarized as boxplots (median, IQR, whiskers to farthest data points within 1.5×IQR of box edges, outliers).
Summary statistics are reported in **Table S16**.

**Supplementary Figure 14. RMSD histograms across MD ensemble sizes.**
For the same peptides and RMSD values analyzed in Supplementary Fig. 13, distributions are replotted as histograms to visualize distribution shape as a function of MD ensemble size. Panels correspond to MD ensemble sizes: (A) 10; (B) 100; (C) 200; (D) 300; (E) 400; (F) 500; (G) 600; (H) 700; (I) 800; (J) 900; (K) 1,001 conformations.
Summary statistics are reported in **Table S16.**

**Supplementary Figure 15. Peptide overlays below the IQR of the Fig. 3C.i RMSD distribution.**
Putative peptides with total RMSD below the lower quartile of **Fig. 3C.i** are shown. Fluorophore coordinates are overlaid after optimal alignment to the molecular-dynamics ensemble (**Supplementary Fig. 6**). Peptide length reflects anchoring, cleavage, and labeling outcomes (**Supplementary Fig. 4**), yielding four or five fluorescein sites.
(A) 1 Atto647N followed by 5 fluoresceins.
(B) 1 Atto647N followed by 4 fluoresceins.

**Supplementary Figure 16. Peptide overlays within the IQR of the Fig. 3C.i RMSD distribution.**
Same analysis and representation as Supplementary Fig. 15, for peptides with RMSD within the interquartile range of Fig. 3C.i.

**Supplementary Figure 17. Peptide overlays above the IQR of the Fig. 3C.i RMSD distribution.**
Same analysis and representation as Supplementary Fig. 15, for peptides with RMSD above the upper quartile of Fig. 3C.i.

**Supplementary Figure 18. Emitter detection using Gaussian correlation to identify true fluorescent emitters.**
Confocal images were acquired as described in Methods (confocal z-stack acquisition). All images shown are raw, with no background subtraction or additional processing. Candidate fluorescent emitters were filtered by intensity and size and evaluated by Gaussian correlation (Methods, 3D emitter detection), using a correlation coefficient >0.70 as the acceptance threshold.
Each subpanel (A.i–A.iii, B.i–B.iii) shows three representative emitters. For each emitter, a central z-slice image is displayed with the emitter center marked by a cross. From this center, one diagonal line scan (upper left to lower right) is shown, along with 3 additional line scans: upper right to lower left, vertical (top to bottom), and horizontal (left to right). Intensity profiles from these 4 line scans are plotted, and their average is shown as a bold trace. In the line-scan plots, the x-axis denotes distance along the line scan and the y-axis denotes fluorescence intensity (arbitrary units).
(A) Atto647N emitters.
(A.i) Accepted emitters with high Gaussian correlation (≈0.9), drawn from 27,426 Atto647N emitters across three gels and eight regions.
(A.ii) Accepted emitters near the Gaussian-correlation threshold (≈0.7), drawn from the same 27,426 Atto647N emitters.
(A.iii) Rejected emitters with Gaussian correlation <0.7, drawn from the 21,705 emitters that failed the Gaussian-correlation threshold after intensity and size filtering (49,131 total candidates across three gels and eight regions).

bioRxiv preprint doi: https://doi.org/10.64898/2026.05.31.729018; this version posted June 1, 2026. The copyright holder for this preprint (which was not certified by peer review) is the author/funder, who has granted bioRxiv a license to display the preprint in perpetuity. It is made available under a CC-BY 4.0 International license.

(B) Fluorescein emitters.
(B.i) Accepted emitters with high Gaussian correlation (≈0.9), drawn from 125,395 fluorescein emitters across three gels and eight regions.
(B.ii) Accepted emitters near the Gaussian-correlation threshold (≈0.7), drawn from the same 125,395 fluorescein emitters.
(B.iii) Rejected emitters with Gaussian correlation <0.7, drawn from the 462,408 emitters that failed the Gaussian-correlation threshold after intensity and size filtering (587,803 total candidates across three gels and eight regions).

**Supplementary Figure 19. k-nearest-neighbor (kNN) 1–5 distance distributions for Atto647N–fluorescein (magenta) and fluorescein–fluorescein (green).**
Histograms use 0.05 μm bins with Gaussian smoothing (σ = 1 bin). Peaks are defined as the maxima of the smoothed distributions. n denotes the number of distances per kNN rank. Summary distance statistics corresponding to each panel are reported in **Tables S5–S8**.
A) All gels combined (3 gels, 8 regions): Atto647N–fluorescein, n = 27,426; fluorescein–fluorescein, n = 125,395.
(See pooled distance statistics in **Table S5**.)
B) Partitioned by four-round gelation experiment (biological replicates); each ~1000× gel was generated by an independent four-network casting sequence, initiated from an independently cast first-round ~18× gel.
B.i) Gel 1 (2 regions): Atto647N–fluorescein, n = 9,266; fluorescein–fluorescein, n = 63,733 (statistics in **Table S6**).
B.ii) Gel 2 (4 regions): Atto647N–fluorescein, n = 11,671; fluorescein–fluorescein, n = 32,765 (statistics in **Table S7**).
B.iii) Gel 3 (2 regions): Atto647N–fluorescein, n = 6,489; fluorescein–fluorescein, n = 28,897 (statistics in **Table S8**).

**Supplementary Figure 20. Randomization analysis of k-nearest-neighbor (kNN) distance distributions, all gels.**
Each region is a three-dimensional imaging volume containing Atto647N and fluorescein emitters, each represented by an (x, y, z) coordinate. For the randomized control, emitter counts were preserved and all coordinates were randomly chosen within the same volume. kNN distance distributions were recomputed from the randomized coordinates. Histograms use 0.05 μm bins with Gaussian smoothing (σ = 1 bin). Peak positions are defined as the bin center corresponding to the global maximum of the smoothed distribution. Distances are aggregated across eight regions from three gels. Observed Atto647N–fluorescein distributions are shown in magenta with randomized controls in gray (n = 27,426 distances per kNN rank). Observed fluorescein–fluorescein distributions are shown in green with randomized controls in gray (n = 125,395 distances per kNN rank). Panels A–E show kNN ranks 1–5. Panel F shows robustness across 100 randomizations assessed using two-sample Kolmogorov–Smirnov tests (***, $p < 0.001$); KS D statistics report effect size. Corresponding Kolmogorov–Smirnov D statistics by kNN rank (1–5) are reported in **Table S9**.

Supplementary Figures 21–23 show the same randomization test as Supplementary Figure 20, with distances aggregated at the level of individual gels (statistics in Tables **S10-12**).

**Supplementary Figure 21. Randomization analysis of k-nearest-neighbor (kNN) distance distributions for gel 1 (statistics in Table S10).**

**Supplementary Figure 22. Randomization analysis of k-nearest-neighbor (kNN) distance distributions for gel 2 (statistics in Table S11).**

bioRxiv preprint doi: https://doi.org/10.64898/2026.05.31.729018; this version posted June 1, 2026. The copyright holder for this preprint (which was not certified by peer review) is the author/funder, who has granted bioRxiv a license to display the preprint in perpetuity. It is made available under a CC-BY 4.0 International license.

**Supplementary Figure 23. Randomization analysis of k-nearest-neighbor (kNN) distance distributions for gel 3 (statistics in Table S12).**

**Supplementary Figure 24. Preprocessing and transformer-based feature extraction pipeline for microscopy images.** Raw fluorescence microscopy images are first partitioned into fixed-size patches. Each patch is flattened into a one-dimensional vector and supplemented with positional encodings to retain spatial information. The resulting sequence of embedded patches is processed by a transformer encoder, which integrates local texture features with global spatial relationships. The output is a structured feature map that serves as input for downstream graph-based modeling.

**Supplementary Figure 25. Graph formulation from transformer-derived patch features.** Each node corresponds to a transformer-derived patch feature. Pairwise similarities between feature vectors define edge weights, forming a weighted, undirected graph G(V, E). Edges are retained only when their similarity exceeds a predefined threshold. The resulting symmetric adjacency matrix A(i, j) encodes structural relationships across the image and enables graph-based segmentation and clustering.

**Supplementary Figure 26**. **Unsupervised graph-based segmentation framework integrating Normalized Cut and correlation clustering losses.** Node features are iteratively updated through aggregation, propagation, and normalization steps to refine graph embeddings. The training objective combines the Normalized Cut (NCut) loss and a correlation clustering loss to promote coherent partitioning. A global max pooling layer aggregates node-wise information, followed by a fully connected layer and softmax classifier that assign each node to foreground or background. The combined loss enforces consistent separation of structurally similar regions within the graph.

**Supplementary Figure 27**. **Post-segmentation analysis for separation of heterogeneous foreground objects.** Following foreground–background separation, connected components are extracted from the primary mask. Objects are filtered using geometric and intensity-based descriptors, including eccentricity, aspect ratio, extent, and intensity criteria. Partitioning around medoids clustering is subsequently applied to group objects of similar identity. Decision boundaries delineate the resulting clusters, and bounding boxes indicate individual, physically plausible particle instances.

**Supplementary Figure 28**. **End-to-end *ab initio* 3D reconstruction pipeline from preprocessed 2D images.** The workflow begins with segmented images and preprocessing steps including intensity normalization, background suppression, Gaussian smoothing, and total variation–based enrichment. Preprocessed inputs are mapped into a Fourier domain representation and encoded using a convolutional variational autoencoder (VAE). The encoder performs amortized inference to learn latent variables under a combined objective incorporating KL divergence and symmetry loss, followed by affine transformations in the decoder to generate consistent reconstructions. Reconstructed 2D slices are stacked and integrated in the spatial domain to produce a volumetric density map V(u, v, w), yielding an ab initio 3D molecular model (**Fig. 4**).

**Supplementary Figure 29. Additional GFP examples at different expansion factors.** Representative 2D ONE GFP images are shown at (A) 50-fold expansion and (B) 100-fold expansion.

**Supplementary Figure 30. GFP imaged with ONE microscopy across expansion factors.**
Representative GFP images at (A) 18-fold, (B) 50-fold, and (C) 100-fold linear expansion. Each panel shows a field-of-view overview and a corresponding magnified region from the same image.

bioRxiv preprint doi: https://doi.org/10.64898/2026.05.31.729018; this version posted June 1, 2026. The copyright holder for this preprint (which was not certified by peer review) is the author/funder, who has granted bioRxiv a license to display the preprint in perpetuity. It is made available under a CC-BY 4.0 International license.

**Supplementary Figure 31. Four-network gels generated using a modified 1000ExM process.** In a modified 1000ExM process, each sequential casting round includes two incubations in activated monomer solution before polymerization. The second incubation replaces the monomer-depleted and/or diluted first solution with fresh activated monomer solution, for further infiltration.
(A) Schematic showing how per-round shrinkage factors, per-round expansion factors, and cumulative linear expansion factors were calculated during four-network gel formation. After the first gel was expanded, each subsequent casting round included incubation in activated monomer solution (two rounds), polymerization of the next network, measurement of the fully gelled composite, and re-expansion in water. Per-round shrinkage factors were measured after full gelation and calculated as gel length after polymerization divided by gel length before monomer solution addition and polymerization (i.e., at the end of the last expansion round). Per-round expansion factors were calculated as gel length after water expansion divided by gel length before water expansion. Cumulative linear expansion factors were calculated by multiplying the sequential shrinkage and expansion factors through all rounds up to the current round.
(B) Per-round shrinkage factors for rounds 2–4. Boxplots (throughout this figure) show the median (middle line), interquartile range (top and bottom of box), whiskers extending to the farthest data point within 1.5×IQR of each box edge, and individual gel measurements as open circles.
(C) Per-round expansion factors for rounds 1–4. Note that some circles have similar or identical values - this is not an error, and we include all the raw data in Table S18.
(D) Cumulative linear expansion factors after each expansion round, with round 4 gels reaching up to 2534× linear expansion.
(E) Images of fully expanded round 4 gels.
(F) Images of round 4 gels (made at the same time) maintained at room temperature on a dry surface for 98 hours (chosen arbitrarily). Gel linear size and height remained roughly unchanged over this period, with linear measurements showing a 0.3±0.5% change in expansion factor.

bioRxiv preprint doi: https://doi.org/10.64898/2026.05.31.729018; this version posted June 1, 2026. The copyright holder for this preprint (which was not certified by peer review) is the author/funder, who has granted bioRxiv a license to display the preprint in perpetuity. It is made available under a CC-BY 4.0 International license.

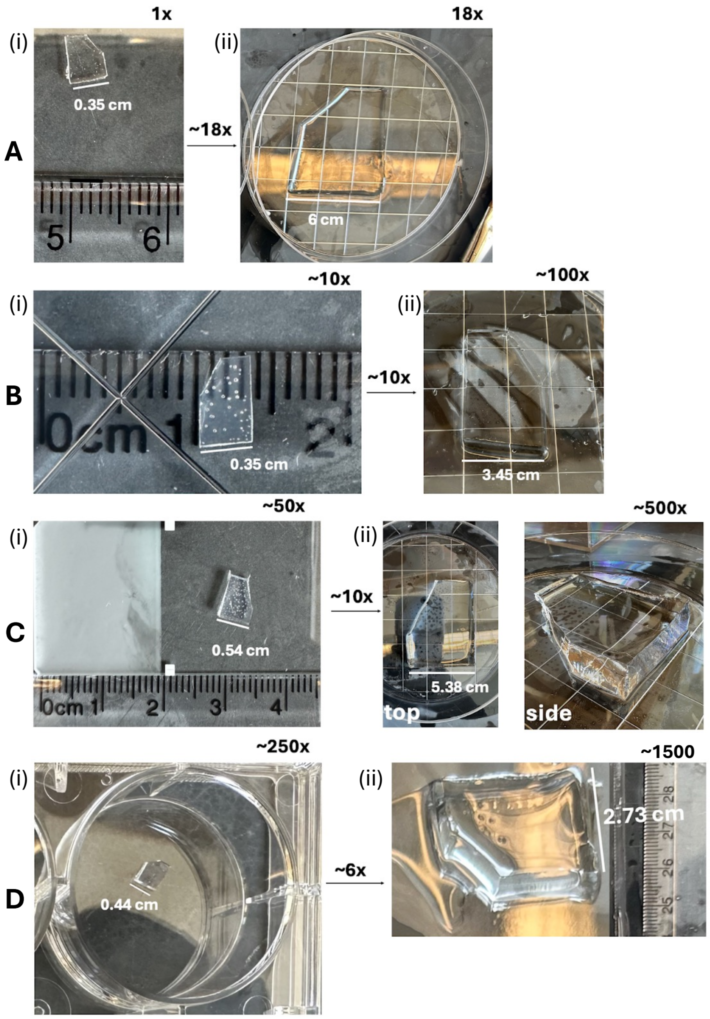


**Supplementary Figure 1. Representative hydrogels at successive expansion stages.**

bioRxiv preprint doi: https://doi.org/10.64898/2026.05.31.729018; this version posted June 1, 2026. The copyright holder for this preprint (which was not certified by peer review) is the author/funder, who has granted bioRxiv a license to display the preprint in perpetuity. It is made available under a CC-BY 4.0 International license.

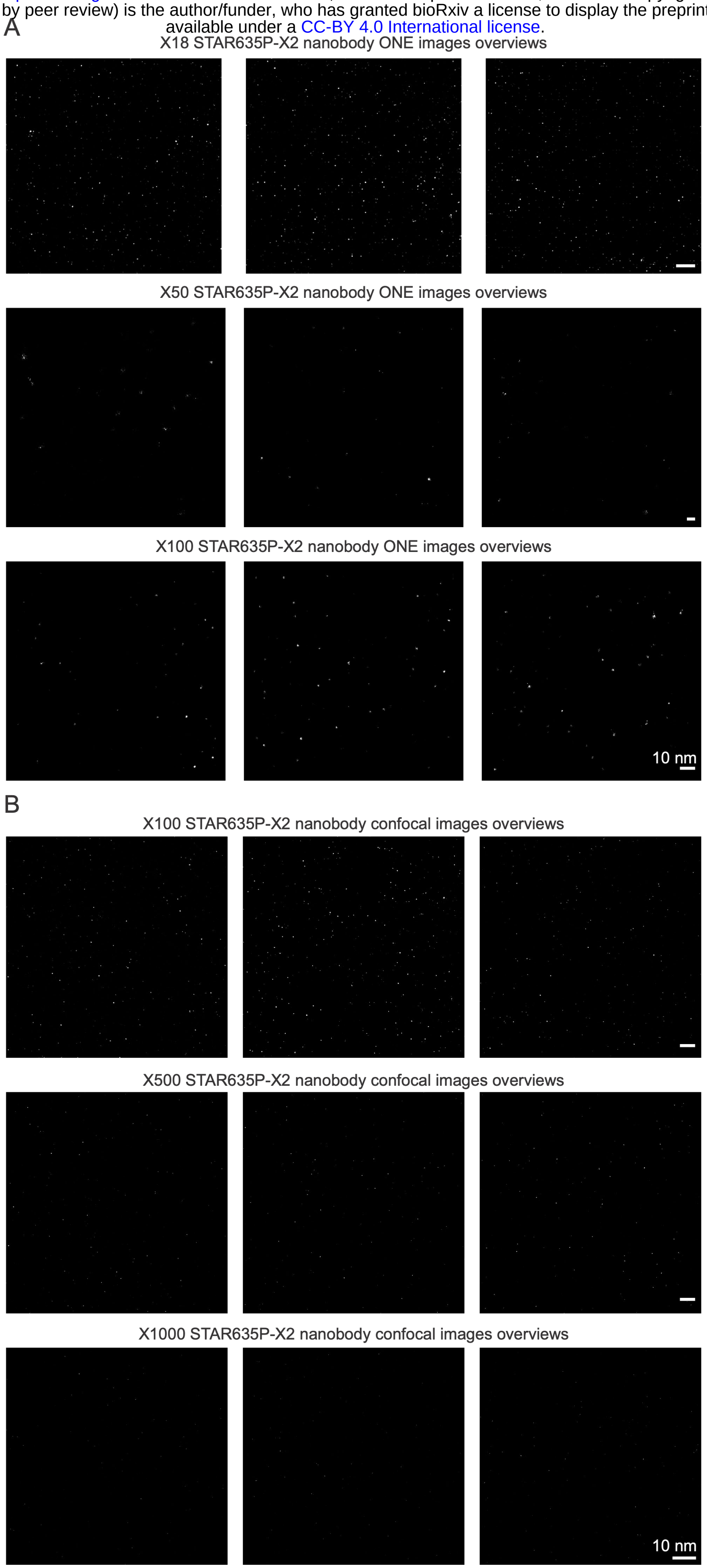


**Supplementary Figure 2. Nanobody imaging across different linear expansion factors.**

bioRxiv preprint doi: https://doi.org/10.64898/2026.05.31.729018; this version posted June 1, 2026. The copyright holder for this preprint (which was not certified by peer review) is the author/funder, who has granted bioRxiv a license to display the preprint in perpetuity. It is made available under a CC-BY 4.0 International license.

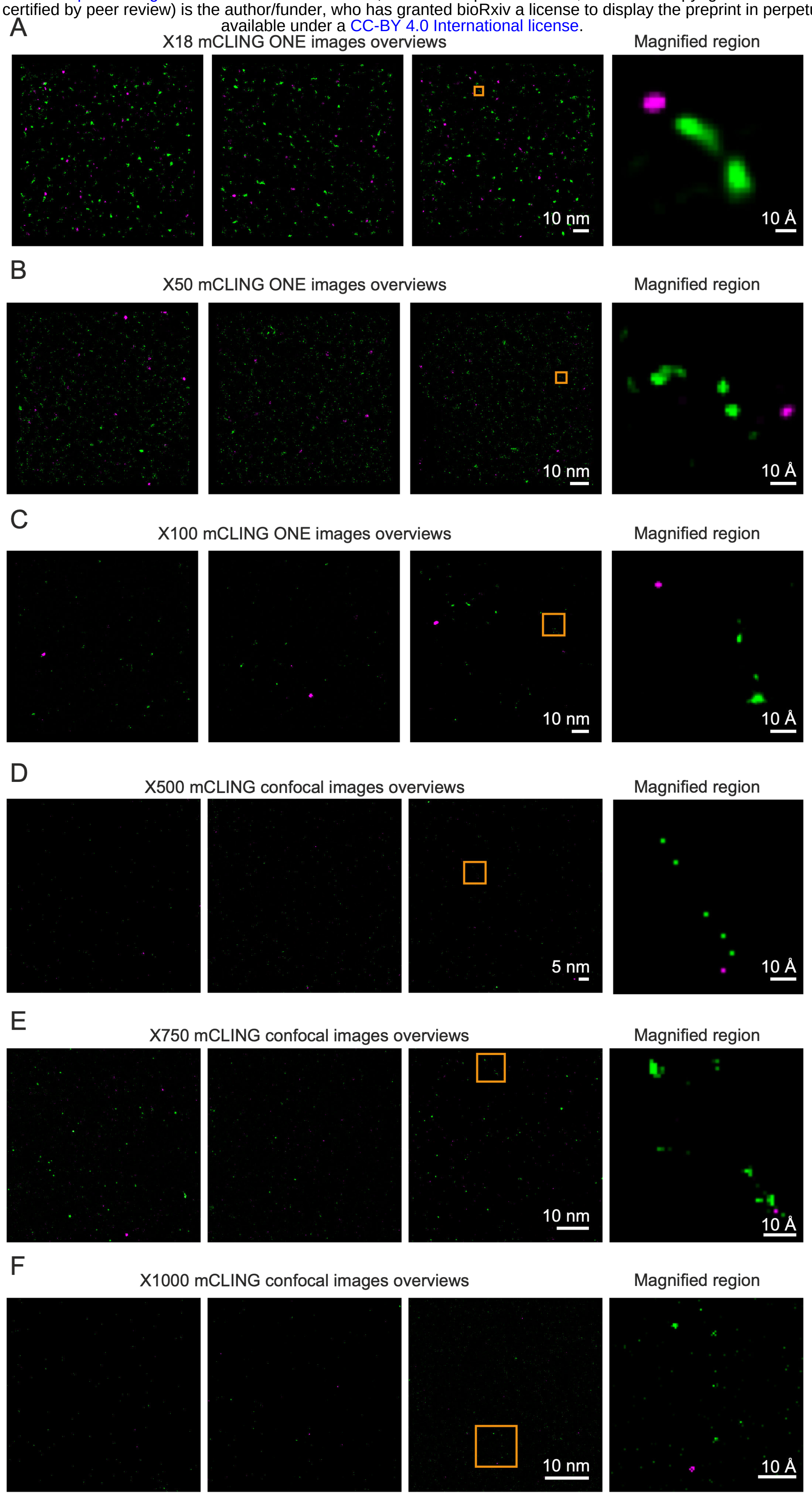


**Supplementary Figure 3. mCLING peptide imaging across different linear expansion factors.**

bioRxiv preprint doi: https://doi.org/10.64898/2026.05.31.729018; this version posted June 1, 2026. The copyright holder for this preprint (which was not certified by peer review) is the author/funder, who has granted bioRxiv a license to display the preprint in perpetuity. It is made available under a CC-BY 4.0 International license.

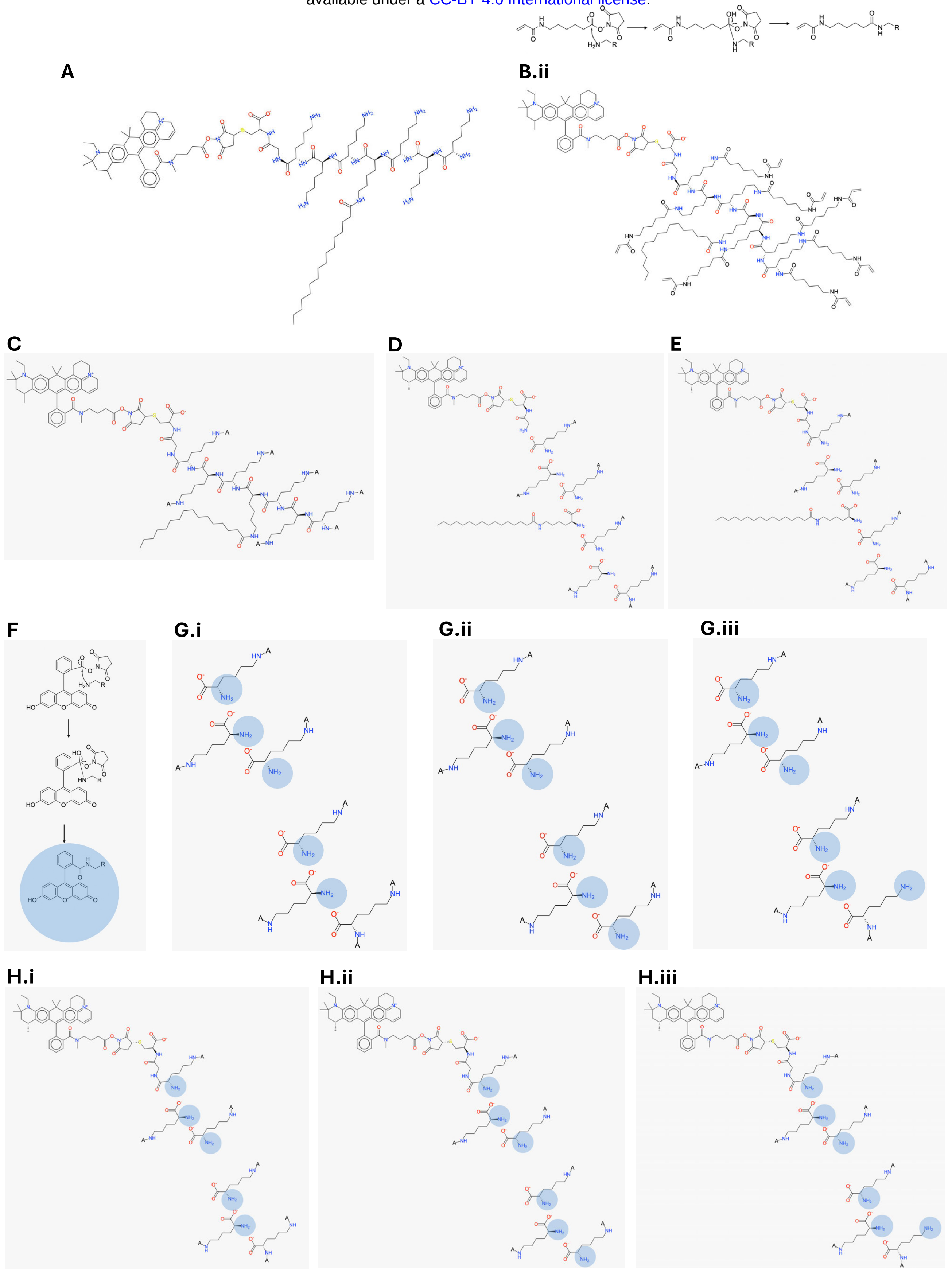


**Supplementary Figure 4. Pan-lysine anchoring, proteolytic cleavage, and post-expansion labeling chemistry illustrated using the mCLING peptide.**

bioRxiv preprint doi: https://doi.org/10.64898/2026.05.31.729018; this version posted June 1, 2026. The copyright holder for this preprint (which was not certified by peer review) is the author/funder, who has granted bioRxiv a license to display the preprint in perpetuity. It is made available under a CC-BY 4.0 International license.

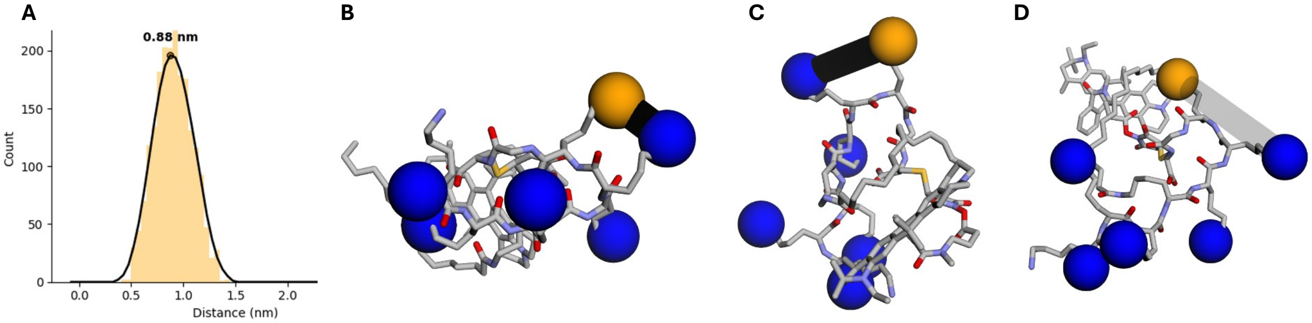


**Supplementary Figure 5. Distance distribution between adjacent lysine side chains.**

bioRxiv preprint doi: https://doi.org/10.64898/2026.05.31.729018; this version posted June 1, 2026. The copyright holder for this preprint (which was not certified by peer review) is the author/funder, who has granted bioRxiv a license to display the preprint in perpetuity. It is made available under a CC-BY 4.0 International license.

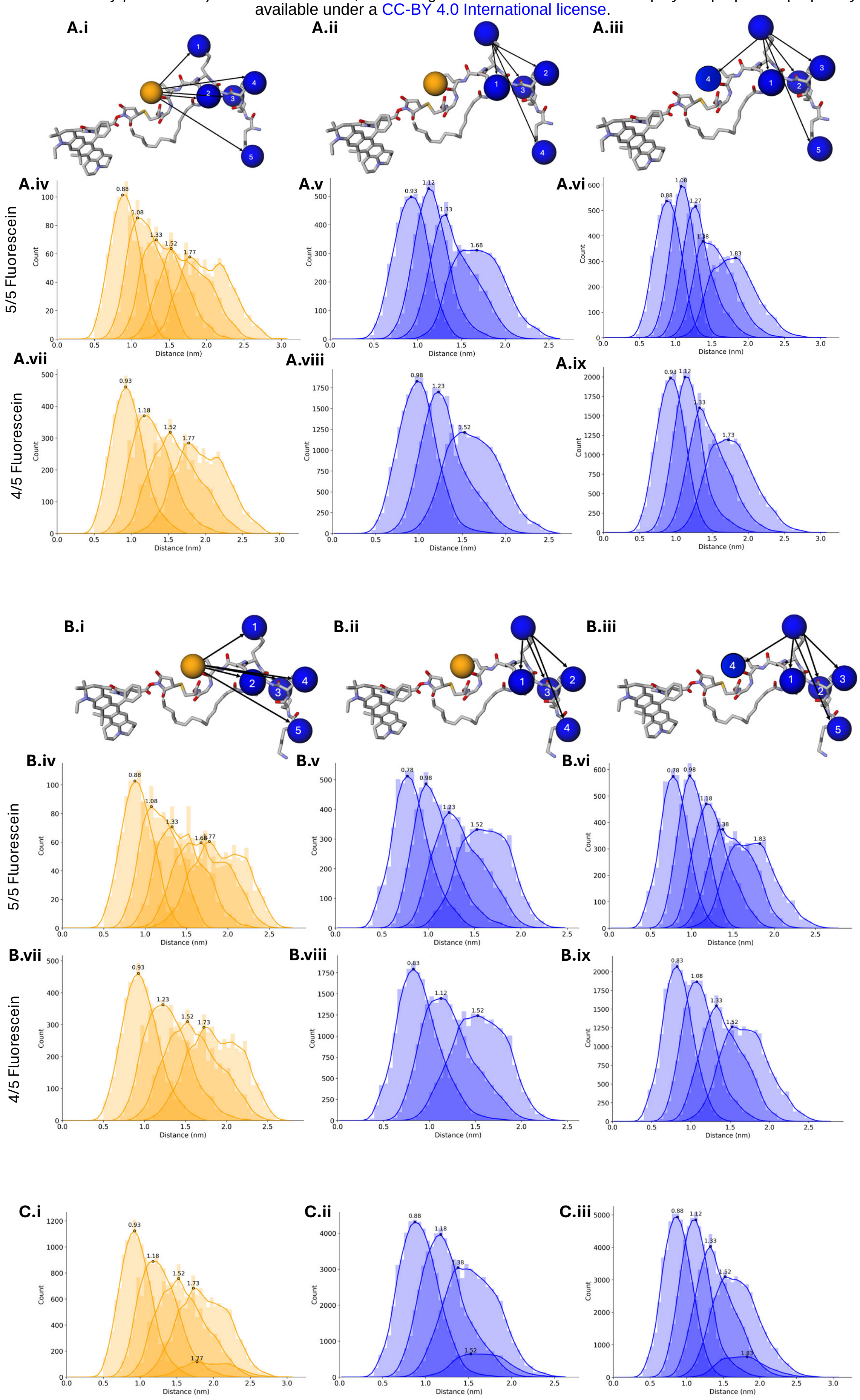


**Supplementary Figure 6. Molecular dynamics (MD) simulation of k-nearest-neighbor (kNN) distance distributions for the mCLING peptide under defined anchoring, cleavage, and labeling scenarios.**

bioRxiv preprint doi: https://doi.org/10.64898/2026.05.31.729018; this version posted June 1, 2026. The copyright holder for this preprint (which was not certified by peer review) is the author/funder, who has granted bioRxiv a license to display the preprint in perpetuity. It is made available under a CC-BY 4.0 International license.

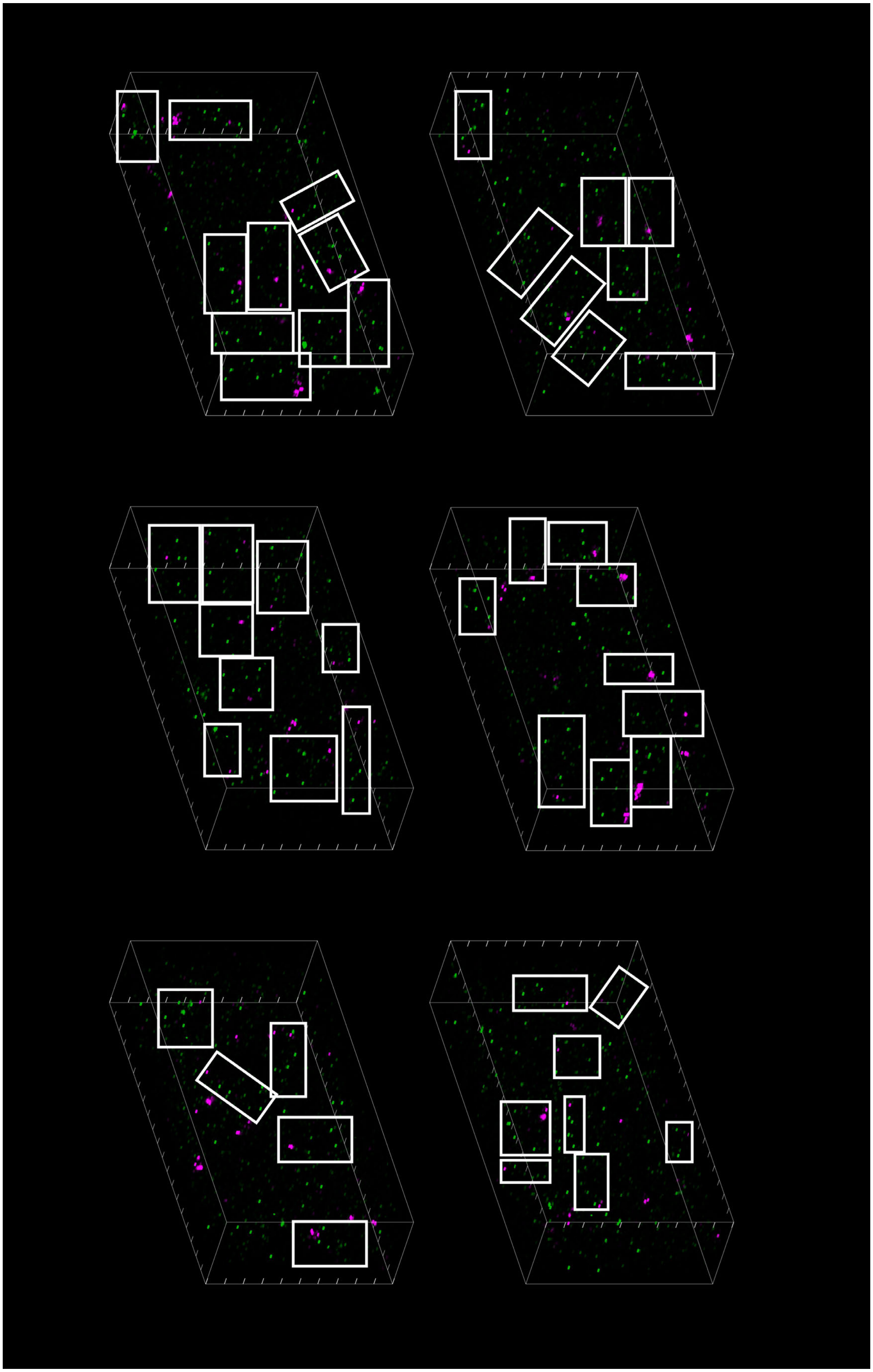

**Supplementary Figure 7. 3D regions containing putative mCLING peptides after ~1000× expansion.**

bioRxiv preprint doi: https://doi.org/10.64898/2026.05.31.729018; this version posted June 1, 2026. The copyright holder for this preprint (which was not certified by peer review) is the author/funder, who has granted bioRxiv a license to display the preprint in perpetuity. It is made available under a CC-BY 4.0 International license.

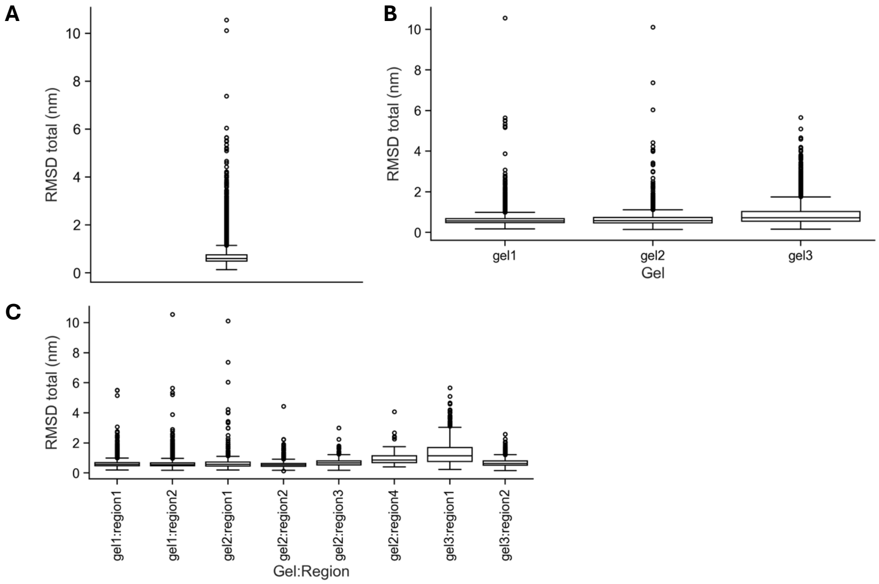


**Supplementary Figure 8. RMSD analysis of coarse putative peptides before nearest-neighbor filtering.**

bioRxiv preprint doi: https://doi.org/10.64898/2026.05.31.729018; this version posted June 1, 2026. The copyright holder for this preprint (which was not certified by peer review) is the author/funder, who has granted bioRxiv a license to display the preprint in perpetuity. It is made available under a CC-BY 4.0 International license.

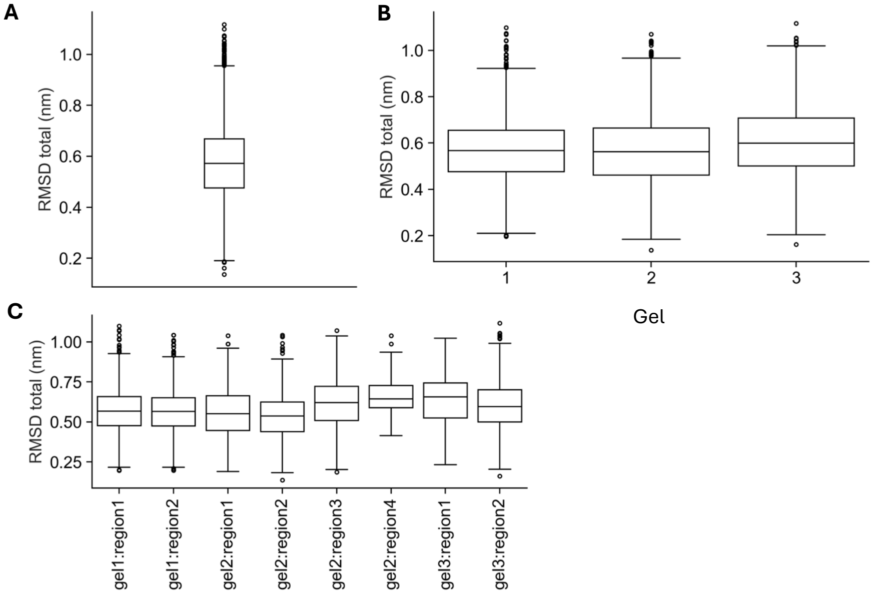


**Supplementary Figure 9. RMSD analysis of putative peptides after nearest-neighbor filtering.**

bioRxiv preprint doi: https://doi.org/10.64898/2026.05.31.729018; this version posted June 1, 2026. The copyright holder for this preprint (which was not certified by peer review) is the author/funder, who has granted bioRxiv a license to display the preprint in perpetuity. It is made available under a CC-BY 4.0 International license.

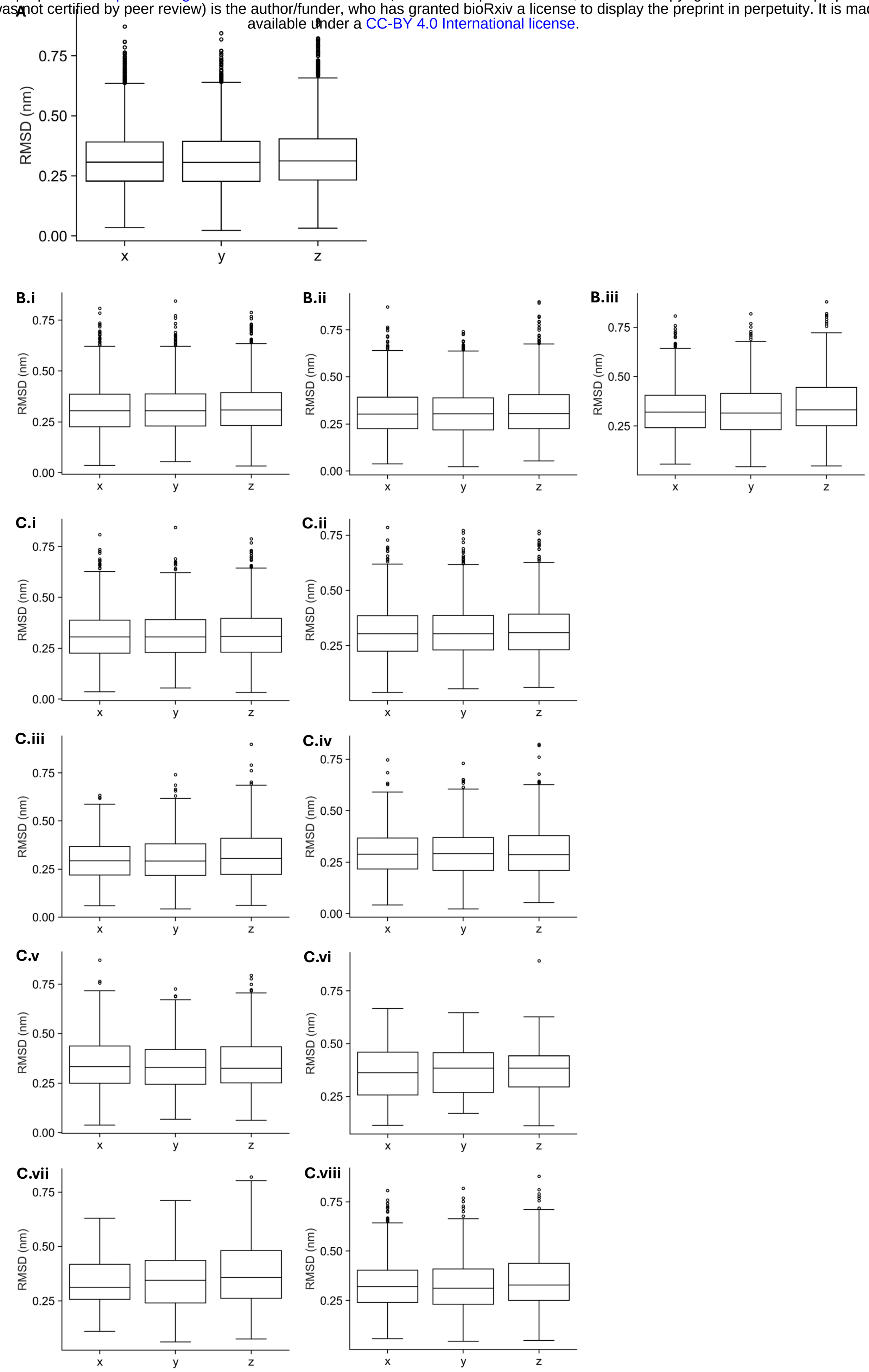


**Supplementary Figure 10. Per-axis RMSD of putative peptides (same peptide set as Fig. 3C).**

bioRxiv preprint doi: https://doi.org/10.64898/2026.05.31.729018; this version posted June 1, 2026. The copyright holder for this preprint (which was not certified by peer review) is the author/funder, who has granted bioRxiv a license to display the preprint in perpetuity. It is made available under a CC-BY 4.0 International license.

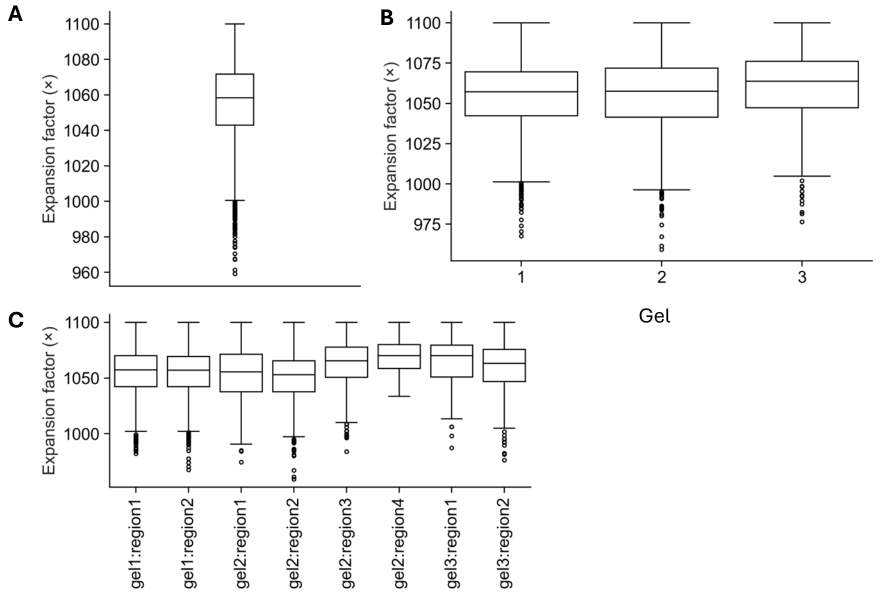


**Supplementary Figure 11. Single-molecule expansion factors (same peptide set as Fig. 3C).**

bioRxiv preprint doi: https://doi.org/10.64898/2026.05.31.729018; this version posted June 1, 2026. The copyright holder for this preprint (which was not certified by peer review) is the author/funder, who has granted bioRxiv a license to display the preprint in perpetuity. It is made available under a CC-BY 4.0 International license.

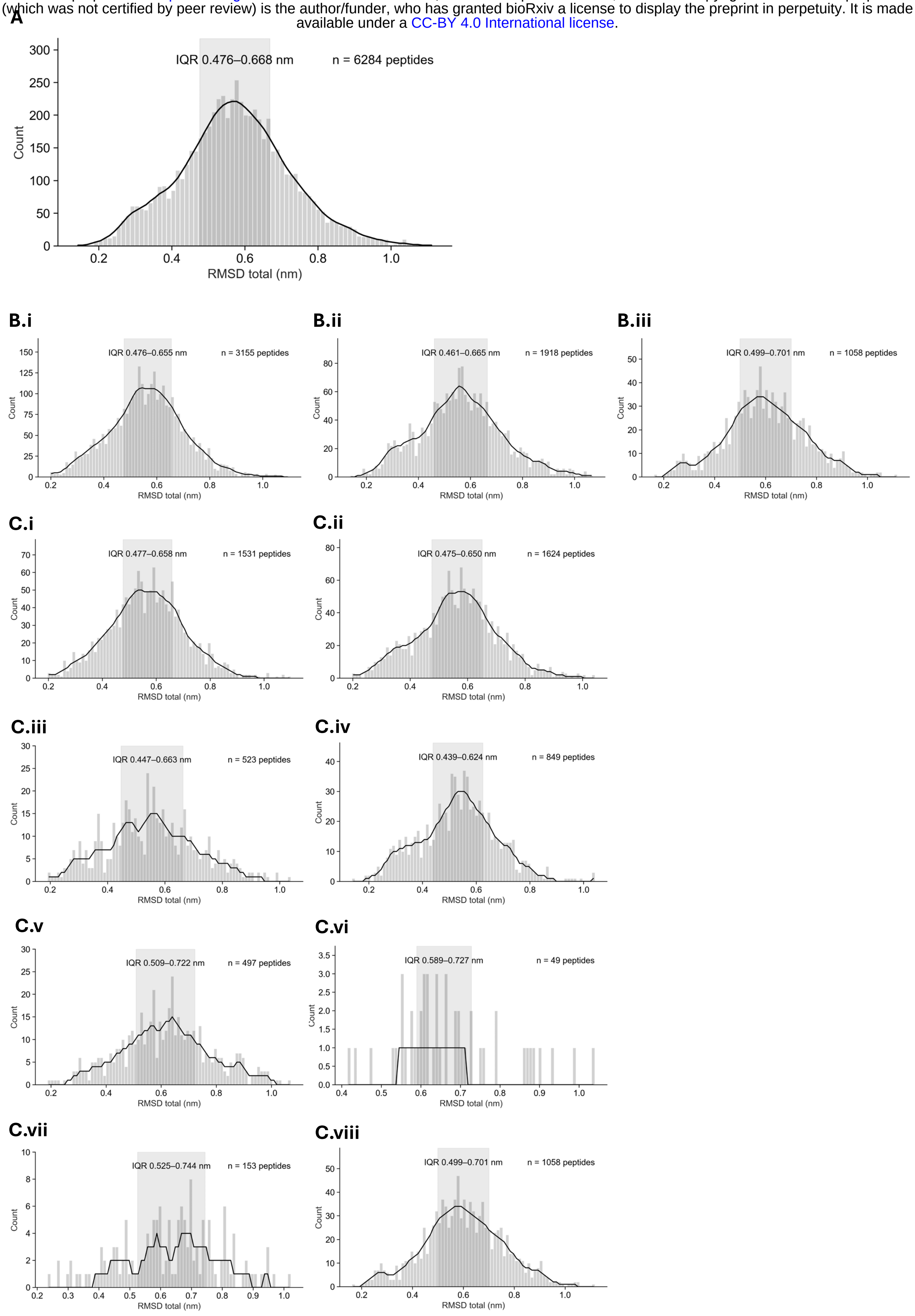


**Supplementary Figure 12. RMSD histograms (same peptide set and RMSD values as Fig. 3C).**

bioRxiv preprint doi: https://doi.org/10.64898/2026.05.31.729018; this version posted June 1, 2026. The copyright holder for this preprint (which was not certified by peer review) is the author/funder, who has granted bioRxiv a license to display the preprint in perpetuity. It is made available under a CC-BY 4.0 International license.

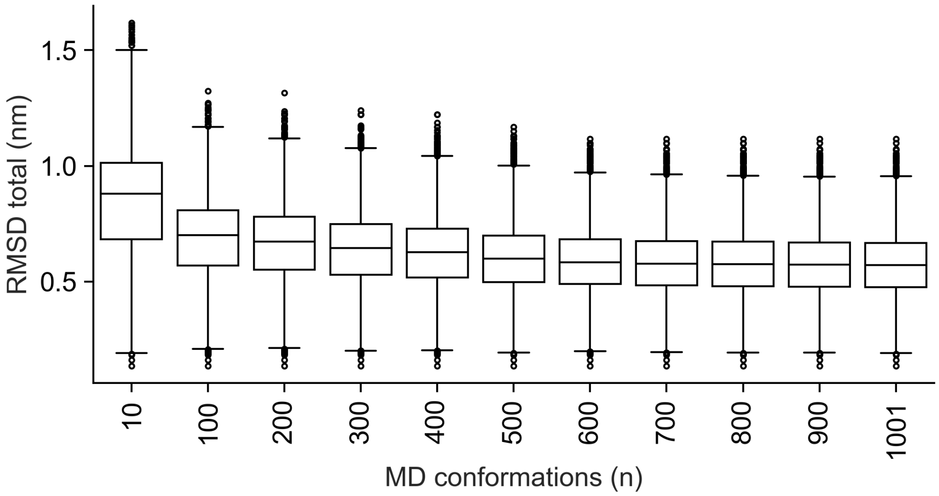


**Supplementary Figure 13. Robustness of RMSD to MD ensemble size.**

bioRxiv preprint doi: https://doi.org/10.64898/2026.05.31.729018; this version posted June 1, 2026. The copyright holder for this preprint (which was not certified by peer review) is the author/funder, who has granted bioRxiv a license to display the preprint in perpetuity. It is made available under a CC-BY 4.0 International license.

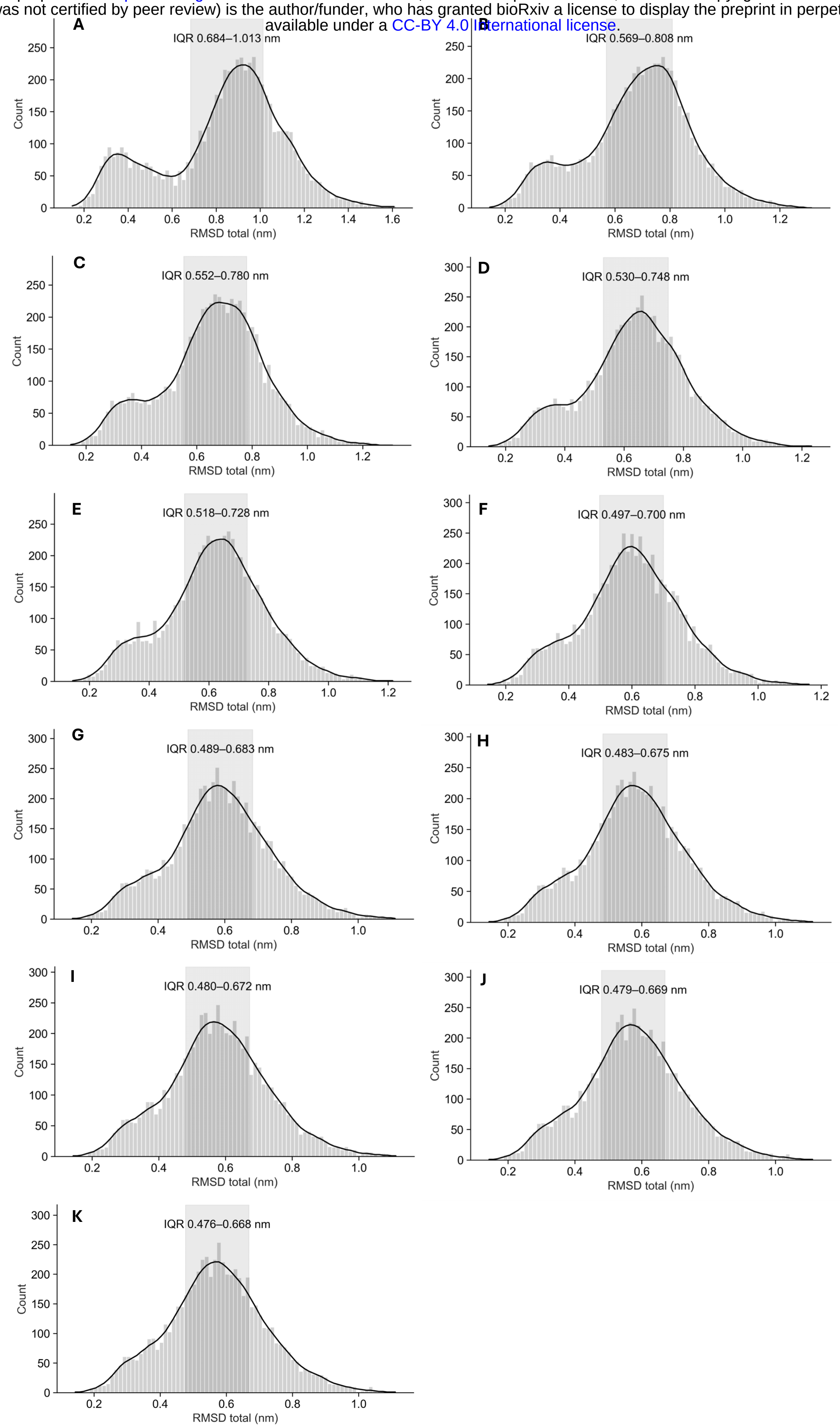


**Supplementary Figure 14. RMSD histograms across MD ensemble sizes.**

bioRxiv preprint doi: https://doi.org/10.64898/2026.05.31.729018; this version posted June 1, 2026. The copyright holder for this preprint (which was not certified by peer review) is the author/funder, who has granted bioRxiv a license to display the preprint in perpetuity. It is made available under a CC-BY 4.0 International license.

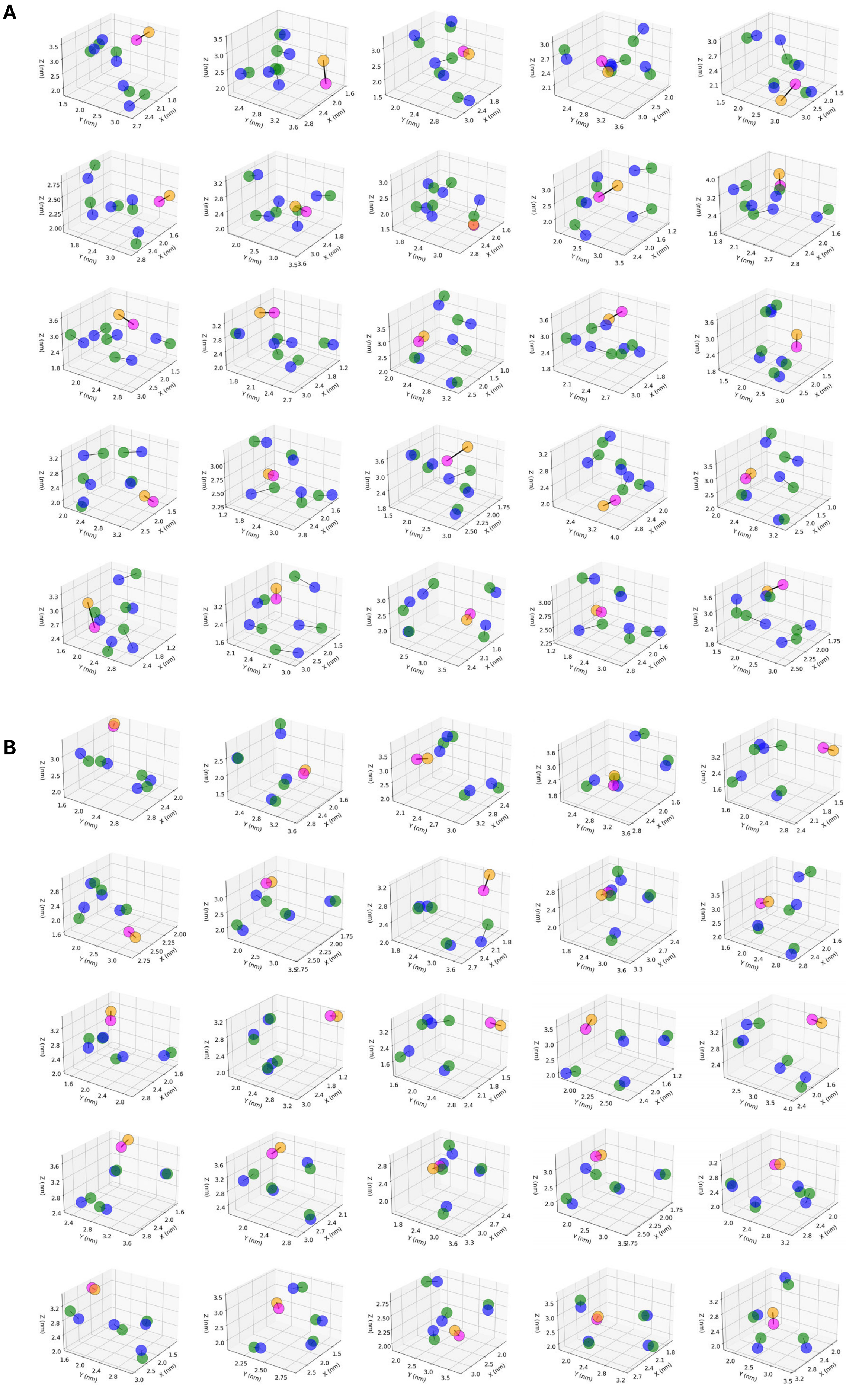


**Supplementary Figure 15. Peptide overlays below the IQR of the Fig. 3C.i RMSD distribution.**

bioRxiv preprint doi: https://doi.org/10.64898/2026.05.31.729018; this version posted June 1, 2026. The copyright holder for this preprint (which was not certified by peer review) is the author/funder, who has granted bioRxiv a license to display the preprint in perpetuity. It is made available under a CC-BY 4.0 International license.

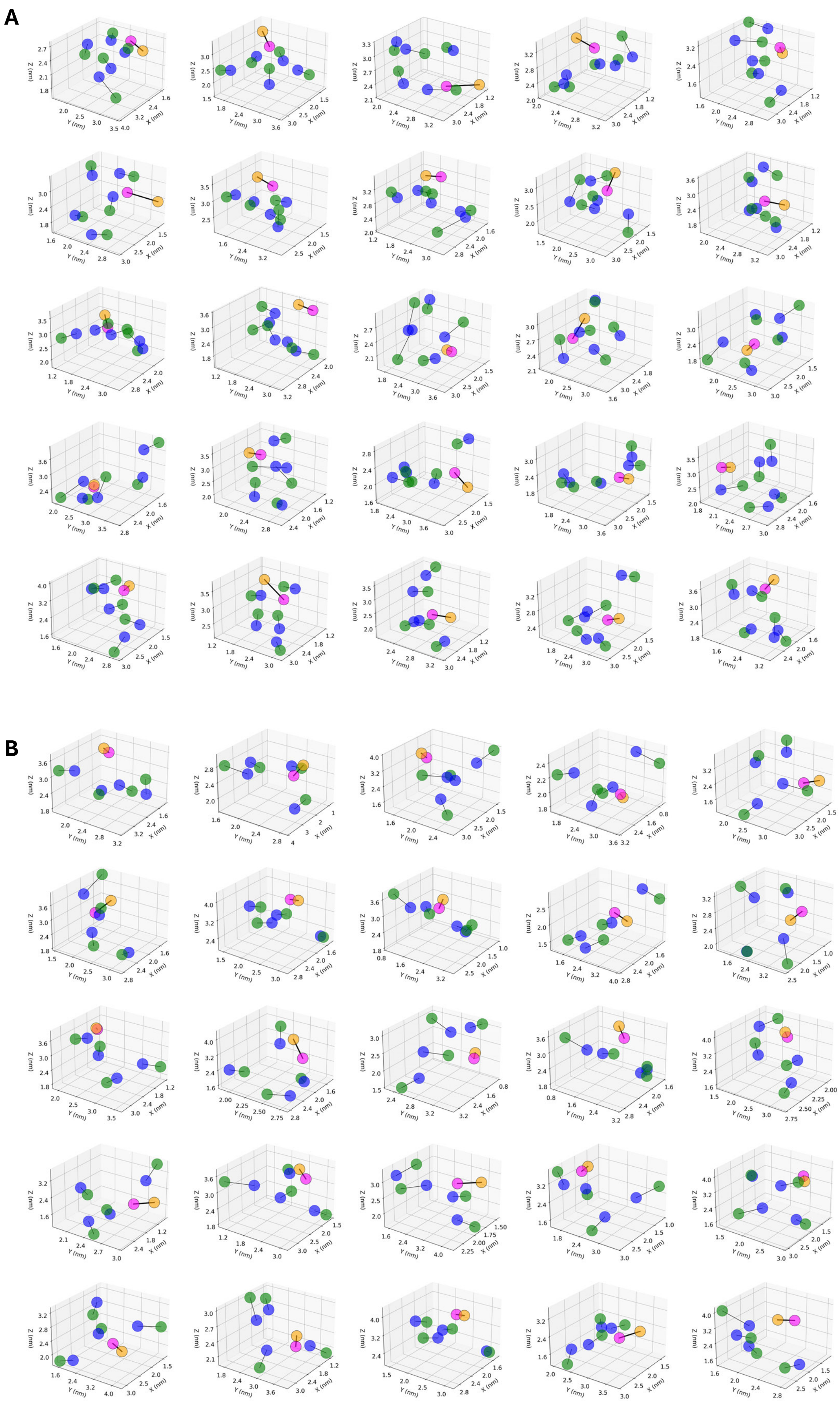


**Supplementary Figure 16. Peptide overlays within the IQR of the Fig. 3C.i RMSD distribution.**

bioRxiv preprint doi: https://doi.org/10.64898/2026.05.31.729018; this version posted June 1, 2026. The copyright holder for this preprint (which was not certified by peer review) is the author/funder, who has granted bioRxiv a license to display the preprint in perpetuity. It is made available under a CC-BY 4.0 International license.

**A**

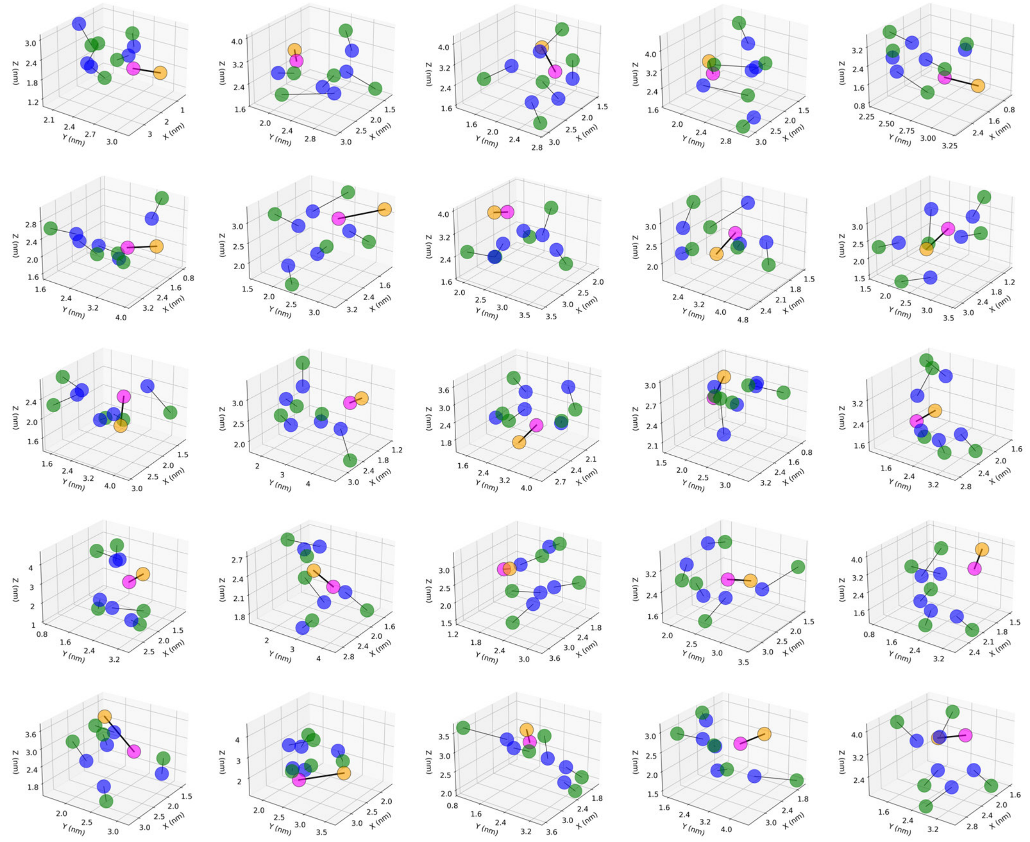

**B**

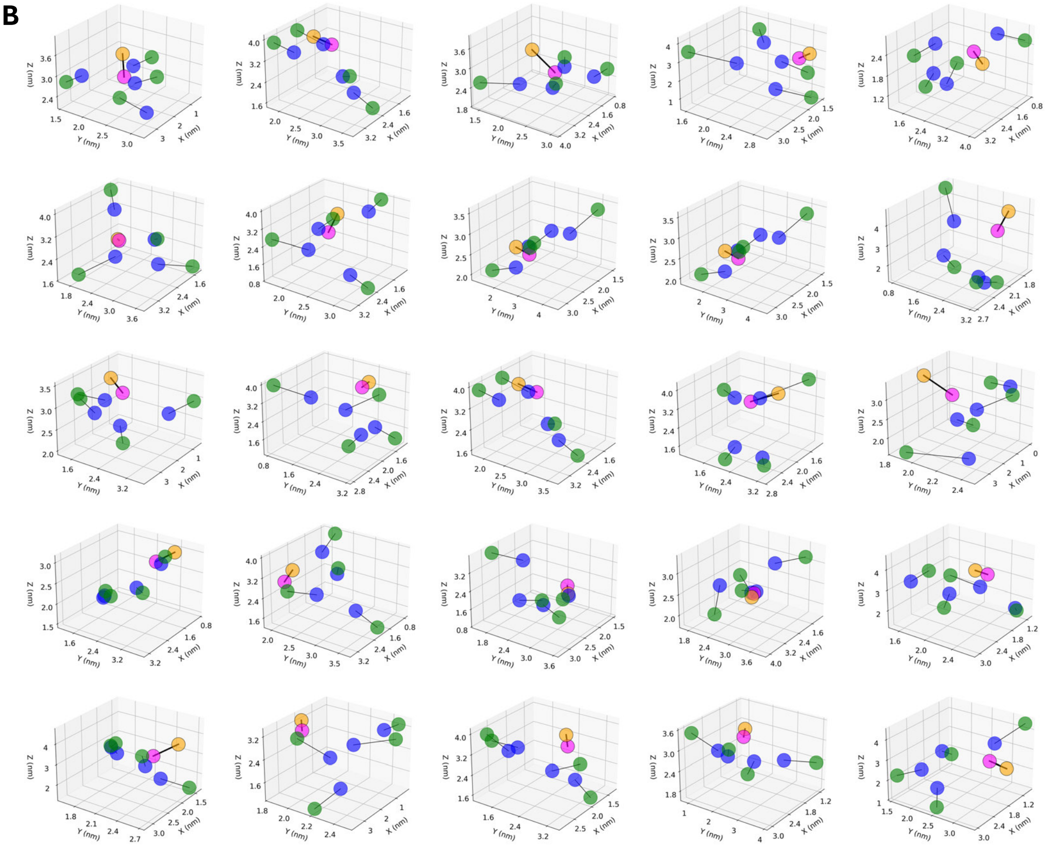

**Supplementary Figure 17. Peptide overlays above the IQR of the Fig. 3C.i RMSD distribution.**

bioRxiv preprint doi: https://doi.org/10.64898/2026.05.31.729018; this version posted June 1, 2026. The copyright holder for this preprint (which was not certified by peer review) is the author/funder, who has granted bioRxiv a license to display the preprint in perpetuity. It is made available under a CC-BY 4.0 International license.

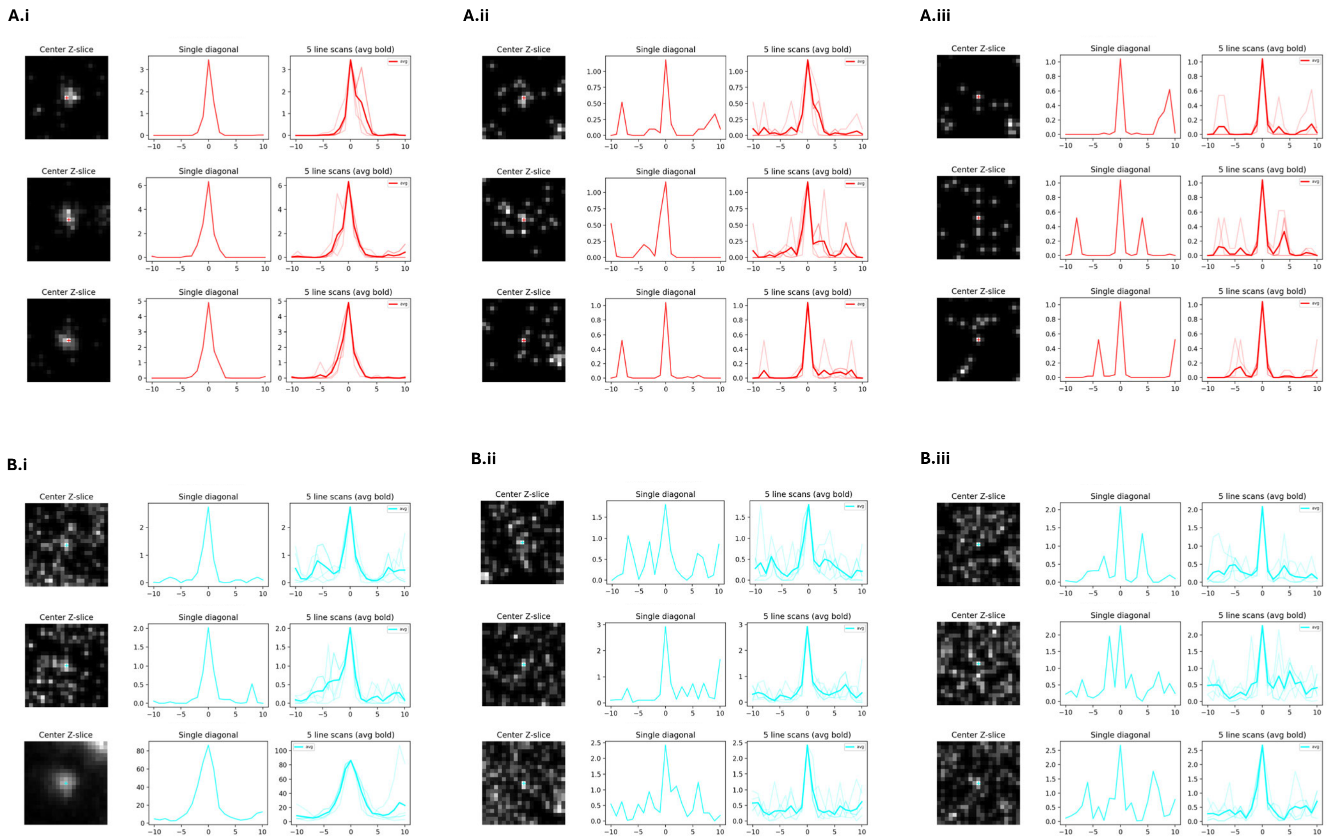


**Supplementary Figure 18. Emitter detection using Gaussian correlation to identify true fluorescent emitters.**

bioRxiv preprint doi: https://doi.org/10.64898/2026.05.31.729018; this version posted June 1, 2026. The copyright holder for this preprint (which was not certified by peer review) is the author/funder, who has granted bioRxiv a license to display the preprint in perpetuity. It is made available under a CC-BY 4.0 International license.

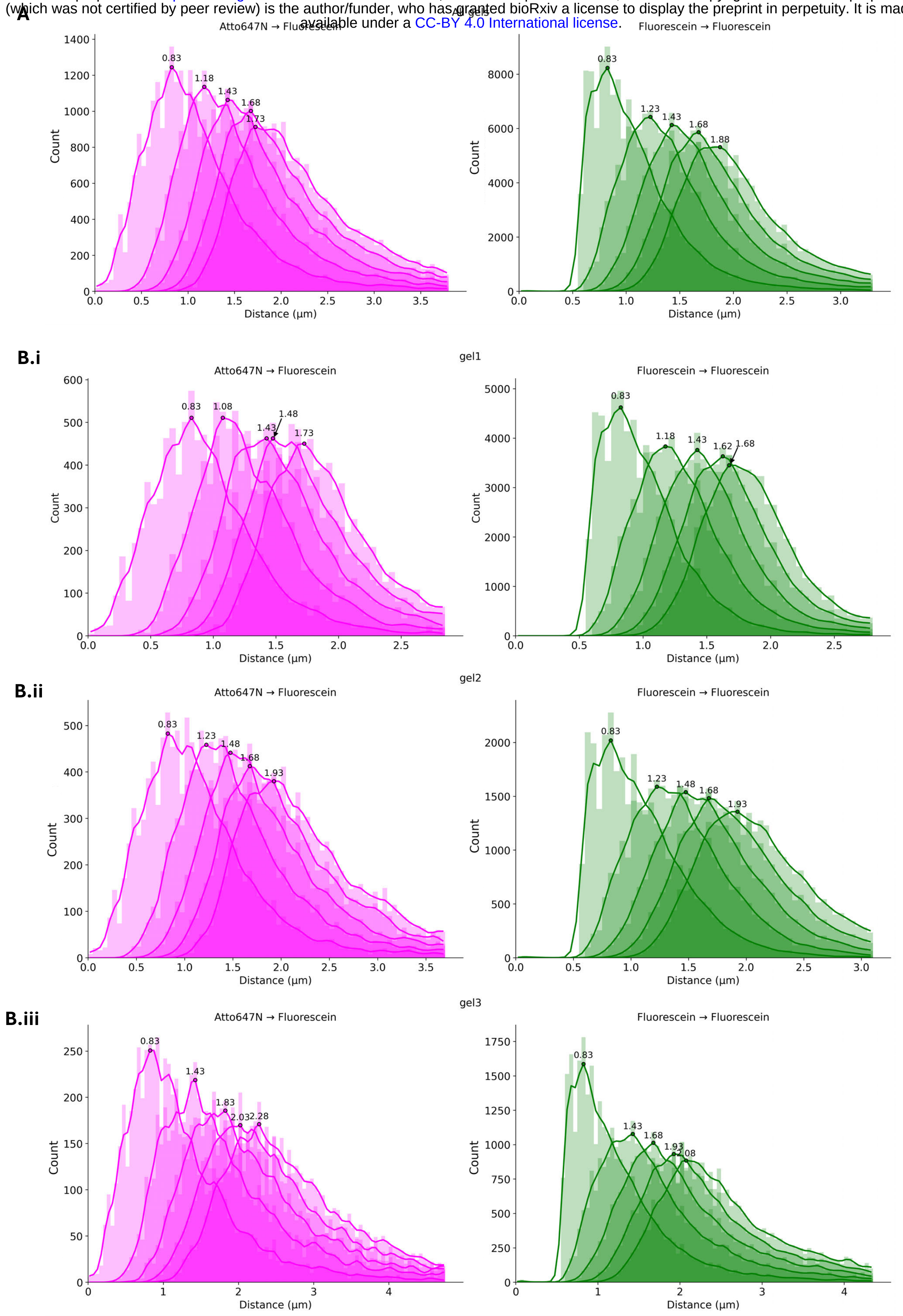


**Supplementary Figure 19. k-nearest-neighbor (kNN) 1–5 distance distributions for Atto647N–fluorescein (magenta) and fluorescein–fluorescein (green).**

bioRxiv preprint doi: https://doi.org/10.64898/2026.05.31.729018; this version posted June 1, 2026. The copyright holder for this preprint (which was not certified by peer review) is the author/funder, who has granted bioRxiv a license to display the preprint in perpetuity. It is made available under a CC-BY 4.0 International license.

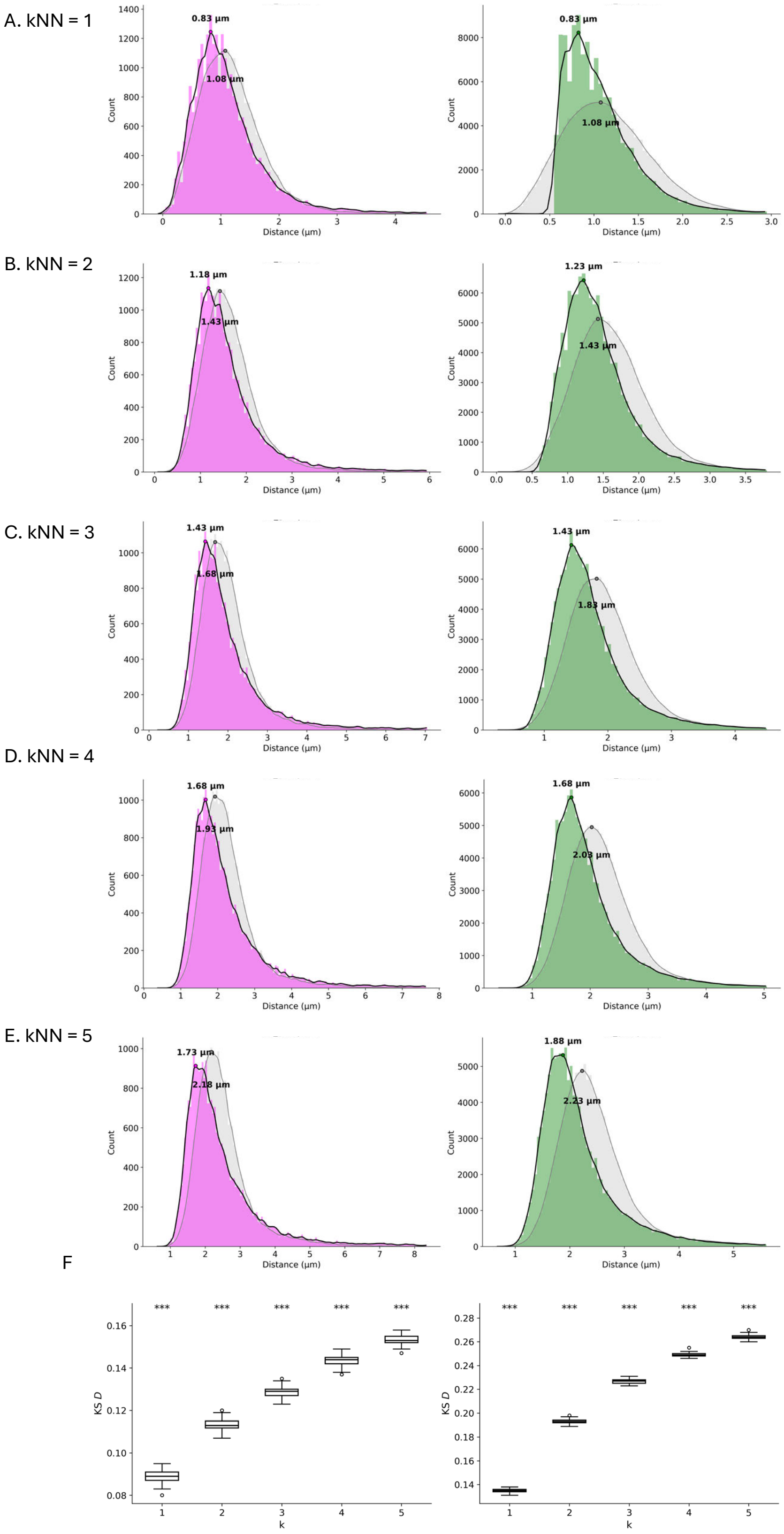


**Supplementary Figure 20. Randomization analysis of k-nearest-neighbor (kNN) distance distributions across all gels.**

bioRxiv preprint doi: https://doi.org/10.64898/2026.05.31.729018; this version posted June 1, 2026. The copyright holder for this preprint (which was not certified by peer review) is the author/funder, who has granted bioRxiv a license to display the preprint in perpetuity. It is made available under a CC-BY 4.0 International license.

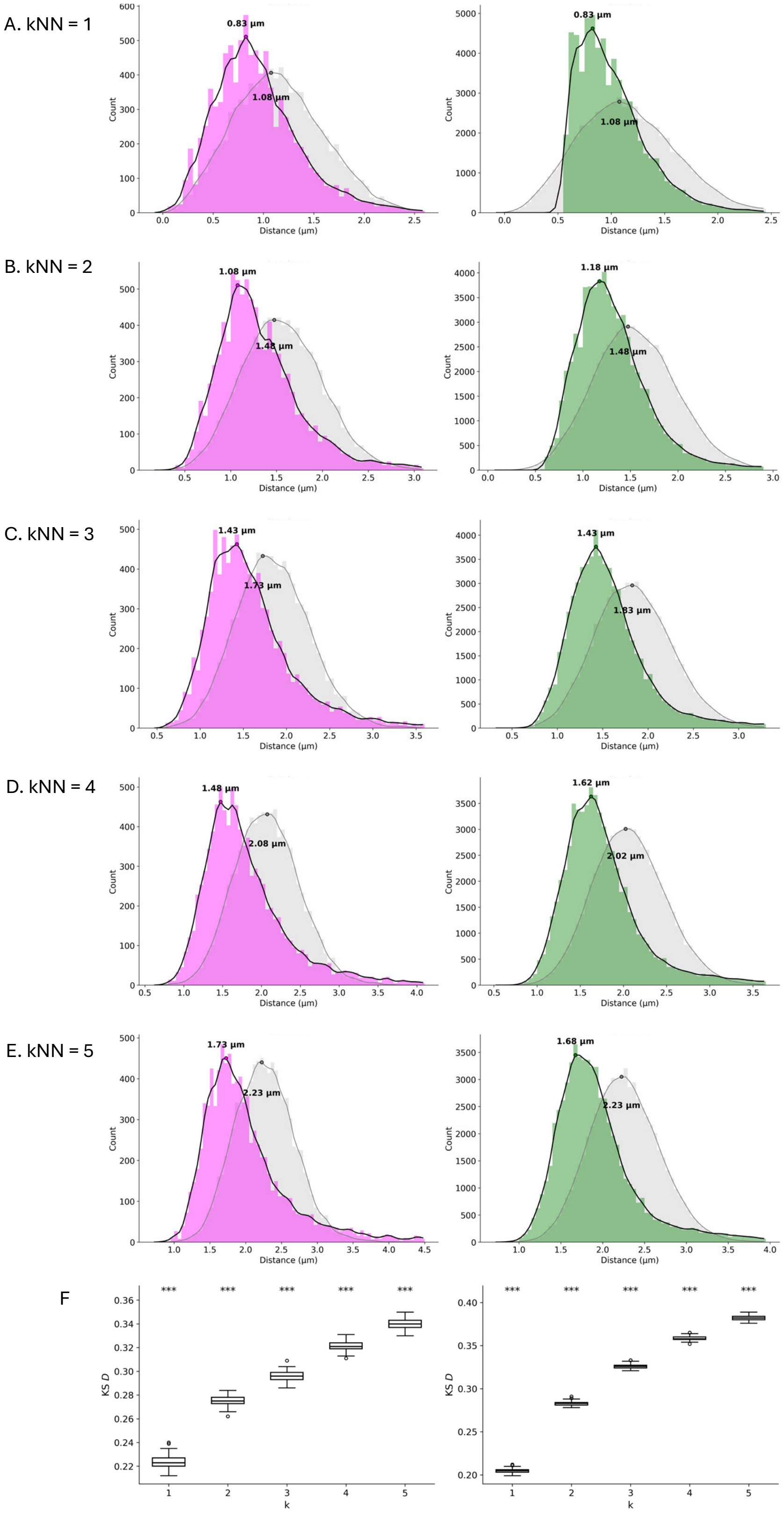


**Supplementary Figure 21. Randomization analysis of k-nearest-neighbor (kNN) distance distributions for gel 1.**

bioRxiv preprint doi: https://doi.org/10.64898/2026.05.31.729018; this version posted June 1, 2026. The copyright holder for this preprint (which was not certified by peer review) is the author/funder, who has granted bioRxiv a license to display the preprint in perpetuity. It is made available under a CC-BY 4.0 International license.

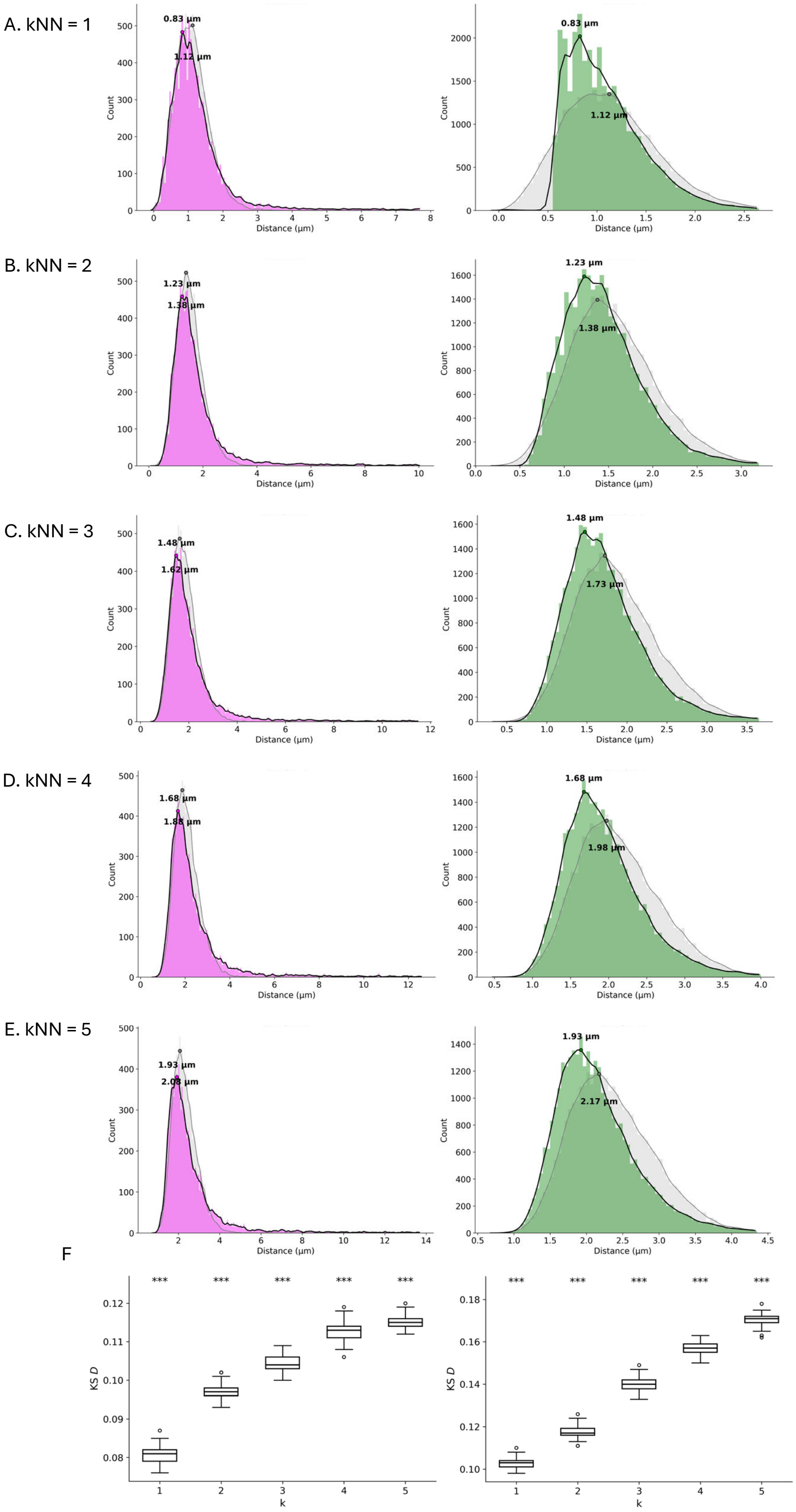


**Supplementary Figure 22. Randomization analysis of k-nearest-neighbor (kNN) distance distributions for gel 2.**

bioRxiv preprint doi: https://doi.org/10.64898/2026.05.31.729018; this version posted June 1, 2026. The copyright holder for this preprint (which was not certified by peer review) is the author/funder, who has granted bioRxiv a license to display the preprint in perpetuity. It is made available under a CC-BY 4.0 International license.

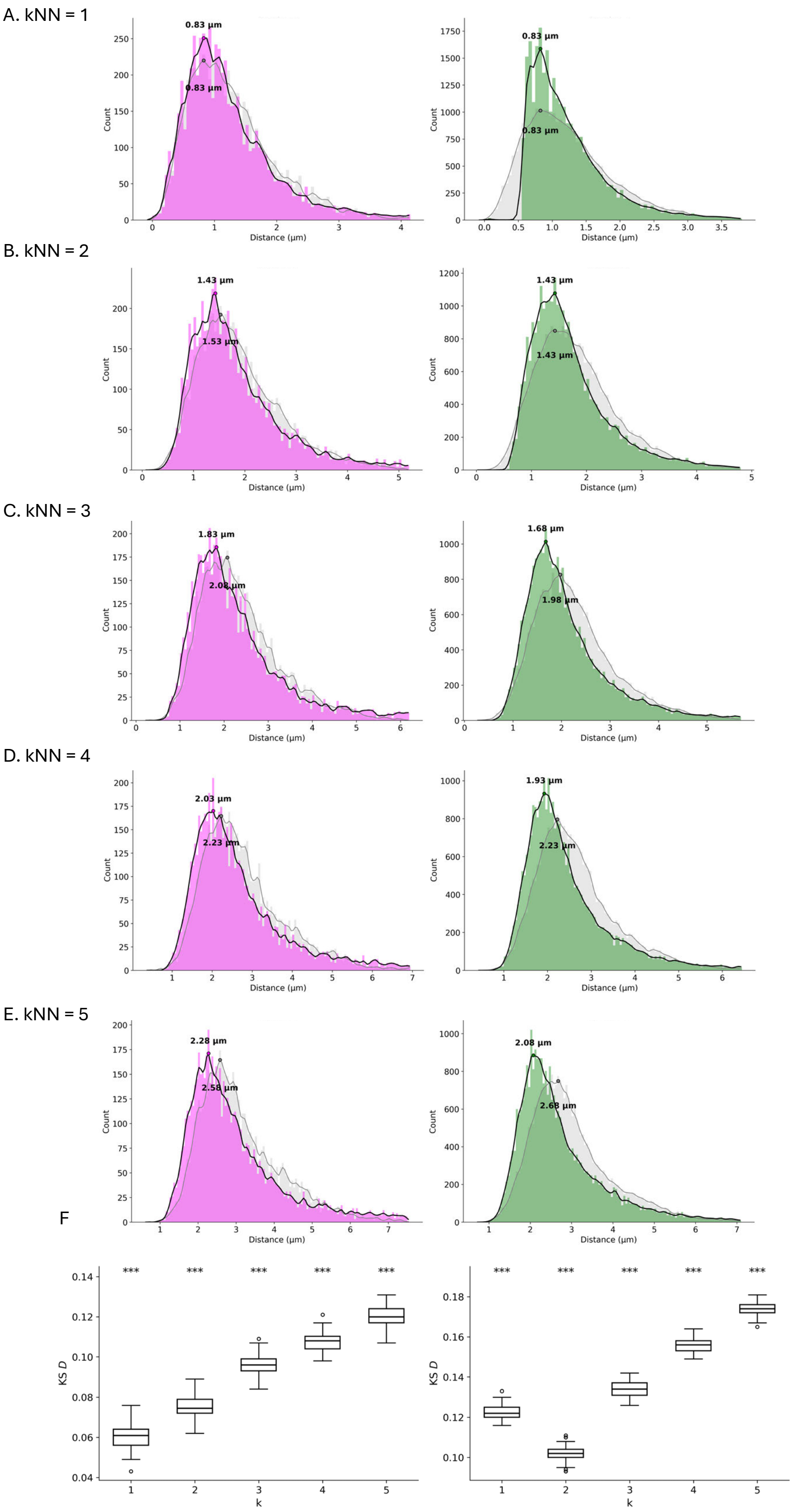


**Supplementary Figure 23. Randomization analysis of k-nearest-neighbor (kNN) distance distributions for gel 3.**

bioRxiv preprint doi: https://doi.org/10.64898/2026.05.31.729018; this version posted June 1, 2026. The copyright holder for this preprint (which was not certified by peer review) is the author/funder, who has granted bioRxiv a license to display the preprint in perpetuity. It is made available under a CC-BY 4.0 International license.

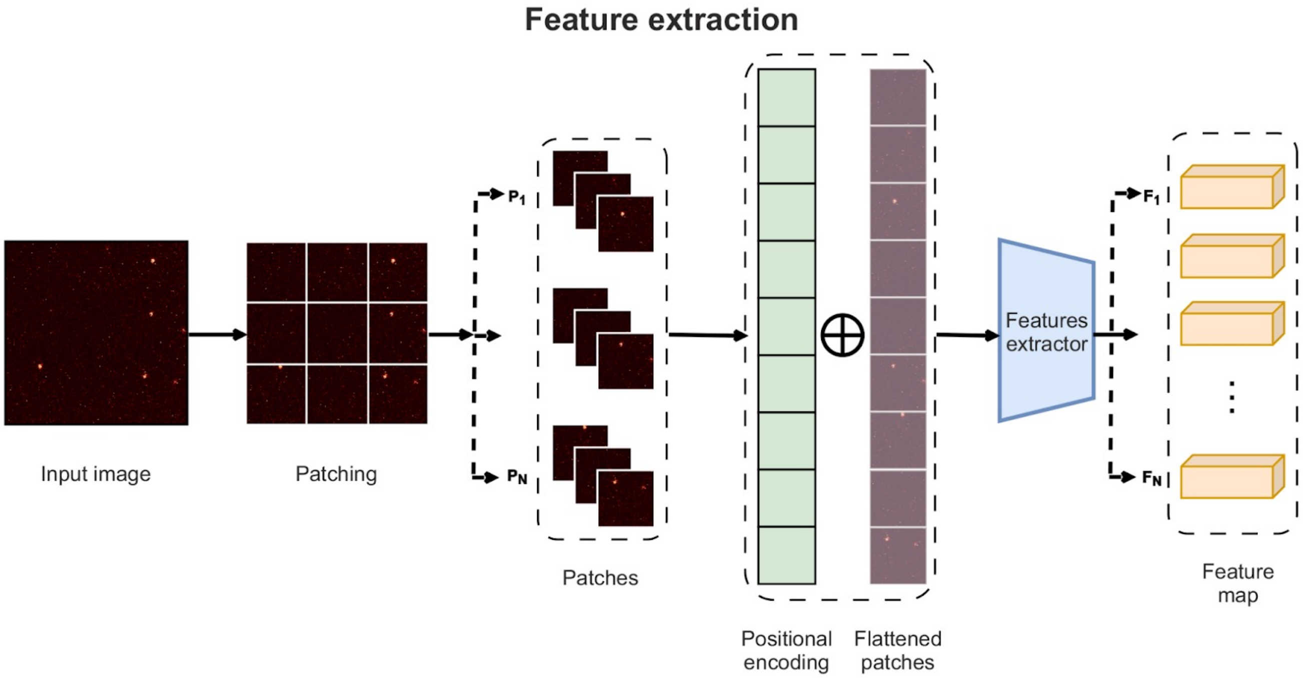


**Supplementary Figure 24. Preprocessing and transformer-based feature extraction pipeline for microscopy images.**

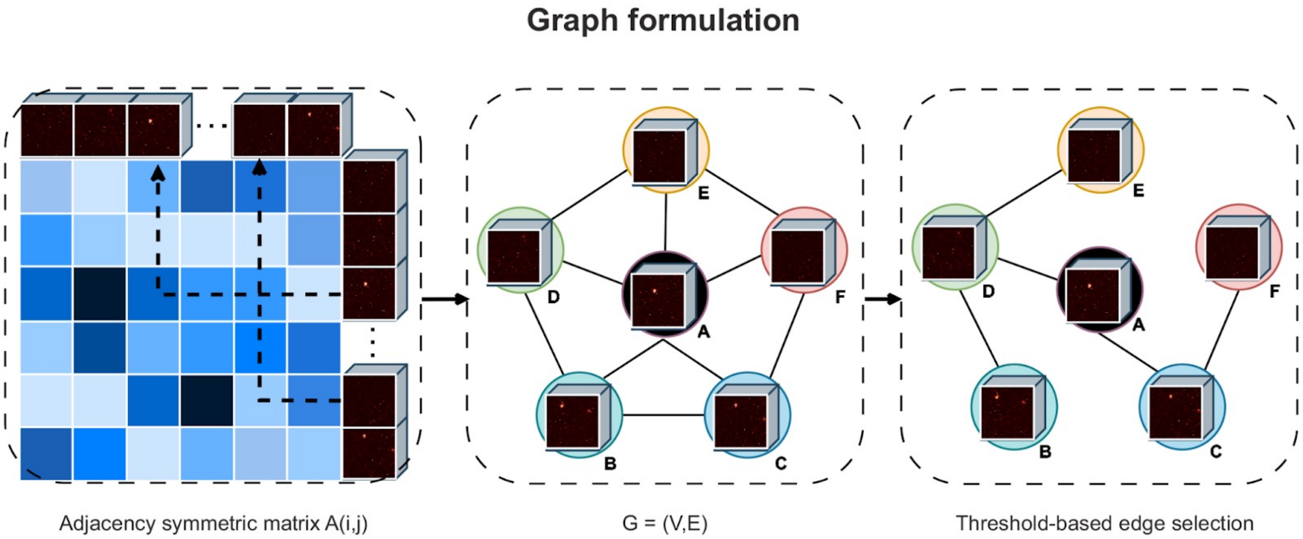


**Supplementary Figure 25. Graph formulation from transformer-derived patch features.**

bioRxiv preprint doi: https://doi.org/10.64898/2026.05.31.729018; this version posted June 1, 2026. The copyright holder for this preprint (which was not certified by peer review) is the author/funder, who has granted bioRxiv a license to display the preprint in perpetuity. It is made available under a CC-BY 4.0 International license.

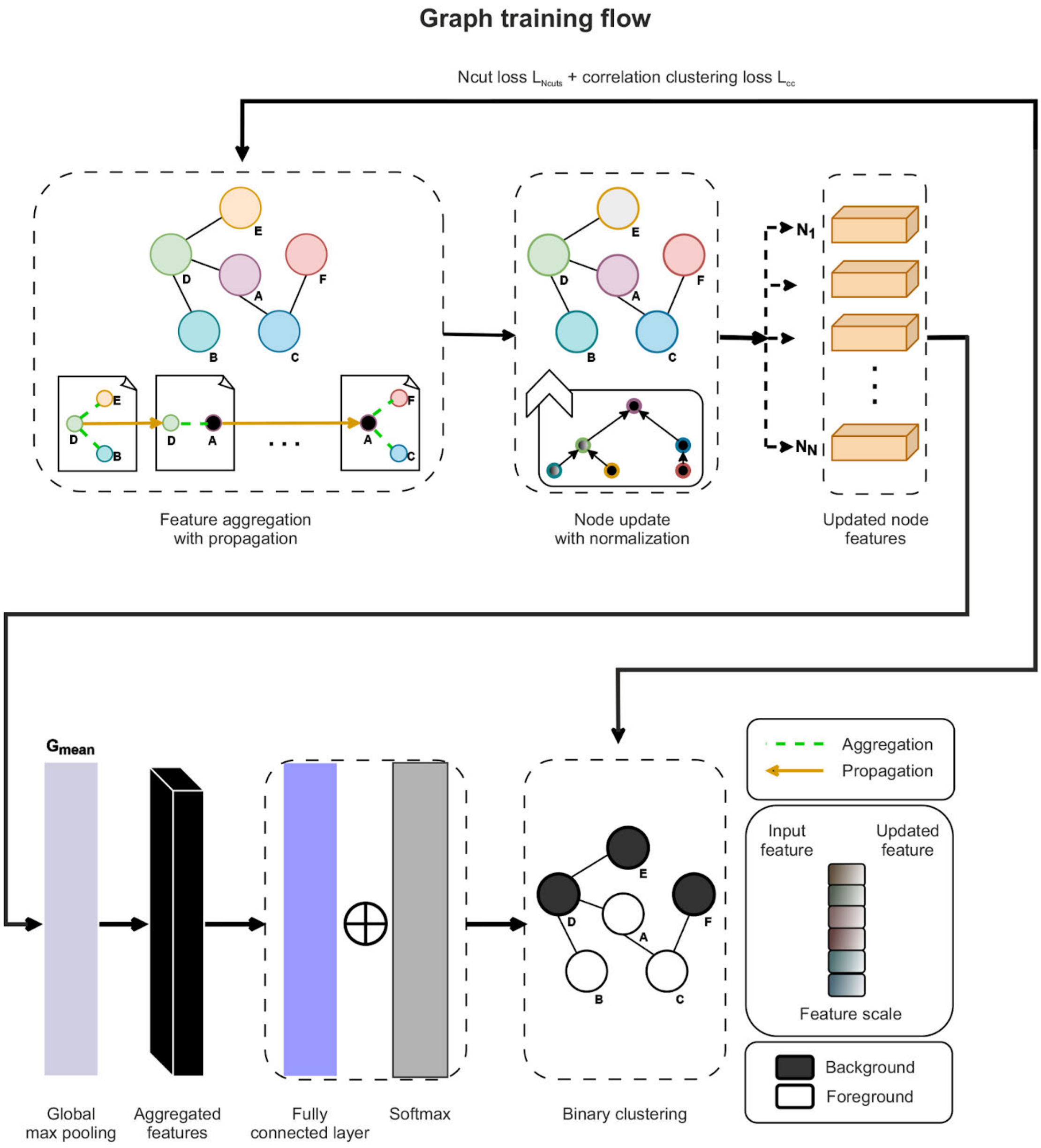


**Supplementary Figure 26. Unsupervised graph-based segmentation framework integrating Normalized Cut and correlation clustering losses.**

bioRxiv preprint doi: https://doi.org/10.64898/2026.05.31.729018; this version posted June 1, 2026. The copyright holder for this preprint (which was not certified by peer review) is the author/funder, who has granted bioRxiv a license to display the preprint in perpetuity. It is made available under a CC-BY 4.0 International license.

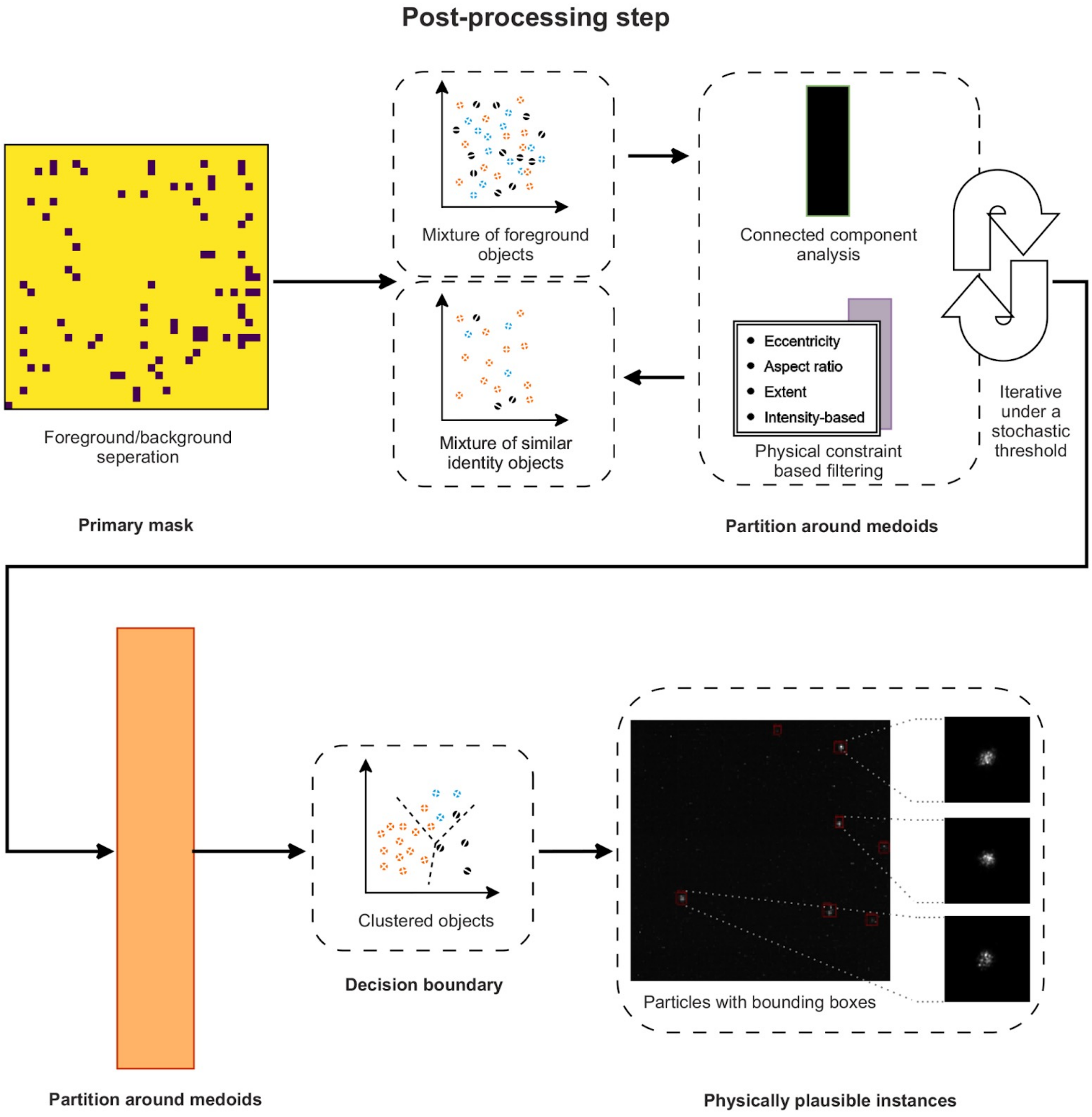


**Supplementary Figure 27. Post-segmentation analysis for separation of heterogeneous foreground objects.**

bioRxiv preprint doi: https://doi.org/10.64898/2026.05.31.729018; this version posted June 1, 2026. The copyright holder for this preprint (which was not certified by peer review) is the author/funder, who has granted bioRxiv a license to display the preprint in perpetuity. It is made available under a CC-BY 4.0 International license.

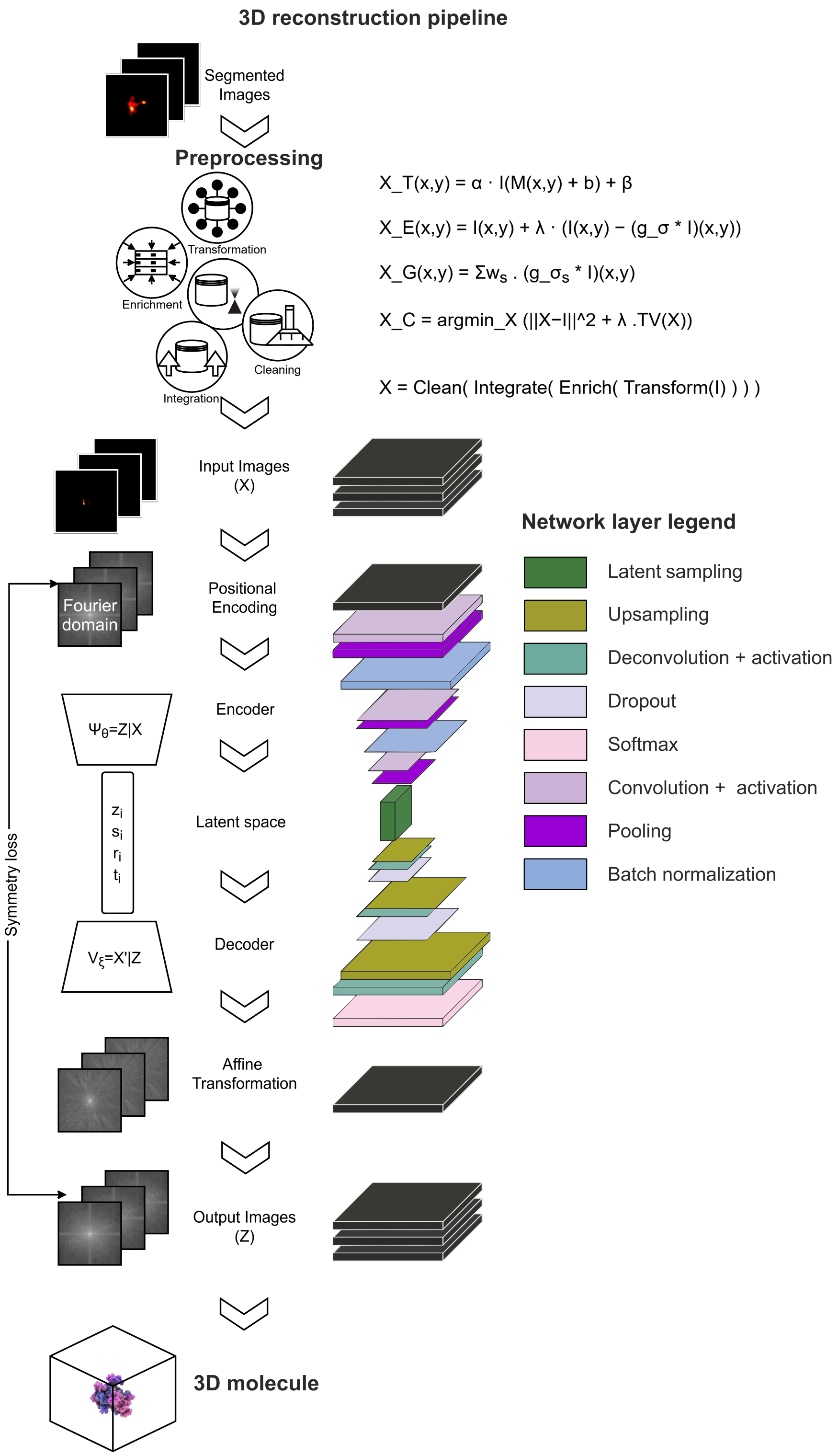


**Supplementary Figure 28. End-to-end *ab initio* 3D reconstruction pipeline from preprocessed 2D images.**

bioRxiv preprint doi: https://doi.org/10.64898/2026.05.31.729018; this version posted June 1, 2026. The copyright holder for this preprint (which was not certified by peer review) is the author/funder, who has granted bioRxiv a license to display the preprint in perpetuity. It is made available under a CC-BY 4.0 International license.

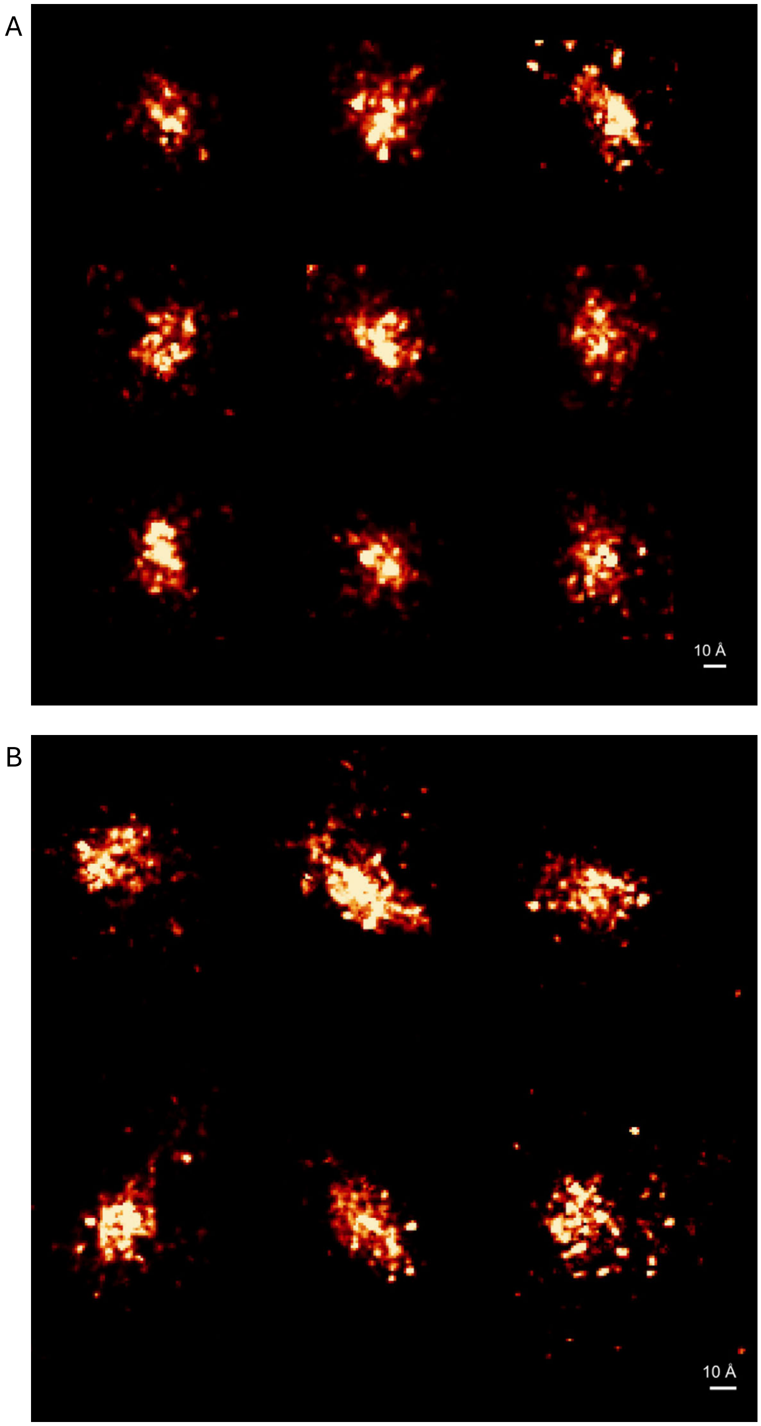


**Supplementary Figure 29. Additional GFP examples at different expansion factors.**

bioRxiv preprint doi: https://doi.org/10.64898/2026.05.31.729018; this version posted June 1, 2026. The copyright holder for this preprint (which was not certified by peer review) is the author/funder, who has granted bioRxiv a license to display the preprint in perpetuity. It is made available under a CC-BY 4.0 International license.

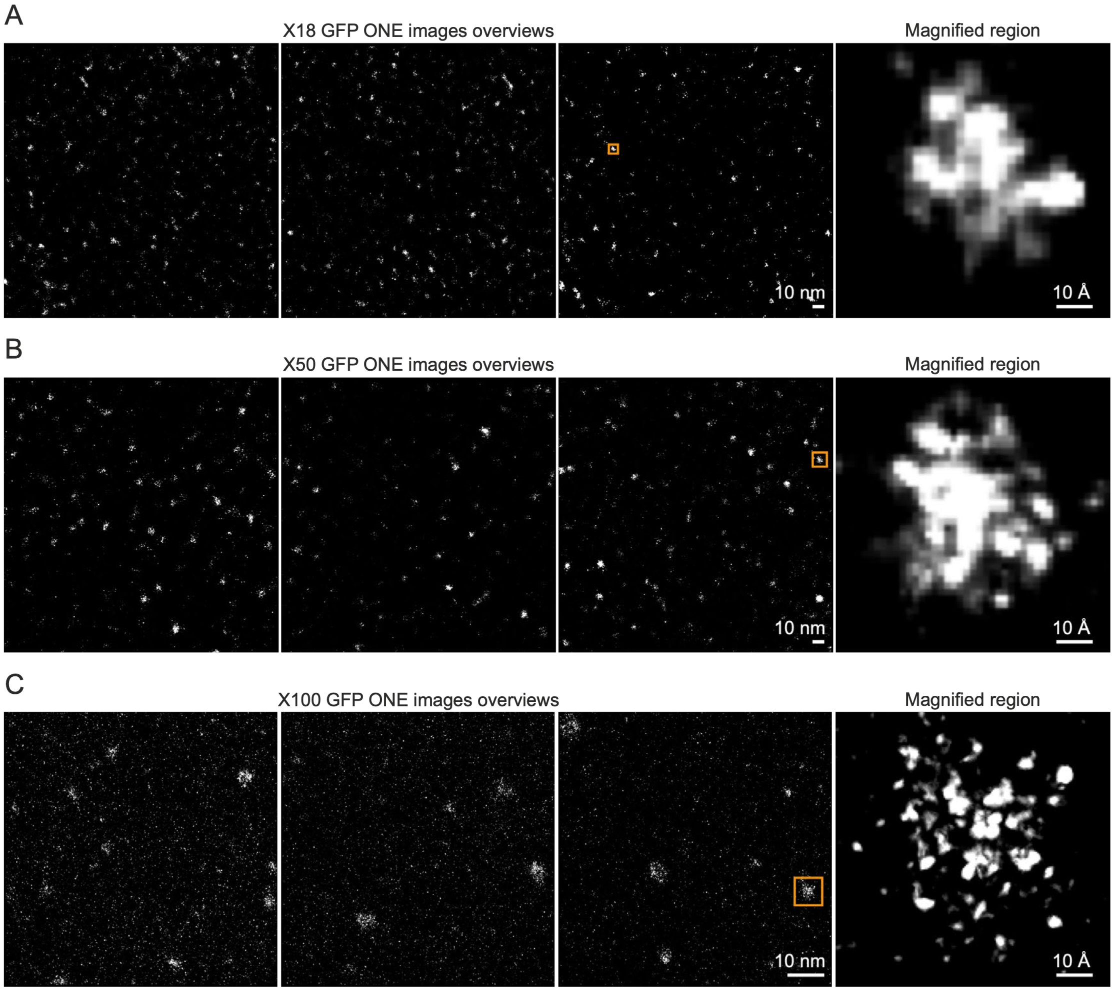


**Supplementary Figure 30. GFP imaged with ONE microscopy across expansion factors.**

bioRxiv preprint doi: https://doi.org/10.64898/2026.05.31.729018; this version posted June 1, 2026. The copyright holder for this preprint (which was not certified by peer review) is the author/funder, who has granted bioRxiv a license to display the preprint in perpetuity. It is made available under a CC-BY 4.0 International license.

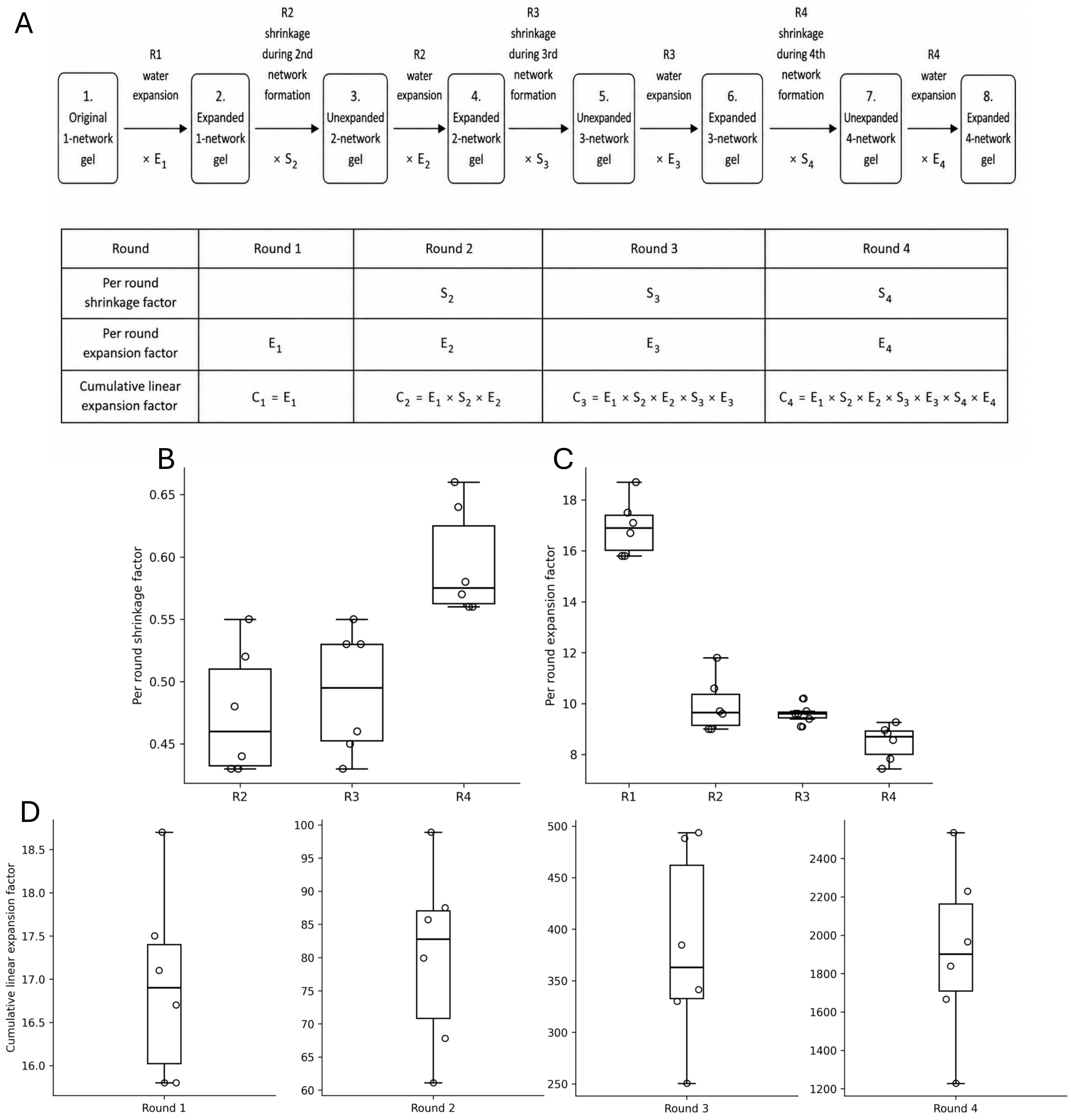


**Supplementary Figure 31. Four-network gels generated using a modified 1000ExM process.**

bioRxiv preprint doi: https://doi.org/10.64898/2026.05.31.729018; this version posted June 1, 2026. The copyright holder for this preprint (which was not certified by peer review) is the author/funder, who has granted bioRxiv a license to display the preprint in perpetuity. It is made available under a CC-BY 4.0 International license.

E

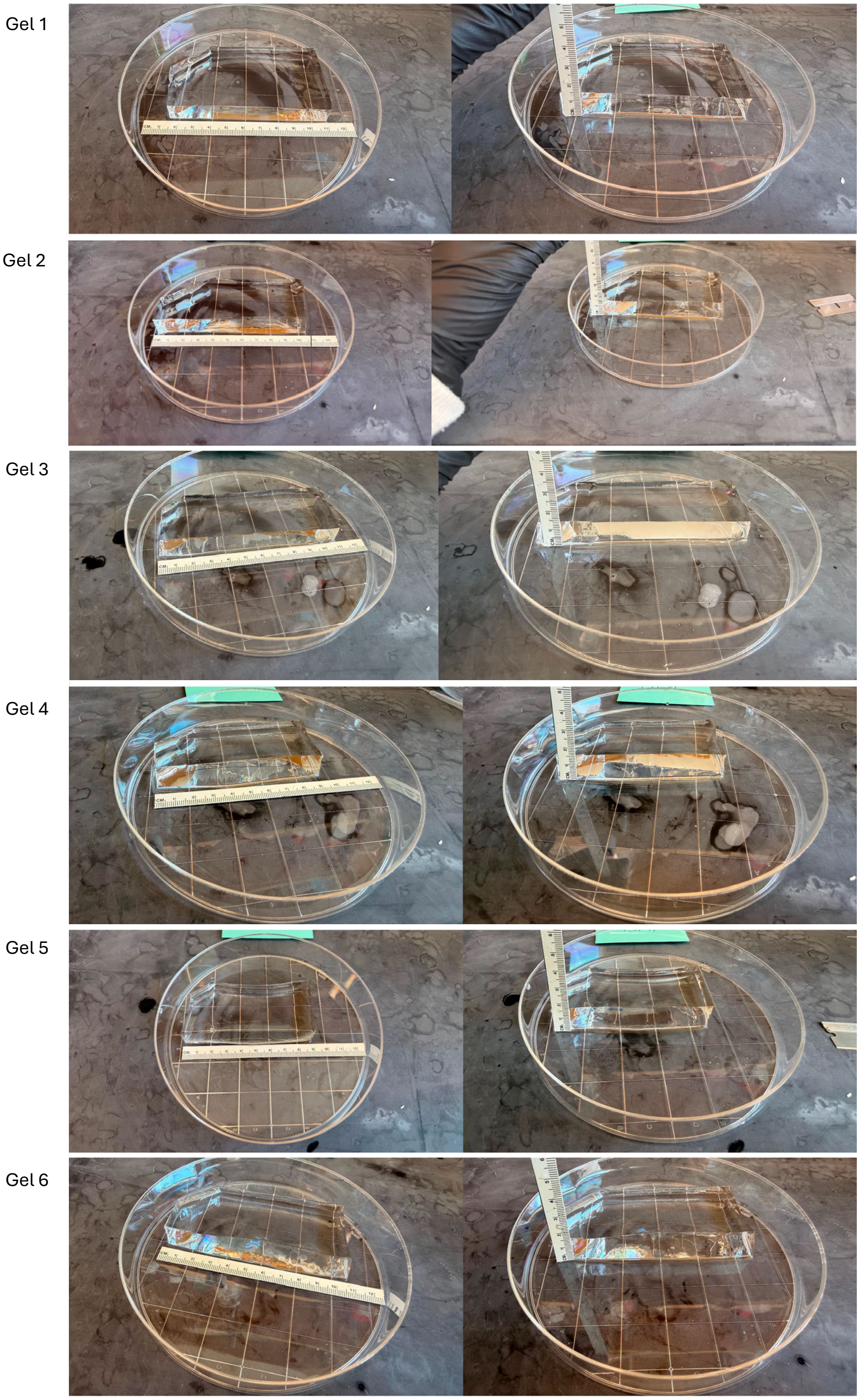


**Supplementary Figure 31. Continued.**

bioRxiv preprint doi: https://doi.org/10.64898/2026.05.31.729018; this version posted June 1, 2026. The copyright holder for this preprint (which was not certified by peer review) is the author/funder, who has granted bioRxiv a license to display the preprint in perpetuity. It is made available under a CC-BY 4.0 International license.

F

Gel 1

Gel 2

Gel 3

Gel 4

Gel 5

Gel 6

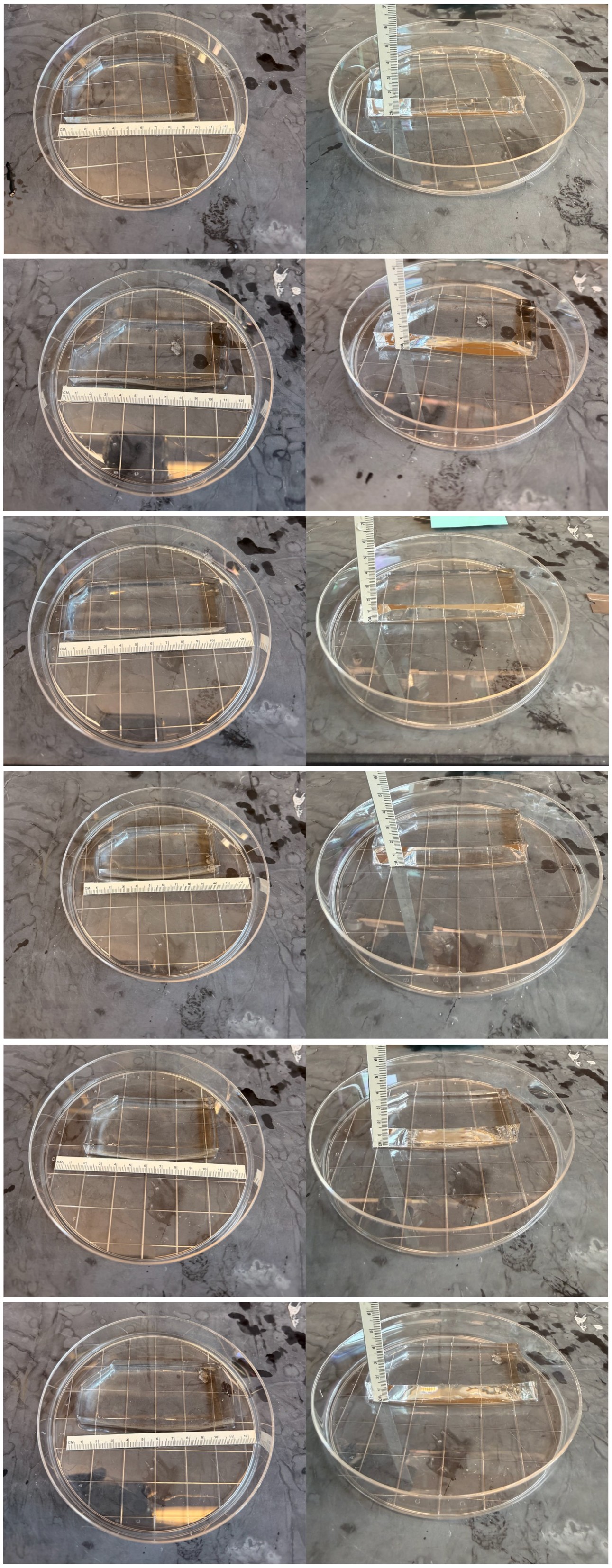

**Supplementary Figure 31. Continued.**

bioRxiv preprint doi: https://doi.org/10.64898/2026.05.31.729018; this version posted June 1, 2026. The copyright holder for this preprint (which was not certified by peer review) is the author/funder, who has granted bioRxiv a license to display the preprint in perpetuity. It is made available under a CC-BY 4.0 International license.

# Supplementary Tables

**Table S1**: x, y, and z dimensions and total volume of the eight imaged 3D regions.

**Table S2**: Atto647N and fluorescein emitter counts after local maxima detection.

**Table S3**: Atto647N and fluorescein emitter counts after local maxima detection and intensity- and size-based filtering

**Table S4**: Atto647N and fluorescein emitter counts after local maxima detection, intensity- and size-based filtering, and Gaussian shape filtering

**Table S5.** Pooled k-nearest neighbor (kNN) distance statistics (k = 1–5) across all gels and all regions (see **Supplementary Figure 19A).**

**Table S6.** Pooled k-nearest neighbor (kNN) distance statistics (k = 1–5) across all regions from gel 1 (see **Supplementary Figure 19B.i**).

**Table S7.** Pooled k-nearest neighbor (kNN) distance statistics (k = 1–5) across all regions from gel 2 (see **Supplementary Figure 19B.ii**).

**Table S8.** Pooled k-nearest neighbor (kNN) distance statistics (k = 1–5) across all regions from gel 3 (see **Supplementary Figure 19B.iii**).

**Table S9**: KS D randomization statistics by kNN rank (1–5) from all gels (see **Supplementary Figure 20**).

**Table S10**: KS D randomization statistics by kNN rank (1–5) from gel 1 (see **Supplementary Figure 21**).

**Table S11**: KS D randomization statistics by kNN rank (1–5) from gel 2 (see **Supplementary Figure 22**).

**Table S12**: KS D randomization statistics by kNN rank (1–5) from gel 3 (see **Supplementary Figure 23**).

**Table S13**: Summary statistics of single-molecule expansion factors (**Supplementary Figure 11A**), per-axis RMSD (**Supplementary Figure 10A**), and total RMSD (see **Supplementary Figure 9A**, boxplots; **Supplementary Figure 12A**, histograms) for all putative peptides pooled across all gels and regions.

**Table S14**: Summary statistics of single-molecule expansion factors (**Supplementary Figure 11B**), per-axis RMSD (**Supplementary Figure 10B**), and total RMSD (see **Supplementary Figure 9A**, boxplots; **Supplementary Figure 12A**, histograms) for all putative peptides, stratified by gel (Gel 1-3).

**Table S15**: Summary statistics of single-molecule expansion factors (**Supplementary Figure 11C**), per-axis RMSD (**Supplementary Figure 10C**), and total RMSD (see **Supplementary Figure 9C**, boxplots; **Supplementary Figure 12C**, histograms) for all putative peptides, stratified by imaging region across Gel 1-3 (n=8 regions).

bioRxiv preprint doi: https://doi.org/10.64898/2026.05.31.729018; this version posted June 1, 2026. The copyright holder for this preprint (which was not certified by peer review) is the author/funder, who has granted bioRxiv a license to display the preprint in perpetuity. It is made available under a CC-BY 4.0 International license.

**Table S16.** Summary statistics of total RMSD distributions as a function of the number of molecular-dynamics (MD) reference conformations (see **Supplementary Figure 13**, boxplots; **Supplementary Figure 14**, histograms).

**Table S17**: Statistics elements for **Fig. 2**.

**Table S18.** Measured gel lengths and calculated shrinkage and expansion factors during four-network gel formation. For each round, the table reports lengths before and after expansion, per-round expansion factors, and cumulative linear expansion factors. For rounds 2–4, the table also reports lengths before monomer addition and after gelation, which were used to calculate per-round shrinkage factors. Cumulative linear expansion factors were calculated by multiplying all sequential shrinkage and expansion factors through the indicated round.

bioRxiv preprint doi: https://doi.org/10.64898/2026.05.31.729018; this version posted June 1, 2026. The copyright holder for this preprint (which was not certified by peer review) is the author/funder, who has granted bioRxiv a license to display the preprint in perpetuity. It is made available under a CC-BY 4.0 International license.

| gel_region | Z thickness (μm) | Y width (μm) | X height (μm) | Volume (μm³) |
|---|---|---|---|---|
| gel1:region1 | 4.4 | 279.09 | 279.09 | $3.42 \times 10^5$ |
| gel1:region2 | 4.4 | 279.09 | 279.09 | $3.42 \times 10^5$ |
| gel2:region1 | 3.4 | 139.59 | 232.59 | $1.10 \times 10^5$ |
| gel2:region2 | 3.4 | 226.49 | 131.4 | $1.01 \times 10^5$ |
| gel2:region3 | 3.4 | 223.31 | 134.95 | $1.02 \times 10^5$ |
| gel2:region4 | 3.4 | 179.18 | 90.82 | $5.53 \times 10^4$ |
| gel3:region1 | 2.2 | 1906.59 | 46.59 | $1.95 \times 10^5$ |
| gel3:region2 | 2.2 | 260.98 | 340.42 | $1.95 \times 10^5$ |

**Table S1**: x, y, and z dimensions and total volume of the eight imaged 3D regions

| gel_region | Atto647N emitters | Fluorescein emitters | Fluorescein / Atto647N |
|---|---|---|---|
| gel1:region1 | 584,761 | 436,827 | **0.75** |
| gel1:region2 | 589,176 | 437,280 | **0.74** |
| gel2:region1 | 172,503 | 145,811 | **0.85** |
| gel2:region2 | 1,309,470 | 709,989 | **0.54** |
| gel2:region3 | 472,152 | 152,744 | **0.32** |
| gel2:region4 | 98,142 | 82,268 | **0.84** |
| gel3:region1 | 349,057 | 287,299 | **0.82** |
| gel3:region2 | 17,664,588 | 16,559,923 | **0.94** |

**Table S2:** Atto647N and fluorescein emitter counts after local maxima detection

| gel_region | Atto647N emitters | Fluorescein emitters | Fluorescein / Atto647N |
|---|---|---|---|
| gel1:region1 | 6,280 | 150,786 | **24** |
| gel1:region2 | 6,773 | 154,567 | **22.8** |
| gel2:region1 | 5,415 | 35,235 | **6.51** |
| gel2:region2 | 11,688 | 50,809 | **4.35** |
| gel2:region3 | 3,998 | 23,157 | **5.79** |
| gel2:region4 | 351 | 8,863 | **25.3** |
| gel3:region1 | 4,962 | 36,289 | **7.31** |
| gel3:region2 | 9,664 | 128,097 | **13.3** |

**Table S3:** Atto647N and fluorescein emitter counts after local maxima detection and intensity- and size-based filtering

| gel_region | Atto647N emitters | Fluorescein emitters | Fluorescein / Atto647N |
|---|---|---|---|
| gel1:region1 | 4,445 | 31,567 | **7.1** |
| gel1:region2 | 4,821 | 32,166 | **6.67** |
| gel2:region1 | 3,254 | 7,206 | **2.21** |
| gel2:region2 | 6,074 | 13,444 | **2.21** |
| gel2:region3 | 2,133 | 8,573 | **4.02** |
| gel2:region4 | 210 | 3,542 | **16.9** |
| gel3:region1 | 2,225 | 8,161 | **3.67** |
| gel3:region2 | 4,264 | 20,736 | **4.86** |

**Table S4:** Atto647N and fluorescein emitter counts after local maxima detection, intensity- and size-based filtering, and Gaussian shape filtering

bioRxiv preprint doi: https://doi.org/10.64898/2026.05.31.729018; this version posted June 1, 2026. The copyright holder for this preprint (which was not certified by peer review) is the author/funder, who has granted bioRxiv a license to display the preprint in perpetuity. It is made available under a CC-BY 4.0 International license.

**Atto647N to Fluorescein**

| subset | k (neighbor order) | n (distance pairs) | Mean (µm) | STD (µm) | Median (µm) | P25 (µm) | P75 (µm) | P5 (µm) | P95 (µm) | P1 (µm) | P99 (µm) | Histogram peak (µm) |
|---|---|---|---|---|---|---|---|---|---|---|---|---|
| all_gels | 1 | 27426 | 1.249 | 1.231 | 1.004 | 0.701 | 1.4 | 0.384 | 2.638 | 0.203 | 7.127 | 0.825 |
| all_gels | 2 | 27426 | 1.758 | 1.547 | 1.416 | 1.101 | 1.876 | 0.76 | 3.538 | 0.599 | 9.48 | 1.175 |
| all_gels | 3 | 27426 | 2.126 | 1.822 | 1.704 | 1.365 | 2.247 | 1.021 | 4.221 | 0.825 | 10.906 | 1.425 |
| all_gels | 4 | 27426 | 2.433 | 2.149 | 1.945 | 1.578 | 2.557 | 1.218 | 4.775 | 1.025 | 11.899 | 1.675 |
| all_gels | 5 | 27426 | 2.7 | 2.365 | 2.158 | 1.754 | 2.838 | 1.384 | 5.294 | 1.198 | 12.963 | 1.725 |

**Fluorescein to Fluorescein**

| subset | k (neighbor order) | n (distance pairs) | Mean (µm) | STD (µm) | Median (µm) | P25 (µm) | P75 (µm) | P5 (µm) | P95 (µm) | P1 (µm) | P99 (µm) | Histogram peak (µm) |
|---|---|---|---|---|---|---|---|---|---|---|---|---|
| all_gels | 1 | 125395 | 1.101 | 0.525 | 0.986 | 0.774 | 1.275 | 0.61 | 1.939 | 0.567 | 2.981 | 0.825 |
| all_gels | 2 | 125395 | 1.476 | 0.671 | 1.339 | 1.093 | 1.671 | 0.825 | 2.562 | 0.692 | 3.914 | 1.225 |
| all_gels | 3 | 125395 | 1.769 | 0.785 | 1.6 | 1.33 | 1.963 | 1.037 | 3.053 | 0.874 | 4.732 | 1.425 |
| all_gels | 4 | 125395 | 2.015 | 0.889 | 1.811 | 1.534 | 2.222 | 1.221 | 3.463 | 1.038 | 5.387 | 1.675 |
| all_gels | 5 | 125395 | 2.232 | 0.978 | 1.998 | 1.698 | 2.451 | 1.38 | 3.826 | 1.2 | 5.968 | 1.875 |

**Table S5.** Pooled k-nearest neighbor (kNN) distance statistics (k = 1–5) across all gels and all regions (see **Supplementary Figure 19A).**

**Atto647N to Fluorescein**

| subset | k (neighbor order) | n (distance pairs) | Mean (µm) | STD (µm) | Median (µm) | P25 (µm) | P75 (µm) | P5 (µm) | P95 (µm) | P1 (µm) | P99 (µm) | Histogram peak (µm) |
|---|---|---|---|---|---|---|---|---|---|---|---|---|
| gel1 | 1 | 9266 | 0.986 | 0.704 | 0.883 | 0.643 | 1.186 | 0.35 | 1.841 | 0.203 | 2.834 | 0.825 |
| gel1 | 2 | 9266 | 1.381 | 0.868 | 1.24 | 1.017 | 1.559 | 0.713 | 2.311 | 0.583 | 3.569 | 1.075 |
| gel1 | 3 | 9266 | 1.665 | 1.004 | 1.5 | 1.249 | 1.832 | 0.96 | 2.72 | 0.8 | 4.21 | 1.425 |
| gel1 | 4 | 9266 | 1.898 | 1.136 | 1.691 | 1.437 | 2.06 | 1.153 | 3.124 | 0.989 | 4.777 | 1.475 |
| gel1 | 5 | 9266 | 2.101 | 1.26 | 1.872 | 1.602 | 2.256 | 1.305 | 3.45 | 1.151 | 5.531 | 1.725 |

**Fluorescein to Fluorescein**

| subset | k (neighbor order) | n (distance pairs) | Mean (µm) | STD (µm) | Median (µm) | P25 (µm) | P75 (µm) | P5 (µm) | P95 (µm) | P1 (µm) | P99 (µm) | Histogram peak (µm) |
|---|---|---|---|---|---|---|---|---|---|---|---|---|
| gel1 | 1 | 63733 | 1.021 | 0.422 | 0.937 | 0.758 | 1.182 | 0.609 | 1.669 | 0.567 | 2.295 | 0.825 |
| gel1 | 2 | 63733 | 1.342 | 0.53 | 1.254 | 1.047 | 1.515 | 0.809 | 2.101 | 0.683 | 3.025 | 1.175 |
| gel1 | 3 | 63733 | 1.592 | 0.606 | 1.493 | 1.268 | 1.758 | 1.011 | 2.484 | 0.862 | 3.559 | 1.425 |
| gel1 | 4 | 63733 | 1.8 | 0.678 | 1.681 | 1.453 | 1.961 | 1.181 | 2.832 | 1.019 | 4.005 | 1.625 |
| gel1 | 5 | 63733 | 1.983 | 0.739 | 1.842 | 1.608 | 2.139 | 1.329 | 3.131 | 1.17 | 4.415 | 1.675 |

**Table S6.** Pooled k-nearest neighbor (kNN) distance statistics (k = 1–5) across all regions from gel 1 (see **Supplementary Figure 19B.i**).

**Atto647N to Fluorescein**

| subset | k (neighbor order) | n (distance pairs) | Mean (µm) | STD (µm) | Median (µm) | P25 (µm) | P75 (µm) | P5 (µm) | P95 (µm) | P1 (µm) | P99 (µm) | Histogram peak (µm) |
|---|---|---|---|---|---|---|---|---|---|---|---|---|
| gel2 | 1 | 11671 | 1.443 | 1.643 | 1.079 | 0.757 | 1.502 | 0.41 | 3.404 | 0.22 | 9.989 | 0.825 |
| gel2 | 2 | 11671 | 1.981 | 2.065 | 1.479 | 1.163 | 1.966 | 0.803 | 4.533 | 0.608 | 12.921 | 1.225 |
| gel2 | 3 | 11671 | 2.368 | 2.444 | 1.763 | 1.425 | 2.313 | 1.047 | 5.313 | 0.843 | 15.236 | 1.475 |
| gel2 | 4 | 11671 | 2.699 | 2.92 | 2.004 | 1.632 | 2.632 | 1.252 | 5.988 | 1.05 | 16.898 | 1.675 |
| gel2 | 5 | 11671 | 2.981 | 3.213 | 2.212 | 1.811 | 2.909 | 1.431 | 6.686 | 1.207 | 19.129 | 1.925 |

**Fluorescein to Fluorescein**

| subset | k (neighbor order) | n (distance pairs) | Mean (µm) | STD (µm) | Median (µm) | P25 (µm) | P75 (µm) | P5 (µm) | P95 (µm) | P1 (µm) | P99 (µm) | Histogram peak (µm) |
|---|---|---|---|---|---|---|---|---|---|---|---|---|
| gel2 | 1 | 32765 | 1.11 | 0.509 | 1.017 | 0.79 | 1.309 | 0.61 | 1.887 | 0.567 | 2.55 | 0.825 |
| gel2 | 2 | 32765 | 1.478 | 0.628 | 1.383 | 1.119 | 1.706 | 0.835 | 2.341 | 0.693 | 3.266 | 1.225 |
| gel2 | 3 | 32765 | 1.76 | 0.733 | 1.653 | 1.375 | 1.996 | 1.061 | 2.728 | 0.881 | 3.747 | 1.475 |
| gel2 | 4 | 32765 | 1.995 | 0.831 | 1.866 | 1.58 | 2.239 | 1.252 | 3.031 | 1.058 | 4.205 | 1.675 |
| gel2 | 5 | 32765 | 2.205 | 0.91 | 2.063 | 1.756 | 2.464 | 1.413 | 3.323 | 1.232 | 4.608 | 1.925 |

**Table S7.** Pooled k-nearest neighbor (kNN) distance statistics (k = 1–5) across all regions from gel 2 (see **Supplementary Figure 19B.ii**).

**Atto647N to Fluorescein**

| subset | k (neighbor order) | n (distance pairs) | Mean (µm) | STD (µm) | Median (µm) | P25 (µm) | P75 (µm) | P5 (µm) | P95 (µm) | P1 (µm) | P99 (µm) | Histogram peak (µm) |
|---|---|---|---|---|---|---|---|---|---|---|---|---|
| gel3 | 1 | 6489 | 1.274 | 0.821 | 1.082 | 0.734 | 1.592 | 0.384 | 2.888 | 0.203 | 4.344 | 0.825 |
| gel3 | 2 | 6489 | 1.896 | 1.026 | 1.638 | 1.219 | 2.274 | 0.817 | 3.958 | 0.612 | 5.745 | 1.425 |
| gel3 | 3 | 6489 | 2.348 | 1.181 | 2.035 | 1.568 | 2.743 | 1.112 | 4.806 | 0.869 | 6.631 | 1.825 |
| gel3 | 4 | 6489 | 2.721 | 1.309 | 2.36 | 1.857 | 3.146 | 1.367 | 5.466 | 1.112 | 7.398 | 2.025 |
| gel3 | 5 | 6489 | 3.052 | 1.428 | 2.653 | 2.113 | 3.506 | 1.598 | 6.118 | 1.317 | 8.041 | 2.275 |

**Fluorescein to Fluorescein**

| subset | k (neighbor order) | n (distance pairs) | Mean (µm) | STD (µm) | Median (µm) | P25 (µm) | P75 (µm) | P5 (µm) | P95 (µm) | P1 (µm) | P99 (µm) | Histogram peak (µm) |
|---|---|---|---|---|---|---|---|---|---|---|---|---|
| gel3 | 1 | 28897 | 1.267 | 0.684 | 1.085 | 0.815 | 1.495 | 0.612 | 2.548 | 0.567 | 3.942 | 0.825 |
| gel3 | 2 | 28897 | 1.768 | 0.872 | 1.557 | 1.203 | 2.055 | 0.862 | 3.463 | 0.707 | 5.089 | 1.425 |
| gel3 | 3 | 28897 | 2.169 | 1.013 | 1.911 | 1.521 | 2.492 | 1.118 | 4.117 | 0.916 | 5.985 | 1.675 |
| gel3 | 4 | 28897 | 2.51 | 1.134 | 2.201 | 1.795 | 2.867 | 1.363 | 4.708 | 1.129 | 6.921 | 1.925 |
| gel3 | 5 | 28897 | 2.81 | 1.24 | 2.458 | 2.025 | 3.209 | 1.571 | 5.232 | 1.329 | 7.704 | 2.075 |

**Table S8.** Pooled k-nearest neighbor (kNN) distance statistics (k = 1–5) across all regions from gel 3 (see **Supplementary Figure 19B.iii**).

bioRxiv preprint doi: https://doi.org/10.64898/2026.05.31.729018; this version posted June 1, 2026. The copyright holder for this preprint (which was not certified by peer review) is the author/funder, who has granted bioRxiv a license to display the preprint in perpetuity. It is made available under a CC-BY 4.0 International license.

**Atto647N to Fluorescein**

| k (neighbor order) | Mean (µm) | STD (µm) | Median (µm) | P25 (µm) | P75 (µm) | P5 (µm) | P95 (µm) | KS tests with p < 0.001 (out of 100) | KS tests with p ≥ 0.001 (out of 100) |
|---|---|---|---|---|---|---|---|---|---|
| 1 | 0.089 | 0.003 | 0.089 | 0.087 | 0.091 | 0.085 | 0.094 | 100 | 0 |
| 2 | 0.113 | 0.003 | 0.113 | 0.112 | 0.115 | 0.108 | 0.118 | 100 | 0 |
| 3 | 0.129 | 0.003 | 0.129 | 0.127 | 0.13 | 0.124 | 0.133 | 100 | 0 |
| 4 | 0.144 | 0.002 | 0.144 | 0.142 | 0.145 | 0.141 | 0.148 | 100 | 0 |
| 5 | 0.153 | 0.002 | 0.153 | 0.152 | 0.155 | 0.15 | 0.157 | 100 | 0 |

**Fluorescein to Fluorescein**

| k (neighbor order) | Mean (µm) | STD (µm) | Median (µm) | P25 (µm) | P75 (µm) | P5 (µm) | P95 (µm) | KS tests with p < 0.001 (out of 100) | KS tests with p ≥ 0.001 (out of 100) |
|---|---|---|---|---|---|---|---|---|---|
| 1 | 0.135 | 0.001 | 0.135 | 0.134 | 0.136 | 0.133 | 0.137 | 100 | 0 |
| 2 | 0.193 | 0.002 | 0.193 | 0.192 | 0.194 | 0.19 | 0.196 | 100 | 0 |
| 3 | 0.227 | 0.002 | 0.227 | 0.225 | 0.228 | 0.224 | 0.229 | 100 | 0 |
| 4 | 0.249 | 0.002 | 0.249 | 0.248 | 0.25 | 0.247 | 0.252 | 100 | 0 |
| 5 | 0.264 | 0.002 | 0.264 | 0.263 | 0.265 | 0.261 | 0.267 | 100 | 0 |

**Table S9**: KS D randomization statistics by kNN rank (1–5) from all gels (see **Supplementary Figure 20**).

**Atto647N to Fluorescein**

| k (neighbor order) | Mean (µm) | STD (µm) | Median (µm) | P25 (µm) | P75 (µm) | P5 (µm) | P95 (µm) | KS tests with p < 0.001 (out of 100) | KS tests with p ≥ 0.001 (out of 100) |
|---|---|---|---|---|---|---|---|---|---|
| 1 | 0.224 | 0.005 | 0.223 | 0.22 | 0.227 | 0.217 | 0.234 | 100 | 0 |
| 2 | 0.275 | 0.004 | 0.275 | 0.273 | 0.278 | 0.268 | 0.283 | 100 | 0 |
| 3 | 0.296 | 0.004 | 0.296 | 0.293 | 0.299 | 0.29 | 0.302 | 100 | 0 |
| 4 | 0.322 | 0.004 | 0.321 | 0.319 | 0.324 | 0.315 | 0.328 | 100 | 0 |
| 5 | 0.34 | 0.004 | 0.34 | 0.337 | 0.343 | 0.333 | 0.347 | 100 | 0 |

**Fluorescein to Fluorescein**

| k (neighbor order) | Mean (µm) | STD (µm) | Median (µm) | P25 (µm) | P75 (µm) | P5 (µm) | P95 (µm) | KS tests with p < 0.001 (out of 100) | KS tests with p ≥ 0.001 (out of 100) |
|---|---|---|---|---|---|---|---|---|---|
| 1 | 0.205 | 0.002 | 0.205 | 0.203 | 0.206 | 0.201 | 0.209 | 100 | 0 |
| 2 | 0.283 | 0.003 | 0.283 | 0.281 | 0.284 | 0.279 | 0.287 | 100 | 0 |
| 3 | 0.326 | 0.002 | 0.326 | 0.324 | 0.327 | 0.323 | 0.33 | 100 | 0 |
| 4 | 0.359 | 0.002 | 0.358 | 0.357 | 0.36 | 0.355 | 0.362 | 100 | 0 |
| 5 | 0.382 | 0.003 | 0.382 | 0.38 | 0.384 | 0.378 | 0.386 | 100 | 0 |

**Table S10**: KS D randomization statistics by kNN rank (1–5) from gel 1 (see **Supplementary Figure 21**).

**Atto647N to Fluorescein**

| k (neighbor order) | Mean (µm) | STD (µm) | Median (µm) | P25 (µm) | P75 (µm) | P5 (µm) | P95 (µm) | KS tests with p < 0.001 (out of 100) | KS tests with p ≥ 0.001 (out of 100) |
|---|---|---|---|---|---|---|---|---|---|
| 1 | 0.224 | 0.005 | 0.223 | 0.22 | 0.227 | 0.217 | 0.234 | 100 | 0 |
| 2 | 0.275 | 0.004 | 0.275 | 0.273 | 0.278 | 0.268 | 0.283 | 100 | 0 |
| 3 | 0.296 | 0.004 | 0.296 | 0.293 | 0.299 | 0.29 | 0.302 | 100 | 0 |
| 4 | 0.322 | 0.004 | 0.321 | 0.319 | 0.324 | 0.315 | 0.328 | 100 | 0 |
| 5 | 0.34 | 0.004 | 0.34 | 0.337 | 0.343 | 0.333 | 0.347 | 100 | 0 |

**Fluorescein to Fluorescein**

| k (neighbor order) | Mean (µm) | STD (µm) | Median (µm) | P25 (µm) | P75 (µm) | P5 (µm) | P95 (µm) | KS tests with p < 0.001 (out of 100) | KS tests with p ≥ 0.001 (out of 100) |
|---|---|---|---|---|---|---|---|---|---|
| 1 | 0.205 | 0.002 | 0.205 | 0.203 | 0.206 | 0.201 | 0.209 | 100 | 0 |
| 2 | 0.283 | 0.003 | 0.283 | 0.281 | 0.284 | 0.279 | 0.287 | 100 | 0 |
| 3 | 0.326 | 0.002 | 0.326 | 0.324 | 0.327 | 0.323 | 0.33 | 100 | 0 |
| 4 | 0.359 | 0.002 | 0.358 | 0.357 | 0.36 | 0.355 | 0.362 | 100 | 0 |
| 5 | 0.382 | 0.003 | 0.382 | 0.38 | 0.384 | 0.378 | 0.386 | 100 | 0 |

**Table S11**: KS D randomization statistics by kNN rank (1–5) from gel 2 (see **Supplementary Figure 22**).

**Atto647N to Fluorescein**

| k (neighbor order) | Mean (µm) | STD (µm) | Median (µm) | P25 (µm) | P75 (µm) | P5 (µm) | P95 (µm) | KS tests with p < 0.001 (out of 100) | KS tests with p ≥ 0.001 (out of 100) |
|---|---|---|---|---|---|---|---|---|---|
| 1 | 0.06 | 0.006 | 0.061 | 0.056 | 0.064 | 0.052 | 0.069 | 100 | 0 |
| 2 | 0.075 | 0.005 | 0.075 | 0.072 | 0.079 | 0.067 | 0.083 | 100 | 0 |
| 3 | 0.096 | 0.005 | 0.096 | 0.093 | 0.099 | 0.087 | 0.104 | 100 | 0 |
| 4 | 0.108 | 0.005 | 0.108 | 0.104 | 0.11 | 0.101 | 0.115 | 100 | 0 |
| 5 | 0.12 | 0.005 | 0.12 | 0.117 | 0.124 | 0.113 | 0.128 | 100 | 0 |

**Fluorescein to Fluorescein**

| k (neighbor order) | Mean (µm) | STD (µm) | Median (µm) | P25 (µm) | P75 (µm) | P5 (µm) | P95 (µm) | KS tests with p < 0.001 (out of 100) | KS tests with p ≥ 0.001 (out of 100) |
|---|---|---|---|---|---|---|---|---|---|
| 1 | 0.122 | 0.003 | 0.122 | 0.12 | 0.125 | 0.117 | 0.127 | 100 | 0 |
| 2 | 0.102 | 0.003 | 0.102 | 0.1 | 0.104 | 0.096 | 0.107 | 100 | 0 |
| 3 | 0.134 | 0.004 | 0.134 | 0.131 | 0.137 | 0.128 | 0.14 | 100 | 0 |
| 4 | 0.156 | 0.004 | 0.156 | 0.153 | 0.158 | 0.15 | 0.163 | 100 | 0 |
| 5 | 0.174 | 0.003 | 0.174 | 0.172 | 0.176 | 0.169 | 0.179 | 100 | 0 |

**Table S12**: KS D randomization statistics by kNN rank (1–5) from gel 3 (see **Supplementary Figure 23**).

bioRxiv preprint doi: https://doi.org/10.64898/2026.05.31.729018; this version posted June 1, 2026. The copyright holder for this preprint (which was not certified by peer review) is the author/funder, who has granted bioRxiv a license to display the preprint in perpetuity. It is made available under a CC-BY 4.0 International license.

| All Gels | n (molecules) | Mean | STD | Median | P25 | P75 | P10 | P90 |
|---|---|---|---|---|---|---|---|---|
| Single-molecule expansion factor | 6284 | 1055.92 | 21.554 | 1058.372 | 1043.009 | 1071.639 | 1027.077 | 1081.537 |
| RMSD (x-axis, nm) | 6284 | 0.317 | 0.121 | 0.308 | 0.228 | 0.392 | 0.17 | 0.475 |
| RMSD (y-axis, nm) | 6284 | 0.316 | 0.12 | 0.306 | 0.228 | 0.393 | 0.166 | 0.475 |
| RMSD (z-axis, nm) | 6284 | 0.325 | 0.126 | 0.312 | 0.232 | 0.404 | 0.174 | 0.493 |
| Total RMSD (nm) | 6284 | 0.573 | 0.151 | 0.572 | 0.476 | 0.668 | 0.372 | 0.764 |

**Table S13**: Summary statistics of single-molecule expansion factors (**Supplementary Figure 11A**), per-axis RMSD (**Supplementary Figure 10A**), and total RMSD (see **Supplementary Figure 9A**, boxplots; **Supplementary Figure 12A**, histograms) for all putative peptides pooled across all gels and regions.

| _ | n (molecules) | Mean | STD | Median | P25 | P75 | P10 | P90 |
|---|---|---|---|---|---|---|---|---|
| **Gel 1** | | | | | | | | |
| Single-molecule expansion factor | 3155 | 1054.678 | 20.745 | 1057.095 | 1042.238 | 1069.63 | 1026.919 | 1079.035 |
| RMSD (x-axis, nm) | 3155 | 0.313 | 0.117 | 0.305 | 0.225 | 0.386 | 0.17 | 0.466 |
| RMSD (y-axis, nm) | 3155 | 0.314 | 0.117 | 0.305 | 0.23 | 0.387 | 0.17 | 0.471 |
| RMSD (z-axis, nm) | 3155 | 0.318 | 0.118 | 0.308 | 0.231 | 0.393 | 0.175 | 0.474 |
| Total RMSD (nm) | 3155 | 0.565 | 0.14 | 0.567 | 0.476 | 0.655 | 0.378 | 0.742 |
| **Gel 2** | | | | | | | | |
| Single-molecule expansion factor | 1918 | 1055.137 | 22.519 | 1057.447 | 1041.44 | 1071.891 | 1025.949 | 1081.618 |
| RMSD (x-axis, nm) | 1918 | 0.313 | 0.123 | 0.303 | 0.225 | 0.393 | 0.164 | 0.48 |
| RMSD (y-axis, nm) | 1918 | 0.31 | 0.12 | 0.304 | 0.219 | 0.389 | 0.156 | 0.469 |
| RMSD (z-axis, nm) | 1918 | 0.322 | 0.13 | 0.305 | 0.225 | 0.405 | 0.166 | 0.493 |
| Total RMSD (nm) | 1918 | 0.565 | 0.16 | 0.562 | 0.461 | 0.665 | 0.352 | 0.772 |
| **Gel 3** | | | | | | | | |
| Single-molecule expansion factor | 1211 | 1060.396 | 21.496 | 1063.804 | 1047.249 | 1076.174 | 1030.608 | 1085.43 |
| RMSD (x-axis, nm) | 1211 | 0.331 | 0.124 | 0.32 | 0.241 | 0.405 | 0.184 | 0.496 |
| RMSD (y-axis, nm) | 1211 | 0.329 | 0.129 | 0.315 | 0.231 | 0.414 | 0.17 | 0.501 |
| RMSD (z-axis, nm) | 1211 | 0.349 | 0.137 | 0.332 | 0.251 | 0.444 | 0.182 | 0.53 |
| Total RMSD (nm) | 1211 | 0.604 | 0.158 | 0.6 | 0.5 | 0.708 | 0.401 | 0.811 |

**Table S14**: Summary statistics of single-molecule expansion factors (**Supplementary Figure 11B**), per-axis RMSD (**Supplementary Figure 10B**), and total RMSD (see **Supplementary Figure 9A**, boxplots; **Supplementary Figure 12A**, histograms) for all putative peptides, stratified by gel (Gel 1-3).

bioRxiv preprint doi: https://doi.org/10.64898/2026.05.31.729018; this version posted June 1, 2026. The copyright holder for this preprint (which was not certified by peer review) is the author/funder, who has granted bioRxiv a license to display the preprint in perpetuity. It is made available under a CC-BY 4.0 International license.

| _ | n (molecules) | Mean | STD | Median | P25 | P75 | P10 | P90 |
|---|---|---|---|---|---|---|---|---|
| **Gel 1, Region 1** | | | | | | | | |
| Single-molecule expansion factor | 1531 | 1055.032 | 20.607 | 1057.373 | 1042.318 | 1070.055 | 1027.387 | 1079.503 |
| RMSD (x-axis, nm) | 1531 | 0.315 | 0.119 | 0.306 | 0.226 | 0.387 | 0.171 | 0.474 |
| RMSD (y-axis, nm) | 1531 | 0.314 | 0.116 | 0.305 | 0.23 | 0.389 | 0.169 | 0.472 |
| RMSD (z-axis, nm) | 1531 | 0.32 | 0.119 | 0.309 | 0.231 | 0.397 | 0.176 | 0.476 |
| Total RMSD (nm) | 1531 | 0.568 | 0.14 | 0.568 | 0.477 | 0.658 | 0.385 | 0.746 |
| **Gel 1, Region 2** | | | | | | | | |
| Single-molecule expansion factor | 1624 | 1054.344 | 20.876 | 1056.945 | 1042.213 | 1069.255 | 1026.531 | 1078.649 |
| RMSD (x-axis, nm) | 1624 | 0.312 | 0.115 | 0.304 | 0.225 | 0.385 | 0.168 | 0.461 |
| RMSD (y-axis, nm) | 1624 | 0.314 | 0.117 | 0.304 | 0.231 | 0.386 | 0.171 | 0.47 |
| RMSD (z-axis, nm) | 1624 | 0.317 | 0.117 | 0.308 | 0.232 | 0.392 | 0.173 | 0.472 |
| Total RMSD (nm) | 1624 | 0.563 | 0.14 | 0.566 | 0.475 | 0.65 | 0.369 | 0.741 |
| **Gel 2, Region 1** | | | | | | | | |
| Single-molecule expansion factor | 523 | 1053.636 | 22.632 | 1055.618 | 1037.743 | 1071.221 | 1024.864 | 1081.227 |
| RMSD (x-axis, nm) | 523 | 0.301 | 0.116 | 0.293 | 0.219 | 0.369 | 0.16 | 0.466 |
| RMSD (y-axis, nm) | 523 | 0.303 | 0.117 | 0.292 | 0.217 | 0.382 | 0.151 | 0.452 |
| RMSD (z-axis, nm) | 523 | 0.322 | 0.137 | 0.306 | 0.222 | 0.41 | 0.165 | 0.512 |
| Total RMSD (nm) | 523 | 0.553 | 0.161 | 0.552 | 0.447 | 0.663 | 0.34 | 0.766 |
| **Gel 2, Region 2** | | | | | | | | |
| Single-molecule expansion factor | 849 | 1050.75 | 21.995 | 1052.864 | 1037.705 | 1065.365 | 1022.149 | 1077.294 |
| RMSD (x-axis, nm) | 849 | 0.296 | 0.112 | 0.289 | 0.217 | 0.367 | 0.159 | 0.445 |
| RMSD (y-axis, nm) | 849 | 0.294 | 0.114 | 0.292 | 0.21 | 0.369 | 0.15 | 0.438 |
| RMSD (z-axis, nm) | 849 | 0.302 | 0.121 | 0.287 | 0.21 | 0.378 | 0.156 | 0.462 |
| Total RMSD (nm) | 849 | 0.533 | 0.146 | 0.537 | 0.439 | 0.624 | 0.332 | 0.716 |
| **Gel 2, Region 3** | | | | | | | | |
| Single-molecule expansion factor | 497 | 1062.861 | 21.276 | 1065.479 | 1050.542 | 1077.634 | 1034.341 | 1088.583 |
| RMSD (x-axis, nm) | 497 | 0.35 | 0.139 | 0.334 | 0.25 | 0.437 | 0.184 | 0.533 |
| RMSD (y-axis, nm) | 497 | 0.338 | 0.127 | 0.33 | 0.245 | 0.419 | 0.176 | 0.505 |
| RMSD (z-axis, nm) | 497 | 0.348 | 0.133 | 0.326 | 0.251 | 0.433 | 0.193 | 0.52 |
| Total RMSD (nm) | 497 | 0.619 | 0.166 | 0.621 | 0.509 | 0.722 | 0.401 | 0.85 |
| **Gel 2, Region 4** | | | | | | | | |
| Single-molecule expansion factor | 49 | 1068.823 | 16.836 | 1070.129 | 1058.476 | 1079.936 | 1043.815 | 1088.267 |
| RMSD (x-axis, nm) | 49 | 0.366 | 0.135 | 0.363 | 0.257 | 0.46 | 0.189 | 0.532 |
| RMSD (y-axis, nm) | 49 | 0.376 | 0.129 | 0.385 | 0.27 | 0.457 | 0.207 | 0.53 |
| RMSD (z-axis, nm) | 49 | 0.383 | 0.132 | 0.385 | 0.296 | 0.443 | 0.235 | 0.534 |
| Total RMSD (nm) | 49 | 0.675 | 0.137 | 0.643 | 0.589 | 0.727 | 0.547 | 0.881 |
| **Gel 3, Region 1** | | | | | | | | |
| Single-molecule expansion factor | 153 | 1063.514 | 22.452 | 1070.043 | 1050.811 | 1079.503 | 1031.755 | 1086.738 |
| RMSD (x-axis, nm) | 153 | 0.337 | 0.121 | 0.313 | 0.257 | 0.417 | 0.192 | 0.501 |
| RMSD (y-axis, nm) | 153 | 0.344 | 0.139 | 0.345 | 0.241 | 0.435 | 0.173 | 0.517 |
| RMSD (z-axis, nm) | 153 | 0.376 | 0.151 | 0.358 | 0.261 | 0.48 | 0.191 | 0.579 |
| Total RMSD (nm) | 153 | 0.635 | 0.164 | 0.656 | 0.525 | 0.744 | 0.416 | 0.839 |
| **Gel 3, Region 2** | | | | | | | | |
| Single-molecule expansion factor | 1058 | 1059.946 | 21.327 | 1063.189 | 1046.846 | 1075.58 | 1030.574 | 1085.255 |
| RMSD (x-axis, nm) | 1058 | 0.33 | 0.124 | 0.321 | 0.24 | 0.403 | 0.18 | 0.492 |
| RMSD (y-axis, nm) | 1058 | 0.326 | 0.127 | 0.313 | 0.231 | 0.409 | 0.169 | 0.498 |
| RMSD (z-axis, nm) | 1058 | 0.345 | 0.135 | 0.329 | 0.251 | 0.437 | 0.179 | 0.521 |
| Total RMSD (nm) | 1058 | 0.6 | 0.157 | 0.596 | 0.499 | 0.701 | 0.399 | 0.801 |

**Table S15**: Summary statistics of single-molecule expansion factors (**Supplementary Figure 11C**), per-axis RMSD (**Supplementary Figure 10C**), and total RMSD (see **Supplementary Figure 9C**, boxplots; **Supplementary Figure 12C**, histograms) for all putative peptides, stratified by imaging region across Gel 1-3 (n=8 regions).

bioRxiv preprint doi: https://doi.org/10.64898/2026.05.31.729018; this version posted June 1, 2026. The copyright holder for this preprint (which was not certified by peer review) is the author/funder, who has granted bioRxiv a license to display the preprint in perpetuity. It is made available under a CC-BY 4.0 International license.

| Number of MD reference conformations | Mean RMSD (nm) | STD (nm) | Median RMSD (nm) | P25 (nm) | P75 (nm) | P10 (nm) | P90 (nm) | Minimum RMSD (nm) | Maximum RMSD (nm) | n (molecules) |
|---|---|---|---|---|---|---|---|---|---|---|
| 10 | 0.8324 | 0.2729 | 0.8793 | 0.6835 | 1.0126 | 0.3847 | 1.1421 | 0.1366 | 1.6169 | 6284 |
| 100 | 0.681 | 0.1943 | 0.7013 | 0.5686 | 0.8085 | 0.3835 | 0.91 | 0.1366 | 1.323 | 6284 |
| 200 | 0.6586 | 0.1835 | 0.6726 | 0.5523 | 0.78 | 0.3833 | 0.8787 | 0.1366 | 1.3151 | 6284 |
| 300 | 0.634 | 0.1738 | 0.6444 | 0.5299 | 0.7485 | 0.3808 | 0.8486 | 0.1366 | 1.2392 | 6284 |
| 400 | 0.6201 | 0.1683 | 0.6275 | 0.518 | 0.728 | 0.3794 | 0.8298 | 0.1366 | 1.2219 | 6284 |
| 500 | 0.597 | 0.1596 | 0.599 | 0.4974 | 0.6997 | 0.3753 | 0.7954 | 0.1366 | 1.1679 | 6284 |
| 600 | 0.5846 | 0.154 | 0.5842 | 0.489 | 0.6831 | 0.3741 | 0.7786 | 0.1366 | 1.1171 | 6284 |
| 700 | 0.5795 | 0.1525 | 0.5778 | 0.4832 | 0.6754 | 0.3724 | 0.7724 | 0.1366 | 1.1171 | 6284 |
| 800 | 0.5771 | 0.1523 | 0.575 | 0.4804 | 0.6719 | 0.3723 | 0.7712 | 0.1366 | 1.1171 | 6284 |
| 900 | 0.5747 | 0.151 | 0.5733 | 0.4788 | 0.669 | 0.3722 | 0.7668 | 0.1366 | 1.1171 | 6284 |
| 1001 | 0.5725 | 0.1509 | 0.5718 | 0.4759 | 0.6678 | 0.3715 | 0.7645 | 0.1366 | 1.1171 | 6284 |

**Table S16.** Summary statistics of total RMSD distributions as a function of the number of molecular-dynamics (MD) reference conformations (see **Supplementary Figure 13**, boxplots; **Supplementary Figure 14**, histograms).

bioRxiv preprint doi: https://doi.org/10.64898/2026.05.31.729018; this version posted June 1, 2026. The copyright holder for this preprint (which was not certified by peer review) is the author/funder, who has granted bioRxiv a license to display the preprint in perpetuity. It is made available under a CC-BY 4.0 International license.

| **Panel B** | 18x (ONE) | 50x (ONE) | 100x (ONE) | 100x (confocal) | 500x (confocal) | 1000x (confocal) | model |
|---|---|---|---|---|---|---|---|
| Mean | 4.434317761 | 5.210717151 | 5.157849609 | 6.177624755 | 3.900797385 | 7.090570252 | 4.88207877 |
| Median | 4.220428333 | 4.269811 | 4.222855 | 5.460546 | 2.408406 | 5.822544 | 4.959 |
| Std deviation | 1.991380873 | 3.786436472 | 3.851486303 | 3.813227427 | 4.826060053 | 6.186493755 | 1.419420211 |
| Std error | 0.02054499 | 0.122462107 | 0.1289573 | 6.67E-03 | 0.037042528 | 0.22819226 | 0.044886008 |
| % CV | 44.90839359 | 72.66632139 | 74.67232655 | 61.72643336 | 123.7198341 | 87.24959396 | 29.07409482 |
| 90% conf | 0.033799843 | 0.201645797 | 0.212355577 | 0.010972448 | 0.060938286 | 0.375850696 | 0.073906027 |
| 95% conf | 0.04027337 | 0.240330327 | 0.253100129 | 0.013073567 | 0.072608534 | 0.447995574 | 0.088083297 |
| 99% conf | 0.052934652 | 0.316094153 | 0.332907159 | 0.017182438 | 0.095432286 | 0.589355788 | 0.1158477 |
| N | 9395 | 956 | 892 | 326824 | 16974 | 735 | 1000 |

| **Panel F** | | | | | | | |
|---|---|---|---|---|---|---|---|
| R | 0.9255 | | Coefficient | Std. Error | t | P | VIF |
| R2 | 0.8566 | y0 | 0.2809 | 0.618 | 0.4545 | 0.6685 | 4.7978< |
| Adj R2 | 0.8279 | a | 0.809 | 0.148 | 5.4646 | 0.0028 | 4.7978< |
| Standard error of estimate | 0.7465 | | | | | | |

| **Panel G** | | | | | | | |
|---|---|---|---|---|---|---|---|
| R | 0.925 | | Coefficient | Std. Error | t | P | VIF |
| R2 | 8556 | | | | | | |
| Adj R2 | 0.8483 | y0 | 0.2613 | 0.1059 | 2.4682 | 0.0227 | 3.0004 |
| Standard error of estimate | 0.3055 | a | 0.699 | 0.0569 | 12.2836 | <0.0001 | 3.0004 |

| **Panel H** | | |
|---|---|---|
| Mean | 1.518872 | 1.161491304 |
| Median | 1.524 | 1.2634 |
| Std deviation | 1.096045975 | 0.79576071 |
| Std error | 0.219209195 | 0.165927578 |
| % CV | 72.16183952 | 68.51198169 |
| 90% conf | 0.375076408 | 0.284948583 |
| 95% conf | 0.45243455 | 0.344119637 |
| 99% conf | 0.613161459 | 0.467745354 |
| N | 25 | 23 |
| Mann-Whitney Ranksum test | P value | 0.2156 |

| **Panel I** | | |
|---|---|---|
| Mean | 0.655143478 | 0.568928571 |
| Median | 0.606 | 0.549 |
| Std deviation | 0.255672923 | 0.06306863 |
| Std error | 0.05331149 | 0.013762703 |
| % CV | 39.02548546 | 11.08550943 |
| 90% conf | 0.091552192 | 0.023739047 |
| 95% conf | 0.11056348 | 0.028709077 |
| 99% conf | 0.150283647 | 0.039162624 |
| N | 23 | 21 |
| Mann-Whitney Ranksum test | P value | 0.3827 |

**Table S17**: Statistics elements for **Fig. 2**.

bioRxiv preprint doi: https://doi.org/10.64898/2026.05.31.729018; this version posted June 1, 2026. The copyright holder for this preprint (which was not certified by peer review) is the author/funder, who has granted bioRxiv a license to display the preprint in perpetuity. It is made available under a CC-BY 4.0 International license.

| | | | | Round 1 before expansion length (cm) | Round 1 after expansion length (cm) | Round 1 expansion factor ($E_1 = C_1$) |
|---|---|---|---|---|---|---|
| | | | | 1 | 15.8 | 15.8 |
| | | | | 1 | 15.8 | 15.8 |
| | | | | 1 | 17.5 | 17.5 |
| | | | | 1 | 16.7 | 16.7 |
| | | | | 1 | 17.1 | 17.1 |
| | | | | 1 | 18.7 | 18.7 |
| **Round 2 before monomer addition length (cm)** | **Round 2 after gelation length (cm)** | **Round 2 shrinkage factor ($S_2$)** | **Round 2 before expansion length (cm)** | **Round 2 after expansion length (cm)** | **Round 2 expansion factor ($E_2$)** | **Round 2 cumulative expansion factor ($C_2$)** |
| 2.9 | 1.25 | 0.43 | 1 | 9 | 9.0 | 61.1 |
| 2.3 | 1.1 | 0.48 | 1 | 9 | 9.0 | 67.8 |
| 2.9 | 1.25 | 0.43 | 0.85 | 9 | 10.6 | 79.9 |
| 2.7 | 1.2 | 0.44 | 0.8 | 9.45 | 11.8 | 87.5 |
| 2.9 | 1.5 | 0.52 | 0.9 | 8.7 | 9.7 | 85.7 |
| 2.9 | 1.6 | 0.55 | 1 | 9.6 | 9.6 | 98.9 |
| | | | | | | |
| **Round 3 before monomer addition length (cm)** | **Round 3 after gelation length (cm)** | **Round 3 shrinkage factor ($S_3$)** | **Round 3 before expansion length (cm)** | **Round 3 after expansion length (cm)** | **Round 3 expansion factor ($E_3$)** | **Round 3 cumulative expansion factor ($C_3$)** |
| 1.4 | 0.6 | 0.43 | 1.2 | 11 | 9.6 | 250.5 |
| 1.9 | 1 | 0.53 | 1.2 | 11 | 9.6 | 341.3 |
| 2.2 | 1 | 0.45 | 1.1 | 10 | 9.1 | 330.0 |
| 2.9 | 1.6 | 0.55 | 0.9 | 9.2 | 10.2 | 493.6 |
| 1.4 | 0.65 | 0.46 | 0.9 | 8.7 | 9.7 | 384.6 |
| 2 | 1.05 | 0.53 | 1.0 | 9.4 | 9.4 | 488.0 |
| | | | | | | |
| **Round 4 before monomer addition length (cm)** | **Round 4 after gelation length (cm)** | **Round 4 shrinkage factor ($S_4$)** | **Round 4 before expansion length (cm)** | **Round 4 after expansion length (cm)** | **Round 4 expansion factor ($E_4$)** | **Round 4 cumulative expansion factor ($C_4$)** |
| 2.2 | 1.45 | 0.66 | 1.25 | 9.3 | 7.44 | 1228.2 |
| 2.1 | 1.35 | 0.64 | 1.15 | 10.3 | 8.96 | 1965.1 |
| 2.8 | 1.6 | 0.57 | 1.2 | 10.6 | 8.83 | 1665.9 |
| 2.6 | 1.5 | 0.58 | 1.15 | 9 | 7.83 | 2228.6 |
| 2.6 | 1.45 | 0.56 | 1.05 | 9 | 8.57 | 1838.6 |
| 2.5 | 1.4 | 0.56 | 1.1 | 10.2 | 9.27 | 2534.1 |

Table S18: Measured gel lengths and calculated shrinkage and expansion factors during four-network gel formation.

bioRxiv preprint doi: https://doi.org/10.64898/2026.05.31.729018; this version posted June 1, 2026. The copyright holder for this preprint (which was not certified by peer review) is the author/funder, who has granted bioRxiv a license to display the preprint in perpetuity. It is made available under a CC-BY 4.0 International license.

# Supplementary Notes

## Supplementary Note 1: 1000ExM Protocol

**Activated Monomer Solution Preparation**

1. Weigh 2.090 ± 0.002 g sodium acrylate and dissolve in 4 mL acidified Tris buffer (pH 6.8). Mix until the solution is completely clear.
2. Add 3.600 mL N,N-dimethylacrylamide to the sodium acrylate solution and mix thoroughly.
3. Prepare a 10% solution of N,N,N′,N′-tetramethylethylenediamine (90 µL water + 10 µL pure compound). Add 30 µL of this solution to the mixture and mix again until fully incorporated.
4. Filter the solution using a syringe filter (Sigma, #SLGSR33SS) and collect the clear supernatant.
5. Bubble the solution with nitrogen gas using a gas dispersion tube (Chemglass, #CG-203-01) for 10 minutes to degas while kept on ice. Alternatively, a Pasteur pipette directly connected to a nitrogen source (Sigma, #BR747725) can be used to degas at room temperature (20 °C). Both approaches work equally well.
6. Pipette 900 µL of the degassed monomer solution into an Eppendorf tube.
7. Add 30 µL potassium persulfate solution (45 mg/mL stock in water). The resulting solution is the activated monomer solution.

| Reagent | Amount in 930 µL Activated Monomer Solution (mol) | Concentration (M) | Percentage |
|---|---|---|---|
| Sodium Acrylate (AK Scientific, R624) | $2.54\times10^{-3}$ mol | 2.73 M | 25.6% (w/v) |
| N,N-Dimethylacrylamide (DMAA; Sigma, 274135) | $3.90\times10^{-3}$ mol | 4.19 M | 44.2% (v/v) |
| N,N,N′,N′-Tetramethylethylenediamine (TEMED; Sigma, T9281) | $2.28\times10^{-6}$ mol | 2.45 mM | 0.037% (v/v) |
| Potassium persulfate (KPS; Sigma, 216224) | $4.99\times10^{-6}$ mol | 5.37 mM | 0.145% (w/v) |

**To make the first gel:**

8. Add 50 µL activated monomer solution onto a hydrophobic microscope slide (CytoSlide Fluorosilane, CYTONIX) in a Tupperware container.
9. Place a coverslip with the attached sample (proteins or cells) on top, with the biological sample face down.
10. Place the gel chamber inside a sealable plastic container (e.g., Tupperware). Before beginning any procedures, modify the lid to contain two small holes: one inlet and one outlet. Insert a pipette tip (connected via rubber tubing to a nitrogen line) into the inlet hole to deliver nitrogen gas. Leave the second hole open to serve as a vent, allowing air to escape as nitrogen fills the container.
11. Purge with nitrogen for 1 hour to displace oxygen. After purging, remove the nitrogen line and seal both holes with tape to maintain a nitrogen atmosphere. Allow the gel to polymerize overnight (≥8 hours).
12. Immerse the gel in excess deionized water at room temperature (~20 °C) for 3 hours to remove unreacted components and allow expansion. Replace the water at least twice, continuing exchanges as needed until full ~18× linear expansion is reached.

**To make an iterative gel throughout an expanded gel:**

13. Place each expanded gel piece into a 6-well plate.

bioRxiv preprint doi: https://doi.org/10.64898/2026.05.31.729018; this version posted June 1, 2026. The copyright holder for this preprint (which was not certified by peer review) is the author/funder, who has granted bioRxiv a license to display the preprint in perpetuity. It is made available under a CC-BY 4.0 International license.

14. Add 900 μL degassed monomer solution and 30 μL potassium persulfate solution (45 mg/mL stock) to each well to make the activated monomer solution.
15. Position the plate at a 30–45° angle in a Tupperware container so that gels are fully immersed from the top, bottom, and sides (critical).
16. Degas the Tupperware with nitrogen gas for 10 minutes, then place on a shaker for 35 minutes to incubate.
17. Remove gels quickly. Place each gel onto a hydrophobic glass slide with the sample facing down and immediately cover with a coverslip (within 10 seconds) to minimize oxygen exposure.
18. Purge the Tupperware with nitrogen gas for 1 hour, then allow the gel to form overnight (≥8 hours).

**To reach the maximum iterative expansion capacity:**

19. After the initial gel, up to three additional rounds of iterative gel casting can be performed. The second round yields ~100× expansion, the third round ~500×, and the fourth round ~1500×.
20. For the 100×, 500×, and 1500× gels, immerse in excess deionized water at room temperature (~20 °C) overnight. Replace the water at least twice, continuing exchanges as needed until full expansion is achieved.

## Supplementary Note 2: Engineering Ultra-High Expansion Factor Gels

### Protocol v1: Iterative ExM with acrylamide re-embedding

Composition and expansion factors:

Network 1 (ionic): 8.625% (w/v) sodium acrylate, 2.5% (w/v) acrylamide, 0.075% (w/v) Bis, APS 0.2% (w/v), TEMED 0.2% (w/v), 4-hydroxy-TEMPO 0.01% (w/v). Original ExM hydrogel [6].
Network 1 expands ~4×.

Network 2 (neutral): 13.75% (w/v) acrylamide, 0.038% (w/v) Bis, APS 0.025% (w/v), TEMED 0.025% (w/v). Re-embedding gel from the ExR paper [12].
Network 2 is polymerized within the expanded Network 1, forming a two-network interpenetrating gel. The composite contracts to ~3.5×.

Network 3 (ionic): Same composition as Network 1.
Network 3 is polymerized within the Network 1+2 interpenetrating gel (three-network composite). Upon swelling, it expands ~4× relative to the contracted ~3.5× state (total ~14×).

Network 4 (neutral): Same composition as Network 2.
Network 4 is polymerized within the three-network composite, forming a four-network interpenetrating gel. The structure contracts slightly to ~13×.

Network 5 (ionic): Same composition as Network 1.
Network 5 is polymerized within the four-network composite (**final ~52x linear expansion**).

Notes:

The final five-network interpenetrating gel is extremely dense. During HCR testing, fluorophore-labeled DNA oligos (including Alexa 488–labeled HCR hairpins) could not be removed by washing and remained physically trapped within the gel mesh. This likely reflects a very small effective pore size combined with significant topological entanglement that restricts diffusion of unbound oligos.

bioRxiv preprint doi: https://doi.org/10.64898/2026.05.31.729018; this version posted June 1, 2026. The copyright holder for this preprint (which was not certified by peer review) is the author/funder, who has granted bioRxiv a license to display the preprint in perpetuity. It is made available under a CC-BY 4.0 International license.

## Protocol v2: Iterative ExM with less dense acrylamide re-embedding

Rationale:
Protocol v1 was too dense and trapped unbound oligos. Protocol v2 reduces neutral network density to increase pore size and improve washout while maintaining multi-round expansion.

Composition and expansion factors:
Network 1 (ionic): 8.625% (w/v) sodium acrylate, 2.5% (w/v) acrylamide, 0.075% (w/v) Bis, APS 0.2% (w/v), TEMED 0.2% (w/v), 4-hydroxy-TEMPO 0.01% (w/v). Original ExM formulation.
Network 1 expands ~4×.

Network 2 (neutral): 2.85% (w/v) acrylamide, 0.15% (w/v) Bis, APS 0.075% (w/v), TEMED 0.075% (w/v).
Re-embedding using the ExSeq approach [22].
Polymerized within expanded Network 1; composite contracts to ~3.3×.

Network 3 (ionic): Same composition as Network 1.
Polymerized within the two-network IPN; expands ~4× from ~3.3× (total ~13×).

Network 4 (neutral): Same composition as Network 2.
Polymerized within the three-network composite; contracts to ~11×.

Network 5 (ionic): Same composition as Network 1.
Polymerized within the four-network composite (**final ~44x linear expansion).**

Notes:
In Protocol v2, reduced neutral network density increases effective pore size and permits diffusion of unbound antibodies and oligonucleotides during washing, reducing molecular trapping. Because the re-embedding gel is less dense, it retains less of the expanded state during each contraction step, resulting in a modest reduction in total linear expansion (52× → ~44×).

## Protocol v3: Iterative DMAA gel (complete polymerization, overnight gelation) with acrylamide re-embedding

Rationale:
Instead of using Bis as the crosslinker (original ExM formulation), Protocol v3 adopts a DMAA-based self-crosslinking architecture [54] to generate highly stretchable, high-expansion hydrogels. The swellable network is based on the SA/DMAA formulation with the highest reported single-round expansion [55]. Gelation is allowed to proceed to completion (overnight) rather than relying on partial polymerization. Complete polymerization improves mechanical robustness for iterative processing but reduces single-round expansion to ~10× compared to the reported ~20× achieved under partial polymerization conditions.

Composition and expansion factors:
Network 1 (SA/DMAA): SA 0.522 g ml$^{-1}$ in acidified Tris buffer (10% (v/v) 1 M Tris-HCl pH 8 + 20% (v/v) 1.2 M HCl in water); DMAA 900 μl ml$^{-1}$; TEMED 7.5 μl of 10% (v/v) stock per ml; KPS (45 mg ml$^{-1}$ stock) added at 20 μl per 411 μl gel solution. Overnight (>8 h) gelation. 20ExM hydrogel [55].
Network 1 expands ~10×.

Network 2 (neutral): 2.85% (w/v) acrylamide, 0.15% (w/v) Bis, APS 0.075% (w/v), TEMED 0.075% (w/v).

bioRxiv preprint doi: https://doi.org/10.64898/2026.05.31.729018; this version posted June 1, 2026. The copyright holder for this preprint (which was not certified by peer review) is the author/funder, who has granted bioRxiv a license to display the preprint in perpetuity. It is made available under a CC-BY 4.0 International license.

Contracts to ~8×.

Network 3 (SA/DMAA): Same as Network 1.
Expands ~6× from ~8× (total ~48×).

Network 4 (neutral): Same as Network 2.
Contracts to ~24×.

Network 5 (SA/DMAA): Same as Network 1.
Expands ~4× from ~24× (**final ~96× linear expansion**).

Notes:
Overnight polymerization reduces single-round expansion but enables ~96× total linear expansion through iteration.

### Protocol v4: Iterative DMAA gel with cleavable acrylamide/DATD re-embedding

Rationale:
Acrylamide re-embedding gels act as a rigid, non-swellable scaffold that preserves the expanded state but limits subsequent swelling. Protocol v4 replaces the permanent re-embedding scaffold with a cleavable neutral acrylamide/DATD network: the neutral network is used to retain the expanded size during handling and casting of the next swellable DMAA gel, then cleaved to release the constraint and allow the charged DMAA network to fully swell.

Composition and expansion factors:
Network 1 (SA/DMAA): Same as Protocol v3.
Network 1 expands ~10×.

Network 2 (neutral, cleavable re-embedding): 10% (w/v) acrylamide, 5% (w/v) DATD, TEMED 0.05% (w/v), APS 0.05% (w/v).
Network 2 is polymerized within expanded Network 1; composite contracts to ~8×.

Network 3 (SA/DMAA): Same as Network 1.
Cast within the Network 1+2 composite; structure contracts to ~6.5×.
Cleave Network 2 by placing the composite gel in DATD cleavage solution (50 mM sodium metaperiodate in 0.1 M sodium acetate buffer).
Network 3 expands ~8× (total ~52×).

Network 4 (neutral, cleavable re-embedding): Same as Network 2.
Cast within the expanded Network 3 composite; structure contracts to ~42×.

Network 5 (SA/DMAA): Same as Network 1.
Cast within the Network 3+4 composite; structure contracts to ~34×.
Cleave Network 4.
Network 5 expands ~8× (**final ~270× linear expansion**).

Result:

bioRxiv preprint doi: https://doi.org/10.64898/2026.05.31.729018; this version posted June 1, 2026. The copyright holder for this preprint (which was not certified by peer review) is the author/funder, who has granted bioRxiv a license to display the preprint in perpetuity. It is made available under a CC-BY 4.0 International license.

Cleavable re-embedding increases total expansion by releasing the neutral scaffold after each casting step, allowing the charged DMAA networks to swell more fully. Protocol v3 achieves ~96× linear expansion, whereas Protocol v4 reaches ~270×, solely by replacing permanent re-embedding with cleavable DATD re-embedding.

### Protocol v5: Iterative DMAA gel with optimized cleavable acrylamide/DATD re-embedding

Rationale:
Minimizing contraction during each re-embedding step preserves more of the expanded state and therefore enables higher overall expansion factors. The acrylamide/DATD re-embedding composition was optimized to reduce shrinkage relative to Protocol v4.

Composition and expansion factors:
Network 1 (SA/DMAA): Same as Protocol v3.
Network 1 expands ~10×.

Network 2 (neutral, cleavable re-embedding; optimized):
5% (w/v) acrylamide, 1% (w/v) DATD, TEMED 0.050% (w/v), APS 0.025% (w/v).
Network 2 is polymerized within expanded Network 1; composite contracts to ~9×.

Network 3 (SA/DMAA): Same as Network 1.
Cast within the Network 1+2 composite; structure contracts to ~7.5×.
Cleave Network 2.
Network 3 expands ~8× (total ~60×).

Network 4 (neutral, cleavable re-embedding): Same as Network 2.
Cast within expanded Network 3; contraction is minimal (~60× retained).

Network 5 (SA/DMAA): Same as Network 1.
Cast within the Network 3+4 composite; structure contracts to ~48×.
Cleave Network 4.
Network 5 expands ~8× (**final ~384× linear expansion**).

Optimization strategy:
Starting from the Protocol v4 composition of 10% (w/v) acrylamide, 5% (w/v) DATD, TEMED 0.05% (w/v), and APS 0.05% (w/v), multiple parameter ranges were tested. DATD was varied from 0.5% to 5%, TEMED from 0.025% to 0.25%, and APS from 0.025% to 0.05%. The optimized formulation that minimized contraction was 5% (w/v) acrylamide, 1% (w/v) DATD, TEMED 0.050% (w/v), and APS 0.025% (w/v).

Result:
By reducing shrinkage relative to Protocol v4, total linear expansion increases from ~270× to ~384×.

### Protocol v6: Iterative SA/DMAA gel with cleavable DATD re-embedding (optimized SA/DMAA formulation)

Rationale:

bioRxiv preprint doi: https://doi.org/10.64898/2026.05.31.729018; this version posted June 1, 2026. The copyright holder for this preprint (which was not certified by peer review) is the author/funder, who has granted bioRxiv a license to display the preprint in perpetuity. It is made available under a CC-BY 4.0 International license.

This protocol uses an optimized SA/DMAA swellable gel formulation to increase single-round expansion under complete (overnight) polymerization by systematically reducing initiator concentration (KPS 0.05–0.5% w/v, step 0.05).
The equilibrium swelling of a polyelectrolyte gel is governed by the balance between ionic osmotic pressure and network elasticity (Flory–Rehner framework). At fixed charge density, increasing linear expansion requires lowering the elastic restoring force of the network. Reducing initiator concentration decreases radical flux during polymerization, which lowers the number of active chain starts and increases the average strand length between crosslinks. Longer strands reduce effective crosslink density and elastic modulus, directly increasing the equilibrium swelling ratio.
Slower polymerization kinetics also delay gelation, allowing more uniform monomer distribution prior to network percolation and reducing formation of locally over-crosslinked regions that mechanically restrict swelling. In addition, rapid radical generation can freeze in topological constraints that function as additional elastic elements; lowering radical flux minimizes these constraints.
Thus, these effects decrease elastic resistance while maintaining full monomer conversion under overnight gelation, enabling higher single-round linear expansion without relying on partial curing.

Composition and expansion factors:
Network 1 (optimized SA/DMAA gel): 25.6% (w/v) sodium acrylate, 44.2% (v/v) DMAA, 0.037% (v/v) TEMED, 0.145% (w/v) KPS. Overnight gelation.
Network 1 expands ~18×.

Network 2 (neutral, cleavable re-embedding): 10% (w/v) acrylamide, 5% (w/v) DATD, TEMED 0.05% (w/v), APS 0.05% (w/v).
Composite contracts to ~11×.

Network 3 (SA/DMAA): Same as Network 1.
Structure contracts to ~8.5×. Cleave Network 2.
Network 3 expands ~10× (total ~85×).

Network 4 (neutral, cleavable): Same as Network 2.
Structure contracts to ~60×.

Network 5 (SA/DMAA): Same as Network 1.
Structure contracts to ~42×. Cleave Network 4.
Network 5 expands ~10× (**final ~420× linear expansion**).

Result:
The optimized SA/DMAA formulation increases single-round expansion under complete polymerization (~18×). Combined with cleavable re-embedding, total expansion reaches ~420×.

### Protocol v7: Iterative SA/DMAA gel with optimized cleavable DATD re-embedding (optimized SA/DMAA formulation)

Rationale:
Protocol v7 combines the optimized SA/DMAA swellable gel with an optimized cleavable DATD re-embedding formulation that minimizes contraction during casting. Reducing shrinkage at each iteration step preserves more of the expanded state, allowing the higher per-round expansion to accumulate more efficiently.

bioRxiv preprint doi: https://doi.org/10.64898/2026.05.31.729018; this version posted June 1, 2026. The copyright holder for this preprint (which was not certified by peer review) is the author/funder, who has granted bioRxiv a license to display the preprint in perpetuity. It is made available under a CC-BY 4.0 International license.

Composition and expansion factors:
Network 1 (optimized SA/DMAA gel): 25.6% (w/v) sodium acrylate, 44.2% (v/v) DMAA, 0.037% (v/v) TEMED, 0.145% (w/v) KPS. Overnight gelation.
Network 1 expands ~18×.

Network 2 (neutral, optimized cleavable re-embedding): 5% (w/v) acrylamide, 1% (w/v) DATD, TEMED 0.050% (w/v), APS 0.025% (w/v).
Composite contracts to ~12.5×.

Network 3 (SA/DMAA): Same as Network 1.
Structure contracts to ~9×. Cleave Network 2.
Network 3 expands ~10× (total ~90×).

Network 4 (neutral, optimized): Same as Network 2.
Structure contracts to ~77×.

Network 5 (SA/DMAA): Same as Network 1.
Structure contracts to ~71.8×. Cleave Network 4.
Network 5 expands ~10× (**final ~718× linear expansion**).

Result:
Minimizing contraction in combination with the optimized SA/DMAA swellable network increases total expansion from ~420× (v6) to ~718×. Also, when the single network SA/DMAA gel is placed in the DATD cleavage solution (50 mM sodium metaperiodate in 0.1 M sodium acetate buffer), the gel partially dissolves. This indicates that metaperiodate treatment is not fully specific to DATD-containing networks and can compromise the integrity of SA/DMAA gels as well.

### (1000ExM) Protocol v8: Four rounds of sequential interpenetrating charged gels using the optimized SA/DMAA formation

Rationale:
Test whether a neutral re-embedding scaffold is required to retain the expanded state during casting. Instead of alternating swellable and re-embedding networks, sequentially cast interpenetrating charged SA/DMAA gels and determine whether the charged network itself can preserve sufficient structure through each casting/shrink step.

Composition and expansion factors:
Network 1 (optimized SA/DMAA): 25.6% (w/v) sodium acrylate, 44.2% (v/v) DMAA, 0.037% (v/v) TEMED, 0.145% (w/v) KPS. Overnight gelation.
Network 1 expands ~18×.

Network 2 (optimized SA/DMAA): Same as Network 1.
Cast within expanded Network 1; structure contracts to ~10×.
Network 2 expands to ~100×.

Network 3 (optimized SA/DMAA): Same as Network 1.
Cast within expanded Network 2 composite; structure contracts to ~50×.

bioRxiv preprint doi: https://doi.org/10.64898/2026.05.31.729018; this version posted June 1, 2026. The copyright holder for this preprint (which was not certified by peer review) is the author/funder, who has granted bioRxiv a license to display the preprint in perpetuity. It is made available under a CC-BY 4.0 International license.

Network 3 expands to ~500×.

Network 4 (optimized SA/DMAA): Same as Network 1.
Cast within expanded Network 3 composite; structure contracts to ~250×.
Network 4 expands ~6× (**final ~1500× linear expansion**).

Result:
Sequential casting of interpenetrating charged SA/DMAA gels is feasible. The expanded gel contracts from ~18× to ~10× during monomer infiltration, yet remains sufficiently swollen to permit formation of a subsequent charged network. Iterative casting yields ~1500× total linear expansion without intermediate neutral re-embedding.
While high ionic strength is known to reduce swelling in polyelectrolyte networks, ionic-in-ionic casting was generally expected to fail because the charged monomer solution was presumed to collapse the expanded gel back toward its original, pre-expanded state [55,56]. Contrary to this expectation, the network does not fully collapse but instead contracts only partially (~18× → ~10×), consistent with a balance between ionic (Donnan) osmotic pressure and nonlinear network elasticity [57], thereby enabling polymerization of an additional charged network within the partially contracted hydrogel.

## Supplementary Note 3: GFP 3D Reconstruction

Raw fluorescence images acquired from One-step Nanometer-scale Expansion (ONE) microscopy [5] are processed to isolate individual instances before reconstruction. For segmentation, we develop an unsupervised framework based on graph neural networks. As shown in **Supplementary Figure 24**, each image is divided into transformer derived [59] patches, that serve as local descriptors of the underlying signal. As shown in **Supplementary Figure 25**, the resulting feature vectors are embedded into a weighted graph whose edges encode patch similarity. Cluster assignments are obtained by minimizing a correlation clustering objective, $L_{CC}(S)=-\sum_{i,j}W_{ij}\sum_{c}S_{ic}S_{jc}$, where $S_{ic} \in 0,1$ denotes the assignment of node i to cluster c. As shown in **Supplementary Figure 26**, foreground and background separation is achieved in a two-stage process, ensuring robust isolation of protein complexes. Each segmented particle is extracted as a fixed-size sub-image $I_{0.}$ Based on connected component analysis and physical constraint-based filtering, we are performing partitions around mediods [60] to segregate the similar identity objects from the foreground mixed population. As shown in **Supplementary Figure 27**, this formulation encourages patches with similar appearance to co-cluster, yielding segmentation masks that isolate individual protein complexes for downstream analysis.

To enhance the structural signal of each isolated complex, we apply a physics-informed deconvolution [61] procedure in polar coordinates. Given an observed blurred image $I_0(r,\theta)$, the estimate $I_k(r,\theta)$ at iteration k, is iteratively refined according to $I_{k+1}(r,\theta)=I_k(r,\theta).I_0(r,\theta)/\{(I_k*R)(r,\theta)\}*R^{flip}(r,\theta)]$, where $R^{flip}(r,\theta)$ is the flipped PSF kernel, * is the convolution in polar coordinates. This formulation improves recovery of fine structural details compared to Gaussian PSF-based Lucy-Richardson deconvolution.

After normalization, the resized image is then processed by an encoder network $\Psi_\theta(X)$ as shown in **Supplementary Figure 28**, which extracts a compact latent representation of the underlying molecular state. This encoder maps the normalized image to a low-dimensional latent variable: $\Psi_\theta(X)=(R,t,s,z)$, where $R \in SO(3)$ is a rotation matrix defining particle orientation, $t \in R^2$ is a translation vector, $s > 0$ is a scaling factor accounting for gel expansion variability and $z \in R^d$ encodes molecular conformation.

As shown in **Supplementary Figure 28**, the decoder $V_\epsilon$ acts as a continuous implicit representation of the 3D density in Fourier space, mapping frequency coordinates and conformation codes to Fourier coefficients:

bioRxiv preprint doi: https://doi.org/10.64898/2026.05.31.729018; this version posted June 1, 2026. The copyright holder for this preprint (which was not certified by peer review) is the author/funder, who has granted bioRxiv a license to display the preprint in perpetuity. It is made available under a CC-BY 4.0 International license.

$V_\epsilon(k,z) \rightarrow X'(k;z)$, $X'(k;z) \in R$, where $k=(k_x,k_y,k_z)^T$ is a 3D frequency coordinate and z is the latent conformation vector. Thus, $V_\epsilon$ defines the Fourier space representation of the protein complex.

To generate a 2D projection, the decoder is evaluated slice-by-slice on a grid of frequency coordinates corresponding to the central slice through the 3D Fourier volume, rotated according to the estimated pose: $k'_{grid}=s^{-1} \cdot Rk_{grid}$, $P'_c=V_\epsilon(k'_{grid},z)$, $P'(u)=P'_c(u) \cdot e^{-2\pi i(uxtx+uyty)}$ . Here, $k_{grid}$ are the 2D frequency coordinates in the projection plane, $k'_{grid}$ are these coordinates rotated into the volume's frame, $P'_c$ is the centered Fourier slice decoded from the density field, and $P'(u)$ is the final Fourier slice after applying the translation phase shift.

Model optimization is performed by comparing this predicted Fourier slice to the Fourier transform of the target image. The target image is transformed to the Fourier domain, $Y'=F[X]$. The symmetric mean squared error loss is minimized through the equation: $L\ (P',\ Y') = \min\ (\|P'-Y'\|^2_2,\ \|P'-F[flip(X)]\|^2_2)$. The overall training objective is $\min_{\theta,\epsilon} E_{X\sim data}[L(P'(X;\Psi_\theta,V_\epsilon);Y')]$, where θ are the encoder parameters and ϵ are the decoder parameters.

Following training, the generator is evaluated slice-by-slice across a full grid of frequency coordinates to build a complete 3D Fourier volume. An inverse Fourier transform then produces a volumetric density map that represents a physically plausible 3D molecular structure obtained without imposing predefined atomic coordinates or using external templates or supervision. The resulting volumetric data is saved in MRC format, and final reconstructions were visualized and analyzed using UCSF ChimeraX [62].
